# Revision of the Mesoamerican species of *Calolydella* Townsend (Diptera: Tachinidae) and description of twenty-three new species reared from caterpillars in Area de Conservación Guanacaste, northwestern Costa Rica

**DOI:** 10.3897/BDJ.6.e11223

**Published:** 2018-02-02

**Authors:** AJ Fleming, D. Monty Wood, M. Alex Smith, Winnie Hallwachs, Daniel H Janzen

**Affiliations:** 1 Agriculture Agri-Food Canada, Rm. 3099I, 960 Carling Avenue, Ottawa, Ontario, Canada; 2 Department of Integrative Biology, Guelph University, Guelph, Ontario, Canada; 3 Department of Biology, University of Pennsylvania, Philadelphia, Pennsylvania 19104, United States of America

**Keywords:** Blondeliini, caterpillars, cloud forest, dry forest, fly, Neotropics, parasitoid, rain forest

## Abstract

**Background:**

Twenty-three new species of the genus *Calolydella* Townsend, 1927 (Diptera: Tachinidae) are described, all reared from multiple species of wild-caught caterpillars across a wide variety of families (Lepidoptera: Crambidae; Erebidae; Geometridae; Hesperiidae; Lycaenidae; Nymphalidae; Pieridae; Riodinidae; and Sphingidae). All caterpillars were collected within Area de Conservación Guanacaste (ACG), in northwestern Costa Rica. This study provides a concise description of each new species using morphology, life history, molecular data, and photographic documentation. In addition to the new species, we also provide a generic redescription and revised key to species of the genus *Calolydella* from Central and South America.

**New information:**

The following 23 new species of *Calolydella* are described by Fleming and Wood: *C. adelinamoralesae* **sp. n.**, *C. alexanderjamesi* **sp. n.**, *C. argentea* **sp. n.**, *C. aureofacies* **sp. n.**, *C. bicolor* **sp. n.**, *C. bifissus* **sp. n.**, *C. crocata* **sp. n.**, *C. destituta* **sp. n.**, *C. discalis* **sp. n.**, *C. erasmocoronadoi* **sp. n.**, *C. felipechavarriai* **sp. n.**, *C. fredriksjobergi* **sp. n.**, *C. inflatipalpis* **sp. n.**, *C. interrupta* **sp. n.**, *C. nigripalpis* **sp. n.**, *C. omissa* **sp. n.**, *C. ordinalis* **sp. n.**, *C. renemalaisei* **sp. n.**, *C. susanaroibasae* **sp. n.**, *C. tanyadapkeyae* **sp. n.**, *C. tenebrosa* **sp. n.**, *C. timjamesi* **sp. n.**, *C. virginiajamesae* **sp. n.** *Lydella frugale* Curran, 1934 is proposed as a new synonym of *Pygophorinia peruviana* Townsend, 1927, **syn. n.**, under the combination *Calolydella frugale* (Curran, 1934), **comb. n.**

## Introduction

The New World genus *Calolydella* Townsend, 1927 (Tachinidae: Blondeliini) was erected for one male and several female specimens collected by Townsend himself at Itaquaquecetuba (now a suburb of São Paolo), Brazil and described as *Calolydella geminata* Townsend, 1927. When Wood (1985) revised the tribe Blondeliini, he synonymized three other genera under *Calolydella*: *Olindopsis* Townsend, 1927, *Pygophorinia* Townsend, 1927, and *Prodexodes* Townsend, 1927. These synonymies raised the number of species in the genus from one to 13 (Wood 1985).

Here, we describe 23 new species of *Calolydella* reared from wild-caught caterpillars collected as part of the ongoing inventory of Lepidoptera larvae (and their parasitoids) in the terrestrial portion of Area de Conservación Guanacaste (ACG), an area of 120,000 hectares in northwestern Costa Rica (Janzen et al. 2009, Janzen and Hallwachs 2016). Each new species is distinguished by morphological differences, differences in CO1 (cox1 or cytochrome oxidase 1) gene sequences, and comparison (by DMW and AJF) with previously described species of *Calolydella*. Although many of the species of *Calolydella* are common throughout the Neotropics (Wood 1985), none of the previously known species have been found in ACG to date. Individual and comparative details of the parasitization ecology of these flies will be treated in later papers, once the overall knowledge of the caterpillar-attacking tachinids found in ACG is more complete (Smith et al. 2006, Smith et al. 2007, Smith et al. 2008, Smith et al. 2012, Fleming et al. 2014a, Fleming et al. 2014b, Fleming et al. 2015a, Fleming et al. 2015b, Fleming et al. 2015c, Fleming et al. 2015d, Fleming et al. 2016a, Fleming et al. 2016b).

## Materials and methods

All flies and rearing information described herein were collected as part of the ongoing ACG inventory of caterpillars, their food plants, and their parasitoids, throughout the three major ACG terrestrial ecosystems (Janzen et al. 2009, Janzen and Hallwachs 2011, Janzen and Hallwachs 2015, Janzen and Hallwachs 2016). The parasitoid rearing methods are described at http://janzen.bio.upenn.edu/caterpillars/methodology/how/parasitoid_husbandry.htm. Since its inception in 1978, this inventory has reared more than 750,000 wild-caught caterpillars. All frequencies of parasitization reported here need to be considered against this background inventory.

Our present treatment of the genus *Calolydella* is limited to species from the Mesoamerican region, from the Mexican provinces of Guerrero and Oaxaca to the Colombian border with Panama. While we included all known species in our comparisons with the new species, only the species distributed within this region are included in our key.

### Acronyms for depositories

AMNH American Museum of Natural History, New York, USA

NHMUK Natural History Museum [formerly British Museum (Natural History)], London, UK

CNC Canadian National Collection of Insects, Arachnids and Nematodes, Ottawa, Canada

MNCR Museo Nacional de Costa Rica [formerly Instituto Nacional de Biodiversidad – INBio], Santo Domingo de Heredia, Costa Rica

NMW Naturhistorisches Museum Wien, Vienna, Austria

USNM National Museum of Natural History [formerly United States National Museum], Washington, D.C., USA

### Voucher specimen management

The management of voucher specimens has been detailed in previous papers in this series (Janzen and Hallwachs 2016). In brief, caterpillars reared from the ACG inventory each receive a unique voucher code in the format yy–SRNP–xxxxx. Parasitoids emerging from these caterpillars receive the same voucher code, indicating the rearing event; when/if individually processed for DNA barcoding, each parasitoid receives a second voucher code unique to it, in the format DHJPARxxxxxxx. The associated data for each voucher code are available at http://janzen.bio.upenn.edu/caterpillars/database.lasso.

All DHJPARxxxxxxx-coded tachinids had one leg removed for DNA barcoding and couriered to the Center for Biodiversity Genomics of the Biodiversity Institute of Ontario (BIO) at the University of Guelph (Canada). All associated data and successful barcodes are permanently and publicly deposited in the Barcode of Life Data System (BOLD) (Ratnasingham and Hebert 2007), and a select set of these data are subsequently migrated to GenBank. Each barcoded specimen also receives accession numbers from the Barcode of Life Data System (BOLD) and GenBank, respectively. The dynamic nature of the inventory means that it is continually adding new specimens; newly added *Calolydella* specimens can be found by searching for the genus in BOLD.

All inventoried specimens discussed herein were collected under Costa Rican government research permits issued to DHJ; the Tachinidae samples were exported under permit by DHJ from Costa Rica to their final depository in the CNC. Tachinid identifications for the inventory are done by DHJ in coordination with a) visual inspection of morphology by AJF and DMW, b) DNA barcoding by MAS and BIO, and c) databasing and association with host caterpillars by DHJ and WH through the inventory itself.

The date of capture of fly specimens is the date of eclosion of the fly and not the date of capture of the caterpillar. The fly eclosion date is much more representative of the time when that species is on the wing than is the date of capture of the parasitized caterpillar. The “collector” is the parataxonomist who collected the caterpillar, rather than the person who later retrieved the newly eclosed fly and processed it by freezing, pinning, labeling and oven-drying. The biology and parasitization rates of individual tachinid species will be the subject of later papers. The holotypes of the new species described herein are deposited in the Diptera collection of the CNC, Ottawa, Canada.

### Imaging

Habitus and terminalia photographs were taken using the methods outlined in Fleming et al. (2014a). Raw image files were first processed with Adobe Photoshop CS6, then digitally stacked to produce a final composite image using Zerene Stacker Software v1.04.

Adult flies were dissected following standard practice (O’Hara 1983). The morphological terminology follows Cumming and Wood (2009). If only one male was available, it was designated as the holotype and not subjected to dissection.

### Interim names for undescribed host species

Names of undescribed host species follow a standardized, interim naming system used for taxonomic units considered to be distinct species and identified by a combination of DNA barcode, morphology, and host-plant information. The interim names are given in the format "*Eois* Janzen52", where the species epithet is composed of the name of the taxonomist who first singled out the species and a number. This prevents confusion with already described species while maintaining traceability of each undescribed species within the ACG project.

### DNA barcoding

We analyzed DNA barcodes (the 5’ region of the cytochrome c oxidase I (CO1) gene, Hebert et al. 2004), where available, for all specimens of ACG *Calolydella*. The 658 bp DNA barcodes were amplified from total DNA extracts obtained from single legs with a standard glass fibre protocol, using the standard insect primers (LepF1–LepR1) (Ivanova et al. 2006,Smith et al. 2006, Smith et al. 2007, Smith et al. 2008).

## Taxon treatments

### Calolydella

Townsend, 1927

Calolydella Townsend, 1927a: 278. Type-species: *Calolydella geminata* Townsend, 1927, by original designation.
Olindopsis Townsend, 1927a: 274. Type-species: *Olindopsis andinensis* Townsend, 1927, by original designation. Synonymy proposed by Wood (1985).
Pygophorinia Townsend, 1927a: 274. Type-species: *Pygophorinia peruviana* Townsend, 1927, by original designation. Synonymy proposed by Wood (1985).
Prodexodes Townsend, 1927a: 280. Type-species: *Prodexodes rufiventris* Townsend, 1927, by original designation. Synonymy proposed by Wood (1985).
Calolydella Calolydella geminata Townsend, 1927

#### Description

*Calolydella* belongs to the tribe Blondeliini. Wood (1985) defined the tribe by thethe following combination of character states: prosternum setose; first postsutural supra-alar seta smaller than first postsutural dorsocentral seta; bend of vein M rounded, obtuse; subapical scutellar setae long, stout, and divergent, their bases situated close together; wing veins R_4+5_ and M ending at or near wing tip; often elongate and narrow body plan; mid-dorsal depression of syntergite 1+2 (ST1+2) rarely reaching hind margin of syntergite; mid tibia, in many genera, with a single long anterodorsal seta.

The following redescription applies to both males and females of *Calolydella*; any differences between the sexes are noted. **Head**: males lacking proclinate orbital setae, females with 2 pairs of proclinate orbital setae; first reclinate orbital seta either equal to or longer than uppermost frontal seta; ocellar setae long; eye bare, if haired then hairs minute and inconspicuous; parafacial bare; facial margin level with vibrissa and not visible in profile, with a few small supra-vibrissal setae; subvibrissal ridge short, with three or fewer setae; margin of postgena straight or slightly convex anteriorly, genal dilation weak or absent; palps normally slender and yellow, some species with black or inflated palps; postpedicel of similar length in both sexes; arista minutely pubescent, slightly thickened at base. **Thorax**: prosternum setose; proepisternum bare; postpronotum with 2–4 setae in a narrow triangle or a straight line (rarely inner basal seta absent); acrostichal setae 1–3:3–4; dorsocentral setae 1–3:2–3; intra-alar setae 1–3:2–3; supra-alar setae 1–2:3; katepisternal setae 2–3 [Wood (1985) described the genus as having three katepisternal setae; however, we show that this trait can vary in females]; lateral scutellar setae shorter than subapical setae and curved medially; fore tibia with one posterior seta; mid tibia with one anterodorsal seta; wings ranging from smoky gray to pale translucent brown; wing vein R_4+5_ with 2–7 setulae dorsally at base, these sometimes extending to crossvein R-M. **Abdomen**: ST1+2 with one pair of median marginal setae, mid-dorsal depression not extending to hind margin of syntergite; 3rd and 4th abdominal tergites with 1–2 pairs of discal setae. Abdominal tergites marginally banded with gold pollinosity; in some species banding interrupted by a dark median stripe; male abdomen elongated, conical, tapering to a sharp point; in females, abdomen rounded and downwardly curved; in some species the coloration and pattern of pollinosity are sexually dimorphic.

**Other species included in *Calolydella* Townsend**

*andinensis* Townsend, 1927: 339 (*Olindopsis*). Holotype female (USNM), by original designation [examined by DMW]. Type locality: Peru, Cordillera Oriental, near Tabalosas, 3000ft.

*blandita* Wulp, 1890: 142 (*Hypostena*). Lectotype male (NHMUK), by fixation of Wood (1985:28). Type locality: Mexico, Guerrero, Sierra de las Aguas Escondidas, 7000ft.

*cingulata* Schiner, 1868: 327 (*Meigenia*). Holotype male (NMW), by original designation [examined by DMW]. Type locality: Brazil.

*cylindriventris* Wulp, 1890: 145 (*Hypostena*). Lectotype male (NHMUK), by fixation of Wood (1985:28). Type locality: Mexico, Guerrero, Sierra de las Aguas Escondidas, 7000ft.

*geminata* Townsend, 1927: 293 (*Calolydella*). Multiple syntypes (USNM) [1 female and several males examined by DMW]. Type locality: Brazil, São Paulo, Itaquaquecetuba.

*gentica* Walker, 1860: 302 (*Masicera*). Lectotype male (NHMUK), by fixation of Wood (1985:28) (examination of “Holotype: ♂” from Mexico in NHMUK is regarded as a lectotype fixation [JE O'Hara, pers. comm.]) [examined by DMW]. Type locality: Mexico.

*lathami* Curran, 1925: 284 (*Lydella*). Holotype male (CNC), by original designation [examined by DMW]. Type locality: USA, New York, Long Island, Greenpoint.

*leucophaea* Wulp, 1890: 141 (*Hypostena*). Lectotype female (NHMUK), by fixation of Wood (1985:28). Type locality: Mexico, Guerrero, Sierra de las Aguas Escondidas, 9500ft.

*peruviana* Townsend, 1927: 355 (*Pygophorinia*). Holotype female (USNM), by original designation [examined by DMW]. Type locality: Peru, [Puno], Casahuiri.

*frugale* Curran, 1934: 511 (*Lydella*). Holotype female (AMNH), by original designation [examined by DMW]. Type locality: Guyana, Bartica, Kartabo. **Syn n., comb. n.**

*rufiventris* Townsend, 1927: 350 (*Prodexodes*). Holotype male (USNM), by original designation [examined by DMW]. Type locality: Brazil, Sao Paulo, Itaquaquecetuba.

*summatis* Reinhard, 1974: 1158 (*Calolydella*). Holotype male (CNC), by original designation [examined by DMW]. Type locality: Mexico, Durango, 14 miles southwest of El Salto, 8000ft.

*triangulifera* Bigot, 1889: 268 (*Homodexia*). Holotype male (NHMUK), by monotypy [examined by DMW]. Type locality: Mexico.

*pictigaster* Bigot, 1889: 261 (*Ceromasia*). Holotype female (NHMUK), by monotypy [examined by DMW]. Type locality: Mexico (NHUMK).

*trifasciata* Walker, 1837: 350 (*Tachina*). Lectotype female (NHMUK), by fixation of Wood (1985:29) (examination of “Holotype: ♀” from South America in NHMUK is regarded as a lectotype fixation [JE O'Hara, pers. comm.]) [examined by DMW]. Type locality: South America, precise locality uncertain (Austen 1907).

*quadrivittata* Bigot, 1889: 261 (*Ceromasia*). Holotype male (NHMUK) [published as female], by monotypy [examined by DMW]. Type locality: Mexico.

*quadristriata* Wulp, 1890: 146 (*Hypostena*). Holotype male (NHMUK), by original designation [examined by DMW]. Type locality: Costa Rica, Volcán de Irazu, 6000ft.

#### Diagnosis

*Calolydella* are slender-bodied flies characterized by an abdomen longer than it is wide, with bold abdominal markings of yellow-gold pollinose stripes on a black background. According to Wood (1985), they lack distinctive chaetotactic characters and are difficult to characterize to genus except by their general appearance. Wood (1985) suggested the presence of only two pairs of presutural acrostichal setae as a diagnostic character; however, our current examination of this trait suggests that it varies among species, as does the rest of the thoracic chaetotaxy. Sternite 5 with only short hair-like setae, all more or less equal in size. Sternites 6 and 7 of female larger and more heavily sclerotized.

#### Distribution

Essentially a Neotropical genus save for one species, *Calolydella lathami*, which is present in the eastern USA and adjacent Canada.

#### Ecology

All species of *Calolydella* are parasitoids of Lepidoptera larvae in the families Crambidae, Erebidae, Geometridae, Hesperiidae, Lycaenidae, Notodontidae, Nymphalidae, Pieridae, Riodinidae, and Sphingidae.

### Calolydella adelinamoralesae

Fleming & Wood sp. n.

urn:lsid:zoobank.org:act:AE0FC424-12A9-4FDE-A8AF-53FE35C429F3

#### Materials

- **Type status:** Holotype. **Occurrence:** occurrenceDetails: http://janzen.sas.upenn.edu; catalogNumber: DHJPAR0017775; recordedBy: D.H. Janzen, W. Hallwachs & Carolina Cano; individualID: DHJPAR0017775; individualCount: 1; sex: M; lifeStage: adult; preparations: pinned; otherCatalogNumbers: 04-SRNP-780, BOLD:AAA7512, ASTAR486-07; **Taxon:** scientificName: Calolydella adelinamoralesae; phylum: Arthropoda; class: Insecta; order: Diptera; family: Tachinidae; genus: Calolydella; specificEpithet: adelinamoralesae; scientificNameAuthorship: Fleming & Wood, 2016; **Location:** continent: Central America; country: Costa Rica; countryCode: CR; stateProvince: Alajuela; county: Sector San Cristobal; locality: Area de Conservacion Guanacaste; verbatimLocality: Puente Palma; verbatimElevation: 460; verbatimLatitude: 10.9163; verbatimLongitude: -85.3787; verbatimCoordinateSystem: Decimal; **Identification:** identifiedBy: AJ Fleming; dateIdentified: 2016; **Event:** samplingProtocol: Reared from the larva of the arctiine erebid moth, Agaraea minuta; verbatimEventDate: 01-Mar-2004; **Record Level:** language: en; institutionCode: CNC; collectionCode: Insects; basisOfRecord: Pinned Specimen
- **Type status:** Paratype. **Occurrence:** occurrenceDetails: http://janzen.sas.upenn.edu; catalogNumber: DHJPAR0017079; recordedBy: D.H. Janzen, W. Hallwachs & Manuel Rios; individualID: DHJPAR0017079; individualCount: 1; sex: F; lifeStage: adult; preparations: pinned; otherCatalogNumbers: 07-SRNP-31260, BOLD:AAA7512, ASTAP517-07; **Taxon:** scientificName: Calolydella adelinamoralesae; phylum: Arthropoda; class: Insecta; order: Diptera; family: Tachinidae; genus: Calolydella; specificEpithet: adelinamoralesae; scientificNameAuthorship: Fleming & Wood, 2016; **Location:** continent: Central America; country: Costa Rica; countryCode: CR; stateProvince: Guanacaste; county: Sector Pitilla; locality: Area de Conservacion Guanacaste; verbatimLocality: Pasmompa; verbatimElevation: 440; verbatimLatitude: 11.0193; verbatimLongitude: -85.41; verbatimCoordinateSystem: Decimal; **Identification:** identifiedBy: AJ Fleming; dateIdentified: 2016; **Event:** samplingProtocol: Reared from the larva of the arctiine erebid moth, Agaraea minuta; verbatimEventDate: 14-Mar-2007; **Record Level:** language: en; institutionCode: CNC; collectionCode: Insects; basisOfRecord: Pinned Specimen
- **Type status:** Paratype. **Occurrence:** occurrenceDetails: http://janzen.sas.upenn.edu; catalogNumber: DHJPAR0035793; recordedBy: D.H. Janzen, W. Hallwachs & Keiner Aragon; individualID: DHJPAR0035793; individualCount: 1; sex: F; lifeStage: adult; preparations: pinned; otherCatalogNumbers: 09-SRNP-67604, BOLD:AAA7512, ASHYD1174-09; **Taxon:** scientificName: Calolydella adelinamoralesae; phylum: Arthropoda; class: Insecta; order: Diptera; family: Tachinidae; genus: Calolydella; specificEpithet: adelinamoralesae; scientificNameAuthorship: Fleming & Wood, 2016; **Location:** continent: Central America; country: Costa Rica; countryCode: CR; stateProvince: Alajuela; county: Sector Rincon Rain Forest; locality: Area de Conservacion Guanacaste; verbatimLocality: Palomo; verbatimElevation: 96; verbatimLatitude: 10.9619; verbatimLongitude: -85.2804; verbatimCoordinateSystem: Decimal; **Identification:** identifiedBy: AJ Fleming; dateIdentified: 2016; **Event:** samplingProtocol: Reared from the larva of the arctiine erebid moth, Agaraea minuta; verbatimEventDate: 19-Jul-2009; **Record Level:** language: en; institutionCode: CNC; collectionCode: Insects; basisOfRecord: Pinned Specimen
- **Type status:** Paratype. **Occurrence:** occurrenceDetails: http://janzen.sas.upenn.edu; catalogNumber: DHJPAR0017085; recordedBy: D.H. Janzen, W. Hallwachs & Manuel Rios; individualID: DHJPAR0017085; individualCount: 1; sex: F; lifeStage: adult; preparations: pinned; otherCatalogNumbers: 07-SRNP-31239, BOLD:AAA7512, ASTAP523-07; **Taxon:** scientificName: Calolydella adelinamoralesae; phylum: Arthropoda; class: Insecta; order: Diptera; family: Tachinidae; genus: Calolydella; specificEpithet: adelinamoralesae; scientificNameAuthorship: Fleming & Wood, 2016; **Location:** continent: Central America; country: Costa Rica; countryCode: CR; stateProvince: Guanacaste; county: Sector Pitilla; locality: Area de Conservacion Guanacaste; verbatimLocality: Pasmompa; verbatimElevation: 440; verbatimLatitude: 11.0193; verbatimLongitude: -85.41; verbatimCoordinateSystem: Decimal; **Identification:** identifiedBy: AJ Fleming; dateIdentified: 2016; **Event:** samplingProtocol: Reared from the larva of the arctiine erebid moth, Agaraea minuta; verbatimEventDate: 16-Mar-2007; **Record Level:** language: en; institutionCode: CNC; collectionCode: Insects; basisOfRecord: Pinned Specimen
- **Type status:** Paratype. **Occurrence:** occurrenceDetails: http://janzen.sas.upenn.edu; catalogNumber: DHJPAR0037401; recordedBy: D.H. Janzen, W. Hallwachs & Cirilo Umana; individualID: DHJPAR0037401; individualCount: 1; sex: F; lifeStage: adult; preparations: pinned; otherCatalogNumbers: 09-SRNP-76683, BOLD:AAA7512, ASHYC4146-10; **Taxon:** scientificName: Calolydella adelinamoralesae; phylum: Arthropoda; class: Insecta; order: Diptera; family: Tachinidae; genus: Calolydella; specificEpithet: adelinamoralesae; scientificNameAuthorship: Fleming & Wood, 2016; **Location:** continent: Central America; country: Costa Rica; countryCode: CR; stateProvince: Alajuela; county: Sector Rincon Rain Forest; locality: Area de Conservacion Guanacaste; verbatimLocality: Estacion Llanura; verbatimElevation: 135; verbatimLatitude: 10.9333; verbatimLongitude: -85.2533; verbatimCoordinateSystem: Decimal; **Identification:** identifiedBy: AJ Fleming; dateIdentified: 2016; **Event:** samplingProtocol: Reared from the larva of the arctiine erebid moth, Agaraea minuta; verbatimEventDate: 20-Dec-2009; **Record Level:** language: en; institutionCode: CNC; collectionCode: Insects; basisOfRecord: Pinned Specimen
- **Type status:** Paratype. **Occurrence:** occurrenceDetails: http://janzen.sas.upenn.edu; catalogNumber: DHJPAR0035728; recordedBy: D.H. Janzen, W. Hallwachs & Cirilo Umana; individualID: DHJPAR0035728; individualCount: 1; sex: F; lifeStage: adult; preparations: pinned; otherCatalogNumbers: 09-SRNP-44796, BOLD:AAA7512, ASHYD1109-09; **Taxon:** scientificName: Calolydella adelinamoralesae; phylum: Arthropoda; class: Insecta; order: Diptera; family: Tachinidae; genus: Calolydella; specificEpithet: adelinamoralesae; scientificNameAuthorship: Fleming & Wood, 2016; **Location:** continent: Central America; country: Costa Rica; countryCode: CR; stateProvince: Alajuela; county: Sector Rincon Rain Forest; locality: Area de Conservacion Guanacaste; verbatimLocality: Estacion Llanura; verbatimElevation: 135; verbatimLatitude: 10.9333; verbatimLongitude: -85.2533; verbatimCoordinateSystem: Decimal; **Identification:** identifiedBy: AJ Fleming; dateIdentified: 2016; **Event:** samplingProtocol: Reared from the larva of the arctiine erebid moth, Agaraea minuta; verbatimEventDate: 29-Jul-2009; **Record Level:** language: en; institutionCode: CNC; collectionCode: Insects; basisOfRecord: Pinned Specimen
- **Type status:** Paratype. **Occurrence:** occurrenceDetails: http://janzen.sas.upenn.edu; catalogNumber: DHJPAR0011693; recordedBy: D.H. Janzen, W. Hallwachs & Roster Moraga; individualID: DHJPAR0011693; individualCount: 1; sex: M; lifeStage: adult; preparations: pinned; otherCatalogNumbers: 04-SRNP-25478, BOLD:AAA7512, ASTAS419-06; **Taxon:** scientificName: Calolydella adelinamoralesae; phylum: Arthropoda; class: Insecta; order: Diptera; family: Tachinidae; genus: Calolydella; specificEpithet: adelinamoralesae; scientificNameAuthorship: Fleming & Wood, 2016; **Location:** continent: Central America; country: Costa Rica; countryCode: CR; stateProvince: Guanacaste; county: Sector Del Oro; locality: Area de Conservacion Guanacaste; verbatimLocality: Uncaria; verbatimElevation: 370; verbatimLatitude: 11.0175; verbatimLongitude: -85.4741; verbatimCoordinateSystem: Decimal; **Identification:** identifiedBy: AJ Fleming; dateIdentified: 2016; **Event:** samplingProtocol: Reared from the larva of the arctiine erebid moth, Agaraea minuta; verbatimEventDate: 25-Oct-2004; **Record Level:** language: en; institutionCode: CNC; collectionCode: Insects; basisOfRecord: Pinned Specimen
- **Type status:** Paratype. **Occurrence:** occurrenceDetails: http://janzen.sas.upenn.edu; catalogNumber: DHJPAR0030226; recordedBy: D.H. Janzen, W. Hallwachs & Duvalier Briceno; individualID: DHJPAR0030226; individualCount: 1; sex: F; lifeStage: adult; preparations: pinned; otherCatalogNumbers: 09-SRNP-65018, BOLD:AAA7512, ASHYB970-09; **Taxon:** scientificName: Calolydella adelinamoralesae; phylum: Arthropoda; class: Insecta; order: Diptera; family: Tachinidae; genus: Calolydella; specificEpithet: adelinamoralesae; scientificNameAuthorship: Fleming & Wood, 2016; **Location:** continent: Central America; country: Costa Rica; countryCode: CR; stateProvince: Alajuela; county: Brasilia; locality: Area de Conservacion Guanacaste; verbatimLocality: Moga; verbatimElevation: 320; verbatimLatitude: 11.0123; verbatimLongitude: -85.3493; verbatimCoordinateSystem: Decimal; **Identification:** identifiedBy: AJ Fleming; dateIdentified: 2016; **Event:** samplingProtocol: Reared from the larva of the arctiine erebid moth, Agaraea minuta; verbatimEventDate: 24-Jan-2009; **Record Level:** language: en; institutionCode: CNC; collectionCode: Insects; basisOfRecord: Pinned Specimen
- **Type status:** Paratype. **Occurrence:** occurrenceDetails: http://janzen.sas.upenn.edu; catalogNumber: DHJPAR0017077; recordedBy: D.H. Janzen, W. Hallwachs & Manuel Rios; individualID: DHJPAR0017077; individualCount: 1; sex: M; lifeStage: adult; preparations: pinned; otherCatalogNumbers: 07-SRNP-31268, BOLD:AAA7512, ASTAP515-07; **Taxon:** scientificName: Calolydella adelinamoralesae; phylum: Arthropoda; class: Insecta; order: Diptera; family: Tachinidae; genus: Calolydella; specificEpithet: adelinamoralesae; scientificNameAuthorship: Fleming & Wood, 2016; **Location:** continent: Central America; country: Costa Rica; countryCode: CR; stateProvince: Guanacaste; county: Sector Pitilla; locality: Area de Conservacion Guanacaste; verbatimLocality: Pasmompa; verbatimElevation: 440; verbatimLatitude: 11.0193; verbatimLongitude: -85.41; verbatimCoordinateSystem: Decimal; **Identification:** identifiedBy: AJ Fleming; dateIdentified: 2016; **Event:** samplingProtocol: Reared from the larva of the arctiine erebid moth, Agaraea minuta; verbatimEventDate: 14-Mar-2007; **Record Level:** language: en; institutionCode: CNC; collectionCode: Insects; basisOfRecord: Pinned Specimen
- **Type status:** Paratype. **Occurrence:** occurrenceDetails: http://janzen.sas.upenn.edu; catalogNumber: DHJPAR0009962; recordedBy: D.H. Janzen, W. Hallwachs & Yessenia Mendoza; individualID: DHJPAR0009962; individualCount: 1; sex: F; lifeStage: adult; preparations: pinned; otherCatalogNumbers: 06-SRNP-3195, BOLD:AAA7512, ASTAV563-06; **Taxon:** scientificName: Calolydella adelinamoralesae; phylum: Arthropoda; class: Insecta; order: Diptera; family: Tachinidae; genus: Calolydella; specificEpithet: adelinamoralesae; scientificNameAuthorship: Fleming & Wood, 2016; **Location:** continent: Central America; country: Costa Rica; countryCode: CR; stateProvince: Alajuela; county: Sector San Cristobal; locality: Area de Conservacion Guanacaste; verbatimLocality: Sendero Huerta; verbatimElevation: 527; verbatimLatitude: 10.9305; verbatimLongitude: -85.3722; verbatimCoordinateSystem: Decimal; **Identification:** identifiedBy: AJ Fleming; dateIdentified: 2016; **Event:** samplingProtocol: Reared from the larva of the arctiine erebid moth, Agaraea minuta; verbatimEventDate: 12-May-2006; **Record Level:** language: en; institutionCode: CNC; collectionCode: Insects; basisOfRecord: Pinned Specimen
- **Type status:** Paratype. **Occurrence:** occurrenceDetails: http://janzen.sas.upenn.edu; catalogNumber: DHJPAR0011699; recordedBy: D.H. Janzen, W. Hallwachs & Roster Moraga; individualID: DHJPAR0011699; individualCount: 1; sex: M; lifeStage: adult; preparations: pinned; otherCatalogNumbers: 04-SRNP-25477, BOLD:AAA7512, ASTAS425-06; **Taxon:** scientificName: Calolydella adelinamoralesae; phylum: Arthropoda; class: Insecta; order: Diptera; family: Tachinidae; genus: Calolydella; specificEpithet: adelinamoralesae; scientificNameAuthorship: Fleming & Wood, 2016; **Location:** continent: Central America; country: Costa Rica; countryCode: CR; stateProvince: Guanacaste; county: Sector Del Oro; locality: Area de Conservacion Guanacaste; verbatimLocality: Uncaria; verbatimElevation: 370; verbatimLatitude: 11.0175; verbatimLongitude: -85.4741; verbatimCoordinateSystem: Decimal; **Identification:** identifiedBy: AJ Fleming; dateIdentified: 2016; **Event:** samplingProtocol: Reared from the larva of the arctiine erebid moth, Agaraea minuta; verbatimEventDate: 26-Oct-2004; **Record Level:** language: en; institutionCode: CNC; collectionCode: Insects; basisOfRecord: Pinned Specimen
- **Type status:** Paratype. **Occurrence:** occurrenceDetails: http://janzen.sas.upenn.edu; catalogNumber: DHJPAR0038669; recordedBy: D.H. Janzen, W. Hallwachs & Keiner Aragon; individualID: DHJPAR0038669; individualCount: 1; sex: F; lifeStage: adult; preparations: pinned; otherCatalogNumbers: 10-SRNP-67234, BOLD:AAA7512, ASHYD2242-10; **Taxon:** scientificName: Calolydella adelinamoralesae; phylum: Arthropoda; class: Insecta; order: Diptera; family: Tachinidae; genus: Calolydella; specificEpithet: adelinamoralesae; scientificNameAuthorship: Fleming & Wood, 2016; **Location:** continent: Central America; country: Costa Rica; countryCode: CR; stateProvince: Alajuela; county: Sector Rincon Rain Forest; locality: Area de Conservacion Guanacaste; verbatimLocality: Palomo; verbatimElevation: 96; verbatimLatitude: 10.9619; verbatimLongitude: -85.2804; verbatimCoordinateSystem: Decimal; **Identification:** identifiedBy: AJ Fleming; dateIdentified: 2016; **Event:** samplingProtocol: Reared from the larva of the arctiine erebid moth, Agaraea minuta; verbatimEventDate: 17-Mar-2010; **Record Level:** language: en; institutionCode: CNC; collectionCode: Insects; basisOfRecord: Pinned Specimen
- **Type status:** Paratype. **Occurrence:** occurrenceDetails: http://janzen.sas.upenn.edu; catalogNumber: DHJPAR0035829; recordedBy: D.H. Janzen, W. Hallwachs & Duvalier Briceno; individualID: DHJPAR0035829; individualCount: 1; sex: F; lifeStage: adult; preparations: pinned; otherCatalogNumbers: 09-SRNP-65918, BOLD:AAA7512, ASHYD1210-09; **Taxon:** scientificName: Calolydella adelinamoralesae; phylum: Arthropoda; class: Insecta; order: Diptera; family: Tachinidae; genus: Calolydella; specificEpithet: adelinamoralesae; scientificNameAuthorship: Fleming & Wood, 2016; **Location:** continent: Central America; country: Costa Rica; countryCode: CR; stateProvince: Alajuela; county: Brasilia; locality: Area de Conservacion Guanacaste; verbatimLocality: Moga; verbatimElevation: 320; verbatimLatitude: 11.0123; verbatimLongitude: -85.3493; verbatimCoordinateSystem: Decimal; **Identification:** identifiedBy: AJ Fleming; dateIdentified: 2016; **Event:** samplingProtocol: Reared from the larva of the arctiine erebid moth, Agaraea minuta; verbatimEventDate: 14-Jul-2009; **Record Level:** language: en; institutionCode: CNC; collectionCode: Insects; basisOfRecord: Pinned Specimen
- **Type status:** Paratype. **Occurrence:** occurrenceDetails: http://janzen.sas.upenn.edu; catalogNumber: DHJPAR0017088; recordedBy: D.H. Janzen, W. Hallwachs & Manuel Rios; individualID: DHJPAR0017088; individualCount: 1; sex: F; lifeStage: adult; preparations: pinned; otherCatalogNumbers: 07-SRNP-31244, BOLD:AAA7512, ASTAP526-07; **Taxon:** scientificName: Calolydella adelinamoralesae; phylum: Arthropoda; class: Insecta; order: Diptera; family: Tachinidae; genus: Calolydella; specificEpithet: adelinamoralesae; scientificNameAuthorship: Fleming & Wood, 2016; **Location:** continent: Central America; country: Costa Rica; countryCode: CR; stateProvince: Guanacaste; county: Sector Pitilla; locality: Area de Conservacion Guanacaste; verbatimLocality: Pasmompa; verbatimElevation: 440; verbatimLatitude: 11.0193; verbatimLongitude: -85.41; verbatimCoordinateSystem: Decimal; **Identification:** identifiedBy: AJ Fleming; dateIdentified: 2016; **Event:** samplingProtocol: Reared from the larva of the arctiine erebid moth, Agaraea minuta; verbatimEventDate: 16-Mar-2007; **Record Level:** language: en; institutionCode: CNC; collectionCode: Insects; basisOfRecord: Pinned Specimen
- **Type status:** Paratype. **Occurrence:** occurrenceDetails: http://janzen.sas.upenn.edu; catalogNumber: DHJPAR0035784; recordedBy: D.H. Janzen, W. Hallwachs & Cirilo Umana; individualID: DHJPAR0035784; individualCount: 1; sex: M; lifeStage: adult; preparations: pinned; otherCatalogNumbers: 09-SRNP-67687, BOLD:AAA7512, ASHYD1165-09; **Taxon:** scientificName: Calolydella adelinamoralesae; phylum: Arthropoda; class: Insecta; order: Diptera; family: Tachinidae; genus: Calolydella; specificEpithet: adelinamoralesae; scientificNameAuthorship: Fleming & Wood, 2016; **Location:** continent: Central America; country: Costa Rica; countryCode: CR; stateProvince: Alajuela; county: Sector Rincon Rain Forest; locality: Area de Conservacion Guanacaste; verbatimLocality: Palomo; verbatimElevation: 96; verbatimLatitude: 10.9619; verbatimLongitude: -85.2804; verbatimCoordinateSystem: Decimal; **Identification:** identifiedBy: AJ Fleming; dateIdentified: 2016; **Event:** samplingProtocol: Reared from the larva of the arctiine erebid moth, Agaraea minuta; verbatimEventDate: 19-Jul-2009; **Record Level:** language: en; institutionCode: CNC; collectionCode: Insects; basisOfRecord: Pinned Specimen
- **Type status:** Paratype. **Occurrence:** occurrenceDetails: http://janzen.sas.upenn.edu; catalogNumber: DHJPAR0030217; recordedBy: D.H. Janzen, W. Hallwachs & Duvalier Briceno; individualID: DHJPAR0030217; individualCount: 1; sex: F; lifeStage: adult; preparations: pinned; otherCatalogNumbers: 09-SRNP-65019, BOLD:AAA7512, ASHYB961-09; **Taxon:** scientificName: Calolydella adelinamoralesae; phylum: Arthropoda; class: Insecta; order: Diptera; family: Tachinidae; genus: Calolydella; specificEpithet: adelinamoralesae; scientificNameAuthorship: Fleming & Wood, 2016; **Location:** continent: Central America; country: Costa Rica; countryCode: CR; stateProvince: Alajuela; county: Brasilia; locality: Area de Conservacion Guanacaste; verbatimLocality: Moga; verbatimElevation: 320; verbatimLatitude: 11.0123; verbatimLongitude: -85.3493; verbatimCoordinateSystem: Decimal; **Identification:** identifiedBy: AJ Fleming; dateIdentified: 2016; **Event:** samplingProtocol: Reared from the larva of the arctiine erebid moth, Agaraea minuta; verbatimEventDate: 28-Jan-2009; **Record Level:** language: en; institutionCode: CNC; collectionCode: Insects; basisOfRecord: Pinned Specimen
- **Type status:** Paratype. **Occurrence:** occurrenceDetails: http://janzen.sas.upenn.edu; catalogNumber: DHJPAR0037372; recordedBy: D.H. Janzen, W. Hallwachs & Cirilo Umana; individualID: DHJPAR0037372; individualCount: 1; sex: F; lifeStage: adult; preparations: pinned; otherCatalogNumbers: 09-SRNP-76670, BOLD:AAA7512, ASHYC4117-10; **Taxon:** scientificName: Calolydella adelinamoralesae; phylum: Arthropoda; class: Insecta; order: Diptera; family: Tachinidae; genus: Calolydella; specificEpithet: adelinamoralesae; scientificNameAuthorship: Fleming & Wood, 2016; **Location:** continent: Central America; country: Costa Rica; countryCode: CR; stateProvince: Alajuela; county: Sector Rincon Rain Forest; locality: Area de Conservacion Guanacaste; verbatimLocality: Estacion Llanura; verbatimElevation: 135; verbatimLatitude: 10.9333; verbatimLongitude: -85.2533; verbatimCoordinateSystem: Decimal; **Identification:** identifiedBy: AJ Fleming; dateIdentified: 2016; **Event:** samplingProtocol: Reared from the larva of the arctiine erebid moth, Agaraea minuta; verbatimEventDate: 19-Dec-2009; **Record Level:** language: en; institutionCode: CNC; collectionCode: Insects; basisOfRecord: Pinned Specimen
- **Type status:** Paratype. **Occurrence:** occurrenceDetails: http://janzen.sas.upenn.edu; catalogNumber: DHJPAR0035795; recordedBy: D.H. Janzen, W. Hallwachs & Cirilo Umana; individualID: DHJPAR0035795; individualCount: 1; sex: M; lifeStage: adult; preparations: pinned; otherCatalogNumbers: 09-SRNP-67644, BOLD:AAA7512, ASHYD1176-09; **Taxon:** scientificName: Calolydella adelinamoralesae; phylum: Arthropoda; class: Insecta; order: Diptera; family: Tachinidae; genus: Calolydella; specificEpithet: adelinamoralesae; scientificNameAuthorship: Fleming & Wood, 2016; **Location:** continent: Central America; country: Costa Rica; countryCode: CR; stateProvince: Alajuela; county: Sector Rincon Rain Forest; locality: Area de Conservacion Guanacaste; verbatimLocality: Palomo; verbatimElevation: 96; verbatimLatitude: 10.9619; verbatimLongitude: -85.2804; verbatimCoordinateSystem: Decimal; **Identification:** identifiedBy: AJ Fleming; dateIdentified: 2016; **Event:** samplingProtocol: Reared from the larva of the arctiine erebid moth, Agaraea minuta; verbatimEventDate: 24-Jul-2009; **Record Level:** language: en; institutionCode: CNC; collectionCode: Insects; basisOfRecord: Pinned Specimen
- **Type status:** Paratype. **Occurrence:** occurrenceDetails: http://janzen.sas.upenn.edu; catalogNumber: DHJPAR0017083; recordedBy: D.H. Janzen, W. Hallwachs & Manuel Rios; individualID: DHJPAR0017083; individualCount: 1; sex: F; lifeStage: adult; preparations: pinned; otherCatalogNumbers: 07-SRNP-31229, BOLD:AAA7512, ASTAP521-07; **Taxon:** scientificName: Calolydella adelinamoralesae; phylum: Arthropoda; class: Insecta; order: Diptera; family: Tachinidae; genus: Calolydella; specificEpithet: adelinamoralesae; scientificNameAuthorship: Fleming & Wood, 2016; **Location:** continent: Central America; country: Costa Rica; countryCode: CR; stateProvince: Guanacaste; county: Sector Pitilla; locality: Area de Conservacion Guanacaste; verbatimLocality: Pasmompa; verbatimElevation: 440; verbatimLatitude: 11.0193; verbatimLongitude: -85.41; verbatimCoordinateSystem: Decimal; **Identification:** identifiedBy: AJ Fleming; dateIdentified: 2016; **Event:** samplingProtocol: Reared from the larva of the arctiine erebid moth, Agaraea minuta; verbatimEventDate: 14-Mar-2007; **Record Level:** language: en; institutionCode: CNC; collectionCode: Insects; basisOfRecord: Pinned Specimen
- **Type status:** Paratype. **Occurrence:** occurrenceDetails: http://janzen.sas.upenn.edu; catalogNumber: DHJPAR0017081; recordedBy: D.H. Janzen, W. Hallwachs & Manuel Rios; individualID: DHJPAR0017081; individualCount: 1; sex: F; lifeStage: adult; preparations: pinned; otherCatalogNumbers: 07-SRNP-31234, BOLD:AAA7512, ASTAP519-07; **Taxon:** scientificName: Calolydella adelinamoralesae; phylum: Arthropoda; class: Insecta; order: Diptera; family: Tachinidae; genus: Calolydella; specificEpithet: adelinamoralesae; scientificNameAuthorship: Fleming & Wood, 2016; **Location:** continent: Central America; country: Costa Rica; countryCode: CR; stateProvince: Guanacaste; county: Sector Pitilla; locality: Area de Conservacion Guanacaste; verbatimLocality: Pasmompa; verbatimElevation: 440; verbatimLatitude: 11.0193; verbatimLongitude: -85.41; verbatimCoordinateSystem: Decimal; **Identification:** identifiedBy: AJ Fleming; dateIdentified: 2016; **Event:** samplingProtocol: Reared from the larva of the arctiine erebid moth, Agaraea minuta; verbatimEventDate: 15-Mar-2007; **Record Level:** language: en; institutionCode: CNC; collectionCode: Insects; basisOfRecord: Pinned Specimen
- **Type status:** Paratype. **Occurrence:** occurrenceDetails: http://janzen.sas.upenn.edu; catalogNumber: DHJPAR0017086; recordedBy: D.H. Janzen, W. Hallwachs & Manuel Rios; individualID: DHJPAR0017086; individualCount: 1; sex: F; lifeStage: adult; preparations: pinned; otherCatalogNumbers: 07-SRNP-31272, BOLD:AAA7512, ASTAP524-07; **Taxon:** scientificName: Calolydella adelinamoralesae; phylum: Arthropoda; class: Insecta; order: Diptera; family: Tachinidae; genus: Calolydella; specificEpithet: adelinamoralesae; scientificNameAuthorship: Fleming & Wood, 2016; **Location:** continent: Central America; country: Costa Rica; countryCode: CR; stateProvince: Guanacaste; county: Sector Pitilla; locality: Area de Conservacion Guanacaste; verbatimLocality: Pasmompa; verbatimElevation: 440; verbatimLatitude: 11.0193; verbatimLongitude: -85.41; verbatimCoordinateSystem: Decimal; **Identification:** identifiedBy: AJ Fleming; dateIdentified: 2016; **Event:** samplingProtocol: Reared from the larva of the arctiine erebid moth, Agaraea minuta; verbatimEventDate: 16-Mar-2007; **Record Level:** language: en; institutionCode: CNC; collectionCode: Insects; basisOfRecord: Pinned Specimen
- **Type status:** Paratype. **Occurrence:** occurrenceDetails: http://janzen.sas.upenn.edu; catalogNumber: DHJPAR0034521; recordedBy: D.H. Janzen, W. Hallwachs & Duvalier Briceno; individualID: DHJPAR0034521; individualCount: 1; sex: M; lifeStage: adult; preparations: pinned; otherCatalogNumbers: 09-SRNP-67040, BOLD:AAA7512, ASHYC1173-09; **Taxon:** scientificName: Calolydella adelinamoralesae; phylum: Arthropoda; class: Insecta; order: Diptera; family: Tachinidae; genus: Calolydella; specificEpithet: adelinamoralesae; scientificNameAuthorship: Fleming & Wood, 2016; **Location:** continent: Central America; country: Costa Rica; countryCode: CR; stateProvince: Alajuela; county: Sector Rincon Rain Forest; locality: Area de Conservacion Guanacaste; verbatimLocality: Palomo; verbatimElevation: 96; verbatimLatitude: 10.9619; verbatimLongitude: -85.2804; verbatimCoordinateSystem: Decimal; **Identification:** identifiedBy: AJ Fleming; dateIdentified: 2016; **Event:** samplingProtocol: Reared from the larva of the arctiine erebid moth, Agaraea minuta; verbatimEventDate: 01-Jun-2009; **Record Level:** language: en; institutionCode: CNC; collectionCode: Insects; basisOfRecord: Pinned Specimen
- **Type status:** Paratype. **Occurrence:** occurrenceDetails: http://janzen.sas.upenn.edu; catalogNumber: DHJPAR0017076; recordedBy: D.H. Janzen, W. Hallwachs & Manuel Rios; individualID: DHJPAR0017076; individualCount: 1; sex: M; lifeStage: adult; preparations: pinned; otherCatalogNumbers: 07-SRNP-31277, BOLD:AAA7512, ASTAP514-07; **Taxon:** scientificName: Calolydella adelinamoralesae; phylum: Arthropoda; class: Insecta; order: Diptera; family: Tachinidae; genus: Calolydella; specificEpithet: adelinamoralesae; scientificNameAuthorship: Fleming & Wood, 2016; **Location:** continent: Central America; country: Costa Rica; countryCode: CR; stateProvince: Guanacaste; county: Sector Pitilla; locality: Area de Conservacion Guanacaste; verbatimLocality: Pasmompa; verbatimElevation: 440; verbatimLatitude: 11.0193; verbatimLongitude: -85.41; verbatimCoordinateSystem: Decimal; **Identification:** identifiedBy: AJ Fleming; dateIdentified: 2016; **Event:** samplingProtocol: Reared from the larva of the arctiine erebid moth, Agaraea minuta; verbatimEventDate: 14-Mar-2007; **Record Level:** language: en; institutionCode: CNC; collectionCode: Insects; basisOfRecord: Pinned Specimen
- **Type status:** Paratype. **Occurrence:** occurrenceDetails: http://janzen.sas.upenn.edu; catalogNumber: DHJPAR0017075; recordedBy: D.H. Janzen, W. Hallwachs & Manuel Rios; individualID: DHJPAR0017075; individualCount: 1; sex: M; lifeStage: adult; preparations: pinned; otherCatalogNumbers: 07-SRNP-31263, BOLD:AAA7512, ASTAP513-07; **Taxon:** scientificName: Calolydella adelinamoralesae; phylum: Arthropoda; class: Insecta; order: Diptera; family: Tachinidae; genus: Calolydella; specificEpithet: adelinamoralesae; scientificNameAuthorship: Fleming & Wood, 2016; **Location:** continent: Central America; country: Costa Rica; countryCode: CR; stateProvince: Guanacaste; county: Sector Pitilla; locality: Area de Conservacion Guanacaste; verbatimLocality: Pasmompa; verbatimElevation: 440; verbatimLatitude: 11.0193; verbatimLongitude: -85.41; verbatimCoordinateSystem: Decimal; **Identification:** identifiedBy: AJ Fleming; dateIdentified: 2016; **Event:** samplingProtocol: Reared from the larva of the arctiine erebid moth, Agaraea minuta; verbatimEventDate: 15-Mar-2007; **Record Level:** language: en; institutionCode: CNC; collectionCode: Insects; basisOfRecord: Pinned Specimen
- **Type status:** Paratype. **Occurrence:** occurrenceDetails: http://janzen.sas.upenn.edu; catalogNumber: DHJPAR0017087; recordedBy: D.H. Janzen, W. Hallwachs & Manuel Rios; individualID: DHJPAR0017087; individualCount: 1; sex: F; lifeStage: adult; preparations: pinned; otherCatalogNumbers: 07-SRNP-31231, BOLD:AAA7512, ASTAP525-07; **Taxon:** scientificName: Calolydella adelinamoralesae; phylum: Arthropoda; class: Insecta; order: Diptera; family: Tachinidae; genus: Calolydella; specificEpithet: adelinamoralesae; scientificNameAuthorship: Fleming & Wood, 2016; **Location:** continent: Central America; country: Costa Rica; countryCode: CR; stateProvince: Guanacaste; county: Sector Pitilla; locality: Area de Conservacion Guanacaste; verbatimLocality: Pasmompa; verbatimElevation: 440; verbatimLatitude: 11.0193; verbatimLongitude: -85.41; verbatimCoordinateSystem: Decimal; **Identification:** identifiedBy: AJ Fleming; dateIdentified: 2016; **Event:** samplingProtocol: Reared from the larva of the arctiine erebid moth, Agaraea minuta; verbatimEventDate: 16-Mar-2007; **Record Level:** language: en; institutionCode: CNC; collectionCode: Insects; basisOfRecord: Pinned Specimen
- **Type status:** Paratype. **Occurrence:** occurrenceDetails: http://janzen.sas.upenn.edu; catalogNumber: DHJPAR0035781; recordedBy: D.H. Janzen, W. Hallwachs & Cirilo Umana; individualID: DHJPAR0035781; individualCount: 1; sex: F; lifeStage: adult; preparations: pinned; otherCatalogNumbers: 09-SRNP-67672, BOLD:AAA7512, ASHYD1162-09; **Taxon:** scientificName: Calolydella adelinamoralesae; phylum: Arthropoda; class: Insecta; order: Diptera; family: Tachinidae; genus: Calolydella; specificEpithet: adelinamoralesae; scientificNameAuthorship: Fleming & Wood, 2016; **Location:** continent: Central America; country: Costa Rica; countryCode: CR; stateProvince: Alajuela; county: Sector Rincon Rain Forest; locality: Area de Conservacion Guanacaste; verbatimLocality: Palomo; verbatimElevation: 96; verbatimLatitude: 10.9619; verbatimLongitude: -85.2804; verbatimCoordinateSystem: Decimal; **Identification:** identifiedBy: AJ Fleming; dateIdentified: 2016; **Event:** samplingProtocol: Reared from the larva of the arctiine erebid moth, Agaraea minuta; verbatimEventDate: 25-Jul-2009; **Record Level:** language: en; institutionCode: CNC; collectionCode: Insects; basisOfRecord: Pinned Specimen
- **Type status:** Paratype. **Occurrence:** occurrenceDetails: http://janzen.sas.upenn.edu; catalogNumber: DHJPAR0035798; recordedBy: D.H. Janzen, W. Hallwachs & Keiner Aragon; individualID: DHJPAR0035798; individualCount: 1; sex: F; lifeStage: adult; preparations: pinned; otherCatalogNumbers: 09-SRNP-67599, BOLD:AAA7512, ASHYD1179-09; **Taxon:** scientificName: Calolydella adelinamoralesae; phylum: Arthropoda; class: Insecta; order: Diptera; family: Tachinidae; genus: Calolydella; specificEpithet: adelinamoralesae; scientificNameAuthorship: Fleming & Wood, 2016; **Location:** continent: Central America; country: Costa Rica; countryCode: CR; stateProvince: Alajuela; county: Sector Rincon Rain Forest; locality: Area de Conservacion Guanacaste; verbatimLocality: Palomo; verbatimElevation: 96; verbatimLatitude: 10.9619; verbatimLongitude: -85.2804; verbatimCoordinateSystem: Decimal; **Identification:** identifiedBy: AJ Fleming; dateIdentified: 2016; **Event:** samplingProtocol: Reared from the larva of the arctiine erebid moth, Agaraea minuta; verbatimEventDate: 25-Jul-2009; **Record Level:** language: en; institutionCode: CNC; collectionCode: Insects; basisOfRecord: Pinned Specimen
- **Type status:** Paratype. **Occurrence:** occurrenceDetails: http://janzen.sas.upenn.edu; catalogNumber: DHJPAR0010349; recordedBy: D.H. Janzen, W. Hallwachs & Minor Carmona; individualID: DHJPAR0010349; individualCount: 1; sex: F; lifeStage: adult; preparations: pinned; otherCatalogNumbers: 06-SRNP-42838, BOLD:AAA7512, ASTAS180-06; **Taxon:** scientificName: Calolydella adelinamoralesae; phylum: Arthropoda; class: Insecta; order: Diptera; family: Tachinidae; genus: Calolydella; specificEpithet: adelinamoralesae; scientificNameAuthorship: Fleming & Wood, 2016; **Location:** continent: Central America; country: Costa Rica; countryCode: CR; stateProvince: Alajuela; county: Sector Rincon Rain Forest; locality: Area de Conservacion Guanacaste; verbatimLocality: Cabanya; verbatimElevation: 340; verbatimLatitude: 10.877; verbatimLongitude: -85.2308; verbatimCoordinateSystem: Decimal; **Identification:** identifiedBy: AJ Fleming; dateIdentified: 2016; **Event:** samplingProtocol: Reared from the larva of the arctiine erebid moth, Agaraea minuta; verbatimEventDate: 28-Aug-2006; **Record Level:** language: en; institutionCode: CNC; collectionCode: Insects; basisOfRecord: Pinned Specimen
- **Type status:** Paratype. **Occurrence:** occurrenceDetails: http://janzen.sas.upenn.edu; catalogNumber: DHJPAR0035732; recordedBy: D.H. Janzen, W. Hallwachs & Cirilo Umana; individualID: DHJPAR0035732; individualCount: 1; sex: M; lifeStage: adult; preparations: pinned; otherCatalogNumbers: 09-SRNP-44788, BOLD:AAA7512, ASHYD1113-09; **Taxon:** scientificName: Calolydella adelinamoralesae; phylum: Arthropoda; class: Insecta; order: Diptera; family: Tachinidae; genus: Calolydella; specificEpithet: adelinamoralesae; scientificNameAuthorship: Fleming & Wood, 2016; **Location:** continent: Central America; country: Costa Rica; countryCode: CR; stateProvince: Alajuela; county: Sector Rincon Rain Forest; locality: Area de Conservacion Guanacaste; verbatimLocality: Estacion Llanura; verbatimElevation: 135; verbatimLatitude: 10.9333; verbatimLongitude: -85.2533; verbatimCoordinateSystem: Decimal; **Identification:** identifiedBy: AJ Fleming; dateIdentified: 2016; **Event:** samplingProtocol: Reared from the larva of the arctiine erebid moth, Agaraea minuta; verbatimEventDate: 27-Jul-2009; **Record Level:** language: en; institutionCode: CNC; collectionCode: Insects; basisOfRecord: Pinned Specimen
- **Type status:** Paratype. **Occurrence:** occurrenceDetails: http://janzen.sas.upenn.edu; catalogNumber: DHJPAR0035707; recordedBy: D.H. Janzen, W. Hallwachs & Cirilo Umana; individualID: DHJPAR0035707; individualCount: 1; sex: M; lifeStage: adult; preparations: pinned; otherCatalogNumbers: 09-SRNP-44825, BOLD:AAA7512, ASHYD1088-09; **Taxon:** scientificName: Calolydella adelinamoralesae; phylum: Arthropoda; class: Insecta; order: Diptera; family: Tachinidae; genus: Calolydella; specificEpithet: adelinamoralesae; scientificNameAuthorship: Fleming & Wood, 2016; **Location:** continent: Central America; country: Costa Rica; countryCode: CR; stateProvince: Alajuela; county: Sector Rincon Rain Forest; locality: Area de Conservacion Guanacaste; verbatimLocality: Estacion Llanura; verbatimElevation: 135; verbatimLatitude: 10.9333; verbatimLongitude: -85.2533; verbatimCoordinateSystem: Decimal; **Identification:** identifiedBy: AJ Fleming; dateIdentified: 2016; **Event:** samplingProtocol: Reared from the larva of the arctiine erebid moth, Agaraea minuta; verbatimEventDate: 21-Jul-2009; **Record Level:** language: en; institutionCode: CNC; collectionCode: Insects; basisOfRecord: Pinned Specimen
- **Type status:** Paratype. **Occurrence:** occurrenceDetails: http://janzen.sas.upenn.edu; catalogNumber: DHJPAR0030227; recordedBy: D.H. Janzen, W. Hallwachs & Duvalier Briceno; individualID: DHJPAR0030227; individualCount: 1; sex: M; lifeStage: adult; preparations: pinned; otherCatalogNumbers: 09-SRNP-65020, BOLD:AAA7512, ASHYB971-09; **Taxon:** scientificName: Calolydella adelinamoralesae; phylum: Arthropoda; class: Insecta; order: Diptera; family: Tachinidae; genus: Calolydella; specificEpithet: adelinamoralesae; scientificNameAuthorship: Fleming & Wood, 2016; **Location:** continent: Central America; country: Costa Rica; countryCode: CR; stateProvince: Alajuela; county: Brasilia; locality: Area de Conservacion Guanacaste; verbatimLocality: Moga; verbatimElevation: 320; verbatimLatitude: 11.0123; verbatimLongitude: -85.3493; verbatimCoordinateSystem: Decimal; **Identification:** identifiedBy: AJ Fleming; dateIdentified: 2016; **Event:** samplingProtocol: Reared from the larva of the arctiine erebid moth, Agaraea minuta; verbatimEventDate: 28-Jan-2009; **Record Level:** language: en; institutionCode: CNC; collectionCode: Insects; basisOfRecord: Pinned Specimen
- **Type status:** Paratype. **Occurrence:** occurrenceDetails: http://janzen.sas.upenn.edu; catalogNumber: DHJPAR0011700; recordedBy: D.H. Janzen, W. Hallwachs & Manuel Rios; individualID: DHJPAR0011700; individualCount: 1; sex: M; lifeStage: adult; preparations: pinned; otherCatalogNumbers: 05-SRNP-30308, BOLD:AAA7512, ASTAS426-06; **Taxon:** scientificName: Calolydella adelinamoralesae; phylum: Arthropoda; class: Insecta; order: Diptera; family: Tachinidae; genus: Calolydella; specificEpithet: adelinamoralesae; scientificNameAuthorship: Fleming & Wood, 2016; **Location:** continent: Central America; country: Costa Rica; countryCode: CR; stateProvince: Guanacaste; county: Sector Pitilla; locality: Area de Conservacion Guanacaste; verbatimLocality: Pasmompa; verbatimElevation: 440; verbatimLatitude: 11.0193; verbatimLongitude: -85.41; verbatimCoordinateSystem: Decimal; **Identification:** identifiedBy: AJ Fleming; dateIdentified: 2016; **Event:** samplingProtocol: Reared from the larva of the arctiine erebid moth, Agaraea minuta; **Record Level:** language: en; institutionCode: CNC; collectionCode: Insects; basisOfRecord: Pinned Specimen
- **Type status:** Paratype. **Occurrence:** occurrenceDetails: http://janzen.sas.upenn.edu; catalogNumber: DHJPAR0035705; recordedBy: D.H. Janzen, W. Hallwachs & Cirilo Umana; individualID: DHJPAR0035705; individualCount: 1; sex: F; lifeStage: adult; preparations: pinned; otherCatalogNumbers: 09-SRNP-44799, BOLD:AAA7512, ASHYD1086-09; **Taxon:** scientificName: Calolydella adelinamoralesae; phylum: Arthropoda; class: Insecta; order: Diptera; family: Tachinidae; genus: Calolydella; specificEpithet: adelinamoralesae; scientificNameAuthorship: Fleming & Wood, 2016; **Location:** continent: Central America; country: Costa Rica; countryCode: CR; stateProvince: Alajuela; county: Sector Rincon Rain Forest; locality: Area de Conservacion Guanacaste; verbatimLocality: Estacion Llanura; verbatimElevation: 135; verbatimLatitude: 10.9333; verbatimLongitude: -85.2533; verbatimCoordinateSystem: Decimal; **Identification:** identifiedBy: AJ Fleming; dateIdentified: 2016; **Event:** samplingProtocol: Reared from the larva of the arctiine erebid moth, Agaraea minuta; verbatimEventDate: 22-Jul-2009; **Record Level:** language: en; institutionCode: CNC; collectionCode: Insects; basisOfRecord: Pinned Specimen
- **Type status:** Paratype. **Occurrence:** occurrenceDetails: http://janzen.sas.upenn.edu; catalogNumber: DHJPAR0011694; recordedBy: D.H. Janzen, W. Hallwachs & Roster Moraga; individualID: DHJPAR0011694; individualCount: 1; sex: M; lifeStage: adult; preparations: pinned; otherCatalogNumbers: 04-SRNP-25355, BOLD:AAA7512, ASTAS420-06; **Taxon:** scientificName: Calolydella adelinamoralesae; phylum: Arthropoda; class: Insecta; order: Diptera; family: Tachinidae; genus: Calolydella; specificEpithet: adelinamoralesae; scientificNameAuthorship: Fleming & Wood, 2016; **Location:** continent: Central America; country: Costa Rica; countryCode: CR; stateProvince: Guanacaste; county: Sector Del Oro; locality: Area de Conservacion Guanacaste; verbatimLocality: Uncaria; verbatimElevation: 370; verbatimLatitude: 11.0175; verbatimLongitude: -85.4741; verbatimCoordinateSystem: Decimal; **Identification:** identifiedBy: AJ Fleming; dateIdentified: 2016; **Event:** samplingProtocol: Reared from the larva of the arctiine erebid moth, Agaraea minuta; verbatimEventDate: 26-Oct-2004; **Record Level:** language: en; institutionCode: CNC; collectionCode: Insects; basisOfRecord: Pinned Specimen
- **Type status:** Paratype. **Occurrence:** occurrenceDetails: http://janzen.sas.upenn.edu; catalogNumber: DHJPAR0035799; recordedBy: D.H. Janzen, W. Hallwachs & Cirilo Umana; individualID: DHJPAR0035799; individualCount: 1; sex: F; lifeStage: adult; preparations: pinned; otherCatalogNumbers: 09-SRNP-67660, BOLD:AAA7512, ASHYD1180-09; **Taxon:** scientificName: Calolydella adelinamoralesae; phylum: Arthropoda; class: Insecta; order: Diptera; family: Tachinidae; genus: Calolydella; specificEpithet: adelinamoralesae; scientificNameAuthorship: Fleming & Wood, 2016; **Location:** continent: Central America; country: Costa Rica; countryCode: CR; stateProvince: Alajuela; county: Sector Rincon Rain Forest; locality: Area de Conservacion Guanacaste; verbatimLocality: Palomo; verbatimElevation: 96; verbatimLatitude: 10.9619; verbatimLongitude: -85.2804; verbatimCoordinateSystem: Decimal; **Identification:** identifiedBy: AJ Fleming; dateIdentified: 2016; **Event:** samplingProtocol: Reared from the larva of the arctiine erebid moth, Agaraea minuta; verbatimEventDate: 25-Jul-2009; **Record Level:** language: en; institutionCode: CNC; collectionCode: Insects; basisOfRecord: Pinned Specimen
- **Type status:** Paratype. **Occurrence:** occurrenceDetails: http://janzen.sas.upenn.edu; catalogNumber: DHJPAR0038666; recordedBy: D.H. Janzen, W. Hallwachs & Keiner Aragon; individualID: DHJPAR0038666; individualCount: 1; sex: F; lifeStage: adult; preparations: pinned; otherCatalogNumbers: 10-SRNP-67215, BOLD:AAA7512, ASHYD2239-10; **Taxon:** scientificName: Calolydella adelinamoralesae; phylum: Arthropoda; class: Insecta; order: Diptera; family: Tachinidae; genus: Calolydella; specificEpithet: adelinamoralesae; scientificNameAuthorship: Fleming & Wood, 2016; **Location:** continent: Central America; country: Costa Rica; countryCode: CR; stateProvince: Alajuela; county: Sector Rincon Rain Forest; locality: Area de Conservacion Guanacaste; verbatimLocality: Palomo; verbatimElevation: 96; verbatimLatitude: 10.9619; verbatimLongitude: -85.2804; verbatimCoordinateSystem: Decimal; **Identification:** identifiedBy: AJ Fleming; dateIdentified: 2016; **Event:** samplingProtocol: Reared from the larva of the arctiine erebid moth, Agaraea minuta; verbatimEventDate: 14-Mar-2010; **Record Level:** language: en; institutionCode: CNC; collectionCode: Insects; basisOfRecord: Pinned Specimen
- **Type status:** Paratype. **Occurrence:** occurrenceDetails: http://janzen.sas.upenn.edu; catalogNumber: DHJPAR0035796; recordedBy: D.H. Janzen, W. Hallwachs & Keiner Aragon; individualID: DHJPAR0035796; individualCount: 1; sex: M; lifeStage: adult; preparations: pinned; otherCatalogNumbers: 09-SRNP-67618, BOLD:AAA7512, ASHYD1177-09; **Taxon:** scientificName: Calolydella adelinamoralesae; phylum: Arthropoda; class: Insecta; order: Diptera; family: Tachinidae; genus: Calolydella; specificEpithet: adelinamoralesae; scientificNameAuthorship: Fleming & Wood, 2016; **Location:** continent: Central America; country: Costa Rica; countryCode: CR; stateProvince: Alajuela; county: Sector Rincon Rain Forest; locality: Area de Conservacion Guanacaste; verbatimLocality: Palomo; verbatimElevation: 96; verbatimLatitude: 10.9619; verbatimLongitude: -85.2804; verbatimCoordinateSystem: Decimal; **Identification:** identifiedBy: AJ Fleming; dateIdentified: 2016; **Event:** samplingProtocol: Reared from the larva of the arctiine erebid moth, Agaraea minuta; verbatimEventDate: 24-Jul-2009; **Record Level:** language: en; institutionCode: CNC; collectionCode: Insects; basisOfRecord: Pinned Specimen
- **Type status:** Paratype. **Occurrence:** occurrenceDetails: http://janzen.sas.upenn.edu; catalogNumber: DHJPAR0019750; recordedBy: D.H. Janzen, W. Hallwachs & Manuel Rios; individualID: DHJPAR0019750; individualCount: 1; sex: M; lifeStage: adult; preparations: pinned; otherCatalogNumbers: 07-SRNP-31242, , ASTAB298-07; **Taxon:** scientificName: Calolydella adelinamoralesae; phylum: Arthropoda; class: Insecta; order: Diptera; family: Tachinidae; genus: Calolydella; specificEpithet: adelinamoralesae; scientificNameAuthorship: Fleming & Wood, 2016; **Location:** continent: Central America; country: Costa Rica; countryCode: CR; stateProvince: Guanacaste; county: Sector Pitilla; locality: Area de Conservacion Guanacaste; verbatimLocality: Pasmompa; verbatimElevation: 440; verbatimLatitude: 11.0193; verbatimLongitude: -85.41; verbatimCoordinateSystem: Decimal; **Identification:** identifiedBy: AJ Fleming; dateIdentified: 2016; **Event:** samplingProtocol: Reared from the larva of the arctiine erebid moth, Agaraea minuta; verbatimEventDate: 30-Mar-2007; **Record Level:** language: en; institutionCode: CNC; collectionCode: Insects; basisOfRecord: Pinned Specimen
- **Type status:** Paratype. **Occurrence:** occurrenceDetails: http://janzen.sas.upenn.edu; catalogNumber: DHJPAR0035792; recordedBy: D.H. Janzen, W. Hallwachs & Cirilo Umana; individualID: DHJPAR0035792; individualCount: 1; sex: M; lifeStage: adult; preparations: pinned; otherCatalogNumbers: 09-SRNP-67630, BOLD:AAA7512, ASHYD1173-09; **Taxon:** scientificName: Calolydella adelinamoralesae; phylum: Arthropoda; class: Insecta; order: Diptera; family: Tachinidae; genus: Calolydella; specificEpithet: adelinamoralesae; scientificNameAuthorship: Fleming & Wood, 2016; **Location:** continent: Central America; country: Costa Rica; countryCode: CR; stateProvince: Alajuela; county: Sector Rincon Rain Forest; locality: Area de Conservacion Guanacaste; verbatimLocality: Palomo; verbatimElevation: 96; verbatimLatitude: 10.9619; verbatimLongitude: -85.2804; verbatimCoordinateSystem: Decimal; **Identification:** identifiedBy: AJ Fleming; dateIdentified: 2016; **Event:** samplingProtocol: Reared from the larva of the arctiine erebid moth, Agaraea minuta; verbatimEventDate: 19-Jul-2009; **Record Level:** language: en; institutionCode: CNC; collectionCode: Insects; basisOfRecord: Pinned Specimen
- **Type status:** Paratype. **Occurrence:** occurrenceDetails: http://janzen.sas.upenn.edu; catalogNumber: DHJPAR0019763; recordedBy: D.H. Janzen, W. Hallwachs & Manuel Rios; individualID: DHJPAR0019763; individualCount: 1; sex: F; lifeStage: adult; preparations: pinned; otherCatalogNumbers: 07-SRNP-31258, BOLD:AAA7512, ASTAB311-07; **Taxon:** scientificName: Calolydella adelinamoralesae; phylum: Arthropoda; class: Insecta; order: Diptera; family: Tachinidae; genus: Calolydella; specificEpithet: adelinamoralesae; scientificNameAuthorship: Fleming & Wood, 2016; **Location:** continent: Central America; country: Costa Rica; countryCode: CR; stateProvince: Guanacaste; county: Sector Pitilla; locality: Area de Conservacion Guanacaste; verbatimLocality: Pasmompa; verbatimElevation: 440; verbatimLatitude: 11.0193; verbatimLongitude: -85.41; verbatimCoordinateSystem: Decimal; **Identification:** identifiedBy: AJ Fleming; dateIdentified: 2016; **Event:** samplingProtocol: Reared from the larva of the arctiine erebid moth, Agaraea minuta; verbatimEventDate: 24-Mar-2007; **Record Level:** language: en; institutionCode: CNC; collectionCode: Insects; basisOfRecord: Pinned Specimen
- **Type status:** Paratype. **Occurrence:** occurrenceDetails: http://janzen.sas.upenn.edu; catalogNumber: DHJPAR0017082; recordedBy: D.H. Janzen, W. Hallwachs & Manuel Rios; individualID: DHJPAR0017082; individualCount: 1; sex: F; lifeStage: adult; preparations: pinned; otherCatalogNumbers: 07-SRNP-31269, BOLD:AAA7512, ASTAP520-07; **Taxon:** scientificName: Calolydella adelinamoralesae; phylum: Arthropoda; class: Insecta; order: Diptera; family: Tachinidae; genus: Calolydella; specificEpithet: adelinamoralesae; scientificNameAuthorship: Fleming & Wood, 2016; **Location:** continent: Central America; country: Costa Rica; countryCode: CR; stateProvince: Guanacaste; county: Sector Pitilla; locality: Area de Conservacion Guanacaste; verbatimLocality: Pasmompa; verbatimElevation: 440; verbatimLatitude: 11.0193; verbatimLongitude: -85.41; verbatimCoordinateSystem: Decimal; **Identification:** identifiedBy: AJ Fleming; dateIdentified: 2016; **Event:** samplingProtocol: Reared from the larva of the arctiine erebid moth, Agaraea minuta; verbatimEventDate: 15-Mar-2007; **Record Level:** language: en; institutionCode: CNC; collectionCode: Insects; basisOfRecord: Pinned Specimen
- **Type status:** Paratype. **Occurrence:** occurrenceDetails: http://janzen.sas.upenn.edu; catalogNumber: DHJPAR0011692; recordedBy: D.H. Janzen, W. Hallwachs & Roster Moraga; individualID: DHJPAR0011692; individualCount: 1; sex: M; lifeStage: adult; preparations: pinned; otherCatalogNumbers: 04-SRNP-25476, BOLD:AAA7512, ASTAS418-06; **Taxon:** scientificName: Calolydella adelinamoralesae; phylum: Arthropoda; class: Insecta; order: Diptera; family: Tachinidae; genus: Calolydella; specificEpithet: adelinamoralesae; scientificNameAuthorship: Fleming & Wood, 2016; **Location:** continent: Central America; country: Costa Rica; countryCode: CR; stateProvince: Guanacaste; county: Sector Del Oro; locality: Area de Conservacion Guanacaste; verbatimLocality: Uncaria; verbatimElevation: 370; verbatimLatitude: 11.0175; verbatimLongitude: -85.4741; verbatimCoordinateSystem: Decimal; **Identification:** identifiedBy: AJ Fleming; dateIdentified: 2016; **Event:** samplingProtocol: Reared from the larva of the arctiine erebid moth, Agaraea minuta; verbatimEventDate: 25-Oct-2004; **Record Level:** language: en; institutionCode: CNC; collectionCode: Insects; basisOfRecord: Pinned Specimen
- **Type status:** Paratype. **Occurrence:** occurrenceDetails: http://janzen.sas.upenn.edu; catalogNumber: DHJPAR0010355; recordedBy: D.H. Janzen, W. Hallwachs & Minor Carmona; individualID: DHJPAR0010355; individualCount: 1; sex: F; lifeStage: adult; preparations: pinned; otherCatalogNumbers: 06-SRNP-42840, BOLD:AAA7512, ASTAS186-06; **Taxon:** scientificName: Calolydella adelinamoralesae; phylum: Arthropoda; class: Insecta; order: Diptera; family: Tachinidae; genus: Calolydella; specificEpithet: adelinamoralesae; scientificNameAuthorship: Fleming & Wood, 2016; **Location:** continent: Central America; country: Costa Rica; countryCode: CR; stateProvince: Alajuela; county: Sector Rincon Rain Forest; locality: Area de Conservacion Guanacaste; verbatimLocality: Cabanya; verbatimElevation: 340; verbatimLatitude: 10.877; verbatimLongitude: -85.2308; verbatimCoordinateSystem: Decimal; **Identification:** identifiedBy: AJ Fleming; dateIdentified: 2016; **Event:** samplingProtocol: Reared from the larva of the arctiine erebid moth, Agaraea minuta; verbatimEventDate: 27-Aug-2006; **Record Level:** language: en; institutionCode: CNC; collectionCode: Insects; basisOfRecord: Pinned Specimen
- **Type status:** Paratype. **Occurrence:** occurrenceDetails: http://janzen.sas.upenn.edu; catalogNumber: DHJPAR0011698; recordedBy: D.H. Janzen, W. Hallwachs & Roster Moraga; individualID: DHJPAR0011698; individualCount: 1; sex: M; lifeStage: adult; preparations: pinned; otherCatalogNumbers: 04-SRNP-25331, BOLD:AAA7512, ASTAS424-06; **Taxon:** scientificName: Calolydella adelinamoralesae; phylum: Arthropoda; class: Insecta; order: Diptera; family: Tachinidae; genus: Calolydella; specificEpithet: adelinamoralesae; scientificNameAuthorship: Fleming & Wood, 2016; **Location:** continent: Central America; country: Costa Rica; countryCode: CR; stateProvince: Guanacaste; county: Sector Del Oro; locality: Area de Conservacion Guanacaste; verbatimLocality: Uncaria; verbatimElevation: 370; verbatimLatitude: 11.0175; verbatimLongitude: -85.4741; verbatimCoordinateSystem: Decimal; **Identification:** identifiedBy: AJ Fleming; dateIdentified: 2016; **Event:** samplingProtocol: Reared from the larva of the arctiine erebid moth, Agaraea minuta; verbatimEventDate: 26-Oct-2004; **Record Level:** language: en; institutionCode: CNC; collectionCode: Insects; basisOfRecord: Pinned Specimen
- **Type status:** Paratype. **Occurrence:** occurrenceDetails: http://janzen.sas.upenn.edu; catalogNumber: DHJPAR0011697; recordedBy: D.H. Janzen, W. Hallwachs & Roster Moraga; individualID: DHJPAR0011697; individualCount: 1; sex: F; lifeStage: adult; preparations: pinned; otherCatalogNumbers: 04-SRNP-25475, BOLD:AAA7512, ASTAS423-06; **Taxon:** scientificName: Calolydella adelinamoralesae; phylum: Arthropoda; class: Insecta; order: Diptera; family: Tachinidae; genus: Calolydella; specificEpithet: adelinamoralesae; scientificNameAuthorship: Fleming & Wood, 2016; **Location:** continent: Central America; country: Costa Rica; countryCode: CR; stateProvince: Guanacaste; county: Sector Del Oro; locality: Area de Conservacion Guanacaste; verbatimLocality: Uncaria; verbatimElevation: 370; verbatimLatitude: 11.0175; verbatimLongitude: -85.4741; verbatimCoordinateSystem: Decimal; **Identification:** identifiedBy: AJ Fleming; dateIdentified: 2016; **Event:** samplingProtocol: Reared from the larva of the arctiine erebid moth, Agaraea minuta; verbatimEventDate: 26-Oct-2004; **Record Level:** language: en; institutionCode: CNC; collectionCode: Insects; basisOfRecord: Pinned Specimen
- **Type status:** Paratype. **Occurrence:** occurrenceDetails: http://janzen.sas.upenn.edu; catalogNumber: DHJPAR0035797; recordedBy: D.H. Janzen, W. Hallwachs & Cirilo Umana; individualID: DHJPAR0035797; individualCount: 1; sex: M; lifeStage: adult; preparations: pinned; otherCatalogNumbers: 09-SRNP-67673, BOLD:AAA7512, ASHYD1178-09; **Taxon:** scientificName: Calolydella adelinamoralesae; phylum: Arthropoda; class: Insecta; order: Diptera; family: Tachinidae; genus: Calolydella; specificEpithet: adelinamoralesae; scientificNameAuthorship: Fleming & Wood, 2016; **Location:** continent: Central America; country: Costa Rica; countryCode: CR; stateProvince: Alajuela; county: Sector Rincon Rain Forest; locality: Area de Conservacion Guanacaste; verbatimLocality: Palomo; verbatimElevation: 96; verbatimLatitude: 10.9619; verbatimLongitude: -85.2804; verbatimCoordinateSystem: Decimal; **Identification:** identifiedBy: AJ Fleming; dateIdentified: 2016; **Event:** samplingProtocol: Reared from the larva of the arctiine erebid moth, Agaraea minuta; verbatimEventDate: 24-Jul-2009; **Record Level:** language: en; institutionCode: CNC; collectionCode: Insects; basisOfRecord: Pinned Specimen
- **Type status:** Paratype. **Occurrence:** occurrenceDetails: http://janzen.sas.upenn.edu; catalogNumber: DHJPAR0017078; recordedBy: D.H. Janzen, W. Hallwachs & Manuel Rios; individualID: DHJPAR0017078; individualCount: 1; sex: M; lifeStage: adult; preparations: pinned; otherCatalogNumbers: 07-SRNP-31251, BOLD:AAA7512, ASTAP516-07; **Taxon:** scientificName: Calolydella adelinamoralesae; phylum: Arthropoda; class: Insecta; order: Diptera; family: Tachinidae; genus: Calolydella; specificEpithet: adelinamoralesae; scientificNameAuthorship: Fleming & Wood, 2016; **Location:** continent: Central America; country: Costa Rica; countryCode: CR; stateProvince: Guanacaste; county: Sector Pitilla; locality: Area de Conservacion Guanacaste; verbatimLocality: Pasmompa; verbatimElevation: 440; verbatimLatitude: 11.0193; verbatimLongitude: -85.41; verbatimCoordinateSystem: Decimal; **Identification:** identifiedBy: AJ Fleming; dateIdentified: 2016; **Event:** samplingProtocol: Reared from the larva of the arctiine erebid moth, Agaraea minuta; verbatimEventDate: 14-Mar-2007; **Record Level:** language: en; institutionCode: CNC; collectionCode: Insects; basisOfRecord: Pinned Specimen
- **Type status:** Paratype. **Occurrence:** occurrenceDetails: http://janzen.sas.upenn.edu; catalogNumber: DHJPAR0017040; recordedBy: D.H. Janzen, W. Hallwachs & Manuel Rios; individualID: DHJPAR0017040; individualCount: 1; sex: F; lifeStage: adult; preparations: pinned; otherCatalogNumbers: 07-SRNP-31250, BOLD:AAA7512, ASTAP478-07; **Taxon:** scientificName: Calolydella adelinamoralesae; phylum: Arthropoda; class: Insecta; order: Diptera; family: Tachinidae; genus: Calolydella; specificEpithet: adelinamoralesae; scientificNameAuthorship: Fleming & Wood, 2016; **Location:** continent: Central America; country: Costa Rica; countryCode: CR; stateProvince: Guanacaste; county: Sector Pitilla; locality: Area de Conservacion Guanacaste; verbatimLocality: Pasmompa; verbatimElevation: 440; verbatimLatitude: 11.0193; verbatimLongitude: -85.41; verbatimCoordinateSystem: Decimal; **Identification:** identifiedBy: AJ Fleming; dateIdentified: 2016; **Event:** samplingProtocol: Reared from the larva of the arctiine erebid moth, Agaraea minuta; verbatimEventDate: 16-Mar-2007; **Record Level:** language: en; institutionCode: CNC; collectionCode: Insects; basisOfRecord: Pinned Specimen
- **Type status:** Paratype. **Occurrence:** occurrenceDetails: http://janzen.sas.upenn.edu; catalogNumber: DHJPAR0035786; recordedBy: D.H. Janzen, W. Hallwachs & Cirilo Umana; individualID: DHJPAR0035786; individualCount: 1; sex: M; lifeStage: adult; preparations: pinned; otherCatalogNumbers: 09-SRNP-67652, BOLD:AAA7512, ASHYD1167-09; **Taxon:** scientificName: Calolydella adelinamoralesae; phylum: Arthropoda; class: Insecta; order: Diptera; family: Tachinidae; genus: Calolydella; specificEpithet: adelinamoralesae; scientificNameAuthorship: Fleming & Wood, 2016; **Location:** continent: Central America; country: Costa Rica; countryCode: CR; stateProvince: Alajuela; county: Sector Rincon Rain Forest; locality: Area de Conservacion Guanacaste; verbatimLocality: Palomo; verbatimElevation: 96; verbatimLatitude: 10.9619; verbatimLongitude: -85.2804; verbatimCoordinateSystem: Decimal; **Identification:** identifiedBy: AJ Fleming; dateIdentified: 2016; **Event:** samplingProtocol: Reared from the larva of the arctiine erebid moth, Agaraea minuta; verbatimEventDate: 22-Jul-2009; **Record Level:** language: en; institutionCode: CNC; collectionCode: Insects; basisOfRecord: Pinned Specimen
- **Type status:** Paratype. **Occurrence:** occurrenceDetails: http://janzen.sas.upenn.edu; catalogNumber: DHJPAR0011703; recordedBy: D.H. Janzen, W. Hallwachs & Roster Moraga; individualID: DHJPAR0011703; individualCount: 1; sex: F; lifeStage: adult; preparations: pinned; otherCatalogNumbers: 04-SRNP-25381, BOLD:AAA7512, ASTAS429-06; **Taxon:** scientificName: Calolydella adelinamoralesae; phylum: Arthropoda; class: Insecta; order: Diptera; family: Tachinidae; genus: Calolydella; specificEpithet: adelinamoralesae; scientificNameAuthorship: Fleming & Wood, 2016; **Location:** continent: Central America; country: Costa Rica; countryCode: CR; stateProvince: Guanacaste; county: Sector Del Oro; locality: Area de Conservacion Guanacaste; verbatimLocality: Uncaria; verbatimElevation: 370; verbatimLatitude: 11.0175; verbatimLongitude: -85.4741; verbatimCoordinateSystem: Decimal; **Identification:** identifiedBy: AJ Fleming; dateIdentified: 2016; **Event:** samplingProtocol: Reared from the larva of the arctiine erebid moth, Agaraea minuta; verbatimEventDate: 31-Oct-2004; **Record Level:** language: en; institutionCode: CNC; collectionCode: Insects; basisOfRecord: Pinned Specimen
- **Type status:** Paratype. **Occurrence:** occurrenceDetails: http://janzen.sas.upenn.edu; catalogNumber: DHJPAR0017080; recordedBy: D.H. Janzen, W. Hallwachs & Manuel Rios; individualID: DHJPAR0017080; individualCount: 1; sex: F; lifeStage: adult; preparations: pinned; otherCatalogNumbers: 07-SRNP-31254, BOLD:AAA7512, ASTAP518-07; **Taxon:** scientificName: Calolydella adelinamoralesae; phylum: Arthropoda; class: Insecta; order: Diptera; family: Tachinidae; genus: Calolydella; specificEpithet: adelinamoralesae; scientificNameAuthorship: Fleming & Wood, 2016; **Location:** continent: Central America; country: Costa Rica; countryCode: CR; stateProvince: Guanacaste; county: Sector Pitilla; locality: Area de Conservacion Guanacaste; verbatimLocality: Pasmompa; verbatimElevation: 440; verbatimLatitude: 11.0193; verbatimLongitude: -85.41; verbatimCoordinateSystem: Decimal; **Identification:** identifiedBy: AJ Fleming; dateIdentified: 2016; **Event:** samplingProtocol: Reared from the larva of the arctiine erebid moth, Agaraea minuta; verbatimEventDate: 18-Mar-2007; **Record Level:** language: en; institutionCode: CNC; collectionCode: Insects; basisOfRecord: Pinned Specimen
- **Type status:** Paratype. **Occurrence:** occurrenceDetails: http://janzen.sas.upenn.edu; catalogNumber: DHJPAR0011702; recordedBy: D.H. Janzen, W. Hallwachs & Roster Moraga; individualID: DHJPAR0011702; individualCount: 1; sex: M; lifeStage: adult; preparations: pinned; otherCatalogNumbers: 04-SRNP-25485, BOLD:AAA7512, ASTAS428-06; **Taxon:** scientificName: Calolydella adelinamoralesae; phylum: Arthropoda; class: Insecta; order: Diptera; family: Tachinidae; genus: Calolydella; specificEpithet: adelinamoralesae; scientificNameAuthorship: Fleming & Wood, 2016; **Location:** continent: Central America; country: Costa Rica; countryCode: CR; stateProvince: Guanacaste; county: Sector Del Oro; locality: Area de Conservacion Guanacaste; verbatimLocality: Uncaria; verbatimElevation: 370; verbatimLatitude: 11.0175; verbatimLongitude: -85.4741; verbatimCoordinateSystem: Decimal; **Identification:** identifiedBy: AJ Fleming; dateIdentified: 2016; **Event:** samplingProtocol: Reared from the larva of the arctiine erebid moth, Agaraea minuta; verbatimEventDate: 31-Oct-2004; **Record Level:** language: en; institutionCode: CNC; collectionCode: Insects; basisOfRecord: Pinned Specimen
- **Type status:** Paratype. **Occurrence:** occurrenceDetails: http://janzen.sas.upenn.edu; catalogNumber: DHJPAR0035780; recordedBy: D.H. Janzen, W. Hallwachs & Cirilo Umana; individualID: DHJPAR0035780; individualCount: 1; sex: F; lifeStage: adult; preparations: pinned; otherCatalogNumbers: 09-SRNP-67684, BOLD:AAA7512, ASHYD1161-09; **Taxon:** scientificName: Calolydella adelinamoralesae; phylum: Arthropoda; class: Insecta; order: Diptera; family: Tachinidae; genus: Calolydella; specificEpithet: adelinamoralesae; scientificNameAuthorship: Fleming & Wood, 2016; **Location:** continent: Central America; country: Costa Rica; countryCode: CR; stateProvince: Alajuela; county: Sector Rincon Rain Forest; locality: Area de Conservacion Guanacaste; verbatimLocality: Palomo; verbatimElevation: 96; verbatimLatitude: 10.9619; verbatimLongitude: -85.2804; verbatimCoordinateSystem: Decimal; **Identification:** identifiedBy: AJ Fleming; dateIdentified: 2016; **Event:** samplingProtocol: Reared from the larva of the arctiine erebid moth, Agaraea minuta; verbatimEventDate: 25-Jul-2009; **Record Level:** language: en; institutionCode: CNC; collectionCode: Insects; basisOfRecord: Pinned Specimen

#### Description

**Male** (Fig. 1a, b, c). Length: 5–8mm. **Head** (Fig. 1b): frontal setae extending to base of postpedicel; fronto-orbital plate gold and sparsely setulose; parafacial silver throughout. **Thorax** (Fig. 1a, c): gold on dorsal surface, silver laterally (>50% coverage); four thoracic vittae, these becoming almost fused postsuturally; postpronotum with three setae in a narrow triangle; 3:3 acrostichal setae; 2:4 dorsocentral setae; 1:3 intra-alar setae; 1:3 supra-alar setae; three katepisternal setae; anatergite bare (rarely setose, one specimen with two hair-like seta); scutellar discal setae situated as wide apart as subapical scutellar setae. Wing vein R_4+5_ with at most four small setulae dorsally at base. **Abdomen** (Fig. 1a, c): ground color dark brown-orange, with transverse marginal pollinose bands; abdominal pollinosity gold dorsally, silver ventrally, with an orange spot lateroventrally at base of ST1+2; T3 with one pair of median marginal setae and 1–2 pairs of discal setae; T4 with 1–2 pairs of discal setae. **Terminalia** (Fig. 2): sternite 5 with two small lobes and a wide U-shaped median cleft, 0.39X the length of the sternite from lobe to apex (Fig. 2c); inner margin covered by dense pollinosity, appearing darker than surrounding cuticle; lobes of sternite with many short stout setae marginally. Cerci, in dorsal view (Fig. 2b), separated by a narrow gap; cercus, in lateral view, long, upturned, slender and pointed, slightly tapered apically from its already narrow base; cercus setose along its anterior 1/2. Surstylus (Fig. 2a), short and spatulate, 4/5 length of cercus, appearing rounded at apex when viewed laterally; apical half with multiple short setae; tip of surstylus slightly angled inwards when viewed dorsally.

**Female** (Fig. 1d, e, f). Length: 5–7mm. As male, except for the following characters: fronto-orbital plate 1.6X as wide as in male; 2:3 acrostichal setae; wing vein R_4+5_ with 6–7 setulae extending to crossvein R-M, and ground color of abdomen appearing slightly darker than in male.

#### Diagnosis

*Calolydella adelinamoralesae* can be distinguished from all other species of *Calolydella* by the following combination of traits: parafacial all silver, frontal setae extending to base of postpedicel, anatergite bare, and T4 with 1–2 pairs of discal setae.

#### Etymology

The specific epithet is in honor of Adelina Morales Chaves of La Cruz, Guanacaste, Costa Rica, in recognition of the many years she dedicated to sorting ACG Malaise trap samples, many of which contained *Calolydella* flies.

#### Distribution

Costa Rica, ACG, Alajuela and Guanacaste provinces, 96–527m.

#### Ecology

*Calolydella adelinamoralesae* has been reared 53 times from *Agaraea minuta* (Schaus, 1892) (Lepidoptera: Erebidae), in rain forest and dry-rain lowland intergrade.

### Calolydella alexanderjamesi

Fleming & Wood sp. n.

urn:lsid:zoobank.org:act:D19E48EA-474C-4B9D-B784-3D2E275CCEF1

#### Materials

- **Type status:** Holotype. **Occurrence:** occurrenceDetails: http://janzen.sas.upenn.edu; catalogNumber: DHJPAR0017782; recordedBy: D.H. Janzen, W. Hallwachs & gusaneros; individualID: DHJPAR0017782; individualCount: 1; sex: M; lifeStage: adult; preparations: pinned; otherCatalogNumbers: 99-SRNP-16175, BOLD:AAW8659, ASTAR493-07; **Taxon:** scientificName: Calolydella alexanderjamesi; phylum: Arthropoda; class: Insecta; order: Diptera; family: Tachinidae; genus: Calolydella; specificEpithet: alexanderjamesi; scientificNameAuthorship: Fleming & Wood, 2016; **Location:** continent: Central America; country: Costa Rica; countryCode: CR; stateProvince: Guanacaste; county: Sector Santa Rosa; locality: Area de Conservacion Guanacaste; verbatimLocality: Area Administrativa; verbatimElevation: 295; verbatimLatitude: 10.8376; verbatimLongitude: -85.6187; verbatimCoordinateSystem: Decimal; **Identification:** identifiedBy: AJ Fleming; dateIdentified: 2016; **Event:** samplingProtocol: Reared from the larva of the erebid moth, Correbia undulata; verbatimEventDate: 16-Nov-1999; **Record Level:** language: en; institutionCode: CNC; collectionCode: Insects; basisOfRecord: Pinned Specimen

#### Description

**Male** (Fig. 3a, b, c). Length: 7mm. **Head** (Fig. 3b): frontal setae extending to base of postpedicel; fronto-orbital plate gold, sparsely setulose throughout; parafacial at least 50% silver pollinose. **Thorax** (Fig. 3a, c): gold pollinose dorsally with a gold tinge on lateral surfaces; outermost two thoracic vittae twice as wide as innermost two, these becoming fused postsuturally; postpronotum with two setae (inner basal seta absent); 3:3 acrostichal setae; 3:4 dorsocentral setae; 2:2 intra-alar setae; 2:3 supra-alar setae; three katepisternal setae; anatergite bare; scutellar discal setae situated as wide apart as subapical scutellar setae. Wing vein R4+5 with at most three small setulae dorsally, near base. **Abdomen** (Fig. 3a): ground color dark brown-orange with uninterrupted transverse marginal pollinose bands; abdominal pollinosity gold dorsally, silver ventrally, with an orange spot lateroventrally at base of ST1+2; T3 with one pair of median marginal setae and one pair of discal setae; T4 with one pair of discal setae. **Terminalia**: not examined.

**Female**: not known at this time.

#### Diagnosis

*Calolydella alexanderjamesi* can be distinguished from all other species of *Calolydella* by the following combination of traits: parafacial mostly gold, fronto-orbital plate with small black setulae interspersed among frontal setae, anatergite bare, and thoracic pollinosity gold on both dorsal and lateral surfaces.

#### Etymology

The specific epithet is in honor of Alexander James of Levittown, Pennsylvania, in recognition of the moral and family support of his mother, Tanya Dapkey, in her efforts curating and preparing ACG parasitoid flies for DNA barcoding.

#### Distribution

Costa Rica, ACG, Guanacaste Province, Area Administrativa, 295m.

#### Ecology

*Calolydella alexanderjamesi* has been reared once from *Correbia undulata* (Druce, 1884) (Lepidoptera: Erebidae), in dry forest.

### Calolydella argentea

Fleming & Wood sp. n.

urn:lsid:zoobank.org:act:DB3A83D1-98DF-498E-B4BA-6A3736CDE596

#### Materials

- **Type status:** Holotype. **Occurrence:** occurrenceDetails: http://janzen.sas.upenn.edu; catalogNumber: DHJPAR0042289; recordedBy: D.H. Janzen, W. Hallwachs & Cirilo Umana; individualID: DHJPAR0042289; individualCount: 1; sex: F; lifeStage: adult; preparations: pinned; otherCatalogNumbers: 11-SRNP-75105, BOLD:ABA7233, ASHYH053-11; **Taxon:** scientificName: Calolydella argentea; phylum: Arthropoda; class: Insecta; order: Diptera; family: Tachinidae; genus: Calolydella; specificEpithet: argentea; scientificNameAuthorship: Fleming & Wood, 2016; **Location:** continent: Central America; country: Costa Rica; countryCode: CR; stateProvince: Alajuela; county: Sector Rincon Rain Forest; locality: Area de Conservacion Guanacaste; verbatimLocality: Finca Esmeralda; verbatimElevation: 123; verbatimLatitude: 10.9355; verbatimLongitude: -85.2531; verbatimCoordinateSystem: Decimal; **Identification:** identifiedBy: AJ Fleming; dateIdentified: 2016; **Event:** samplingProtocol: Reared from the larva of the noctuid moth, Antiblemma Poole33; verbatimEventDate: 17-Mar-2011; **Record Level:** language: en; institutionCode: CNC; collectionCode: Insects; basisOfRecord: Pinned Specimen

#### Description

**Female** (Fig. 4a, b, c). Length: 6mm. **Head** (Fig. 4b): frontal setae extending to base of postpedicel; fronto-orbital plate all silver, sparsely setulose throughout; parafacial silver throughout. **Thorax** (Fig. 4a, c): silver pollinose on both dorsal and lateral surfaces; with four regular thoracic vittae; postpronotum with three setae; 3:3 acrostichal setae; 2:3 dorsocentral setae; 2:3 intra-alar setae; 2:3 supra-alar setae; three katepisternal setae; anatergite with three or more setae, often in a small tuft; scutellar discal setae situated slightly wider apart than subapical scutellar setae. Wing vein R_4+5_ with 6–7 setulae dorsally, reaching from base almost to crossvein R-M. **Abdomen** (Fig. 4a): ground color black with uninterrupted transverse marginal pollinose bands covering anterior marginal 2/3 of tergites; both abdominal surfaces (dorsal and ventral) concolorous; base of ST1+2 black lateroventrally; T3 with one pair of median marginal setae and one pair of discal setae; T4 with one pair of discal setae.

**Male**: not known at this time.

#### Diagnosis

*Calolydella argentea* can be distinguished from all other species of *Calolydella* by the following combination of traits: parafacial and fronto-orbital plate entirely silver, frontal setae extending to base of postpedicel with intermingled small setulae, 3:3 acrostichal setae, anterior 2/3 of abdominal tergites with silver pollinose bands, anatergite with three or more hair-like setae, and wing vein R_4+5_ with 6–7 setulae, almost reaching to crossvein R-M.

#### Etymology

The specific epithet is derived from the Latin adjective "*argentum*", meaning silver, in reference to its entirely silver parafacial and fronto-orbital plate, a feature unique to this species.

#### Distribution

Costa Rica, ACG, Alajuela Province, Finca Esmeralda, 123m.

#### Ecology

*Calolydella argentea* has been reared once from *Antiblemma* Poole33 (Lepidoptera: Noctuidae), in rain forest.

### Calolydella aureofacies

Fleming & Wood sp. n.

urn:lsid:zoobank.org:act:EFC688A2-D2B7-4530-B9CD-47A41F29087C

#### Materials

- **Type status:** Holotype. **Occurrence:** occurrenceDetails: http://janzen.sas.upenn.edu; catalogNumber: DHJPAR0029582; recordedBy: D.H. Janzen, W. Hallwachs & Manuel Pereira; individualID: DHJPAR0029582; individualCount: 1; sex: F; lifeStage: adult; preparations: pinned; otherCatalogNumbers: 08-SRNP-36592, ASHYM1003-09; **Taxon:** scientificName: Calolydella aureofacies; phylum: Arthropoda; class: Insecta; order: Diptera; family: Tachinidae; genus: Calolydella; specificEpithet: aureofacies; scientificNameAuthorship: Fleming & Wood, 2016; **Location:** continent: Central America; country: Costa Rica; countryCode: CR; stateProvince: Guanacaste; county: Sector Cacao; locality: Area de Conservacion Guanacaste; verbatimLocality: Sendero Ponderosa; verbatimElevation: 1060; verbatimLatitude: 10.9146; verbatimLongitude: -85.4626; verbatimCoordinateSystem: Decimal; **Identification:** identifiedBy: AJ Fleming; dateIdentified: 2016; **Event:** samplingProtocol: Reared from the larvae of the geometrid moth, Cyclophora Janzen23; verbatimEventDate: 26-Sep-2008; **Record Level:** language: en; institutionCode: CNC; collectionCode: Insects; basisOfRecord: Pinned Specimen
- **Type status:** Paratype. **Occurrence:** occurrenceDetails: http://janzen.sas.upenn.edu; catalogNumber: DHJPAR0029601; recordedBy: D.H. Janzen, W. Hallwachs & Manuel Pereira; individualID: DHJPAR0029601; individualCount: 1; sex: F; lifeStage: adult; preparations: pinned; otherCatalogNumbers: 08-SRNP-36589, BOLD:AAH9540, ASHYM1022-09; **Taxon:** scientificName: Calolydella aureofacies; phylum: Arthropoda; class: Insecta; order: Diptera; family: Tachinidae; genus: Calolydella; specificEpithet: aureofacies; scientificNameAuthorship: Fleming & Wood, 2016; **Location:** continent: Central America; country: Costa Rica; countryCode: CR; stateProvince: Guanacaste; county: Sector Cacao; locality: Area de Conservacion Guanacaste; verbatimLocality: Sendero Ponderosa; verbatimElevation: 1060; verbatimLatitude: 10.9146; verbatimLongitude: -85.4626; verbatimCoordinateSystem: Decimal; **Identification:** identifiedBy: AJ Fleming; dateIdentified: 2016; **Event:** samplingProtocol: Reared from the larvae of the geometrid moth, Cyclophora Janzen23; verbatimEventDate: 26-Sep-2008; **Record Level:** language: en; institutionCode: CNC; collectionCode: Insects; basisOfRecord: Pinned Specimen

#### Description

**Female**: 6mm (Fig. 5). **Head** (Fig. 5b): frontal setae extending to base of postpedicel; fronto-orbital plate gold, sparsely setulose along upper half; parafacial almost entirely gold (>90% coverage); palps black basally. **Thorax** (Fig. 5a, c): gold on both dorsal and lateral surfaces; four regular thoracic vittae; postpronotum with three setae; 2:3 acrostichal setae; 2:3 dorsocentral setae; 2:3 intra-alar setae; 2:3 supra-alar setae; three katepisternal setae; anatergite with three or more hair-like setae, often in a small tuft; scutellar discal setae absent. Wing vein R_4+5_ with 5–7 setulae dorsally, extending almost to crossvein R-M. **Abdomen** (Fig. 5a): ground color black, typically with uninterrupted transverse marginal pollinose bands (these can sometimes appear interrupted by a thick median stripe under certain angles of light); both dorsal and ventral surfaces concolorous, silver; base of ST1+2 black lateroventrally; T3 with one pair of median marginal setae and one pair of discal setae; T4 with a pair of discal setae.

**Male**: not known at this time.

#### Diagnosis

*Calolydella aureofacies* can be distinguished from all other species of *Calolydella* by the following combination of traits: parafacial mostly gold, female palps black basally, anatergite with three or more hair-like setae, and scutellar discal setae absent.

#### Etymology

The specific epithet is derived from the Latin adjective "*aurum*", meaning gold, and "*facies*", meaning face, in reference to its gold parafacial and fronto-orbital plate.

#### Distribution

Costa Rica, ACG, Guanacaste Province, Sendero Ponderosa, 1060m.

#### Ecology

*Calolydella aureofacies* has been reared once from *Cyclophora* Janzen23 (Lepidoptera: Geometridae), in cloud forest.

### Calolydella bicolor

Fleming & Wood sp. n.

urn:lsid:zoobank.org:act:266003AB-9934-4077-92EC-A89D557EEE43

#### Materials

- **Type status:** Holotype. **Occurrence:** occurrenceDetails: http://janzen.sas.upenn.edu; catalogNumber: DHJPAR0054050; recordedBy: D.H. Janzen, W. Hallwachs & Sebastian Kleppe; individualID: DHJPAR0054050; individualCount: 1; sex: F; lifeStage: adult; preparations: pinned; otherCatalogNumbers: 13-SRNP-31573, BOLD:ACM2409, ASHYD3218-14; **Taxon:** scientificName: Calolydella bicolor; phylum: Arthropoda; class: Insecta; order: Diptera; family: Tachinidae; genus: Calolydella; specificEpithet: bicolor; scientificNameAuthorship: Fleming & Wood, 2016; **Location:** continent: Central America; country: Costa Rica; countryCode: CR; stateProvince: Guanacaste; county: Sector Pitilla; locality: Area de Conservacion Guanacaste; verbatimLocality: Sendero Bernales; verbatimElevation: 660; verbatimLatitude: 10.9835; verbatimLongitude: -85.4212; verbatimCoordinateSystem: Decimal; **Identification:** identifiedBy: AJ Fleming; dateIdentified: 2016; **Event:** samplingProtocol: Reared from the larva of the nymphalid butterfly, Callicore lyca; verbatimEventDate: 26-Nov-2013; **Record Level:** language: en; institutionCode: CNC; collectionCode: Insects; basisOfRecord: Pinned Specimen

#### Description

**Female** (Fig. 6a, b, c). Length: 7mm. **Head** (Fig. 6b): frontal setae extending to base of postpedicel; fronto-orbital plate gold throughout, ranging from bare to almost bare; parafacial silver throughout. **Thorax** (Fig. 6a, c): gold on dorsal surface, silver laterally (>50% coverage); vittae fused into two prominent dark stripes; postpronotum with three setae; 3:3 acrostichal setae; 2:3 dorsocentral setae; 1:3 intra-alar setae; 2:3 supra-alar setae; three katepisternal setae; anatergite with three or more hair-like setae, often in a small tuft; scutellar discal setae situated slightly closer together than subapical scutellar setae. Wing vein R_4+5_ with at most 2–3 setulae dorsally at base, not extending to crossvein R-M. **Abdomen** (Fig. 6a): ground color orange-yellow, with median dark stripe breaking up pollinose marginal banding; abdominal pollinosity gold dorsally, silver ventrally; base of ST1+2 black lateroventrally; T3 with a row of marginal setae and one pair of discal setae; T4 with one pair of discal setae.

**Male**: not known at this time.

#### Diagnosis

*Calolydella bicolor* can be distinguished from all other species of *Calolydella* by the following combination of traits: fronto-orbital plate entirely gold, parafacial entirely silver, anatergite with three or more hair-like setae, and gold abdominal pollinose bands interrupted by a median dark stripe.

#### Etymology

The specific epithet is derived from the Latin adjective “*bicolor*”, meaning two-toned, in reference to its gold fronto-orbital plate and silver parafacial.

#### Distribution

Costa Rica, ACG, Guanacaste Province, Sendero Bernales, 660m.

#### Ecology

*Calolydella bicolor* has been reared once from *Callicore lyca* (Doubleday, 1847) (Lepidoptera: Nymphalidae), in rain forest.

### Calolydella bifissus

Fleming & Wood sp. n.

urn:lsid:zoobank.org:act:11FFAD38-EC3E-48AD-81FB-6F45B23276DE

#### Materials

- **Type status:** Holotype. **Occurrence:** occurrenceDetails: http://janzen.sas.upenn.edu; catalogNumber: DHJPAR0017132; recordedBy: D.H. Janzen, W. Hallwachs & Jose Perez; individualID: DHJPAR0017132; individualCount: 1; sex: M; lifeStage: adult; preparations: pinned; otherCatalogNumbers: 07-SRNP-40315, BOLD:AAA1939, ASTAP570-07; **Taxon:** scientificName: Calolydella bifissus; phylum: Arthropoda; class: Insecta; order: Diptera; family: Tachinidae; genus: Calolydella; specificEpithet: bifissus; scientificNameAuthorship: Fleming & Wood, 2016; **Location:** continent: Central America; country: Costa Rica; countryCode: CR; stateProvince: Alajuela; county: Sector Rincon Rain Forest; locality: Area de Conservacion Guanacaste; verbatimLocality: Rio Francia Arriba; verbatimElevation: 400; verbatimLatitude: 10.8967; verbatimLongitude: -85.29; verbatimCoordinateSystem: Decimal; **Identification:** identifiedBy: AJ Fleming; dateIdentified: 2016; **Event:** samplingProtocol: Reared from the larva of the nymphalidae butterfly, Dircenna klugii; verbatimEventDate: 20-Feb-2007; **Record Level:** language: en; institutionCode: CNC; collectionCode: Insects; basisOfRecord: Pinned Specimen
- **Type status:** Paratype. **Occurrence:** occurrenceDetails: http://janzen.sas.upenn.edu; catalogNumber: DHJPAR0036636; recordedBy: D.H. Janzen, W. Hallwachs & Manuel Pereira; individualID: DHJPAR0036636; individualCount: 1; sex: M; lifeStage: adult; preparations: pinned; otherCatalogNumbers: 09-SRNP-36303, BOLD:AAA1939, ASHYE1547-09; **Taxon:** scientificName: Calolydella bifissus; phylum: Arthropoda; class: Insecta; order: Diptera; family: Tachinidae; genus: Calolydella; specificEpithet: bifissus; scientificNameAuthorship: Fleming & Wood, 2016; **Location:** continent: Central America; country: Costa Rica; countryCode: CR; stateProvince: Guanacaste; county: Sector Cacao; locality: Area de Conservacion Guanacaste; verbatimLocality: Estacion Cacao; verbatimElevation: 1150; verbatimLatitude: 10.9269; verbatimLongitude: -85.4682; verbatimCoordinateSystem: Decimal; **Identification:** identifiedBy: AJ Fleming; dateIdentified: 2016; **Event:** samplingProtocol: Reared from the larva of the geometridae moth, Phyllodonta latrata; verbatimEventDate: 19-Jul-2009; **Record Level:** language: en; institutionCode: CNC; collectionCode: Insects; basisOfRecord: Pinned Specimen
- **Type status:** Paratype. **Occurrence:** occurrenceDetails: http://janzen.sas.upenn.edu; catalogNumber: 07-SRNP-40315; recordedBy: D.H. Janzen, W. Hallwachs & Jose Perez; individualCount: 2, siblings of holotype; sex: 1M, 1F; lifeStage: adult; preparations: pinned; otherCatalogNumbers: 07-SRNP-40315; **Taxon:** scientificName: Calolydella bifissus; phylum: Arthropoda; class: Insecta; order: Diptera; family: Tachinidae; genus: Calolydella; specificEpithet: bifissus; scientificNameAuthorship: Fleming & Wood, 2016; **Location:** continent: Central America; country: Costa Rica; countryCode: CR; stateProvince: Alajuela; county: Sector Rincon Rain Forest; locality: Area de Conservacion Guanacaste; verbatimLocality: Rio Francia Arriba; verbatimElevation: 400; verbatimLatitude: 10.8967; verbatimLongitude: -85.29; verbatimCoordinateSystem: Decimal; **Identification:** identifiedBy: AJ Fleming; dateIdentified: 2016; **Event:** samplingProtocol: Reared from the larva of the nymphalidae butterfly, Dircenna klugii; verbatimEventDate: 20-Feb-2007; **Record Level:** language: en; institutionCode: CNC; collectionCode: Insects; basisOfRecord: Pinned Specimens

#### Description

**Male** (Fig. 7a, b, c). Length: 5–6mm. **Head** (Fig. 7b): frontal setae extending to base of postpedicel; frontal vitta tapering apically; fronto-orbital plate gold throughout, with a single row of fine setulae outside of frontal setae; parafacial at least 50% silver pollinose. **Thorax** (Fig. 7a, c): gold on dorsal surface, silver laterally (>50% coverage); vittae fused into two prominent dark stripes; postpronotum with two setae (inner basal seta absent); 1:3 acrostichal setae; 2:2 dorsocentral setae; 1:3 intra-alar setae; 1:3 supra-alar setae; three katepisternal setae; anatergite with three or more hair-like setae, often in a small tuft; scutellar discal setae situated slightly closer together than subapical scutellar setae. Wing vein R_4+5_ with at most 2–3 setulae dorsally at base, not extending to crossvein R-M. **Abdomen** (Fig. 7a): ground color dark brown-orange with uninterrupted transverse marginal pollinose bands; pollinosity gold dorsally, silver ventrally, with an orange spot lateroventrally at base of ST1+2; T3 with one pair of median marginal setae and one pair of discal setae; T4 with one pair of discal setae. **Terminalia** (Fig. 8): sternite 5 (Fig. 8c) with a wide U-shaped median cleft, 0.46X the length of the sternite from lobe to apex; each lobe with a smaller protrusion along upper margin of median cleft; inner margin covered by light pollinosity, appearing slightly darker than surrounding cuticle; lobe of sternite entirely covered with short setae, of varying lengths. Cerci with a fused base and cleft apex, slightly pinched at center (Fig. 8b); cercus, in lateral view, broad and with a slight downward bend. Surstylus digitiform and straight (Fig. 8a), appearing subequal to length of cercus when viewed laterally; surstylus with short setae along entire length; tip of surstylus appearing curved inwards when viewed dorsally.

**Female** (Fig. 7d, e, f). Length: 5mm. As male, except for the following characters: fronto-orbital plate 3X as wide as in male; frontal vitta not tapered, apically 1.2X as wide as in male; two katepisternal setae.

#### Diagnosis

*Calolydella bifissus* can be distinguished from all other species of *Calolydella* by the following combination of characters: two bold thoracic vittae, abdominal pollinosity concolorous on both dorsal and ventral surfaces, anatergite with a small tuft of hair-like setae, and abdominal ground color dark brown-orange.

#### Etymology

The specific epithet is derived from the Latin adjective "*bifissus*", meaning cleft or cloven, in reference to cleft nature of the cerci, which are fused basally, a character state unique to this species.

#### Distribution

Costa Rica, ACG, Alajuela and Guanacaste provinces, 400–1150m.

#### Ecology

*Calolydella bifissus* has been reared twice, from two separate hosts, *Dircenna kluggii* (Geyer, 1837) (Lepidoptera: Nymphalidae) and *Phyllodonta latrata* (Guenée, 1857) (Lepidoptera: Geometridae), in rain forest and cloud forest ecosystems.

### Calolydella crocata

Fleming & Wood sp. n.

urn:lsid:zoobank.org:act:252596B5-9766-4D84-AD8D-60846458BA5E

#### Materials

- **Type status:** Holotype. **Occurrence:** occurrenceDetails: http://janzen.sas.upenn.edu; catalogNumber: DHJPAR0053232; recordedBy: D.H. Janzen, W. Hallwachs & Ricardo Calero; individualID: DHJPAR0053232; individualCount: 1; sex: M; lifeStage: adult; preparations: pinned; otherCatalogNumbers: 13-SRNP-70888, BOLD:ACK3862, ASHYM2586-13; **Taxon:** scientificName: Calolydella crocata; phylum: Arthropoda; class: Insecta; order: Diptera; family: Tachinidae; genus: Calolydella; specificEpithet: crocata; scientificNameAuthorship: Fleming & Wood, 2016; **Location:** continent: Central America; country: Costa Rica; countryCode: CR; stateProvince: Guanacaste; county: Sector Pitilla; locality: Area de Conservacion Guanacaste; verbatimLocality: Estacion Quica; verbatimElevation: 470; verbatimLatitude: 10.997; verbatimLongitude: -85.3967; verbatimCoordinateSystem: Decimal; **Identification:** identifiedBy: AJ Fleming; dateIdentified: 2016; **Event:** samplingProtocol: Reared from the larva of the geometrid moth, Semaeopus Janzen17; verbatimEventDate: 25-Jun-2013; **Record Level:** language: en; institutionCode: CNC; collectionCode: Insects; basisOfRecord: Pinned Specimen
- **Type status:** Paratype. **Occurrence:** occurrenceDetails: http://janzen.sas.upenn.edu; catalogNumber: DHJPAR0055883; recordedBy: D.H. Janzen, W. Hallwachs & Dinia Martinez; individualID: DHJPAR0055883; individualCount: 1; sex: M; lifeStage: adult; preparations: pinned; otherCatalogNumbers: 14-SRNP-71256, BOLD:ACK3862, ASHYH2615-14; **Taxon:** scientificName: Calolydella crocata; phylum: Arthropoda; class: Insecta; order: Diptera; family: Tachinidae; genus: Calolydella; specificEpithet: crocata; scientificNameAuthorship: Fleming & Wood, 2016; **Location:** continent: Central America; country: Costa Rica; countryCode: CR; stateProvince: Guanacaste; county: Sector Pitilla; locality: Area de Conservacion Guanacaste; verbatimLocality: Medrano; verbatimElevation: 380; verbatimLatitude: 11.016; verbatimLongitude: -85.3805; verbatimCoordinateSystem: Decimal; **Identification:** identifiedBy: AJ Fleming; dateIdentified: 2016; **Event:** samplingProtocol: Reared from the larva of the geometrid moth, Semaeopus Janzen05; verbatimEventDate: 02-Aug-2014; **Record Level:** language: en; institutionCode: CNC; collectionCode: Insects; basisOfRecord: Pinned Specimen

#### Description

**Male** (Fig. 9a, b, c). Length: 6mm. **Head** (Fig. 9b): frontal setae extending to base of postpedicel; fronto-orbital plate entirely gold, and sparsely setulose only along upper half; parafacial silver. **Thorax**: (Fig. 9a, c) gold on both dorsal and lateral surfaces; thorax with four vittae visible only under certain angles of light; postpronotum with three setae; 3:3 acrostichal setae; 3:3 dorsocentral setae; 2:3 intra-alar setae; 2:3 supra-alar setae; three katepisternal setae; anatergite bare; scutellar discal setae absent. Wing vein R_4+5_ with 3–4 setulae dorsally at base. **Abdomen** (Fig. 9a): ground color black, with thin median dark stripe breaking up pollinose marginal banding; abdominal pollinosity gold dorsally, silver ventrally; base of ST1+2 black lateroventrally; T3 with one pair of median marginal setae and one pair of discal setae; T4 with one pair of discal setae. **Terminalia** (Fig. 10): sternite 5 (Fig. 10c) with two small lobes and a wide U-shaped median cleft, 0.39X the length of the sternite from lobe to apex; inner margin covered by dense pollinosity, appearing darker than surrounding cuticle; upper margin of sternite with many stout, outwardly-pointing setae. Cerci (Fig. 10b) separated by a narrow gap basally, widening to an oval about midway; cercus, in dorsal view, long and rounded and not significantly tapering from base to tip; cercus, in lateral view, straight and setose along basal half, with a slight bulb at its tip. Surstylus (Fig. 10a) short, 2/3 length of cercus, and knife-like, appearing sharply pointed when viewed laterally; apical half of surstylus with short setae; tip of surstylus slightly angled outwards when viewed dorsally.

**Female**: not known at this time.

#### Diagnosis

*Calolydella crocata* can be distinguished from all other species of *Calolydella* by the following combination of traits: fronto-orbital plate mostly gold-pollinose but with some silver pollinosity and sparsely setulose, thorax with four vittae visible only under certain angles of light, anatergite bare, and abdomen with transverse marginal pollinose bands interrupted by a thin dark median stripe.

#### Etymology

The specific epithet is derived from the Latin adjective "*crocatum*", meaning yellow, in reference to the gold pollinosity present over most of the body of the new species.

#### Distribution

Costa Rica, ACG, Guanacaste Province, Estación Quica, 470m.

#### Ecology

*Calolydella crocata* has been reared once from *Semaeopus* Janzen05 (Lepidoptera: Geometridae), in rain forest.

### Calolydella destituta

Fleming & Wood sp. n.

urn:lsid:zoobank.org:act:93C849D2-BF4A-4086-95EE-D69A4898598B

#### Materials

- **Type status:** Holotype. **Occurrence:** occurrenceDetails: http://janzen.sas.upenn.edu; catalogNumber: DHJPAR0017799; recordedBy: D.H. Janzen, W. Hallwachs & Roster Moraga; individualID: DHJPAR0017799; individualCount: 1; sex: M; lifeStage: adult; preparations: pinned; otherCatalogNumbers: 01-SRNP-11844, BOLD:AAE7382, ASTAR510-07; **Taxon:** scientificName: Calolydella destituta; phylum: Arthropoda; class: Insecta; order: Diptera; family: Tachinidae; genus: Calolydella; specificEpithet: destituta; scientificNameAuthorship: Fleming & Wood, 2016; **Location:** continent: Central America; country: Costa Rica; countryCode: CR; stateProvince: Guanacaste; county: Sector Del Oro; locality: Area de Conservacion Guanacaste; verbatimLocality: Mena Central; verbatimElevation: 345; verbatimLatitude: 11.0299; verbatimLongitude: -85.4536; verbatimCoordinateSystem: Decimal; **Identification:** identifiedBy: AJ Fleming; dateIdentified: 2016; **Event:** samplingProtocol: Reared from the larva of the erebid moth, Ramphia albizona; verbatimEventDate: 20-Nov-2001; **Record Level:** language: en; institutionCode: CNC; collectionCode: Insects; basisOfRecord: Pinned Specimen
- **Type status:** Paratype. **Occurrence:** occurrenceDetails: http://janzen.sas.upenn.edu; catalogNumber: DHJPAR0017768; recordedBy: D.H. Janzen, W. Hallwachs & Roster Moraga; individualID: DHJPAR0017768; individualCount: 1; sex: M; lifeStage: adult; preparations: pinned; otherCatalogNumbers: 01-SRNP-11820, BOLD:AAE7382, ASTAR479-07; **Taxon:** scientificName: Calolydella destituta; phylum: Arthropoda; class: Insecta; order: Diptera; family: Tachinidae; genus: Calolydella; specificEpithet: destituta; scientificNameAuthorship: Fleming & Wood, 2016; **Location:** continent: Central America; country: Costa Rica; countryCode: CR; stateProvince: Guanacaste; county: Sector Del Oro; locality: Area de Conservacion Guanacaste; verbatimLocality: Mena Central; verbatimElevation: 345; verbatimLatitude: 11.0299; verbatimLongitude: -85.4536; verbatimCoordinateSystem: Decimal; **Identification:** identifiedBy: AJ Fleming; dateIdentified: 2016; **Event:** samplingProtocol: Reared from the larva of the erebid moth, Ramphia albizona; verbatimEventDate: 20-Nov-2001; **Record Level:** language: en; institutionCode: CNC; collectionCode: Insects; basisOfRecord: Pinned Specimen
- **Type status:** Paratype. **Occurrence:** occurrenceDetails: http://janzen.sas.upenn.edu; catalogNumber: DHJPAR0017771; recordedBy: D.H. Janzen, W. Hallwachs & Roster Moraga; individualID: DHJPAR0017771; individualCount: 1; sex: F; lifeStage: adult; preparations: pinned; otherCatalogNumbers: 01-SRNP-11844, BOLD:AAE7382, ASTAR482-07; **Taxon:** scientificName: Calolydella destituta; phylum: Arthropoda; class: Insecta; order: Diptera; family: Tachinidae; genus: Calolydella; specificEpithet: destituta; scientificNameAuthorship: Fleming & Wood, 2016; **Location:** continent: Central America; country: Costa Rica; countryCode: CR; stateProvince: Guanacaste; county: Sector Del Oro; locality: Area de Conservacion Guanacaste; verbatimLocality: Mena Central; verbatimElevation: 345; verbatimLatitude: 11.0299; verbatimLongitude: -85.4536; verbatimCoordinateSystem: Decimal; **Identification:** identifiedBy: AJ Fleming; dateIdentified: 2016; **Event:** samplingProtocol: Reared from the larva of the erebid moth, Ramphia albizona; verbatimEventDate: 20-Nov-2001; **Record Level:** language: en; institutionCode: CNC; collectionCode: Insects; basisOfRecord: Pinned Specimen
- **Type status:** Paratype. **Occurrence:** occurrenceDetails: http://janzen.sas.upenn.edu; catalogNumber: DHJPAR0017776; recordedBy: D.H. Janzen, W. Hallwachs & Roster Moraga; individualID: DHJPAR0017776; individualCount: 1; sex: F; lifeStage: adult; preparations: pinned; otherCatalogNumbers: 01-SRNP-11820, BOLD:AAE7382, ASTAR487-07; **Taxon:** scientificName: Calolydella destituta; phylum: Arthropoda; class: Insecta; order: Diptera; family: Tachinidae; genus: Calolydella; specificEpithet: destituta; scientificNameAuthorship: Fleming & Wood, 2016; **Location:** continent: Central America; country: Costa Rica; countryCode: CR; stateProvince: Guanacaste; county: Sector Del Oro; locality: Area de Conservacion Guanacaste; verbatimLocality: Mena Central; verbatimElevation: 345; verbatimLatitude: 11.0299; verbatimLongitude: -85.4536; verbatimCoordinateSystem: Decimal; **Identification:** identifiedBy: AJ Fleming; dateIdentified: 2016; **Event:** samplingProtocol: Reared from the larva of the erebid moth, Ramphia albizona; verbatimEventDate: 20-Nov-2001; **Record Level:** language: en; institutionCode: CNC; collectionCode: Insects; basisOfRecord: Pinned Specimen

#### Description

**Male** (Fig. 11a, b, c). Length: 8–9mm. **Head** (Fig. 11b): frontal setae extending beyond base of postpedicel; fronto-orbital plate gold, sparsely setulose throughout; parafacial silver along lower half. **Thorax** (Fig. 11a, c): gold on dorsal surface, silver laterally (>50% coverage); four regular thoracic vittae; postpronotum with four setae; 3:3 acrostichal setae; 2:3 dorsocentral setae; 2:3 intra-alar setae; 2:3 supra-alar setae; three katepisternal setae; anatergite with three or more hair-like setae, often in a small tuft; scutellar discal setae situated as wide apart as subapical setae. Wing vein R_4+5_ with at most three small setulae dorsally at base. **Abdomen** (Fig. 11a): ground color dark orange-brown, with uninterrupted transverse marginal pollinose bands, and with an orange spot lateroventrally at base of ST1+2; T3 lacking median marginal and discal setae; T4 with one pair of discal setae. **Terminalia** (Fig. 12): sternite 5 (Fig. 12c) with two small lobes and a wide U-shaped median cleft, 0.37X the length of the sternite from lobe to apex; inner margin covered by dense pollinosity, appearing darker than surrounding cuticle; entire lobe of sternite with medium-length setae, all of equal length. Cerci (Fig. 12b), in dorsal view, separated by a gap, widening at apex; each cercus long and very slightly tapering from its already narrow base; cercus, when viewed laterally, setose along basal half, slender and slightly arched, slightly upturned apically. Surstylus (Fig. 12a) 5/6 the length of cercus, slender and digitiform when viewed laterally, with a sharp pinch where basal section meets apical section, 2X as wide as cercus; surstylus with short setae along its entire length; tip of surstylus curved inwards when viewed dorsally.

**Female** (Fig. 11d, e, f). Length: 5–6mm. As male, except for the following characters: fronto-orbital plate 2X as wide as in male; and T3 with one pair of median marginal setae and one pair of discal setae.

#### Diagnosis

*Calolydella destituta* can be distinguished from all other species of *Calolydella* by the following combination of traits: anatergite with a small tuft of setae, pollinose bands on abdomen not interrupted by a dark median stripe, and T3 lacking median marginal setae.

#### Etymology

The specific epithet is derived from the Latin adjective "*destituta*", meaning absent or lacking, in reference to the absence of median marginal setae on T3, a character state unique to this species.

#### Distribution

Costa Rica, ACG, Guanacaste Province, 345.

#### Ecology

*Calolydella destituta* has been reared four times from *Ramphia albizona* (Latreille, 1817) (Lepidoptera: Erebidae), in rain forest and dry-rain lowland intergrade.

### Calolydella discalis

Fleming & Wood sp. n.

urn:lsid:zoobank.org:act:584603D5-5F1C-457A-B7FC-6746A9EDA163

#### Materials

- **Type status:** Holotype. **Occurrence:** occurrenceDetails: http://janzen.sas.upenn.edu; catalogNumber: DHJPAR0017774; recordedBy: D.H. Janzen, W. Hallwachs & Androny Valdelomar; individualID: DHJPAR0017774; individualCount: 1; sex: M; lifeStage: adult; preparations: pinned; otherCatalogNumbers: 04-SRNP-196, BOLD:AAI6844, ASTAR485-07; **Taxon:** scientificName: Calolydella discalis; phylum: Arthropoda; class: Insecta; order: Diptera; family: Tachinidae; genus: Calolydella; specificEpithet: discalis; scientificNameAuthorship: Fleming & Wood, 2016; **Location:** continent: Central America; country: Costa Rica; countryCode: CR; stateProvince: Alajuela; county: Sector San Cristobal; locality: Area de Conservacion Guanacaste; verbatimLocality: Rio Blanco Abajo; verbatimElevation: 500; verbatimLatitude: 10.9004; verbatimLongitude: -85.3725; verbatimCoordinateSystem: Decimal; **Identification:** identifiedBy: AJ Fleming; dateIdentified: 2016; **Event:** samplingProtocol: Reared from the larva of the erebid moth, Turuptiana obliqua; verbatimEventDate: 04-Feb-2004; **Record Level:** language: en; institutionCode: CNC; collectionCode: Insects; basisOfRecord: Pinned Specimen
- **Type status:** Paratype. **Occurrence:** occurrenceDetails: http://janzen.sas.upenn.edu; catalogNumber: DHJPAR0017773; recordedBy: D.H. Janzen, W. Hallwachs & Androny Valdelomar; individualID: DHJPAR0017773; individualCount: 1; sex: M; lifeStage: adult; preparations: pinned; otherCatalogNumbers: 04-SRNP-211, BOLD:AAI6844, ASTAR484-07; **Taxon:** scientificName: Calolydella discalis; phylum: Arthropoda; class: Insecta; order: Diptera; family: Tachinidae; genus: Calolydella; specificEpithet: discalis; scientificNameAuthorship: Fleming & Wood, 2016; **Location:** continent: Central America; country: Costa Rica; countryCode: CR; stateProvince: Alajuela; county: Sector San Cristobal; locality: Area de Conservacion Guanacaste; verbatimLocality: Rio Blanco Abajo; verbatimElevation: 500; verbatimLatitude: 10.9004; verbatimLongitude: -85.3725; verbatimCoordinateSystem: Decimal; **Identification:** identifiedBy: AJ Fleming; dateIdentified: 2016; **Event:** samplingProtocol: Reared from the larva of the erebid moth, Turuptiana obliqua; verbatimEventDate: 03-Feb-2004; **Record Level:** language: en; institutionCode: CNC; collectionCode: Insects; basisOfRecord: Pinned Specimen

#### Description

**Male** (Fig. 13a, b, c). Length: 8–9mm. **Head** (Fig. 13b): frontal setae extending to base of postpedicel; fronto-orbital plate gold, with a single row of short setulae outside of frontal setae; parafacial at least 50% silver pollinose. **Thorax** (Fig. 13a, c): gold on both dorsal and lateral surfaces; outermost two thoracic vittae twice as wide as innermost two; scutellar discal setae situated as wide apart as subapical scutellar setae; postpronotum with three setae; 3:3 acrostichal setae; 2:3 dorsocentral setae; 3:3 intra-alar setae; 2:3 supra-alar setae; three katepisternal setae; anatergite with three or more hair-like setae, often in a small tuft. Wing vein R4+5 with 4–5 small setulae dorsally at base. **Abdomen** (Fig. 13a): ground color dark brown-orange with uninterrupted transverse marginal pollinose bands; abdominal pollinosity gold dorsally, silver ventrally, and with an orange spot lateroventrally at base of ST1+2; T3 with one pair of median marginal setae pair and two pairs of discal setae; T4 with two pairs of discal setae. **Terminalia** (Fig. 14): sternite 5 (Fig. 14c) with two small lobes and a wide U-shaped median cleft, 0.37X the length of the sternite from lobe to apex; inner margin covered by dense pollinosity, appearing darker than surrounding cuticle; entire lobe of sternite covered with short setae, all of equal length. Cerci (Fig. 14b), in dorsal view, narrow and tapered, separated by a narrow gap widening at apex; cercus long, slender and straight when viewed laterally, very slightly tapered from its already narrow base; setose along its basal half. Surstylus (Fig. 14a) subequal to length to cercus, slender and digitiform when viewed laterally; with short setae along entire length; tip of surstylus not curved inwards when viewed dorsally.

**Female**: not known at this time.

#### Diagnosis

*Calolydella discalis* can be distinguished from all other species of *Calolydella* by the following combination of traits: parafacial mostly gold, fronto-orbital plate with a single row of small black setulae outside of the frontal setae, anatergite with a small tuft of three or more hair-like setae, and T3 and T4 each with two pairs of discal setae.

#### Etymology

The specific epithet refers to the two pairs of discal setae on T3 and T4.

#### Distribution

Costa Rica, ACG, Alajuela Province, Rio Blanco Abajo, 500m.

#### Ecology

*Calolydella discalis* has been reared twice from *Turuptiana obliqua* Walker, 1869 (Lepidoptera: Erebidae: Arctiinae), in rain forest.

### Calolydella erasmocoronadoi

Fleming & Wood sp. n.

urn:lsid:zoobank.org:act:FAC2D446-637C-42C4-B744-69491257D313

#### Materials

- **Type status:** Holotype. **Occurrence:** occurrenceDetails: http://janzen.sas.upenn.edu; catalogNumber: DHJPAR0039246; recordedBy: D.H. Janzen, W. Hallwachs & Lucia Rios; individualID: DHJPAR0039246; individualCount: 1; sex: M; lifeStage: adult; preparations: pinned; otherCatalogNumbers: 10-SRNP-20642, BOLD:AAC8991, ASTAV809-10; **Taxon:** scientificName: Calolydella erasmocoronadoi; phylum: Arthropoda; class: Insecta; order: Diptera; family: Tachinidae; genus: Calolydella; specificEpithet: erasmocoronadoi; scientificNameAuthorship: Fleming & Wood, 2016; **Location:** continent: Central America; country: Costa Rica; countryCode: CR; stateProvince: Guanacaste; county: Sector Del Oro; locality: Area de Conservacion Guanacaste; verbatimLocality: Monte Cristo; verbatimElevation: 525; verbatimLatitude: 11.0137; verbatimLongitude: -85.4253; verbatimCoordinateSystem: Decimal; **Identification:** identifiedBy: AJ Fleming; dateIdentified: 2016; **Event:** samplingProtocol: Reared from the larva of the riodinid moth, Euselasia chrysippe; verbatimEventDate: 14-May-2010; **Record Level:** language: en; institutionCode: CNC; collectionCode: Insects; basisOfRecord: Pinned Specimen
- **Type status:** Paratype. **Occurrence:** occurrenceDetails: http://janzen.sas.upenn.edu; catalogNumber: DHJPAR0011706; recordedBy: D.H. Janzen, W. Hallwachs & Osvaldo Espinoza; individualID: DHJPAR0011706; individualCount: 1; sex: F; lifeStage: adult; preparations: pinned; otherCatalogNumbers: 04-SRNP-2399, BOLD:AAC8991, ASTAS432-06; **Taxon:** scientificName: Calolydella erasmocoronadoi; phylum: Arthropoda; class: Insecta; order: Diptera; family: Tachinidae; genus: Calolydella; specificEpithet: erasmocoronadoi; scientificNameAuthorship: Fleming & Wood, 2016; **Location:** continent: Central America; country: Costa Rica; countryCode: CR; stateProvince: Alajuela; county: Sector San Cristobal; locality: Area de Conservacion Guanacaste; verbatimLocality: Sendero Huerta; verbatimElevation: 527; verbatimLatitude: 10.9305; verbatimLongitude: -85.3722; verbatimCoordinateSystem: Decimal; **Identification:** identifiedBy: AJ Fleming; dateIdentified: 2016; **Event:** samplingProtocol: Reared from the larva of the riodinid moth, Euselasia chrysippe; verbatimEventDate: 17-Jun-2004; **Record Level:** language: en; institutionCode: CNC; collectionCode: Insects; basisOfRecord: Pinned Specimen
- **Type status:** Paratype. **Occurrence:** occurrenceDetails: http://janzen.sas.upenn.edu; catalogNumber: DHJPAR0037554; recordedBy: D.H. Janzen, W. Hallwachs & Noe Castillo; individualID: DHJPAR0037554; individualCount: 1; sex: F; lifeStage: adult; preparations: pinned; otherCatalogNumbers: 09-SRNP-69806, BOLD:AAC8991, ASHYC4299-10; **Taxon:** scientificName: Calolydella erasmocoronadoi; phylum: Arthropoda; class: Insecta; order: Diptera; family: Tachinidae; genus: Calolydella; specificEpithet: erasmocoronadoi; scientificNameAuthorship: Fleming & Wood, 2016; **Location:** continent: Central America; country: Costa Rica; countryCode: CR; stateProvince: Alajuela; county: Sector Rincon Rain Forest; locality: Area de Conservacion Guanacaste; verbatimLocality: Jacobo; verbatimElevation: 461; verbatimLatitude: 10.9408; verbatimLongitude: -85.3177; verbatimCoordinateSystem: Decimal; **Identification:** identifiedBy: AJ Fleming; dateIdentified: 2016; **Event:** samplingProtocol: Reared from the larva of the riodinid moth, Euselasia chrysippe; verbatimEventDate: 10-Sep-2009; **Record Level:** language: en; institutionCode: CNC; collectionCode: Insects; basisOfRecord: Pinned Specimen
- **Type status:** Paratype. **Occurrence:** occurrenceDetails: http://janzen.sas.upenn.edu; catalogNumber: DHJPAR0011688; recordedBy: D.H. Janzen, W. Hallwachs & Manuel Rios; individualID: DHJPAR0011688; individualCount: 1; sex: F; lifeStage: adult; preparations: pinned; otherCatalogNumbers: 04-SRNP-55482, BOLD:AAC8991, ASTAS414-06; **Taxon:** scientificName: Calolydella erasmocoronadoi; phylum: Arthropoda; class: Insecta; order: Diptera; family: Tachinidae; genus: Calolydella; specificEpithet: erasmocoronadoi; scientificNameAuthorship: Fleming & Wood, 2016; **Location:** continent: Central America; country: Costa Rica; countryCode: CR; stateProvince: Guanacaste; county: Sector Pitilla; locality: Area de Conservacion Guanacaste; verbatimLocality: Sendero Mismo; verbatimElevation: 680; verbatimLatitude: 10.9876; verbatimLongitude: -85.4197; verbatimCoordinateSystem: Decimal; **Identification:** identifiedBy: AJ Fleming; dateIdentified: 2016; **Event:** samplingProtocol: Reared from the larva of the riodinid moth, Euselasia chrysippe; verbatimEventDate: 19-Oct-2004; **Record Level:** language: en; institutionCode: CNC; collectionCode: Insects; basisOfRecord: Pinned Specimen
- **Type status:** Paratype. **Occurrence:** occurrenceDetails: http://janzen.sas.upenn.edu; catalogNumber: DHJPAR0039244; recordedBy: D.H. Janzen, W. Hallwachs & Lucia Rios; individualID: DHJPAR0039244; individualCount: 1; sex: M; lifeStage: adult; preparations: pinned; otherCatalogNumbers: 10-SRNP-20643, BOLD:AAC8991, ASTAV807-10; **Taxon:** scientificName: Calolydella erasmocoronadoi; phylum: Arthropoda; class: Insecta; order: Diptera; family: Tachinidae; genus: Calolydella; specificEpithet: erasmocoronadoi; scientificNameAuthorship: Fleming & Wood, 2016; **Location:** continent: Central America; country: Costa Rica; countryCode: CR; stateProvince: Guanacaste; county: Sector Del Oro; locality: Area de Conservacion Guanacaste; verbatimLocality: Monte Cristo; verbatimElevation: 525; verbatimLatitude: 11.0137; verbatimLongitude: -85.4253; verbatimCoordinateSystem: Decimal; **Identification:** identifiedBy: AJ Fleming; dateIdentified: 2016; **Event:** samplingProtocol: Reared from the larva of the riodinid moth, Euselasia chrysippe; verbatimEventDate: 11-May-2010; **Record Level:** language: en; institutionCode: CNC; collectionCode: Insects; basisOfRecord: Pinned Specimen
- **Type status:** Paratype. **Occurrence:** occurrenceDetails: http://janzen.sas.upenn.edu; catalogNumber: DHJPAR0039242; recordedBy: D.H. Janzen, W. Hallwachs & Lucia Rios; individualID: DHJPAR0039242; individualCount: 1; sex: M; lifeStage: adult; preparations: pinned; otherCatalogNumbers: 10-SRNP-20622, , ASTAV805-10; **Taxon:** scientificName: Calolydella erasmocoronadoi; phylum: Arthropoda; class: Insecta; order: Diptera; family: Tachinidae; genus: Calolydella; specificEpithet: erasmocoronadoi; scientificNameAuthorship: Fleming & Wood, 2016; **Location:** continent: Central America; country: Costa Rica; countryCode: CR; stateProvince: Guanacaste; county: Sector Del Oro; locality: Area de Conservacion Guanacaste; verbatimLocality: Monte Cristo; verbatimElevation: 525; verbatimLatitude: 11.0137; verbatimLongitude: -85.4253; verbatimCoordinateSystem: Decimal; **Identification:** identifiedBy: AJ Fleming; dateIdentified: 2016; **Event:** samplingProtocol: Reared from the larva of the riodinid moth, Euselasia chrysippe; verbatimEventDate: 11-May-2010; **Record Level:** language: en; institutionCode: CNC; collectionCode: Insects; basisOfRecord: Pinned Specimen
- **Type status:** Paratype. **Occurrence:** occurrenceDetails: http://janzen.sas.upenn.edu; catalogNumber: DHJPAR0039245; recordedBy: D.H. Janzen, W. Hallwachs & Lucia Rios; individualID: DHJPAR0039245; individualCount: 1; sex: M; lifeStage: adult; preparations: pinned; otherCatalogNumbers: 10-SRNP-20618, BOLD:AAC8991, ASTAV808-10; **Taxon:** scientificName: Calolydella erasmocoronadoi; phylum: Arthropoda; class: Insecta; order: Diptera; family: Tachinidae; genus: Calolydella; specificEpithet: erasmocoronadoi; scientificNameAuthorship: Fleming & Wood, 2016; **Location:** continent: Central America; country: Costa Rica; countryCode: CR; stateProvince: Guanacaste; county: Sector Del Oro; locality: Area de Conservacion Guanacaste; verbatimLocality: Monte Cristo; verbatimElevation: 525; verbatimLatitude: 11.0137; verbatimLongitude: -85.4253; verbatimCoordinateSystem: Decimal; **Identification:** identifiedBy: AJ Fleming; dateIdentified: 2016; **Event:** samplingProtocol: Reared from the larva of the riodinid moth, Euselasia chrysippe; verbatimEventDate: 11-May-2010; **Record Level:** language: en; institutionCode: CNC; collectionCode: Insects; basisOfRecord: Pinned Specimen
- **Type status:** Paratype. **Occurrence:** occurrenceDetails: http://janzen.sas.upenn.edu; catalogNumber: DHJPAR0037556; recordedBy: D.H. Janzen, W. Hallwachs & Noe Castillo; individualID: DHJPAR0037556; individualCount: 1; sex: M; lifeStage: adult; preparations: pinned; otherCatalogNumbers: 09-SRNP-69793, BOLD:AAC8991, ASHYC4301-10; **Taxon:** scientificName: Calolydella erasmocoronadoi; phylum: Arthropoda; class: Insecta; order: Diptera; family: Tachinidae; genus: Calolydella; specificEpithet: erasmocoronadoi; scientificNameAuthorship: Fleming & Wood, 2016; **Location:** continent: Central America; country: Costa Rica; countryCode: CR; stateProvince: Alajuela; county: Sector Rincon Rain Forest; locality: Area de Conservacion Guanacaste; verbatimLocality: Jacobo; verbatimElevation: 461; verbatimLatitude: 10.9408; verbatimLongitude: -85.3177; verbatimCoordinateSystem: Decimal; **Identification:** identifiedBy: AJ Fleming; dateIdentified: 2016; **Event:** samplingProtocol: Reared from the larva of the riodinid moth, Euselasia chrysippe; verbatimEventDate: 10-Sep-2009; **Record Level:** language: en; institutionCode: CNC; collectionCode: Insects; basisOfRecord: Pinned Specimen
- **Type status:** Paratype. **Occurrence:** occurrenceDetails: http://janzen.sas.upenn.edu; catalogNumber: DHJPAR0037555; recordedBy: D.H. Janzen, W. Hallwachs & Noe Castillo; individualID: DHJPAR0037555; individualCount: 1; sex: F; lifeStage: adult; preparations: pinned; otherCatalogNumbers: 09-SRNP-69805, BOLD:AAC8991, ASHYC4300-10; **Taxon:** scientificName: Calolydella erasmocoronadoi; phylum: Arthropoda; class: Insecta; order: Diptera; family: Tachinidae; genus: Calolydella; specificEpithet: erasmocoronadoi; scientificNameAuthorship: Fleming & Wood, 2016; **Location:** continent: Central America; country: Costa Rica; countryCode: CR; stateProvince: Alajuela; county: Sector Rincon Rain Forest; locality: Area de Conservacion Guanacaste; verbatimLocality: Jacobo; verbatimElevation: 461; verbatimLatitude: 10.9408; verbatimLongitude: -85.3177; verbatimCoordinateSystem: Decimal; **Identification:** identifiedBy: AJ Fleming; dateIdentified: 2016; **Event:** samplingProtocol: Reared from the larva of the riodinid moth, Euselasia chrysippe; verbatimEventDate: 10-Sep-2009; **Record Level:** language: en; institutionCode: CNC; collectionCode: Insects; basisOfRecord: Pinned Specimen
- **Type status:** Paratype. **Occurrence:** occurrenceDetails: http://janzen.sas.upenn.edu; catalogNumber: DHJPAR0039243; recordedBy: D.H. Janzen, W. Hallwachs & Lucia Rios; individualID: DHJPAR0039243; individualCount: 1; sex: F; lifeStage: adult; preparations: pinned; otherCatalogNumbers: 10-SRNP-20621, BOLD:AAC8991, ASTAV806-10; **Taxon:** scientificName: Calolydella erasmocoronadoi; phylum: Arthropoda; class: Insecta; order: Diptera; family: Tachinidae; genus: Calolydella; specificEpithet: erasmocoronadoi; scientificNameAuthorship: Fleming & Wood, 2016; **Location:** continent: Central America; country: Costa Rica; countryCode: CR; stateProvince: Guanacaste; county: Sector Del Oro; locality: Area de Conservacion Guanacaste; verbatimLocality: Monte Cristo; verbatimElevation: 525; verbatimLatitude: 11.0137; verbatimLongitude: -85.4253; verbatimCoordinateSystem: Decimal; **Identification:** identifiedBy: AJ Fleming; dateIdentified: 2016; **Event:** samplingProtocol: Reared from the larva of the riodinid moth, Euselasia chrysippe; verbatimEventDate: 11-May-2010; **Record Level:** language: en; institutionCode: CNC; collectionCode: Insects; basisOfRecord: Pinned Specimen
- **Type status:** Paratype. **Occurrence:** occurrenceDetails: http://janzen.sas.upenn.edu; catalogNumber: DHJPAR0011686; recordedBy: D.H. Janzen, W. Hallwachs & Manuel Rios; individualID: DHJPAR0011686; individualCount: 1; sex: M; lifeStage: adult; preparations: pinned; otherCatalogNumbers: 04-SRNP-55195, BOLD:AAC8991, ASTAS412-06; **Taxon:** scientificName: Calolydella erasmocoronadoi; phylum: Arthropoda; class: Insecta; order: Diptera; family: Tachinidae; genus: Calolydella; specificEpithet: erasmocoronadoi; scientificNameAuthorship: Fleming & Wood, 2016; **Location:** continent: Central America; country: Costa Rica; countryCode: CR; stateProvince: Guanacaste; county: Sector Pitilla; locality: Area de Conservacion Guanacaste; verbatimLocality: Sendero Mismo; verbatimElevation: 680; verbatimLatitude: 10.9876; verbatimLongitude: -85.4197; verbatimCoordinateSystem: Decimal; **Identification:** identifiedBy: AJ Fleming; dateIdentified: 2016; **Event:** samplingProtocol: Reared from the larva of the riodinid moth, Euselasia chrysippe; verbatimEventDate: 02-Oct-2004; **Record Level:** language: en; institutionCode: CNC; collectionCode: Insects; basisOfRecord: Pinned Specimen
- **Type status:** Paratype. **Occurrence:** occurrenceDetails: http://janzen.sas.upenn.edu; catalogNumber: DHJPAR0011687; recordedBy: D.H. Janzen, W. Hallwachs & Manuel Rios; individualID: DHJPAR0011687; individualCount: 1; sex: F; lifeStage: adult; preparations: pinned; otherCatalogNumbers: 04-SRNP-55483, BOLD:AAC8991, ASTAS413-06; **Taxon:** scientificName: Calolydella erasmocoronadoi; phylum: Arthropoda; class: Insecta; order: Diptera; family: Tachinidae; genus: Calolydella; specificEpithet: erasmocoronadoi; scientificNameAuthorship: Fleming & Wood, 2016; **Location:** continent: Central America; country: Costa Rica; countryCode: CR; stateProvince: Guanacaste; county: Sector Pitilla; locality: Area de Conservacion Guanacaste; verbatimLocality: Sendero Mismo; verbatimElevation: 680; verbatimLatitude: 10.9876; verbatimLongitude: -85.4197; verbatimCoordinateSystem: Decimal; **Identification:** identifiedBy: AJ Fleming; dateIdentified: 2016; **Event:** samplingProtocol: Reared from the larva of the riodinid moth, Euselasia chrysippe; verbatimEventDate: 20-Oct-2004; **Record Level:** language: en; institutionCode: CNC; collectionCode: Insects; basisOfRecord: Pinned Specimen
- **Type status:** Paratype. **Occurrence:** occurrenceDetails: http://janzen.sas.upenn.edu; catalogNumber: DHJPAR0011685; recordedBy: D.H. Janzen, W. Hallwachs & Manuel Rios; individualID: DHJPAR0011685; individualCount: 1; sex: M; lifeStage: adult; preparations: pinned; otherCatalogNumbers: 04-SRNP-55194, BOLD:AAC8991, ASTAS411-06; **Taxon:** scientificName: Calolydella erasmocoronadoi; phylum: Arthropoda; class: Insecta; order: Diptera; family: Tachinidae; genus: Calolydella; specificEpithet: erasmocoronadoi; scientificNameAuthorship: Fleming & Wood, 2016; **Location:** continent: Central America; country: Costa Rica; countryCode: CR; stateProvince: Guanacaste; county: Sector Pitilla; locality: Area de Conservacion Guanacaste; verbatimLocality: Sendero Mismo; verbatimElevation: 680; verbatimLatitude: 10.9876; verbatimLongitude: -85.4197; verbatimCoordinateSystem: Decimal; **Identification:** identifiedBy: AJ Fleming; dateIdentified: 2016; **Event:** samplingProtocol: Reared from the larva of the riodinid moth, Euselasia chrysippe; verbatimEventDate: 03-Oct-2004; **Record Level:** language: en; institutionCode: CNC; collectionCode: Insects; basisOfRecord: Pinned Specimen
- **Type status:** Paratype. **Occurrence:** occurrenceDetails: http://janzen.sas.upenn.edu; catalogNumber: DHJPAR0039251; recordedBy: D.H. Janzen, W. Hallwachs & Lucia Rios; individualID: DHJPAR0039251; individualCount: 1; sex: F; lifeStage: adult; preparations: pinned; otherCatalogNumbers: 10-SRNP-20639, BOLD:AAC8991, ASTAV814-10; **Taxon:** scientificName: Calolydella erasmocoronadoi; phylum: Arthropoda; class: Insecta; order: Diptera; family: Tachinidae; genus: Calolydella; specificEpithet: erasmocoronadoi; scientificNameAuthorship: Fleming & Wood, 2016; **Location:** continent: Central America; country: Costa Rica; countryCode: CR; stateProvince: Guanacaste; county: Sector Del Oro; locality: Area de Conservacion Guanacaste; verbatimLocality: Monte Cristo; verbatimElevation: 525; verbatimLatitude: 11.0137; verbatimLongitude: -85.4253; verbatimCoordinateSystem: Decimal; **Identification:** identifiedBy: AJ Fleming; dateIdentified: 2016; **Event:** samplingProtocol: Reared from the larva of the riodinid moth, Euselasia chrysippe; verbatimEventDate: 11-May-2010; **Record Level:** language: en; institutionCode: CNC; collectionCode: Insects; basisOfRecord: Pinned Specimen
- **Type status:** Paratype. **Occurrence:** occurrenceDetails: http://janzen.sas.upenn.edu; catalogNumber: DHJPAR0039250; recordedBy: D.H. Janzen, W. Hallwachs & Lucia Rios; individualID: DHJPAR0039250; individualCount: 1; sex: M; lifeStage: adult; preparations: pinned; otherCatalogNumbers: 10-SRNP-20619, BOLD:AAC8991, ASTAV813-10; **Taxon:** scientificName: Calolydella erasmocoronadoi; phylum: Arthropoda; class: Insecta; order: Diptera; family: Tachinidae; genus: Calolydella; specificEpithet: erasmocoronadoi; scientificNameAuthorship: Fleming & Wood, 2016; **Location:** continent: Central America; country: Costa Rica; countryCode: CR; stateProvince: Guanacaste; county: Sector Del Oro; locality: Area de Conservacion Guanacaste; verbatimLocality: Monte Cristo; verbatimElevation: 525; verbatimLatitude: 11.0137; verbatimLongitude: -85.4253; verbatimCoordinateSystem: Decimal; **Identification:** identifiedBy: AJ Fleming; dateIdentified: 2016; **Event:** samplingProtocol: Reared from the larva of the riodinid moth, Euselasia chrysippe; verbatimEventDate: 11-May-2010; **Record Level:** language: en; institutionCode: CNC; collectionCode: Insects; basisOfRecord: Pinned Specimen
- **Type status:** Paratype. **Occurrence:** occurrenceDetails: http://janzen.sas.upenn.edu; catalogNumber: DHJPAR0011707; recordedBy: D.H. Janzen, W. Hallwachs & Osvaldo Espinoza; individualID: DHJPAR0011707; individualCount: 1; sex: F; lifeStage: adult; preparations: pinned; otherCatalogNumbers: 04-SRNP-2393, BOLD:AAC8991, ASTAS433-06; **Taxon:** scientificName: Calolydella erasmocoronadoi; phylum: Arthropoda; class: Insecta; order: Diptera; family: Tachinidae; genus: Calolydella; specificEpithet: erasmocoronadoi; scientificNameAuthorship: Fleming & Wood, 2016; **Location:** continent: Central America; country: Costa Rica; countryCode: CR; stateProvince: Alajuela; county: Sector San Cristobal; locality: Area de Conservacion Guanacaste; verbatimLocality: Sendero Huerta; verbatimElevation: 527; verbatimLatitude: 10.9305; verbatimLongitude: -85.3722; verbatimCoordinateSystem: Decimal; **Identification:** identifiedBy: AJ Fleming; dateIdentified: 2016; **Event:** samplingProtocol: Reared from the larva of the riodinid moth, Euselasia chrysippe; verbatimEventDate: 03-Jun-2004; **Record Level:** language: en; institutionCode: CNC; collectionCode: Insects; basisOfRecord: Pinned Specimen
- **Type status:** Paratype. **Occurrence:** occurrenceDetails: http://janzen.sas.upenn.edu; catalogNumber: DHJPAR0011689; recordedBy: D.H. Janzen, W. Hallwachs & Manuel Rios; individualID: DHJPAR0011689; individualCount: 1; sex: F; lifeStage: adult; preparations: pinned; otherCatalogNumbers: 04-SRNP-55450, BOLD:AAC8991, ASTAS415-06; **Taxon:** scientificName: Calolydella erasmocoronadoi; phylum: Arthropoda; class: Insecta; order: Diptera; family: Tachinidae; genus: Calolydella; specificEpithet: erasmocoronadoi; scientificNameAuthorship: Fleming & Wood, 2016; **Location:** continent: Central America; country: Costa Rica; countryCode: CR; stateProvince: Guanacaste; county: Sector Pitilla; locality: Area de Conservacion Guanacaste; verbatimLocality: Sendero Mismo; verbatimElevation: 680; verbatimLatitude: 10.9876; verbatimLongitude: -85.4197; verbatimCoordinateSystem: Decimal; **Identification:** identifiedBy: AJ Fleming; dateIdentified: 2016; **Event:** samplingProtocol: Reared from the larva of the riodinid moth, Euselasia chrysippe; verbatimEventDate: 18-Oct-2004; **Record Level:** language: en; institutionCode: CNC; collectionCode: Insects; basisOfRecord: Pinned Specimen
- **Type status:** Paratype. **Occurrence:** occurrenceDetails: http://janzen.sas.upenn.edu; catalogNumber: DHJPAR0039248; recordedBy: D.H. Janzen, W. Hallwachs & Lucia Rios; individualID: DHJPAR0039248; individualCount: 1; sex: F; lifeStage: adult; preparations: pinned; otherCatalogNumbers: 10-SRNP-20624, BOLD:AAC8991, ASTAV811-10; **Taxon:** scientificName: Calolydella erasmocoronadoi; phylum: Arthropoda; class: Insecta; order: Diptera; family: Tachinidae; genus: Calolydella; specificEpithet: erasmocoronadoi; scientificNameAuthorship: Fleming & Wood, 2016; **Location:** continent: Central America; country: Costa Rica; countryCode: CR; stateProvince: Guanacaste; county: Sector Del Oro; locality: Area de Conservacion Guanacaste; verbatimLocality: Monte Cristo; verbatimElevation: 525; verbatimLatitude: 11.0137; verbatimLongitude: -85.4253; verbatimCoordinateSystem: Decimal; **Identification:** identifiedBy: AJ Fleming; dateIdentified: 2016; **Event:** samplingProtocol: Reared from the larva of the riodinid moth, Euselasia chrysippe; verbatimEventDate: 11-May-2010; **Record Level:** language: en; institutionCode: CNC; collectionCode: Insects; basisOfRecord: Pinned Specimen
- **Type status:** Paratype. **Occurrence:** occurrenceDetails: http://janzen.sas.upenn.edu; catalogNumber: DHJPAR0037553; recordedBy: D.H. Janzen, W. Hallwachs & Noe Castillo; individualID: DHJPAR0037553; individualCount: 1; sex: M; lifeStage: adult; preparations: pinned; otherCatalogNumbers: 09-SRNP-69808, BOLD:AAC8991, ASHYC4298-10; **Taxon:** scientificName: Calolydella erasmocoronadoi; phylum: Arthropoda; class: Insecta; order: Diptera; family: Tachinidae; genus: Calolydella; specificEpithet: erasmocoronadoi; scientificNameAuthorship: Fleming & Wood, 2016; **Location:** continent: Central America; country: Costa Rica; countryCode: CR; stateProvince: Alajuela; county: Sector Rincon Rain Forest; locality: Area de Conservacion Guanacaste; verbatimLocality: Jacobo; verbatimElevation: 461; verbatimLatitude: 10.9408; verbatimLongitude: -85.3177; verbatimCoordinateSystem: Decimal; **Identification:** identifiedBy: AJ Fleming; dateIdentified: 2016; **Event:** samplingProtocol: Reared from the larva of the riodinid moth, Euselasia chrysippe; verbatimEventDate: 08-Sep-2009; **Record Level:** language: en; institutionCode: CNC; collectionCode: Insects; basisOfRecord: Pinned Specimen
- **Type status:** Paratype. **Occurrence:** occurrenceDetails: http://janzen.sas.upenn.edu; catalogNumber: DHJPAR0011708; recordedBy: D.H. Janzen, W. Hallwachs & Osvaldo Espinoza; individualID: DHJPAR0011708; individualCount: 1; sex: F; lifeStage: adult; preparations: pinned; otherCatalogNumbers: 04-SRNP-2401, BOLD:AAC8991, ASTAS434-06; **Taxon:** scientificName: Calolydella erasmocoronadoi; phylum: Arthropoda; class: Insecta; order: Diptera; family: Tachinidae; genus: Calolydella; specificEpithet: erasmocoronadoi; scientificNameAuthorship: Fleming & Wood, 2016; **Location:** continent: Central America; country: Costa Rica; countryCode: CR; stateProvince: Alajuela; county: Sector San Cristobal; locality: Area de Conservacion Guanacaste; verbatimLocality: Sendero Huerta; verbatimElevation: 527; verbatimLatitude: 10.9305; verbatimLongitude: -85.3722; verbatimCoordinateSystem: Decimal; **Identification:** identifiedBy: AJ Fleming; dateIdentified: 2016; **Event:** samplingProtocol: Reared from the larva of the riodinid moth, Euselasia chrysippe; verbatimEventDate: 17-Jun-2004; **Record Level:** language: en; institutionCode: CNC; collectionCode: Insects; basisOfRecord: Pinned Specimen
- **Type status:** Paratype. **Occurrence:** occurrenceDetails: http://janzen.sas.upenn.edu; catalogNumber: DHJPAR0039247; recordedBy: D.H. Janzen, W. Hallwachs & Lucia Rios; individualID: DHJPAR0039247; individualCount: 1; sex: F; lifeStage: adult; preparations: pinned; otherCatalogNumbers: 10-SRNP-20640, BOLD:AAC8991, ASTAV810-10; **Taxon:** scientificName: Calolydella erasmocoronadoi; phylum: Arthropoda; class: Insecta; order: Diptera; family: Tachinidae; genus: Calolydella; specificEpithet: erasmocoronadoi; scientificNameAuthorship: Fleming & Wood, 2016; **Location:** continent: Central America; country: Costa Rica; countryCode: CR; stateProvince: Guanacaste; county: Sector Del Oro; locality: Area de Conservacion Guanacaste; verbatimLocality: Monte Cristo; verbatimElevation: 525; verbatimLatitude: 11.0137; verbatimLongitude: -85.4253; verbatimCoordinateSystem: Decimal; **Identification:** identifiedBy: AJ Fleming; dateIdentified: 2016; **Event:** samplingProtocol: Reared from the larva of the riodinid moth, Euselasia chrysippe; verbatimEventDate: 14-May-2010; **Record Level:** language: en; institutionCode: CNC; collectionCode: Insects; basisOfRecord: Pinned Specimen
- **Type status:** Paratype. **Occurrence:** occurrenceDetails: http://janzen.sas.upenn.edu; catalogNumber: DHJPAR0039249; recordedBy: D.H. Janzen, W. Hallwachs & Lucia Rios; individualID: DHJPAR0039249; individualCount: 1; sex: F; lifeStage: adult; preparations: pinned; otherCatalogNumbers: 10-SRNP-20623, BOLD:AAC8991, ASTAV812-10; **Taxon:** scientificName: Calolydella erasmocoronadoi; phylum: Arthropoda; class: Insecta; order: Diptera; family: Tachinidae; genus: Calolydella; specificEpithet: erasmocoronadoi; scientificNameAuthorship: Fleming & Wood, 2016; **Location:** continent: Central America; country: Costa Rica; countryCode: CR; stateProvince: Guanacaste; county: Sector Del Oro; locality: Area de Conservacion Guanacaste; verbatimLocality: Quebrada Serrano; verbatimElevation: 585; verbatimLatitude: 11.0002; verbatimLongitude: -85.4561; verbatimCoordinateSystem: Decimal; **Identification:** identifiedBy: AJ Fleming; dateIdentified: 2016; **Event:** samplingProtocol: Reared from the larva of the riodinid moth, Euselasia chrysippe; verbatimEventDate: 11-May-2010; **Record Level:** language: en; institutionCode: CNC; collectionCode: Insects; basisOfRecord: Pinned Specimen
- **Type status:** Paratype. **Occurrence:** occurrenceDetails: http://janzen.sas.upenn.edu; catalogNumber: DHJPAR0037558; recordedBy: D.H. Janzen, W. Hallwachs & Noe Castillo; individualID: DHJPAR0037558; individualCount: 1; sex: M; lifeStage: adult; preparations: pinned; otherCatalogNumbers: 09-SRNP-69807, BOLD:AAC8991, ASHYC4303-10; **Taxon:** scientificName: Calolydella erasmocoronadoi; phylum: Arthropoda; class: Insecta; order: Diptera; family: Tachinidae; genus: Calolydella; specificEpithet: erasmocoronadoi; scientificNameAuthorship: Fleming & Wood, 2016; **Location:** continent: Central America; country: Costa Rica; countryCode: CR; stateProvince: Alajuela; county: Sector Rincon Rain Forest; locality: Area de Conservacion Guanacaste; verbatimLocality: Jacobo; verbatimElevation: 461; verbatimLatitude: 10.9408; verbatimLongitude: -85.3177; verbatimCoordinateSystem: Decimal; **Identification:** identifiedBy: AJ Fleming; dateIdentified: 2016; **Event:** samplingProtocol: Reared from the larva of the riodinid moth, Euselasia chrysippe; verbatimEventDate: 14-Sep-2009; **Record Level:** language: en; institutionCode: CNC; collectionCode: Insects; basisOfRecord: Pinned Specimen
- **Type status:** Paratype. **Occurrence:** occurrenceDetails: http://janzen.sas.upenn.edu; catalogNumber: DHJPAR0011684; recordedBy: D.H. Janzen, W. Hallwachs & Manuel Rios; individualID: DHJPAR0011684; individualCount: 1; sex: F; lifeStage: adult; preparations: pinned; otherCatalogNumbers: 04-SRNP-55193, BOLD:AAC8991, ASTAS410-06; **Taxon:** scientificName: Calolydella erasmocoronadoi; phylum: Arthropoda; class: Insecta; order: Diptera; family: Tachinidae; genus: Calolydella; specificEpithet: erasmocoronadoi; scientificNameAuthorship: Fleming & Wood, 2016; **Location:** continent: Central America; country: Costa Rica; countryCode: CR; stateProvince: Guanacaste; county: Sector Pitilla; locality: Area de Conservacion Guanacaste; verbatimLocality: Sendero Mismo; verbatimElevation: 680; verbatimLatitude: 10.9876; verbatimLongitude: -85.4197; verbatimCoordinateSystem: Decimal; **Identification:** identifiedBy: AJ Fleming; dateIdentified: 2016; **Event:** samplingProtocol: Reared from the larva of the riodinid moth, Euselasia chrysippe; verbatimEventDate: 04-Oct-2004; **Record Level:** language: en; institutionCode: CNC; collectionCode: Insects; basisOfRecord: Pinned Specimen
- **Type status:** Paratype. **Occurrence:** occurrenceDetails: http://janzen.sas.upenn.edu; catalogNumber: 04-SRNP-55202; recordedBy: D.H. Janzen, W. Hallwachs & Manuel Rios; individualID: 04-SRNP-55202; individualCount: 1; sex: F; lifeStage: adult; preparations: pinned; otherCatalogNumbers: 04-SRNP-55202; **Taxon:** scientificName: Calolydella erasmocoronadoi; phylum: Arthropoda; class: Insecta; order: Diptera; family: Tachinidae; genus: Calolydella; specificEpithet: erasmocoronadoi; scientificNameAuthorship: Fleming & Wood, 2016; **Location:** continent: Central America; country: Costa Rica; countryCode: CR; stateProvince: Guanacaste; county: Sector Pitilla; locality: Area de Conservacion Guanacaste; verbatimLocality: Sendero Mismo; verbatimElevation: 680; verbatimLatitude: 10.9876; verbatimLongitude: -85.4197; verbatimCoordinateSystem: Decimal; **Identification:** identifiedBy: AJ Fleming; dateIdentified: 2016; **Event:** samplingProtocol: Reared from the larva of the riodinid moth, Euselasia chrysippe; verbatimEventDate: 04-Oct-2004; **Record Level:** language: en; institutionCode: CNC; collectionCode: Insects; basisOfRecord: Pinned Specimen

#### Description

**Male** (Fig. 15a, b, c). Length: 6–8mm. **Head** (Fig. 15b): frontal setae extending to base of postpedicel; fronto-orbital plate up to 50% silver pollinose, almost bare, with a few small setulae interspersed among frontal setae; parafacial silver throughout. **Thorax** (Fig. 15a, c): gold on dorsal surface, silver laterally (>50% coverage); thoracic vittae visible only under certain angles of light; postpronotum with three setae; 3:3 acrostichal setae; 3:3 dorsocentral setae; 1:3 intra-alar setae; 2:3 supra-alar setae; three katepisternal setae; anatergite with three or more hair-like setae, often in a small tuft; scutellar discal setae situated as wide apart as subapical scutellar setae. Wing vein R_4+5_ with 6–7 setulae dorsally, extending halfway to crossvein R-M. **Abdomen** (Fig. 15a): ground color black with uninterrupted transverse gold pollinose bands; abdominal pollinosity gold dorsally, silver ventrally; base of ST1+2 black lateroventrally; T3 with one pair of median marginal setae and one pair of discal setae; T4 with one pair of discal setae. **Terminalia** (Fig. 16): sternite 5 (Fig. 16c) with two small lobes and a wide U-shaped median cleft, 0.34X the length of the sternite from lobe to apex; inner margin covered by dense pollinosity, appearing darker than surrounding cuticle; upper half of sternite with many stout, outwardly-pointing setae. Cerci (Fig. 16b), in dorsal view, separated by a narrow gap; each cercus long and rounded, and not significantly tapering from base to tip; cercus straight when viewed laterally, with a slight downward hook at its tip, setose almost along its entire length. Surstylus (Fig. 16c) stout and digitiform; apical half with short setae; tip of surstylus not lobed or angled inwards when viewed dorsally; subequal in length to cercus.

**Female** (Fig. 15d, e, f). Length: 4–7mm. Fronto-orbital plate 2.5X as wide as in male.

#### Diagnosis

*Calolydella erasmocoronadoi* can be distinguished from all other species of *Calolydella* by the following combination of traits: fronto-orbital plate bare, up to 50% silver pollinose, thoracic vittae visible only under certain angles of light, anatergite with three or more setae arranged in a small tuft, wing vein R_4+5_ with 6–7 setulae, and abdomen with uninterrupted transverse marginal pollinose bands.

#### Etymology

The specific epithet is in honor of Erasmo Coronado Caballo of Liberia, Costa Rica, in recognition of his high-quality assistantship to Sigifredo Marin of Liberia and to the ACG parataxonomists who collected and reared the caterpillars from which the new species of ACG *Calolydella* described here emerged.

#### Distribution

Costa Rica, ACG, Guanacaste Province, Sendero Mismo, 680m.

#### Ecology

*Calolydella erasmocoronadoi* has been reared 42 times from *Euselasia chryssipe* (Bates, 1866) (Lepidoptera: Riodinidae), in rain forest.

### Calolydella felipechavarriai

Fleming & Wood sp. n.

urn:lsid:zoobank.org:act:538AEEF3-B6C0-458D-B5D3-518CBD726E0E

#### Materials

- **Type status:** Holotype. **Occurrence:** occurrenceDetails: http://janzen.sas.upenn.edu; catalogNumber: DHJPAR0050491; recordedBy: D.H. Janzen, W. Hallwachs & Keiner Aragon; individualID: DHJPAR0050491; individualCount: 1; sex: M; lifeStage: adult; preparations: pinned; otherCatalogNumbers: 12-SRNP-79033, BOLD:ACJ3769, ACGBA3083-13; **Taxon:** scientificName: Calolydella felipechavarriai; phylum: Arthropoda; class: Insecta; order: Diptera; family: Tachinidae; genus: Calolydella; specificEpithet: felipechavarriai; scientificNameAuthorship: Fleming & Wood, 2016; **Location:** continent: Central America; country: Costa Rica; countryCode: CR; stateProvince: Alajuela; county: Sector Rincon Rain Forest; locality: Area de Conservacion Guanacaste; verbatimLocality: Palomo; verbatimElevation: 96; verbatimLatitude: 10.9619; verbatimLongitude: -85.2804; verbatimCoordinateSystem: Decimal; **Identification:** identifiedBy: AJ Fleming; dateIdentified: 2016; **Event:** samplingProtocol: Reared from the larva of the notodontid moth, Disphragis perses; verbatimEventDate: 08-Jan-2013; **Record Level:** language: en; institutionCode: CNC; collectionCode: Insects; basisOfRecord: Pinned Specimen
- **Type status:** Paratype. **Occurrence:** occurrenceDetails: http://janzen.sas.upenn.edu; catalogNumber: DHJPAR0050489; recordedBy: D.H. Janzen, W. Hallwachs & Keiner Aragon; individualID: DHJPAR0050489; individualCount: 1; sex: F; lifeStage: adult; preparations: pinned; otherCatalogNumbers: 12-SRNP-79034, BOLD:ACJ3769, ACGBA3081-13; **Taxon:** scientificName: Calolydella felipechavarriai; phylum: Arthropoda; class: Insecta; order: Diptera; family: Tachinidae; genus: Calolydella; specificEpithet: felipechavarriai; scientificNameAuthorship: Fleming & Wood, 2016; **Location:** continent: Central America; country: Costa Rica; countryCode: CR; stateProvince: Alajuela; county: Sector Rincon Rain Forest; locality: Area de Conservacion Guanacaste; verbatimLocality: Palomo; verbatimElevation: 96; verbatimLatitude: 10.9619; verbatimLongitude: -85.2804; verbatimCoordinateSystem: Decimal; **Identification:** identifiedBy: AJ Fleming; dateIdentified: 2016; **Event:** samplingProtocol: Reared from the larva of the notodontid moth, Disphragis perses; verbatimEventDate: 10-Jan-2013; **Record Level:** language: en; institutionCode: CNC; collectionCode: Insects; basisOfRecord: Pinned Specimen
- **Type status:** Paratype. **Occurrence:** occurrenceDetails: http://janzen.sas.upenn.edu; catalogNumber: 12-SRNP-79034; recordedBy: D.H. Janzen, W. Hallwachs & Keiner Aragon; individualID: 12-SRNP-79034; individualCount: 3; sex: 1M, F; lifeStage: adult; preparations: pinned; otherCatalogNumbers: 12-SRNP-79034, BOLD:ACJ3769, ACGBA3081-13; **Taxon:** scientificName: Calolydella felipechavarriai; phylum: Arthropoda; class: Insecta; order: Diptera; family: Tachinidae; genus: Calolydella; specificEpithet: felipechavarriai; scientificNameAuthorship: Fleming & Wood, 2016; **Location:** continent: Central America; country: Costa Rica; countryCode: CR; stateProvince: Alajuela; county: Sector Rincon Rain Forest; locality: Area de Conservacion Guanacaste; verbatimLocality: Palomo; verbatimElevation: 96; verbatimLatitude: 10.9619; verbatimLongitude: -85.2804; verbatimCoordinateSystem: Decimal; **Identification:** identifiedBy: AJ Fleming; dateIdentified: 2016; **Event:** samplingProtocol: Reared from the larva of the notodontid moth, Disphragis perses; verbatimEventDate: 10-Jan-2013; **Record Level:** language: en; institutionCode: CNC; collectionCode: Insects; basisOfRecord: Pinned Specimen

#### Description

**Male** (Fig. 17a, b, c). Length: 4–6mm. **Head** (Fig. 17b): frontal setae extending beyond base of postpedicel; fronto-orbital plate gold, almost bare, with a few small setulae interspersed among frontal setae; parafacial silver throughout. **Thorax** (Fig. 17a, c): gold on dorsal surface, silver laterally (>50% coverage); with four regular thoracic vittae; postpronotum with three setae; 3:3 acrostichal setae; 2:3 dorsocentral setae; 2:3 intra-alar setae; 2:3 supra-alar setae 2:3; three katepisternal setae; anatergite with two hair-like setae; scutellar discal setae slightly closer together than subapical scutellar setae. Wing vein R_4+5_ with 6–7 small setulae dorsally, extending to crossvein R-M. **Abdomen** (Fig. 17a): black in ground color, with uninterrupted transverse marginal pollinose bands; abdominal pollinosity gold dorsally, silver ventrally; base of ST1+2 black lateroventrally; T3 with one pair of median marginal setae and one pair of discal setae; T4 with one pair of discal setae. **Terminalia** (Fig. 18): sternite 5 (Fig. 18c) with two small lobes and a wide U-shaped median cleft, 0.31X the length of the sternite from lobe to apex; inner margin covered by dense pollinosity, appearing darker than surrounding cuticle; upper half of sternite with many stout, outwardly-pointing setae. Cerci (Fig. 18b), in dorsal view, separated by a narrow gap; each cercus long and rounded, and not significantly tapering from base to tip; cercus straight when viewed laterally, with a slight downward hook at its tip, setose almost along its entire length. Surstylus (Fig. 18a) stout and digitiform, bent midway, with a strong downward bias; apical half with short setae; tip of surstylus not lobed or angled inwards when viewed dorsally; subequal in length to cercus.

**Female** (Fig. 17d, e, f). Length: 4–5mm. Fronto-orbital plate 1.36X as wide as in male.

#### Diagnosis

*Calolydella felipechavarriai* can be distinguished from all other species of *Calolydella* by the following combination of traits: fronto-orbital plate almost bare, anatergite with at most two hair-like setae, and T4 with one pair of discal setae.

#### Etymology

The specific epithet is in honor of Luis Felipe Chavarria Diaz of ACG, Guanacaste, Costa Rica, in recognition of his high quality accounting, photography and administrative support of the Guanacaste Dry Forest Conservation Fund (www.gdfcf.org) and of the ACG parataxonomists who collected and reared the caterpillars from which the new species of ACG *Calolydella* described here emerged.

#### Distribution

Costa Rica, ACG, Alajuela Province, Palomo, 96m.

#### Ecology

*Calolydella felipechavarriai* has been reared twice from *Bardaxima perses* Druce, 1900 (Lepidoptera: Notodontidae), in rain forest.

### Calolydella fredriksjobergi

Fleming & Wood sp. n.

urn:lsid:zoobank.org:act:9E389552-FFEF-43B1-B371-081170A9EB6A

#### Materials

- **Type status:** Holotype. **Occurrence:** occurrenceDetails: http://janzen.sas.upenn.edu; catalogNumber: DHJPAR0011711; recordedBy: D.H. Janzen, W. Hallwachs & Asitente de Diana; individualID: DHJPAR0011711; individualCount: 1; sex: M; lifeStage: adult; preparations: pinned; otherCatalogNumbers: 03-SRNP-21135, BOLD:ABY7242, ASTAS437-06; **Taxon:** scientificName: Calolydella fredriksjobergi; phylum: Arthropoda; class: Insecta; order: Diptera; family: Tachinidae; genus: Calolydella; specificEpithet: fredriksjobergi; scientificNameAuthorship: Fleming & Wood, 2016; **Location:** continent: Central America; country: Costa Rica; countryCode: CR; stateProvince: Guanacaste; county: Sector Pitilla; locality: Area de Conservacion Guanacaste; verbatimLocality: Estacion Pitilla; verbatimElevation: 675; verbatimLatitude: 10.9893; verbatimLongitude: -85.4258; verbatimCoordinateSystem: Decimal; **Identification:** identifiedBy: AJ Fleming; dateIdentified: 2016; **Event:** samplingProtocol: Reared from the larvae of the erebid moth, Dysschema jansonis; verbatimEventDate: 28-Sep-2003; **Record Level:** language: en; institutionCode: CNC; collectionCode: Insects; basisOfRecord: Pinned Specimen
- **Type status:** Paratype. **Occurrence:** occurrenceDetails: http://janzen.sas.upenn.edu; catalogNumber: DHJPAR0054013; recordedBy: D.H. Janzen, W. Hallwachs & Cirilo Umana; individualID: DHJPAR0054013; individualCount: 1; sex: F; lifeStage: adult; preparations: pinned; otherCatalogNumbers: 13-SRNP-77901, BOLD:ABY7242, ASHYD3181-14; **Taxon:** scientificName: Calolydella fredriksjobergi; phylum: Arthropoda; class: Insecta; order: Diptera; family: Tachinidae; genus: Calolydella; specificEpithet: fredriksjobergi; scientificNameAuthorship: Fleming & Wood, 2016; **Location:** continent: Central America; country: Costa Rica; countryCode: CR; stateProvince: Alajuela; county: Sector Rincon Rain Forest; locality: Area de Conservacion Guanacaste; verbatimLocality: Finca Esmeralda; verbatimElevation: 123; verbatimLatitude: 10.9355; verbatimLongitude: -85.2531; verbatimCoordinateSystem: Decimal; **Identification:** identifiedBy: AJ Fleming; dateIdentified: 2016; **Event:** samplingProtocol: Reared from the larvae of the nymphalid butterfly, Danaus plexippus; verbatimEventDate: 21-Nov-2013; **Record Level:** language: en; institutionCode: CNC; collectionCode: Insects; basisOfRecord: Pinned Specimen
- **Type status:** Paratype. **Occurrence:** occurrenceDetails: http://janzen.sas.upenn.edu; catalogNumber: DHJPAR0017784; recordedBy: D.H. Janzen, W. Hallwachs & gusaneros; individualID: DHJPAR0017784; individualCount: 1; sex: F; lifeStage: adult; preparations: pinned; otherCatalogNumbers: 99-SRNP-8296, BOLD:ABY7242, ASTAR495-07; **Taxon:** scientificName: Calolydella fredriksjobergi; phylum: Arthropoda; class: Insecta; order: Diptera; family: Tachinidae; genus: Calolydella; specificEpithet: fredriksjobergi; scientificNameAuthorship: Fleming & Wood, 2016; **Location:** continent: Central America; country: Costa Rica; countryCode: CR; stateProvince: Guanacaste; county: Sector Horizontes; locality: Area de Conservacion Guanacaste; verbatimLocality: Sitio La Dama; verbatimElevation: 105; verbatimLatitude: 10.7863; verbatimLongitude: -85.5583; verbatimCoordinateSystem: Decimal; **Identification:** identifiedBy: AJ Fleming; dateIdentified: 2016; **Event:** samplingProtocol: Reared from the larvae of the nymphalid butterfly, Anaea aidea; verbatimEventDate: 17-Jun-1999; **Record Level:** language: en; institutionCode: CNC; collectionCode: Insects; basisOfRecord: Pinned Specimen
- **Type status:** Paratype. **Occurrence:** occurrenceDetails: http://janzen.sas.upenn.edu; catalogNumber: DHJPAR0050643; recordedBy: D.H. Janzen, W. Hallwachs & Cirilo Umana; individualID: DHJPAR0050643; individualCount: 1; sex: M; lifeStage: adult; preparations: pinned; otherCatalogNumbers: 12-SRNP-77815, BOLD:ABY7242, ACGBA3235-13; **Taxon:** scientificName: Calolydella fredriksjobergi; phylum: Arthropoda; class: Insecta; order: Diptera; family: Tachinidae; genus: Calolydella; specificEpithet: fredriksjobergi; scientificNameAuthorship: Fleming & Wood, 2016; **Location:** continent: Central America; country: Costa Rica; countryCode: CR; stateProvince: Alajuela; county: Sector Rincon Rain Forest; locality: Area de Conservacion Guanacaste; verbatimLocality: Finca Esmeralda; verbatimElevation: 123; verbatimLatitude: 10.9355; verbatimLongitude: -85.2531; verbatimCoordinateSystem: Decimal; **Identification:** identifiedBy: AJ Fleming; dateIdentified: 2016; **Event:** samplingProtocol: Reared from the larvae of the erebid moth, Melese Espinoza01; verbatimEventDate: 16-Dec-2012; **Record Level:** language: en; institutionCode: CNC; collectionCode: Insects; basisOfRecord: Pinned Specimen
- **Type status:** Paratype. **Occurrence:** occurrenceDetails: http://janzen.sas.upenn.edu; catalogNumber: DHJPAR0035673; recordedBy: D.H. Janzen, W. Hallwachs & Mariano Pereira; individualID: DHJPAR0035673; individualCount: 1; sex: M; lifeStage: adult; preparations: pinned; otherCatalogNumbers: 09-SRNP-56992, BOLD:ABY7242, ASHYD1054-09; **Taxon:** scientificName: Calolydella fredriksjobergi; phylum: Arthropoda; class: Insecta; order: Diptera; family: Tachinidae; genus: Calolydella; specificEpithet: fredriksjobergi; scientificNameAuthorship: Fleming & Wood, 2016; **Location:** continent: Central America; country: Costa Rica; countryCode: CR; stateProvince: Guanacaste; county: Sector Mundo Nuevo; locality: Area de Conservacion Guanacaste; verbatimLocality: Camino Pozo Tres; verbatimElevation: 733; verbatimLatitude: 10.7708; verbatimLongitude: -85.3742; verbatimCoordinateSystem: Decimal; **Identification:** identifiedBy: AJ Fleming; dateIdentified: 2016; **Event:** samplingProtocol: Reared from the larvae of the nymphalid butterfly, Chlosyne rosita; verbatimEventDate: 09-Aug-2009; **Record Level:** language: en; institutionCode: CNC; collectionCode: Insects; basisOfRecord: Pinned Specimen
- **Type status:** Paratype. **Occurrence:** occurrenceDetails: http://janzen.sas.upenn.edu; catalogNumber: DHJPAR0029600; recordedBy: D.H. Janzen, W. Hallwachs & Dunia Garcia; individualID: DHJPAR0029600; individualCount: 1; sex: F; lifeStage: adult; preparations: pinned; otherCatalogNumbers: 08-SRNP-36696, BOLD:ABY7242, ASHYM1021-09; **Taxon:** scientificName: Calolydella fredriksjobergi; phylum: Arthropoda; class: Insecta; order: Diptera; family: Tachinidae; genus: Calolydella; specificEpithet: fredriksjobergi; scientificNameAuthorship: Fleming & Wood, 2016; **Location:** continent: Central America; country: Costa Rica; countryCode: CR; stateProvince: Guanacaste; county: Sector Cacao; locality: Area de Conservacion Guanacaste; verbatimLocality: Sendero Ponderosa; verbatimElevation: 1060; verbatimLatitude: 10.9146; verbatimLongitude: -85.4626; verbatimCoordinateSystem: Decimal; **Identification:** identifiedBy: AJ Fleming; dateIdentified: 2016; **Event:** samplingProtocol: Reared from the larvae of the crambid moth, Cliniodes opalalis; verbatimEventDate: 02-Oct-2008; **Record Level:** language: en; institutionCode: CNC; collectionCode: Insects; basisOfRecord: Pinned Specimen

#### Description

**Male** (Fig. 19a, b, c). Length: 7–9mm. **Head** (Fig. 19b): frontal setae extending to base of postpedicel; fronto-orbital plate gold, with a single row of fine setulae outside of frontal setae; parafacial silver throughout. **Thorax** (Fig. 19a, c): gold on both dorsal and lateral surfaces; outermost two thoracic vittae, twice as wide as innermost two; postpronotum with three setae; 3:3 acrostichal setae; 3:3 dorsocentral setae; 2:3 intra-alar setae; 2:3 supra-alar setae; three katepisternal setae; anatergite with three or more hair-like setae, often in a small tuft; scutellar discal setae situated as wide apart as subapical scutellar setae. Wing vein R4+5 with 6–7 setulae dorsally, reaching from base almost to crossvein R-M. **Abdomen** (Fig. 19a): ground color dark brown-orange, with uninterrupted transverse marginal pollinose bands; abdominal pollinosity gold dorsally, silver ventrally, with an orange spot lateroventrally at base of ST1+2; T3 with one pair of median marginal setae and two pairs of discal setae; T4 with a row of marginal setae and one pair of discal setae. **Terminalia** (Fig. 20): sternite 5 (Fig. 20c) with two small lobes and a wide U-shaped median cleft, 0.47X the length of the sternite from lobe to apex; inner margin covered by dense pollinosity, appearing darker than surrounding cuticle; entire lobe of sternite with short setae, of varying lengths. Cerci (Fig. 20b), in dorsal view, separated by a narrow gap not widened at apex; each cercus very slightly tapered from its already narrow base; cercus long, slender and with an upward bend when viewed laterally; setose along its entire length. Surstylus (Fig. 20a) slender and digitiform with a strong divot at tip, giving it an overall bent look; appearing 0.2X shorter than cercus when viewed laterally; with short setae along entire length; tip of surstylus slightly curved inwards when viewed dorsally.

**Female** (Fig. 19d, e, f). Length: 5–6mm. Fronto-orbital plate 2.2X wider than in male; thoracic vittae 0.5X as wide as in male.

#### Diagnosis

*Calolydella fredriksjobergi* can be distinguished from all other species of *Calolydella* by the following combination of characters: parafacial mostly gold, anatergite with three or more hair-like setae arranged in a small tuft, two pairs of discal setae on T3 and one pair of discal setae on T4.

#### Etymology

The specific epithet is in honor of Fredrik Sjoberg of Runmaro Island, Sweden, in recognition of his wonderful book "The Fly Trap" (Sjöberg 2014, translation by Thomas Teal); a book that explains much about Malaise traps, which have captured so many ACG Tachinidae.

#### Distribution

Costa Rica, ACG, Alajuela and Guanacaste provinces, 105–1060m.

#### Ecology

*Calolydella fredriksjobergi* has been reared six times from six different species belonging to various families of Lepidoptera (Nymphalidae; Erebidae; and Crambidae), in rain forest, cloud forest, and dry forest ecosystems.

### Calolydella inflatipalpis

Fleming & Wood sp. n.

urn:lsid:zoobank.org:act:BB67E463-DE23-474A-9900-9EC0193FC403

#### Materials

- **Type status:** Holotype. **Occurrence:** occurrenceDetails: http://janzen.sas.upenn.edu; catalogNumber: DHJPAR0016181; recordedBy: D.H. Janzen, W. Hallwachs & Jose Cortez; individualID: DHJPAR0016181; individualCount: 1; sex: M; lifeStage: adult; preparations: pinned; otherCatalogNumbers: 06-SRNP-58499, BOLD:ABX6062, ASTAP210-06; **Taxon:** scientificName: Calolydella inflatipalpis; phylum: Arthropoda; class: Insecta; order: Diptera; family: Tachinidae; genus: Calolydella; specificEpithet: inflatipalpis; scientificNameAuthorship: Fleming & Wood, 2016; **Location:** continent: Central America; country: Costa Rica; countryCode: CR; stateProvince: Guanacaste; county: Sector Mundo Nuevo; locality: Area de Conservacion Guanacaste; verbatimLocality: Vado Licania; verbatimElevation: 470; verbatimLatitude: 10.7722; verbatimLongitude: -85.4122; verbatimCoordinateSystem: Decimal; **Identification:** identifiedBy: AJ Fleming; dateIdentified: 2016; **Event:** samplingProtocol: Reared from the larva of the nymphalid butterfly, Dione juno; verbatimEventDate: 10-Oct-2006; **Record Level:** language: en; institutionCode: CNC; collectionCode: Insects; basisOfRecord: Pinned Specimen
- **Type status:** Paratype. **Occurrence:** occurrenceDetails: http://janzen.sas.upenn.edu; catalogNumber: DHJPAR0017796; recordedBy: D.H. Janzen, W. Hallwachs & Mariano Pereira; individualID: DHJPAR0017796; individualCount: 1; sex: F; lifeStage: adult; preparations: pinned; otherCatalogNumbers: 02-SRNP-9198, BOLD:ABX6062, ASTAR507-07; **Taxon:** scientificName: Calolydella inflatipalpis; phylum: Arthropoda; class: Insecta; order: Diptera; family: Tachinidae; genus: Calolydella; specificEpithet: inflatipalpis; scientificNameAuthorship: Fleming & Wood, 2016; **Location:** continent: Central America; country: Costa Rica; countryCode: CR; stateProvince: Guanacaste; county: Sector Cacao; locality: Area de Conservacion Guanacaste; verbatimLocality: Estacion Cacao; verbatimElevation: 1150; verbatimLatitude: 10.9269; verbatimLongitude: -85.4682; verbatimCoordinateSystem: Decimal; **Identification:** identifiedBy: AJ Fleming; dateIdentified: 2016; **Event:** samplingProtocol: Reared from the larva of the nymphalid butterfly, Dione juno; verbatimEventDate: 08-Jun-2002; **Record Level:** language: en; institutionCode: CNC; collectionCode: Insects; basisOfRecord: Pinned Specimen
- **Type status:** Paratype. **Occurrence:** occurrenceDetails: http://janzen.sas.upenn.edu; catalogNumber: DHJPAR0017806; recordedBy: D.H. Janzen, W. Hallwachs & Roster Moraga; individualID: DHJPAR0017806; individualCount: 1; sex: M; lifeStage: adult; preparations: pinned; otherCatalogNumbers: 00-SRNP-3991.32, BOLD:ABX6062, ASTAR517-07; **Taxon:** scientificName: Calolydella inflatipalpis; phylum: Arthropoda; class: Insecta; order: Diptera; family: Tachinidae; genus: Calolydella; specificEpithet: inflatipalpis; scientificNameAuthorship: Fleming & Wood, 2016; **Location:** continent: Central America; country: Costa Rica; countryCode: CR; stateProvince: Guanacaste; county: Sector El Hacha; locality: Area de Conservacion Guanacaste; verbatimLocality: Casa Uno; verbatimElevation: 280; verbatimLatitude: 11.0056; verbatimLongitude: -85.5743; verbatimCoordinateSystem: Decimal; **Identification:** identifiedBy: AJ Fleming; dateIdentified: 2016; **Event:** samplingProtocol: Reared from the larva of the nymphalid butterfly, Dione juno; verbatimEventDate: 21-Sep-2000; **Record Level:** language: en; institutionCode: CNC; collectionCode: Insects; basisOfRecord: Pinned Specimen
- **Type status:** Paratype. **Occurrence:** occurrenceDetails: http://janzen.sas.upenn.edu; catalogNumber: DHJPAR0016179; recordedBy: D.H. Janzen, W. Hallwachs & Jose Cortez; individualID: DHJPAR0016179; individualCount: 1; sex: M; lifeStage: adult; preparations: pinned; otherCatalogNumbers: 06-SRNP-58475, BOLD:ABX6062, ASTAP208-06; **Taxon:** scientificName: Calolydella inflatipalpis; phylum: Arthropoda; class: Insecta; order: Diptera; family: Tachinidae; genus: Calolydella; specificEpithet: inflatipalpis; scientificNameAuthorship: Fleming & Wood, 2016; **Location:** continent: Central America; country: Costa Rica; countryCode: CR; stateProvince: Guanacaste; county: Sector Mundo Nuevo; locality: Area de Conservacion Guanacaste; verbatimLocality: Vado Licania; verbatimElevation: 470; verbatimLatitude: 10.7722; verbatimLongitude: -85.4122; verbatimCoordinateSystem: Decimal; **Identification:** identifiedBy: AJ Fleming; dateIdentified: 2016; **Event:** samplingProtocol: Reared from the larva of the nymphalid butterfly, Dione juno; verbatimEventDate: 10-Oct-2006; **Record Level:** language: en; institutionCode: CNC; collectionCode: Insects; basisOfRecord: Pinned Specimen
- **Type status:** Paratype. **Occurrence:** occurrenceDetails: http://janzen.sas.upenn.edu; catalogNumber: DHJPAR0016169; recordedBy: D.H. Janzen, W. Hallwachs & Jose Cortez; individualID: DHJPAR0016169; individualCount: 1; sex: F; lifeStage: adult; preparations: pinned; otherCatalogNumbers: 06-SRNP-58472, BOLD:ABX6062, ASTAP198-06; **Taxon:** scientificName: Calolydella inflatipalpis; phylum: Arthropoda; class: Insecta; order: Diptera; family: Tachinidae; genus: Calolydella; specificEpithet: inflatipalpis; scientificNameAuthorship: Fleming & Wood, 2016; **Location:** continent: Central America; country: Costa Rica; countryCode: CR; stateProvince: Guanacaste; county: Sector Mundo Nuevo; locality: Area de Conservacion Guanacaste; verbatimLocality: Vado Licania; verbatimElevation: 470; verbatimLatitude: 10.7722; verbatimLongitude: -85.4122; verbatimCoordinateSystem: Decimal; **Identification:** identifiedBy: AJ Fleming; dateIdentified: 2016; **Event:** samplingProtocol: Reared from the larva of the nymphalid butterfly, Dione juno; verbatimEventDate: 08-Oct-2006; **Record Level:** language: en; institutionCode: CNC; collectionCode: Insects; basisOfRecord: Pinned Specimen
- **Type status:** Paratype. **Occurrence:** occurrenceDetails: http://janzen.sas.upenn.edu; catalogNumber: DHJPAR0017793; recordedBy: D.H. Janzen, W. Hallwachs & Mariano Pereira; individualID: DHJPAR0017793; individualCount: 1; sex: F; lifeStage: adult; preparations: pinned; otherCatalogNumbers: 02-SRNP-9197, BOLD:ABX6062, ASTAR504-07; **Taxon:** scientificName: Calolydella inflatipalpis; phylum: Arthropoda; class: Insecta; order: Diptera; family: Tachinidae; genus: Calolydella; specificEpithet: inflatipalpis; scientificNameAuthorship: Fleming & Wood, 2016; **Location:** continent: Central America; country: Costa Rica; countryCode: CR; stateProvince: Guanacaste; county: Sector Cacao; locality: Area de Conservacion Guanacaste; verbatimLocality: Estacion Cacao; verbatimElevation: 1150; verbatimLatitude: 10.9269; verbatimLongitude: -85.4682; verbatimCoordinateSystem: Decimal; **Identification:** identifiedBy: AJ Fleming; dateIdentified: 2016; **Event:** samplingProtocol: Reared from the larva of the nymphalid butterfly, Dione juno; verbatimEventDate: 08-Jun-2002; **Record Level:** language: en; institutionCode: CNC; collectionCode: Insects; basisOfRecord: Pinned Specimen
- **Type status:** Paratype. **Occurrence:** occurrenceDetails: http://janzen.sas.upenn.edu; catalogNumber: DHJPAR0016170; recordedBy: D.H. Janzen, W. Hallwachs & Jose Cortez; individualID: DHJPAR0016170; individualCount: 1; sex: M; lifeStage: adult; preparations: pinned; otherCatalogNumbers: 06-SRNP-58481, BOLD:ABX6062, ASTAP199-06; **Taxon:** scientificName: Calolydella inflatipalpis; phylum: Arthropoda; class: Insecta; order: Diptera; family: Tachinidae; genus: Calolydella; specificEpithet: inflatipalpis; scientificNameAuthorship: Fleming & Wood, 2016; **Location:** continent: Central America; country: Costa Rica; countryCode: CR; stateProvince: Guanacaste; county: Sector Mundo Nuevo; locality: Area de Conservacion Guanacaste; verbatimLocality: Vado Licania; verbatimElevation: 470; verbatimLatitude: 10.7722; verbatimLongitude: -85.4122; verbatimCoordinateSystem: Decimal; **Identification:** identifiedBy: AJ Fleming; dateIdentified: 2016; **Event:** samplingProtocol: Reared from the larva of the nymphalid butterfly, Dione juno; verbatimEventDate: 07-Oct-2006; **Record Level:** language: en; institutionCode: CNC; collectionCode: Insects; basisOfRecord: Pinned Specimen
- **Type status:** Paratype. **Occurrence:** occurrenceDetails: http://janzen.sas.upenn.edu; catalogNumber: DHJPAR0017800; recordedBy: D.H. Janzen, W. Hallwachs & Roster Moraga; individualID: DHJPAR0017800; individualCount: 1; sex: M; lifeStage: adult; preparations: pinned; otherCatalogNumbers: 00-SRNP-3991.22, BOLD:ABX6062, ASTAR511-07; **Taxon:** scientificName: Calolydella inflatipalpis; phylum: Arthropoda; class: Insecta; order: Diptera; family: Tachinidae; genus: Calolydella; specificEpithet: inflatipalpis; scientificNameAuthorship: Fleming & Wood, 2016; **Location:** continent: Central America; country: Costa Rica; countryCode: CR; stateProvince: Guanacaste; county: Sector El Hacha; locality: Area de Conservacion Guanacaste; verbatimLocality: Casa Uno; verbatimElevation: 280; verbatimLatitude: 11.0056; verbatimLongitude: -85.5743; verbatimCoordinateSystem: Decimal; **Identification:** identifiedBy: AJ Fleming; dateIdentified: 2016; **Event:** samplingProtocol: Reared from the larva of the nymphalid butterfly, Dione juno; verbatimEventDate: 23-Sep-2000; **Record Level:** language: en; institutionCode: CNC; collectionCode: Insects; basisOfRecord: Pinned Specimen
- **Type status:** Paratype. **Occurrence:** occurrenceDetails: http://janzen.sas.upenn.edu; catalogNumber: DHJPAR0016197; recordedBy: D.H. Janzen, W. Hallwachs & Roster Moraga; individualID: DHJPAR0016197; individualCount: 1; sex: M; lifeStage: adult; preparations: pinned; otherCatalogNumbers: 06-SRNP-22670, BOLD:ABX6062, ASTAP226-06; **Taxon:** scientificName: Calolydella inflatipalpis; phylum: Arthropoda; class: Insecta; order: Diptera; family: Tachinidae; genus: Calolydella; specificEpithet: inflatipalpis; scientificNameAuthorship: Fleming & Wood, 2016; **Location:** continent: Central America; country: Costa Rica; countryCode: CR; stateProvince: Guanacaste; county: Sector Del Oro; locality: Area de Conservacion Guanacaste; verbatimLocality: San Antonio; verbatimElevation: 335; verbatimLatitude: 11.0353; verbatimLongitude: -85.4453; verbatimCoordinateSystem: Decimal; **Identification:** identifiedBy: AJ Fleming; dateIdentified: 2016; **Event:** samplingProtocol: Reared from the larva of the nymphalid butterfly, Dione juno; verbatimEventDate: 28-Sep-2006; **Record Level:** language: en; institutionCode: CNC; collectionCode: Insects; basisOfRecord: Pinned Specimen
- **Type status:** Paratype. **Occurrence:** occurrenceDetails: http://janzen.sas.upenn.edu; catalogNumber: DHJPAR0017801; recordedBy: D.H. Janzen, W. Hallwachs & Dunia Garcia; individualID: DHJPAR0017801; individualCount: 1; sex: M; lifeStage: adult; preparations: pinned; otherCatalogNumbers: 03-SRNP-25826, BOLD:ABX6062, ASTAR512-07; **Taxon:** scientificName: Calolydella inflatipalpis; phylum: Arthropoda; class: Insecta; order: Diptera; family: Tachinidae; genus: Calolydella; specificEpithet: inflatipalpis; scientificNameAuthorship: Fleming & Wood, 2016; **Location:** continent: Central America; country: Costa Rica; countryCode: CR; stateProvince: Guanacaste; county: Sector Santa Rosa; locality: Area de Conservacion Guanacaste; verbatimLocality: Bosque Humedo; verbatimElevation: 290; verbatimLatitude: 10.8514; verbatimLongitude: -85.608; verbatimCoordinateSystem: Decimal; **Identification:** identifiedBy: AJ Fleming; dateIdentified: 2016; **Event:** samplingProtocol: Reared from the larva of the nymphalid butterfly, Dione juno; verbatimEventDate: 25-Sep-2003; **Record Level:** language: en; institutionCode: CNC; collectionCode: Insects; basisOfRecord: Pinned Specimen
- **Type status:** Paratype. **Occurrence:** occurrenceDetails: http://janzen.sas.upenn.edu; catalogNumber: DHJPAR0017802; recordedBy: D.H. Janzen, W. Hallwachs & gusaneros; individualID: DHJPAR0017802; individualCount: 1; sex: M; lifeStage: adult; preparations: pinned; otherCatalogNumbers: 95-SRNP-8266, BOLD:ABX6062, ASTAR513-07; **Taxon:** scientificName: Calolydella inflatipalpis; phylum: Arthropoda; class: Insecta; order: Diptera; family: Tachinidae; genus: Calolydella; specificEpithet: inflatipalpis; scientificNameAuthorship: Fleming & Wood, 2016; **Location:** continent: Central America; country: Costa Rica; countryCode: CR; stateProvince: Guanacaste; county: Sector Santa Rosa; locality: Area de Conservacion Guanacaste; verbatimLocality: Bosque San Emilio; verbatimElevation: 300; verbatimLatitude: 10.8439; verbatimLongitude: -85.6138; verbatimCoordinateSystem: Decimal; **Identification:** identifiedBy: AJ Fleming; dateIdentified: 2016; **Event:** samplingProtocol: Reared from the larva of the nymphalid butterfly, Dione juno; verbatimEventDate: 09-Feb-1995; **Record Level:** language: en; institutionCode: CNC; collectionCode: Insects; basisOfRecord: Pinned Specimen
- **Type status:** Paratype. **Occurrence:** occurrenceDetails: http://janzen.sas.upenn.edu; catalogNumber: DHJPAR0017798; recordedBy: D.H. Janzen, W. Hallwachs & Roster Moraga; individualID: DHJPAR0017798; individualCount: 1; sex: M; lifeStage: adult; preparations: pinned; otherCatalogNumbers: 00-SRNP-3828, BOLD:ABX6062, ASTAR509-07; **Taxon:** scientificName: Calolydella inflatipalpis; phylum: Arthropoda; class: Insecta; order: Diptera; family: Tachinidae; genus: Calolydella; specificEpithet: inflatipalpis; scientificNameAuthorship: Fleming & Wood, 2016; **Location:** continent: Central America; country: Costa Rica; countryCode: CR; stateProvince: Guanacaste; county: Sector El Hacha; locality: Area de Conservacion Guanacaste; verbatimLocality: Casa Uno; verbatimElevation: 280; verbatimLatitude: 11.0056; verbatimLongitude: -85.5743; verbatimCoordinateSystem: Decimal; **Identification:** identifiedBy: AJ Fleming; dateIdentified: 2016; **Event:** samplingProtocol: Reared from the larva of the nymphalid butterfly, Dione juno; verbatimEventDate: 06-Sep-2000; **Record Level:** language: en; institutionCode: CNC; collectionCode: Insects; basisOfRecord: Pinned Specimen
- **Type status:** Paratype. **Occurrence:** occurrenceDetails: http://janzen.sas.upenn.edu; catalogNumber: DHJPAR0017804; recordedBy: D.H. Janzen, W. Hallwachs & Roster Moraga; individualID: DHJPAR0017804; individualCount: 1; sex: M; lifeStage: adult; preparations: pinned; otherCatalogNumbers: 00-SRNP-3991.03, BOLD:ABX6062, ASTAR515-07; **Taxon:** scientificName: Calolydella inflatipalpis; phylum: Arthropoda; class: Insecta; order: Diptera; family: Tachinidae; genus: Calolydella; specificEpithet: inflatipalpis; scientificNameAuthorship: Fleming & Wood, 2016; **Location:** continent: Central America; country: Costa Rica; countryCode: CR; stateProvince: Guanacaste; county: Sector El Hacha; locality: Area de Conservacion Guanacaste; verbatimLocality: Casa Uno; verbatimElevation: 280; verbatimLatitude: 11.0056; verbatimLongitude: -85.5743; verbatimCoordinateSystem: Decimal; **Identification:** identifiedBy: AJ Fleming; dateIdentified: 2016; **Event:** samplingProtocol: Reared from the larva of the nymphalid butterfly, Dione juno; verbatimEventDate: 20-Sep-2000; **Record Level:** language: en; institutionCode: CNC; collectionCode: Insects; basisOfRecord: Pinned Specimen
- **Type status:** Paratype. **Occurrence:** occurrenceDetails: http://janzen.sas.upenn.edu; catalogNumber: DHJPAR0016098; recordedBy: D.H. Janzen, W. Hallwachs & Jose Cortez; individualID: DHJPAR0016098; individualCount: 1; sex: F; lifeStage: adult; preparations: pinned; otherCatalogNumbers: 06-SRNP-58469, BOLD:ABX6062, ASTAP127-06; **Taxon:** scientificName: Calolydella inflatipalpis; phylum: Arthropoda; class: Insecta; order: Diptera; family: Tachinidae; genus: Calolydella; specificEpithet: inflatipalpis; scientificNameAuthorship: Fleming & Wood, 2016; **Location:** continent: Central America; country: Costa Rica; countryCode: CR; stateProvince: Guanacaste; county: Sector Mundo Nuevo; locality: Area de Conservacion Guanacaste; verbatimLocality: Vado Licania; verbatimElevation: 470; verbatimLatitude: 10.7722; verbatimLongitude: -85.4122; verbatimCoordinateSystem: Decimal; **Identification:** identifiedBy: AJ Fleming; dateIdentified: 2016; **Event:** samplingProtocol: Reared from the larva of the nymphalid butterfly, Dione juno; verbatimEventDate: 03-Oct-2006; **Record Level:** language: en; institutionCode: CNC; collectionCode: Insects; basisOfRecord: Pinned Specimen
- **Type status:** Paratype. **Occurrence:** occurrenceDetails: http://janzen.sas.upenn.edu; catalogNumber: DHJPAR0017803; recordedBy: D.H. Janzen, W. Hallwachs & Roster Moraga; individualID: DHJPAR0017803; individualCount: 1; sex: F; lifeStage: adult; preparations: pinned; otherCatalogNumbers: 00-SRNP-3842, BOLD:ABX6062, ASTAR514-07; **Taxon:** scientificName: Calolydella inflatipalpis; phylum: Arthropoda; class: Insecta; order: Diptera; family: Tachinidae; genus: Calolydella; specificEpithet: inflatipalpis; scientificNameAuthorship: Fleming & Wood, 2016; **Location:** continent: Central America; country: Costa Rica; countryCode: CR; stateProvince: Guanacaste; county: Sector El Hacha; locality: Area de Conservacion Guanacaste; verbatimLocality: Casa Uno; verbatimElevation: 280; verbatimLatitude: 11.0056; verbatimLongitude: -85.5743; verbatimCoordinateSystem: Decimal; **Identification:** identifiedBy: AJ Fleming; dateIdentified: 2016; **Event:** samplingProtocol: Reared from the larva of the nymphalid butterfly, Dione juno; verbatimEventDate: 06-Sep-2000; **Record Level:** language: en; institutionCode: CNC; collectionCode: Insects; basisOfRecord: Pinned Specimen
- **Type status:** Paratype. **Occurrence:** occurrenceDetails: http://janzen.sas.upenn.edu; catalogNumber: DHJPAR0017807; recordedBy: D.H. Janzen, W. Hallwachs & Roster Moraga; individualID: DHJPAR0017807; individualCount: 1; sex: F; lifeStage: adult; preparations: pinned; otherCatalogNumbers: 00-SRNP-3991.04, BOLD:ABX6062, ASTAR518-07; **Taxon:** scientificName: Calolydella inflatipalpis; phylum: Arthropoda; class: Insecta; order: Diptera; family: Tachinidae; genus: Calolydella; specificEpithet: inflatipalpis; scientificNameAuthorship: Fleming & Wood, 2016; **Location:** continent: Central America; country: Costa Rica; countryCode: CR; stateProvince: Guanacaste; county: Sector El Hacha; locality: Area de Conservacion Guanacaste; verbatimLocality: Casa Uno; verbatimElevation: 280; verbatimLatitude: 11.0056; verbatimLongitude: -85.5743; verbatimCoordinateSystem: Decimal; **Identification:** identifiedBy: AJ Fleming; dateIdentified: 2016; **Event:** samplingProtocol: Reared from the larva of the nymphalid butterfly, Dione juno; verbatimEventDate: 19-Sep-2000; **Record Level:** language: en; institutionCode: CNC; collectionCode: Insects; basisOfRecord: Pinned Specimen
- **Type status:** Paratype. **Occurrence:** occurrenceDetails: http://janzen.sas.upenn.edu; catalogNumber: DHJPAR0011704; recordedBy: D.H. Janzen, W. Hallwachs & Manuel Rios; individualID: DHJPAR0011704; individualCount: 1; sex: F; lifeStage: adult; preparations: pinned; otherCatalogNumbers: 05-SRNP-31118, BOLD:ABX6062, ASTAS430-06; **Taxon:** scientificName: Calolydella inflatipalpis; phylum: Arthropoda; class: Insecta; order: Diptera; family: Tachinidae; genus: Calolydella; specificEpithet: inflatipalpis; scientificNameAuthorship: Fleming & Wood, 2016; **Location:** continent: Central America; country: Costa Rica; countryCode: CR; stateProvince: Guanacaste; county: Sector Pitilla; locality: Area de Conservacion Guanacaste; verbatimLocality: Pasmompa; verbatimElevation: 440; verbatimLatitude: 11.0193; verbatimLongitude: -85.41; verbatimCoordinateSystem: Decimal; **Identification:** identifiedBy: AJ Fleming; dateIdentified: 2016; **Event:** samplingProtocol: Reared from the larva of the nymphalid butterfly, Dryadula phaetusa; verbatimEventDate: 13-Apr-2005; **Record Level:** language: en; institutionCode: CNC; collectionCode: Insects; basisOfRecord: Pinned Specimen
- **Type status:** Paratype. **Occurrence:** occurrenceDetails: http://janzen.sas.upenn.edu; catalogNumber: DHJPAR0016168; recordedBy: D.H. Janzen, W. Hallwachs & Jose Cortez; individualID: DHJPAR0016168; individualCount: 1; sex: M; lifeStage: adult; preparations: pinned; otherCatalogNumbers: 06-SRNP-58471, BOLD:ABX6062, ASTAP197-06; **Taxon:** scientificName: Calolydella inflatipalpis; phylum: Arthropoda; class: Insecta; order: Diptera; family: Tachinidae; genus: Calolydella; specificEpithet: inflatipalpis; scientificNameAuthorship: Fleming & Wood, 2016; **Location:** continent: Central America; country: Costa Rica; countryCode: CR; stateProvince: Guanacaste; county: Sector Mundo Nuevo; locality: Area de Conservacion Guanacaste; verbatimLocality: Vado Licania; verbatimElevation: 470; verbatimLatitude: 10.7722; verbatimLongitude: -85.4122; verbatimCoordinateSystem: Decimal; **Identification:** identifiedBy: AJ Fleming; dateIdentified: 2016; **Event:** samplingProtocol: Reared from the larva of the nymphalid butterfly, Dione juno; verbatimEventDate: 07-Oct-2006; **Record Level:** language: en; institutionCode: CNC; collectionCode: Insects; basisOfRecord: Pinned Specimen
- **Type status:** Paratype. **Occurrence:** occurrenceDetails: http://janzen.sas.upenn.edu; catalogNumber: DHJPAR0029990; recordedBy: D.H. Janzen, W. Hallwachs & Elda Araya; individualID: DHJPAR0029990; individualCount: 1; sex: M; lifeStage: adult; preparations: pinned; otherCatalogNumbers: 08-SRNP-5495, BOLD:ABX6062, ASHYB734-09; **Taxon:** scientificName: Calolydella inflatipalpis; phylum: Arthropoda; class: Insecta; order: Diptera; family: Tachinidae; genus: Calolydella; specificEpithet: inflatipalpis; scientificNameAuthorship: Fleming & Wood, 2016; **Location:** continent: Central America; country: Costa Rica; countryCode: CR; stateProvince: Alajuela; county: Buenos Aires; locality: Area de Conservacion Guanacaste; verbatimLocality: Finca Tomate; verbatimElevation: 360; verbatimLatitude: 10.9035; verbatimLongitude: -85.3092; verbatimCoordinateSystem: Decimal; **Identification:** identifiedBy: AJ Fleming; dateIdentified: 2016; **Event:** samplingProtocol: Reared from the larva of the nymphalid butterfly, Dione juno; verbatimEventDate: 02-Nov-2008; **Record Level:** language: en; institutionCode: CNC; collectionCode: Insects; basisOfRecord: Pinned Specimen
- **Type status:** Paratype. **Occurrence:** occurrenceDetails: http://janzen.sas.upenn.edu; catalogNumber: DHJPAR0017797; recordedBy: D.H. Janzen, W. Hallwachs & Roster Moraga; individualID: DHJPAR0017797; individualCount: 1; sex: F; lifeStage: adult; preparations: pinned; otherCatalogNumbers: 00-SRNP-3829, BOLD:ABX6062, ASTAR508-07; **Taxon:** scientificName: Calolydella inflatipalpis; phylum: Arthropoda; class: Insecta; order: Diptera; family: Tachinidae; genus: Calolydella; specificEpithet: inflatipalpis; scientificNameAuthorship: Fleming & Wood, 2016; **Location:** continent: Central America; country: Costa Rica; countryCode: CR; stateProvince: Guanacaste; county: Sector El Hacha; locality: Area de Conservacion Guanacaste; verbatimLocality: Casa Uno; verbatimElevation: 280; verbatimLatitude: 11.0056; verbatimLongitude: -85.5743; verbatimCoordinateSystem: Decimal; **Identification:** identifiedBy: AJ Fleming; dateIdentified: 2016; **Event:** samplingProtocol: Reared from the larva of the nymphalid butterfly, Dione juno; verbatimEventDate: 05-Sep-2000; **Record Level:** language: en; institutionCode: CNC; collectionCode: Insects; basisOfRecord: Pinned Specimen
- **Type status:** Paratype. **Occurrence:** occurrenceDetails: http://janzen.sas.upenn.edu; catalogNumber: DHJPAR0015983; recordedBy: D.H. Janzen, W. Hallwachs & Mariano Pereira; individualID: DHJPAR0015983; individualCount: 1; sex: M; lifeStage: adult; preparations: pinned; otherCatalogNumbers: 06-SRNP-58420, BOLD:ABX6062, ASTAP012-06; **Taxon:** scientificName: Calolydella inflatipalpis; phylum: Arthropoda; class: Insecta; order: Diptera; family: Tachinidae; genus: Calolydella; specificEpithet: inflatipalpis; scientificNameAuthorship: Fleming & Wood, 2016; **Location:** continent: Central America; country: Costa Rica; countryCode: CR; stateProvince: Guanacaste; county: Sector Mundo Nuevo; locality: Area de Conservacion Guanacaste; verbatimLocality: Vado Licania; verbatimElevation: 470; verbatimLatitude: 10.7722; verbatimLongitude: -85.4122; verbatimCoordinateSystem: Decimal; **Identification:** identifiedBy: AJ Fleming; dateIdentified: 2016; **Event:** samplingProtocol: Reared from the larva of the nymphalid butterfly, Dione juno; verbatimEventDate: 06-Oct-2006; **Record Level:** language: en; institutionCode: CNC; collectionCode: Insects; basisOfRecord: Pinned Specimen
- **Type status:** Paratype. **Occurrence:** occurrenceDetails: http://janzen.sas.upenn.edu; catalogNumber: DHJPAR0016196; recordedBy: D.H. Janzen, W. Hallwachs & Elieth Cantillano; individualID: DHJPAR0016196; individualCount: 1; sex: M; lifeStage: adult; preparations: pinned; otherCatalogNumbers: 06-SRNP-22687, BOLD:ABX6062, ASTAP225-06; **Taxon:** scientificName: Calolydella inflatipalpis; phylum: Arthropoda; class: Insecta; order: Diptera; family: Tachinidae; genus: Calolydella; specificEpithet: inflatipalpis; scientificNameAuthorship: Fleming & Wood, 2016; **Location:** continent: Central America; country: Costa Rica; countryCode: CR; stateProvince: Guanacaste; county: Sector Del Oro; locality: Area de Conservacion Guanacaste; verbatimLocality: San Antonio; verbatimElevation: 335; verbatimLatitude: 11.0353; verbatimLongitude: -85.4453; verbatimCoordinateSystem: Decimal; **Identification:** identifiedBy: AJ Fleming; dateIdentified: 2016; **Event:** samplingProtocol: Reared from the larva of the nymphalid butterfly, Dione juno; verbatimEventDate: 25-Sep-2006; **Record Level:** language: en; institutionCode: CNC; collectionCode: Insects; basisOfRecord: Pinned Specimen
- **Type status:** Paratype. **Occurrence:** occurrenceDetails: http://janzen.sas.upenn.edu; catalogNumber: DHJPAR0015972; recordedBy: D.H. Janzen, W. Hallwachs & Jose Cortez; individualID: DHJPAR0015972; individualCount: 1; sex: M; lifeStage: adult; preparations: pinned; otherCatalogNumbers: 06-SRNP-58451, BOLD:ABX6062, ASTAP001-06; **Taxon:** scientificName: Calolydella inflatipalpis; phylum: Arthropoda; class: Insecta; order: Diptera; family: Tachinidae; genus: Calolydella; specificEpithet: inflatipalpis; scientificNameAuthorship: Fleming & Wood, 2016; **Location:** continent: Central America; country: Costa Rica; countryCode: CR; stateProvince: Guanacaste; county: Sector Mundo Nuevo; locality: Area de Conservacion Guanacaste; verbatimLocality: Vado Licania; verbatimElevation: 470; verbatimLatitude: 10.7722; verbatimLongitude: -85.4122; verbatimCoordinateSystem: Decimal; **Identification:** identifiedBy: AJ Fleming; dateIdentified: 2016; **Event:** samplingProtocol: Reared from the larva of the nymphalid butterfly, Dione juno; verbatimEventDate: 09-Oct-2006; **Record Level:** language: en; institutionCode: CNC; collectionCode: Insects; basisOfRecord: Pinned Specimen
- **Type status:** Paratype. **Occurrence:** occurrenceDetails: http://janzen.sas.upenn.edu; catalogNumber: DHJPAR0011691; recordedBy: D.H. Janzen, W. Hallwachs & Manuel Rios; individualID: DHJPAR0011691; individualCount: 1; sex: F; lifeStage: adult; preparations: pinned; otherCatalogNumbers: 04-SRNP-33986, BOLD:ABX6062, ASTAS417-06; **Taxon:** scientificName: Calolydella inflatipalpis; phylum: Arthropoda; class: Insecta; order: Diptera; family: Tachinidae; genus: Calolydella; specificEpithet: inflatipalpis; scientificNameAuthorship: Fleming & Wood, 2016; **Location:** continent: Central America; country: Costa Rica; countryCode: CR; stateProvince: Guanacaste; county: Sector Pitilla; locality: Area de Conservacion Guanacaste; verbatimLocality: Loaiciga; verbatimElevation: 445; verbatimLatitude: 11.0198; verbatimLongitude: -85.4134; verbatimCoordinateSystem: Decimal; **Identification:** identifiedBy: AJ Fleming; dateIdentified: 2016; **Event:** samplingProtocol: Reared from the larva of the nymphalid butterfly, Dryas iulia; verbatimEventDate: 14-Aug-2004; **Record Level:** language: en; institutionCode: CNC; collectionCode: Insects; basisOfRecord: Pinned Specimen
- **Type status:** Paratype. **Occurrence:** occurrenceDetails: http://janzen.sas.upenn.edu; catalogNumber: DHJPAR0015975; recordedBy: D.H. Janzen, W. Hallwachs & Jose Cortez; individualID: DHJPAR0015975; individualCount: 1; sex: F; lifeStage: adult; preparations: pinned; otherCatalogNumbers: 06-SRNP-58470, BOLD:ABX6062, ASTAP004-06; **Taxon:** scientificName: Calolydella inflatipalpis; phylum: Arthropoda; class: Insecta; order: Diptera; family: Tachinidae; genus: Calolydella; specificEpithet: inflatipalpis; scientificNameAuthorship: Fleming & Wood, 2016; **Location:** continent: Central America; country: Costa Rica; countryCode: CR; stateProvince: Guanacaste; county: Sector Mundo Nuevo; locality: Area de Conservacion Guanacaste; verbatimLocality: Vado Licania; verbatimElevation: 470; verbatimLatitude: 10.7722; verbatimLongitude: -85.4122; verbatimCoordinateSystem: Decimal; **Identification:** identifiedBy: AJ Fleming; dateIdentified: 2016; **Event:** samplingProtocol: Reared from the larva of the nymphalid butterfly, Dione juno; verbatimEventDate: 03-Oct-2006; **Record Level:** language: en; institutionCode: CNC; collectionCode: Insects; basisOfRecord: Pinned Specimen
- **Type status:** Paratype. **Occurrence:** occurrenceDetails: http://janzen.sas.upenn.edu; catalogNumber: DHJPAR0024555; recordedBy: D.H. Janzen, W. Hallwachs & Duvalier Briceno; individualID: DHJPAR0024555; individualCount: 1; sex: M; lifeStage: adult; preparations: pinned; otherCatalogNumbers: 08-SRNP-65294, BOLD:ABX6062, ASTAW665-08; **Taxon:** scientificName: Calolydella inflatipalpis; phylum: Arthropoda; class: Insecta; order: Diptera; family: Tachinidae; genus: Calolydella; specificEpithet: inflatipalpis; scientificNameAuthorship: Fleming & Wood, 2016; **Location:** continent: Central America; country: Costa Rica; countryCode: CR; stateProvince: Alajuela; county: Brasilia; locality: Area de Conservacion Guanacaste; verbatimLocality: Piedrona; verbatimElevation: 340; verbatimLatitude: 11.0162; verbatimLongitude: -85.359; verbatimCoordinateSystem: Decimal; **Identification:** identifiedBy: AJ Fleming; dateIdentified: 2016; **Event:** samplingProtocol: Reared from the larva of the nymphalid butterfly, Heliconius charithonia; verbatimEventDate: 15-Apr-2008; **Record Level:** language: en; institutionCode: CNC; collectionCode: Insects; basisOfRecord: Pinned Specimen
- **Type status:** Paratype. **Occurrence:** occurrenceDetails: http://janzen.sas.upenn.edu; catalogNumber: DHJPAR0017805; recordedBy: D.H. Janzen, W. Hallwachs & Roster Moraga; individualID: DHJPAR0017805; individualCount: 1; sex: M; lifeStage: adult; preparations: pinned; otherCatalogNumbers: 00-SRNP-3991.10, BOLD:ABX6062, ASTAR516-07; **Taxon:** scientificName: Calolydella inflatipalpis; phylum: Arthropoda; class: Insecta; order: Diptera; family: Tachinidae; genus: Calolydella; specificEpithet: inflatipalpis; scientificNameAuthorship: Fleming & Wood, 2016; **Location:** continent: Central America; country: Costa Rica; countryCode: CR; stateProvince: Guanacaste; county: Sector El Hacha; locality: Area de Conservacion Guanacaste; verbatimLocality: Casa Uno; verbatimElevation: 280; verbatimLatitude: 11.0056; verbatimLongitude: -85.5743; verbatimCoordinateSystem: Decimal; **Identification:** identifiedBy: AJ Fleming; dateIdentified: 2016; **Event:** samplingProtocol: Reared from the larva of the nymphalid butterfly, Dione juno; verbatimEventDate: 20-Sep-2000; **Record Level:** language: en; institutionCode: CNC; collectionCode: Insects; basisOfRecord: Pinned Specimen
- **Type status:** Paratype. **Occurrence:** occurrenceDetails: http://janzen.sas.upenn.edu; catalogNumber: DHJPAR0016185; recordedBy: D.H. Janzen, W. Hallwachs & Jose Cortez; individualID: DHJPAR0016185; individualCount: 1; sex: F; lifeStage: adult; preparations: pinned; otherCatalogNumbers: 06-SRNP-58496, BOLD:ABX6062, ASTAP214-06; **Taxon:** scientificName: Calolydella inflatipalpis; phylum: Arthropoda; class: Insecta; order: Diptera; family: Tachinidae; genus: Calolydella; specificEpithet: inflatipalpis; scientificNameAuthorship: Fleming & Wood, 2016; **Location:** continent: Central America; country: Costa Rica; countryCode: CR; stateProvince: Guanacaste; county: Sector Mundo Nuevo; locality: Area de Conservacion Guanacaste; verbatimLocality: Vado Licania; verbatimElevation: 470; verbatimLatitude: 10.7722; verbatimLongitude: -85.4122; verbatimCoordinateSystem: Decimal; **Identification:** identifiedBy: AJ Fleming; dateIdentified: 2016; **Event:** samplingProtocol: Reared from the larva of the nymphalid butterfly, Dione juno; verbatimEventDate: 12-Oct-2006; **Record Level:** language: en; institutionCode: CNC; collectionCode: Insects; basisOfRecord: Pinned Specimen
- **Type status:** Paratype. **Occurrence:** occurrenceDetails: http://janzen.sas.upenn.edu; catalogNumber: DHJPAR0017794; recordedBy: D.H. Janzen, W. Hallwachs & Mariano Pereira; individualID: DHJPAR0017794; individualCount: 1; sex: M; lifeStage: adult; preparations: pinned; otherCatalogNumbers: 02-SRNP-9224, BOLD:ABX6062, ASTAR505-07; **Taxon:** scientificName: Calolydella inflatipalpis; phylum: Arthropoda; class: Insecta; order: Diptera; family: Tachinidae; genus: Calolydella; specificEpithet: inflatipalpis; scientificNameAuthorship: Fleming & Wood, 2016; **Location:** continent: Central America; country: Costa Rica; countryCode: CR; stateProvince: Guanacaste; county: Sector Cacao; locality: Area de Conservacion Guanacaste; verbatimLocality: Estacion Cacao; verbatimElevation: 1150; verbatimLatitude: 10.9269; verbatimLongitude: -85.4682; verbatimCoordinateSystem: Decimal; **Identification:** identifiedBy: AJ Fleming; dateIdentified: 2016; **Event:** samplingProtocol: Reared from the larva of the nymphalid butterfly, Dione juno; verbatimEventDate: 05-Jun-2002; **Record Level:** language: en; institutionCode: CNC; collectionCode: Insects; basisOfRecord: Pinned Specimen
- **Type status:** Paratype. **Occurrence:** occurrenceDetails: http://janzen.sas.upenn.edu; catalogNumber: DHJPAR0017795; recordedBy: D.H. Janzen, W. Hallwachs & Mariano Pereira; individualID: DHJPAR0017795; individualCount: 1; sex: M; lifeStage: adult; preparations: pinned; otherCatalogNumbers: 02-SRNP-9199, BOLD:ABX6062, ASTAR506-07; **Taxon:** scientificName: Calolydella inflatipalpis; phylum: Arthropoda; class: Insecta; order: Diptera; family: Tachinidae; genus: Calolydella; specificEpithet: inflatipalpis; scientificNameAuthorship: Fleming & Wood, 2016; **Location:** continent: Central America; country: Costa Rica; countryCode: CR; stateProvince: Guanacaste; county: Sector Cacao; locality: Area de Conservacion Guanacaste; verbatimLocality: Estacion Cacao; verbatimElevation: 1150; verbatimLatitude: 10.9269; verbatimLongitude: -85.4682; verbatimCoordinateSystem: Decimal; **Identification:** identifiedBy: AJ Fleming; dateIdentified: 2016; **Event:** samplingProtocol: Reared from the larva of the nymphalid butterfly, Dione juno; verbatimEventDate: 08-Jun-2002; **Record Level:** language: en; institutionCode: CNC; collectionCode: Insects; basisOfRecord: Pinned Specimen

#### Description

**Male** (Fig. 21a, b, c). Length: 5–9mm. **Head** (Fig. 21b, e): frontal setae extending beyond base of postpedicel; fronto-orbital plate gold, sparsely setulose throughout; parafacial almost entirely gold (75-90% coverage). **Thorax** (Fig. 21a, c, d, f): pollinosity gold on both dorsal and lateral surfaces; or gold on dorsal surface and silver laterally (more than 50% coverage); thorax with outermost two vittae twice as wide as innermost two; postpronotum with three setae; 3:3 acrostichal setae; 3:3 dorsocentral setae; 2:3 intra-alar setae; 2:3 supra-alar setae; three katepisternal setae; anatergite with three or more hair-like setae, often in a small tuft; scutellar discal setae situated as wide apart as subapical scutellar setae. Wing vein R_4+5_ with at most 2–3 small setulae dorsally at base. **Abdomen** (Fig. 21a, d): ground color dark brown-orange; with uninterrupted transverse marginal pollinose bands; pollinosity gold dorsally, silver ventrally, and with an orange spot lateroventrally at base of ST1+2; T3 with one pair of median marginal setae and two pairs of discal setae; T4 with two pairs of discal setae. **Terminalia** (Fig. 22): sternite 5 (Fig. 22c) with two small lobes and a wide U-shaped median cleft, 0.37X the length of the sternite from lobe to apex; inner margin covered by dense pollinosity, appearing darker than surrounding cuticle; entire lobe of sternite with sparse short setae, of varying lengths. Cerci (Fig. 22b), in dorsal view, separated by a narrow gap widening at apex; slender and straight; cercus, in lateral view, long, very slightly tapered from its already narrow base, setose along its basal half. Surstylus (Fig. 22a) subequal to length of cercus, turned slightly inward at tip, appearing as a small apical lobe when viewed dorsally; surstylus slender and digitiform when viewed laterally; with short setae along entire length; tip of surstylus not curved inwards when viewed dorsally.

**Female** (Fig. 21d, e, f). Length: 5–7mm. Fronto-orbital plate 1.6X as wide as in male; palpus significantly inflated apically.

#### Diagnosis

*Calolydella inflatipalpis* can be distinguished from all other species of *Calolydella* by the following combination of traits: fine setulae interspersed among frontal setae, palps normal in male but significantly inflated in female, anatergite with three or more hair-like setae arranged in a small tuft, and T3 and T4 each with two pairs of discal setae.

#### Etymology

The specific epithet is derived from the Latin adjective “*inflatum*”, meaning inflated, and "*palpus*", meaning palp, in reference to the inflated, bulbous palpi of females of this species.

#### Distribution

Costa Rica, ACG, Alajuela and Guanacaste provinces, 290–1150m.

#### Ecology

*Calolydella inflatipalpis* has been reared 31 times from four separate species of Heliconiini: *Dione juno* (Cramer, 1779), *Dryas iulia* (Fab., 1775), *Dryadula phaetusa* (Linn., 1758), and *Heliconius charithonia* (Linn., 1767) (Lepidoptera: Nymphalidae), in rain forest, dry forest, cloud forest and dry-rain lowland intergrade ecosystems.

### Calolydella interrupta

Fleming & Wood sp. n.

urn:lsid:zoobank.org:act:1B9143F7-AA12-4105-9EF3-B5BA3B836D20

#### Materials

- **Type status:** Holotype. **Occurrence:** occurrenceDetails: http://janzen.sas.upenn.edu; catalogNumber: DHJPAR0029598; recordedBy: D.H. Janzen, W. Hallwachs & Dunia Garcia; individualID: DHJPAR0029598; individualCount: 1; sex: M; lifeStage: adult; preparations: pinned; otherCatalogNumbers: 08-SRNP-36564, BOLD:AAW8657, ASHYM1019-09; **Taxon:** scientificName: Calolydella interrupta; phylum: Arthropoda; class: Insecta; order: Diptera; family: Tachinidae; genus: Calolydella; specificEpithet: interrupta; scientificNameAuthorship: Fleming & Wood, 2016; **Location:** continent: Central America; country: Costa Rica; countryCode: CR; stateProvince: Guanacaste; county: Sector Cacao; locality: Area de Conservacion Guanacaste; verbatimLocality: Sendero Arenales; verbatimElevation: 1080; verbatimLatitude: 10.9247; verbatimLongitude: -85.4674; verbatimCoordinateSystem: Decimal; **Identification:** identifiedBy: AJ Fleming; dateIdentified: 2016; **Event:** samplingProtocol: Reared from the larva of the limacodid moth, Acharia ophelians; verbatimEventDate: 17-Sep-2008; **Record Level:** language: en; institutionCode: CNC; collectionCode: Insects; basisOfRecord: Pinned Specimen
- **Type status:** Paratype. **Occurrence:** occurrenceDetails: http://janzen.sas.upenn.edu; catalogNumber: 08-SRNP-36564; recordedBy: D.H. Janzen, W. Hallwachs & Dunia Garcia; individualID: 08-SRNP-36564; individualCount: 1, sibling of holotype; sex: F; lifeStage: adult; preparations: pinned; otherCatalogNumbers: 08-SRNP-36564; **Taxon:** scientificName: Calolydella interrupta; phylum: Arthropoda; class: Insecta; order: Diptera; family: Tachinidae; genus: Calolydella; specificEpithet: interrupta; scientificNameAuthorship: Fleming & Wood, 2016; **Location:** continent: Central America; country: Costa Rica; countryCode: CR; stateProvince: Guanacaste; county: Sector Cacao; locality: Area de Conservacion Guanacaste; verbatimLocality: Sendero Arenales; verbatimElevation: 1080; verbatimLatitude: 10.9247; verbatimLongitude: -85.4674; verbatimCoordinateSystem: Decimal; **Identification:** identifiedBy: AJ Fleming; dateIdentified: 2016; **Event:** samplingProtocol: Reared from the larva of the limacodid moth, Acharia ophelians; verbatimEventDate: 17-Sep-2008; **Record Level:** language: en; institutionCode: CNC; collectionCode: Insects; basisOfRecord: Pinned Specimen

#### Description

**Male** (Fig. 23a, b, c). Length: 8mm. **Head** (Fig. 23b): frontal setae extending to base of postpedicel; fronto-orbital plate gold, ranging from bare to almost bare; parafacial silver along lower half. **Thorax** (Fig. 23a, c): gold on dorsal surface, silver laterally (>50% coverage); with two bold thoracic vittae; postpronotum with three setae; 2:3 acrostichal setae; 2:3 dorsocentral setae; 2:3 intra-alar setae; 1:3 supra-alar setae; three katepisternal setae; anatergite with three or more hair-like setae, often in a small tuft; scutellar discal setae slightly closer together than subapical scutellar setae. Wing vein R_4+5_ with 2–4 setulae dorsally, confined to base of wing vein, not extending up to crossvein R-M. **Abdomen** (Fig. 23a): ground color dark orange, with a median dark stripe breaking up pollinose marginal banding; abdominal pollinosity gold dorsally, silver ventrally; base of ST1+2 black lateroventrally, mostly gold pollinose dorsally; T3 with a row of marginal setae and one pair of discal setae; T4 with one pair of discal setae. **Terminalia**: not examined.

**Female** (Fig. 23d, e, f). Length: 6mm. Fronto-orbital plate 2X as wide as in male.

#### Diagnosis

*Calolydella interrupta* can be distinguished from all other species of *Calolydella* by the following combination of traits: parafacial at least 50% silver pollinose, two bold thoracic vittae, abdominal pollinosity interrupted by median stripe, anatergite with three or more setae arranged in a small tuft, and T3 with a complete row of marginal setae.

#### Etymology

The specific epithet is derived from the Latin adjective “*interruptum*", meaning breach or interruption, in reference to dark median stripe breaking up the gold marginal pollinosity on the abdomen of this species.

#### Distribution

Costa Rica, ACG, Guanacaste Province, Sendero Arenales 1080m.

#### Ecology

*Calolydella interrupta* has been reared once from *Acharia ophelians* (Dyar, 1927) (Lepidoptera: Limacodidae), in cloud forest.

### Calolydella nigripalpis

Fleming & Wood sp. n.

urn:lsid:zoobank.org:act:612EE188-6B86-49E4-BD72-EBCAE03A2D91

#### Materials

- **Type status:** Holotype. **Occurrence:** occurrenceDetails: http://janzen.sas.upenn.edu; catalogNumber: DHJPAR0017812; recordedBy: D.H. Janzen, W. Hallwachs & Carolina Cano; individualID: DHJPAR0017812; individualCount: 1; sex: M; lifeStage: adult; preparations: pinned; otherCatalogNumbers: 05-SRNP-1172, BOLD:ABY2592, ASTAR523-07; **Taxon:** scientificName: Calolydella nigripalpis; phylum: Arthropoda; class: Insecta; order: Diptera; family: Tachinidae; genus: Calolydella; specificEpithet: nigripalpis; scientificNameAuthorship: Fleming & Wood, 2016; **Location:** continent: Central America; country: Costa Rica; countryCode: CR; stateProvince: Alajuela; county: Sector San Cristobal; locality: Area de Conservacion Guanacaste; verbatimLocality: Puente Palma; verbatimElevation: 460; verbatimLatitude: 10.9163; verbatimLongitude: -85.3787; verbatimCoordinateSystem: Decimal; **Identification:** identifiedBy: AJ Fleming; dateIdentified: 2016; **Event:** samplingProtocol: Reared from the larva of the geometrid moth, Semaeopus Janzen08; verbatimEventDate: 04-Apr-2005; **Record Level:** language: en; institutionCode: CNC; collectionCode: Insects; basisOfRecord: Pinned Specimen
- **Type status:** Paratype. **Occurrence:** occurrenceDetails: http://janzen.sas.upenn.edu; catalogNumber: DHJPAR0040836; recordedBy: D.H. Janzen, W. Hallwachs & Ricardo Calero; individualID: DHJPAR0040836; individualCount: 1; sex: M; lifeStage: adult; preparations: pinned; otherCatalogNumbers: 10-SRNP-73126, BOLD:ABY2592, ASHYF751-11; **Taxon:** scientificName: Calolydella nigripalpis; phylum: Arthropoda; class: Insecta; order: Diptera; family: Tachinidae; genus: Calolydella; specificEpithet: nigripalpis; scientificNameAuthorship: Fleming & Wood, 2016; **Location:** continent: Central America; country: Costa Rica; countryCode: CR; stateProvince: Guanacaste; county: Sector Pitilla; locality: Area de Conservacion Guanacaste; verbatimLocality: Calma; verbatimElevation: 412; verbatimLatitude: 11.0099; verbatimLongitude: -85.3921; verbatimCoordinateSystem: Decimal; **Identification:** identifiedBy: AJ Fleming; dateIdentified: 2016; **Event:** samplingProtocol: Reared from the larva of the geometrid moth, Semaeopus Janzen08; verbatimEventDate: 20-Nov-2010; **Record Level:** language: en; institutionCode: CNC; collectionCode: Insects; basisOfRecord: Pinned Specimen
- **Type status:** Paratype. **Occurrence:** occurrenceDetails: http://janzen.sas.upenn.edu; catalogNumber: DHJPAR0052029; recordedBy: D.H. Janzen, W. Hallwachs & Edwin Apu; individualID: DHJPAR0052029; individualCount: 1; sex: M; lifeStage: adult; preparations: pinned; otherCatalogNumbers: 13-SRNP-69658, BOLD:ABY2592, ASHYH1141-13; **Taxon:** scientificName: Calolydella nigripalpis; phylum: Arthropoda; class: Insecta; order: Diptera; family: Tachinidae; genus: Calolydella; specificEpithet: nigripalpis; scientificNameAuthorship: Fleming & Wood, 2016; **Location:** continent: Central America; country: Costa Rica; countryCode: CR; stateProvince: Alajuela; county: Sector Rincon Rain Forest; locality: Area de Conservacion Guanacaste; verbatimLocality: Jacobo; verbatimElevation: 461; verbatimLatitude: 10.9408; verbatimLongitude: -85.3177; verbatimCoordinateSystem: Decimal; **Identification:** identifiedBy: AJ Fleming; dateIdentified: 2016; **Event:** samplingProtocol: Reared from the larva of the geometrid moth, Semaeopus Janzen08; verbatimEventDate: 26-May-2013; **Record Level:** language: en; institutionCode: CNC; collectionCode: Insects; basisOfRecord: Pinned Specimen
- **Type status:** Paratype. **Occurrence:** occurrenceDetails: http://janzen.sas.upenn.edu; catalogNumber: DHJPAR0017811; recordedBy: D.H. Janzen, W. Hallwachs & Yessenia Mendoza; individualID: DHJPAR0017811; individualCount: 1; sex: M; lifeStage: adult; preparations: pinned; otherCatalogNumbers: 05-SRNP-4251, BOLD:ABY2592, ASTAR522-07; **Taxon:** scientificName: Calolydella nigripalpis; phylum: Arthropoda; class: Insecta; order: Diptera; family: Tachinidae; genus: Calolydella; specificEpithet: nigripalpis; scientificNameAuthorship: Fleming & Wood, 2016; **Location:** continent: Central America; country: Costa Rica; countryCode: CR; stateProvince: Alajuela; county: Sector San Cristobal; locality: Area de Conservacion Guanacaste; verbatimLocality: Potrero Argentina; verbatimElevation: 520; verbatimLatitude: 10.8902; verbatimLongitude: -85.388; verbatimCoordinateSystem: Decimal; **Identification:** identifiedBy: AJ Fleming; dateIdentified: 2016; **Event:** samplingProtocol: Reared from the larva of the geometrid moth, Semaeopus Janzen08; verbatimEventDate: 20-Aug-2005; **Record Level:** language: en; institutionCode: CNC; collectionCode: Insects; basisOfRecord: Pinned Specimen
- **Type status:** Paratype. **Occurrence:** occurrenceDetails: http://janzen.sas.upenn.edu; catalogNumber: DHJPAR0054198; recordedBy: D.H. Janzen, W. Hallwachs & Cirilo Umana; individualID: DHJPAR0054198; individualCount: 1; sex: M; lifeStage: adult; preparations: pinned; otherCatalogNumbers: 14-SRNP-75117, BOLD:ABY2592, ASHYD3366-14; **Taxon:** scientificName: Calolydella nigripalpis; phylum: Arthropoda; class: Insecta; order: Diptera; family: Tachinidae; genus: Calolydella; specificEpithet: nigripalpis; scientificNameAuthorship: Fleming & Wood, 2016; **Location:** continent: Central America; country: Costa Rica; countryCode: CR; stateProvince: Alajuela; county: Sector Rincon Rain Forest; locality: Area de Conservacion Guanacaste; verbatimLocality: Estacion Llanura; verbatimElevation: 135; verbatimLatitude: 10.9333; verbatimLongitude: -85.2533; verbatimCoordinateSystem: Decimal; **Identification:** identifiedBy: AJ Fleming; dateIdentified: 2016; **Event:** samplingProtocol: Reared from the larva of the geometrid moth, Semaeopus Janzen08; verbatimEventDate: 29-Jan-2014; **Record Level:** language: en; institutionCode: CNC; collectionCode: Insects; basisOfRecord: Pinned Specimen
- **Type status:** Paratype. **Occurrence:** occurrenceDetails: http://janzen.sas.upenn.edu; catalogNumber: DHJPAR0054195; recordedBy: D.H. Janzen, W. Hallwachs & Cirilo Umana; individualID: DHJPAR0054195; individualCount: 1; sex: M; lifeStage: adult; preparations: pinned; otherCatalogNumbers: 14-SRNP-75120, BOLD:ABY2592, ASHYD3363-14; **Taxon:** scientificName: Calolydella nigripalpis; phylum: Arthropoda; class: Insecta; order: Diptera; family: Tachinidae; genus: Calolydella; specificEpithet: nigripalpis; scientificNameAuthorship: Fleming & Wood, 2016; **Location:** continent: Central America; country: Costa Rica; countryCode: CR; stateProvince: Alajuela; county: Sector Rincon Rain Forest; locality: Area de Conservacion Guanacaste; verbatimLocality: Estacion Llanura; verbatimElevation: 135; verbatimLatitude: 10.9333; verbatimLongitude: -85.2533; verbatimCoordinateSystem: Decimal; **Identification:** identifiedBy: AJ Fleming; dateIdentified: 2016; **Event:** samplingProtocol: Reared from the larva of the geometrid moth, Semaeopus Janzen08; verbatimEventDate: 01-Feb-2014; **Record Level:** language: en; institutionCode: CNC; collectionCode: Insects; basisOfRecord: Pinned Specimen

#### Description

**Male** (Fig. 24a, b, c). Length: 5–6mm. **Head** (Fig. 24b): frontal setae extending to base of postpedicel; fronto-orbital plate gold, sparsely setulose throughout; parafacial silver throughout; palps black. **Thorax** (Fig. 24a, c): gold on both dorsal and lateral surfaces; four almost indistinct thoracic vittae barely visible except under certain angles of light; postpronotum with three setae; 3:3 acrostichal setae; 3:3 dorsocentral setae; 2:3 intra-alar setae; 2:3 supra-alar setae; three katepisternal setae; anatergite bare; scutellar discal setae situated as wide apart as subapical scutellar setae. Wing vein R_4+5_ with 6–7 setulae dorsally, reaching from the base almost to crossvein R-M. **Abdomen** (Fig. 24a): ground color black, with uninterrupted transverse marginal pollinose bands; pollinosity gold dorsally, silver ventrally; base of ST1+2 black lateroventrally; T3 with one pair of median marginal setae and two pairs of discal setae; T4 with a row of marginal setae and two pairs of discal setae. **Terminalia** (Fig. 25): sternite 5 (Fig. 25c) with two small lobes and a wide U-shaped median cleft, 0.62X the length of the sternite from lobe to apex; inner margin covered by dense pollinosity, appearing darker than surrounding cuticle; with 4–6 short setae, of varying lengths. Cerci (Fig. 25b), in dorsal view, separated by a narrow gap, divergent at apex; each cercus sharply tapered, 2.2X broader at base than at apex, digitiform when viewed dorsally; cercus with a very slight downward bend at tip when viewed laterally; setose along its entire length. Surstylus (Fig. 25a) digitiform, with a strong apical downward curve giving a hooked tip, appearing 0.2X longer than cercus when viewed laterally; with short setae along entire length; tip of surstylus appearing curved inwards when viewed dorsally.

**Female**: not known at this time.

#### Diagnosis

*Calolydella nigripalpis* can be distinguished from all other species of *Calolydella* by the following combination of characters: frontal setae not extending beyond base of postpedicel, palps black in male, four barely visible thoracic vittae, anatergite bare, and abdominal ground color black.

#### Etymology

The specific epithet is derived from the Latin adjective "*nigrum*", meaning black, and "*palpus*", meaning palp, in reference to the black palpi of males of this species.

#### Distribution

Costa Rica, ACG, Alajuela and Guanacaste provinces, 135–520m.

#### Ecology

*Calolydella nigripalpis* has been reared six times from *Semaeopus* Janzen08 (Lepidoptera: Geometridae), in rain forest.

### Calolydella omissa

Fleming & Wood sp. n.

urn:lsid:zoobank.org:act:7E31F35E-1BFB-4A68-B246-F0E22EC4AEDD

#### Materials

- **Type status:** Holotype. **Occurrence:** occurrenceDetails: http://janzen.sas.upenn.edu; catalogNumber: DHJPAR0016605; recordedBy: D.H. Janzen, W. Hallwachs & Jose Perez; individualID: DHJPAR0016605; individualCount: 1; sex: M; lifeStage: adult; preparations: pinned; otherCatalogNumbers: 06-SRNP-43825, BOLD:AAD9874, ASTAP809-07; **Taxon:** scientificName: Calolydella omissa; phylum: Arthropoda; class: Insecta; order: Diptera; family: Tachinidae; genus: Calolydella; specificEpithet: omissa; scientificNameAuthorship: Fleming & Wood, 2016; **Location:** continent: Central America; country: Costa Rica; countryCode: CR; stateProvince: Alajuela; county: Sector Rincon Rain Forest; locality: Area de Conservacion Guanacaste; verbatimLocality: Cabanya; verbatimElevation: 340; verbatimLatitude: 10.877; verbatimLongitude: -85.2308; verbatimCoordinateSystem: Decimal; **Identification:** identifiedBy: AJ Fleming; dateIdentified: 2016; **Event:** samplingProtocol: Reared from the larva of the erebid moth, Letis buteoDHJ03; verbatimEventDate: 02-Nov-2006; **Record Level:** language: en; institutionCode: CNC; collectionCode: Insects; basisOfRecord: Pinned Specimen
- **Type status:** Paratype. **Occurrence:** occurrenceDetails: http://janzen.sas.upenn.edu; catalogNumber: 06-SRNP-43825; recordedBy: D.H. Janzen, W. Hallwachs & Jose Perez; individualID: 06-SRNP-43825; individualCount: 5; sex: 3M, 2F; lifeStage: adult; preparations: pinned; otherCatalogNumbers: 06-SRNP-43825; **Taxon:** scientificName: Calolydella omissa; phylum: Arthropoda; class: Insecta; order: Diptera; family: Tachinidae; genus: Calolydella; specificEpithet: omissa; scientificNameAuthorship: Fleming & Wood, 2016; **Location:** continent: Central America; country: Costa Rica; countryCode: CR; stateProvince: Alajuela; county: Sector Rincon Rain Forest; locality: Area de Conservacion Guanacaste; verbatimLocality: Cabanya; verbatimElevation: 340; verbatimLatitude: 10.877; verbatimLongitude: -85.2308; verbatimCoordinateSystem: Decimal; **Identification:** identifiedBy: AJ Fleming; dateIdentified: 2016; **Event:** samplingProtocol: Reared from the larva of the erebid moth, Letis buteoDHJ03; verbatimEventDate: 02-Nov-2006; **Record Level:** language: en; institutionCode: CNC; collectionCode: Insects; basisOfRecord: Pinned Specimen

#### Description

**Male** (Fig. 26a, b, c). Length: 7–8mm. **Head** (Fig. 26b): frontal setae extending to base of postpedicel; fronto-orbital plate gold, with a single row of fine setulae outside of frontal setae; parafacial silver throughout. **Thorax** (Fig. 26a, c): gold on dorsal surface, silver laterally (>50% coverage); with four regular thoracic vittae; postpronotum with four setae; 3:3 acrostichal setae; 2:3 dorsocentral setae; 2:3 intra-alar setae; 2:3 supra-alar setae; three katepisternal setae; anatergite bare or rarely with up to two hair-like setae; scutellar discal setae situated as wide apart as subapical scutellar setae. Wing vein R_4+5_ with 6–7 setulae dorsally, reaching from base almost to crossvein R-M. **Abdomen** (Fig. 26a): with uninterrupted transverse marginal pollinose bands; abdominal pollinosity gold dorsally, silver ventrally, and with an orange spot lateroventrally at base of ST1+2; T3 with one pair of median marginal setae, lacking discal setae; T4 with one pair of discal setae. **Terminalia** sternite 5 (Fig. 27c): with two small lobes and a wide U-shaped median cleft, 0.45X the length of the sternite from lobe to apex; inner margin covered by dense pollinosity, appearing darker than surrounding cuticle; entire lobe of sternite with short setae, of varying lengths. Cerci (Fig. 27b), in dorsal view, separated by a narrow gap but rejoining at apex; each cercus long and very slightly tapered, almost of equal width along entire length; cercus slender with an upward bend when viewed laterally; setose along its entire length. Surstylus (Fig. 27a) slender and digitiform with a slight upward curve, appearing 0.2X shorter than cercus when viewed laterally; with short setae along entire length; tip of surstylus very slightly curved inwards when viewed dorsally.

**Female** (Fig. 26d, e, f). Length: 5–6mm. Fronto-orbital plate 1.4X wider than in male.

#### Diagnosis

*Calolydella omissa* can be distinguished from all other species of *Calolydella* by the following combination of traits: fronto-orbital plate gold, parafacial silver, abdomen with uninterrupted transverse silver pollinose bands across all tergites, anatergite bare (rarely setose), and T3 without discal setae.

#### Etymology

The specific epithet is derived from the Latin adjective "*omissa*", meaning dropped or absent, in reference to the absence of discal setae on T3, a character state unique to this species.

#### Distribution

Costa Rica, ACG, Alajuela Province, Cabanya, 340m.

#### Ecology

*Calolydella omissa* has been reared once from *Letis* Janzen03 (Lepidoptera: Erebidae), in rain forest.

### Calolydella ordinalis

Fleming & Wood sp. n.

urn:lsid:zoobank.org:act:410DAC20-265A-4703-B31D-228436174914

#### Materials

- **Type status:** Holotype. **Occurrence:** occurrenceDetails: http://janzen.sas.upenn.edu; catalogNumber: DHJPAR0017787; recordedBy: D.H. Janzen, W. Hallwachs & Mariano Pereira; individualID: DHJPAR0017787; individualCount: 1; sex: M; lifeStage: adult; preparations: pinned; otherCatalogNumbers: 98-SRNP-15880, BOLD:ACF1517, ASTAR498-07; **Taxon:** scientificName: Calolydella ordinalis; phylum: Arthropoda; class: Insecta; order: Diptera; family: Tachinidae; genus: Calolydella; specificEpithet: ordinalis; scientificNameAuthorship: Fleming & Wood, 2016; **Location:** continent: Central America; country: Costa Rica; countryCode: CR; stateProvince: Guanacaste; county: Sector Cacao; locality: Area de Conservacion Guanacaste; verbatimLocality: Sendero Maritza; verbatimElevation: 760; verbatimLatitude: 10.9364; verbatimLongitude: -85.4776; verbatimCoordinateSystem: Decimal; **Identification:** identifiedBy: AJ Fleming; dateIdentified: 2016; **Event:** samplingProtocol: Reared from the larva of the erebid moth, Eloria torrida; verbatimEventDate: 20-Jan-1999; **Record Level:** language: en; institutionCode: CNC; collectionCode: Insects; basisOfRecord: Pinned Specimen
- **Type status:** Paratype. **Occurrence:** occurrenceDetails: http://janzen.sas.upenn.edu; catalogNumber: DHJPAR0017792; recordedBy: D.H. Janzen, W. Hallwachs & Mariano Pereira; individualID: DHJPAR0017792; individualCount: 1; sex: F; lifeStage: adult; preparations: pinned; otherCatalogNumbers: 98-SRNP-15883, BOLD:ACF1517, ASTAR503-07; **Taxon:** scientificName: Calolydella ordinalis; phylum: Arthropoda; class: Insecta; order: Diptera; family: Tachinidae; genus: Calolydella; specificEpithet: ordinalis; scientificNameAuthorship: Fleming & Wood, 2016; **Location:** continent: Central America; country: Costa Rica; countryCode: CR; stateProvince: Guanacaste; county: Sector Cacao; locality: Area de Conservacion Guanacaste; verbatimLocality: Sendero Maritza; verbatimElevation: 760; verbatimLatitude: 10.9364; verbatimLongitude: -85.4776; verbatimCoordinateSystem: Decimal; **Identification:** identifiedBy: AJ Fleming; dateIdentified: 2016; **Event:** samplingProtocol: Reared from the larva of the erebid moth, Eloria torrida; verbatimEventDate: 20-Jan-1999; **Record Level:** language: en; institutionCode: CNC; collectionCode: Insects; basisOfRecord: Pinned Specimen
- **Type status:** Paratype. **Occurrence:** occurrenceDetails: http://janzen.sas.upenn.edu; catalogNumber: DHJPAR0017791; recordedBy: D.H. Janzen, W. Hallwachs & Mariano Pereira; individualID: DHJPAR0017791; individualCount: 1; sex: M; lifeStage: adult; preparations: pinned; otherCatalogNumbers: 98-SRNP-15887, BOLD:ACF1517, ASTAR502-07; **Taxon:** scientificName: Calolydella ordinalis; phylum: Arthropoda; class: Insecta; order: Diptera; family: Tachinidae; genus: Calolydella; specificEpithet: ordinalis; scientificNameAuthorship: Fleming & Wood, 2016; **Location:** continent: Central America; country: Costa Rica; countryCode: CR; stateProvince: Guanacaste; county: Sector Cacao; locality: Area de Conservacion Guanacaste; verbatimLocality: Sendero Maritza; verbatimElevation: 760; verbatimLatitude: 10.9364; verbatimLongitude: -85.4776; verbatimCoordinateSystem: Decimal; **Identification:** identifiedBy: AJ Fleming; dateIdentified: 2016; **Event:** samplingProtocol: Reared from the larva of the erebid moth, Eloria torrida; verbatimEventDate: 20-Jan-1999; **Record Level:** language: en; institutionCode: CNC; collectionCode: Insects; basisOfRecord: Pinned Specimen
- **Type status:** Paratype. **Occurrence:** occurrenceDetails: http://janzen.sas.upenn.edu; catalogNumber: DHJPAR0017789; recordedBy: D.H. Janzen, W. Hallwachs & Mariano Pereira; individualID: DHJPAR0017789; individualCount: 1; sex: F; lifeStage: adult; preparations: pinned; otherCatalogNumbers: 98-SRNP-15883, BOLD:ACF1517, ASTAR500-07; **Taxon:** scientificName: Calolydella ordinalis; phylum: Arthropoda; class: Insecta; order: Diptera; family: Tachinidae; genus: Calolydella; specificEpithet: ordinalis; scientificNameAuthorship: Fleming & Wood, 2016; **Location:** continent: Central America; country: Costa Rica; countryCode: CR; stateProvince: Guanacaste; county: Sector Cacao; locality: Area de Conservacion Guanacaste; verbatimLocality: Sendero Maritza; verbatimElevation: 760; verbatimLatitude: 10.9364; verbatimLongitude: -85.4776; verbatimCoordinateSystem: Decimal; **Identification:** identifiedBy: AJ Fleming; dateIdentified: 2016; **Event:** samplingProtocol: Reared from the larva of the erebid moth, Eloria torrida; verbatimEventDate: 20-Jan-1999; **Record Level:** language: en; institutionCode: CNC; collectionCode: Insects; basisOfRecord: Pinned Specimen
- **Type status:** Paratype. **Occurrence:** occurrenceDetails: http://janzen.sas.upenn.edu; catalogNumber: DHJPAR0017790; recordedBy: D.H. Janzen, W. Hallwachs & Mariano Pereira; individualID: DHJPAR0017790; individualCount: 1; sex: M; lifeStage: adult; preparations: pinned; otherCatalogNumbers: 98-SRNP-15887, BOLD:ACF1517, ASTAR501-07; **Taxon:** scientificName: Calolydella ordinalis; phylum: Arthropoda; class: Insecta; order: Diptera; family: Tachinidae; genus: Calolydella; specificEpithet: ordinalis; scientificNameAuthorship: Fleming & Wood, 2016; **Location:** continent: Central America; country: Costa Rica; countryCode: CR; stateProvince: Guanacaste; county: Sector Cacao; locality: Area de Conservacion Guanacaste; verbatimLocality: Sendero Maritza; verbatimElevation: 760; verbatimLatitude: 10.9364; verbatimLongitude: -85.4776; verbatimCoordinateSystem: Decimal; **Identification:** identifiedBy: AJ Fleming; dateIdentified: 2016; **Event:** samplingProtocol: Reared from the larva of the erebid moth, Eloria torrida; verbatimEventDate: 20-Jan-1999; **Record Level:** language: en; institutionCode: CNC; collectionCode: Insects; basisOfRecord: Pinned Specimen
- **Type status:** Paratype. **Occurrence:** occurrenceDetails: http://janzen.sas.upenn.edu; catalogNumber: DHJPAR0017788; recordedBy: D.H. Janzen, W. Hallwachs & Mariano Pereira; individualID: DHJPAR0017788; individualCount: 1; sex: M; lifeStage: adult; preparations: pinned; otherCatalogNumbers: 98-SRNP-15891, BOLD:ACF1517, ASTAR499-07; **Taxon:** scientificName: Calolydella ordinalis; phylum: Arthropoda; class: Insecta; order: Diptera; family: Tachinidae; genus: Calolydella; specificEpithet: ordinalis; scientificNameAuthorship: Fleming & Wood, 2016; **Location:** continent: Central America; country: Costa Rica; countryCode: CR; stateProvince: Guanacaste; county: Sector Cacao; locality: Area de Conservacion Guanacaste; verbatimLocality: Sendero Maritza; verbatimElevation: 760; verbatimLatitude: 10.9364; verbatimLongitude: -85.4776; verbatimCoordinateSystem: Decimal; **Identification:** identifiedBy: AJ Fleming; dateIdentified: 2016; **Event:** samplingProtocol: Reared from the larva of the erebid moth, Eloria torrida; verbatimEventDate: 20-Jan-1999; **Record Level:** language: en; institutionCode: CNC; collectionCode: Insects; basisOfRecord: Pinned Specimen

#### Description

**Male** (Fig. 28a, b, c). Length: 6–8mm. **Head** (Fig. 28b): frontal setae extending to base of postpedicel; fronto-orbital plate gold, sparsely setulose, throughout; parafacial almost entirely gold (75-90% coverage). **Thorax** (Fig. 28a, c): gold on both dorsal and lateral surfaces; outermost two vittae twice as wide as innermost two; postpronotum with three setae; 3:3 acrostichal setae; 2:3 dorsocentral setae; 2:3 intra-alar setae; 2:3 supra-alar setae; three katepisternal setae; anatergite bare; scutellar discal setae situated as wide apart as or slightly wider apart than subapical scutellar setae. Wing vein R_4+5_ with at most 2–3 small setulae dorsally at base. **Abdomen** (Fig. 28a): ground color dark brown-orange to orange with uninterrupted transverse marginal pollinose bands; pollinosity gold dorsally, silver ventrally, and with an orange spot lateroventrally at base of ST1+2; T3 with one pair of median marginal setae and one to more than two pairs of discal setae; T4 with a row of marginal setae and a row of discal setae. **Terminalia** (Fig. 29): sternite 5 (Fig. 29a) with two small lobes and a wide U-shaped median cleft, 0.34X the length of the sternite from lobe to apex; inner margin covered by dense pollinosity, appearing darker than surrounding cuticle; entire lobe of sternite with short setae, of varying lengths. Cerci (Fig. 29b), in dorsal view, separated by a narrow gap, rejoining at apex; each cercus broad and digitiform, setose along its entire length; cercus with a very slight upward bend when viewed laterally. Surstylus (Fig. 29a) spatulate with a downward curve, appearing subequal to length of cercus when viewed laterally; with short setae along entire length; tip of surstylus not appearing curved inwards when viewed dorsally.

**Female** (Fig. 28d, e, f). Length: 5–6mm. Fronto-orbital plate 2.1X as wide as in male.

#### Diagnosis

*Calolydella ordinalis* can be distinguished from all other species of *Calolydella* by the following combination of characters: parafacial mostly gold, fronto-orbital plate with setulae sparsely interspersed among frontal setae, anatergite bare, and a complete row of discal setae on T4.

#### Etymology

The specific epithet is derived from the Latin adjective "*ordinarius*", meaning ordinary or complete, in reference to the complete, regular row of discal setae on T4, a character state unique to this species.

#### Distribution

Costa Rica, ACG, Guanacaste Province, Sendero Maritza, 760m.

#### Ecology

*Calolydella ordinalis* has been reared six times from *Eloria torrida* Schaus, 1910 (Lepidoptera: Erebidae) in cloud forest.

### Calolydella renemalaisei

Fleming & Wood sp. n.

urn:lsid:zoobank.org:act:FA671FB0-0BC4-48CA-86CE-B183BB3AD479

#### Materials

- **Type status:** Holotype. **Occurrence:** occurrenceDetails: http://janzen.sas.upenn.edu; catalogNumber: DHJPAR0011709; recordedBy: D.H. Janzen, W. Hallwachs & Manuel Rios; individualID: DHJPAR0011709; individualCount: 1; sex: M; lifeStage: adult; preparations: pinned; otherCatalogNumbers: 04-SRNP-31285, BOLD:AAD9853, ASTAS435-06; **Taxon:** scientificName: Calolydella renemalaisei; phylum: Arthropoda; class: Insecta; order: Diptera; family: Tachinidae; genus: Calolydella; specificEpithet: renemalaisei; scientificNameAuthorship: Fleming & Wood, 2016; **Location:** continent: Central America; country: Costa Rica; countryCode: CR; stateProvince: Guanacaste; county: Sector Pitilla; locality: Area de Conservacion Guanacaste; verbatimLocality: Sendero Mismo; verbatimElevation: 680; verbatimLatitude: 10.9876; verbatimLongitude: -85.4197; verbatimCoordinateSystem: Decimal; **Identification:** identifiedBy: AJ Fleming; dateIdentified: 2016; **Event:** samplingProtocol: Reared from the larva of the erebid moth, Eucereon aeolum; verbatimEventDate: 10-Apr-2004; **Record Level:** language: en; institutionCode: CNC; collectionCode: Insects; basisOfRecord: Pinned Specimen
- **Type status:** Paratype. **Occurrence:** occurrenceDetails: http://janzen.sas.upenn.edu; catalogNumber: DHJPAR0011710; recordedBy: D.H. Janzen, W. Hallwachs & Manuel Rios; individualID: DHJPAR0011710; individualCount: 1; sex: M; lifeStage: adult; preparations: pinned; otherCatalogNumbers: 04-SRNP-31280, BOLD:AAD9853, ASTAS436-06; **Taxon:** scientificName: Calolydella renemalaisei; phylum: Arthropoda; class: Insecta; order: Diptera; family: Tachinidae; genus: Calolydella; specificEpithet: renemalaisei; scientificNameAuthorship: Fleming & Wood, 2016; **Location:** continent: Central America; country: Costa Rica; countryCode: CR; stateProvince: Guanacaste; county: Sector Pitilla; locality: Area de Conservacion Guanacaste; verbatimLocality: Sendero Mismo; verbatimElevation: 680; verbatimLatitude: 10.9876; verbatimLongitude: -85.4197; verbatimCoordinateSystem: Decimal; **Identification:** identifiedBy: AJ Fleming; dateIdentified: 2016; **Event:** samplingProtocol: Reared from the larva of the erebid moth, Eucereon aeolum; verbatimEventDate: 08-Apr-2004; **Record Level:** language: en; institutionCode: CNC; collectionCode: Insects; basisOfRecord: Pinned Specimen
- **Type status:** Paratype. **Occurrence:** occurrenceDetails: http://janzen.sas.upenn.edu; catalogNumber: DHJPAR0057608; recordedBy: D.H. Janzen, W. Hallwachs & Gilberth Ampie; individualID: DHJPAR0057608; individualCount: 1; sex: M; lifeStage: adult; preparations: pinned; otherCatalogNumbers: 15-SRNP-891, BOLD:AAD9853, ASTAX020-15; **Taxon:** scientificName: Calolydella renemalaisei; phylum: Arthropoda; class: Insecta; order: Diptera; family: Tachinidae; genus: Calolydella; specificEpithet: renemalaisei; scientificNameAuthorship: Fleming & Wood, 2016; **Location:** continent: Central America; country: Costa Rica; countryCode: CR; stateProvince: Alajuela; county: Sector Rincon Rain Forest; locality: Area de Conservacion Guanacaste; verbatimLocality: Sendero al Crater; verbatimCoordinateSystem: Decimal; **Identification:** identifiedBy: AJ Fleming; dateIdentified: 2016; **Event:** samplingProtocol: Reared from the larva of the erebid moth, Eucereon aeolum; verbatimEventDate: 20-Apr-2015; **Record Level:** language: en; institutionCode: CNC; collectionCode: Insects; basisOfRecord: Pinned Specimen
- **Type status:** Paratype. **Occurrence:** occurrenceDetails: http://janzen.sas.upenn.edu; catalogNumber: DHJPAR0011705; recordedBy: D.H. Janzen, W. Hallwachs & Manuel Rios; individualID: DHJPAR0011705; individualCount: 1; sex: M; lifeStage: adult; preparations: pinned; otherCatalogNumbers: 04-SRNP-31286, BOLD:AAD9853, ASTAS431-06; **Taxon:** scientificName: Calolydella renemalaisei; phylum: Arthropoda; class: Insecta; order: Diptera; family: Tachinidae; genus: Calolydella; specificEpithet: renemalaisei; scientificNameAuthorship: Fleming & Wood, 2016; **Location:** continent: Central America; country: Costa Rica; countryCode: CR; stateProvince: Guanacaste; county: Sector Pitilla; locality: Area de Conservacion Guanacaste; verbatimLocality: Sendero Mismo; verbatimElevation: 680; verbatimLatitude: 10.9876; verbatimLongitude: -85.4197; verbatimCoordinateSystem: Decimal; **Identification:** identifiedBy: AJ Fleming; dateIdentified: 2016; **Event:** samplingProtocol: Reared from the larva of the erebid moth, Eucereon aeolum; verbatimEventDate: 09-Apr-2004; **Record Level:** language: en; institutionCode: CNC; collectionCode: Insects; basisOfRecord: Pinned Specimen
- **Type status:** Paratype. **Occurrence:** occurrenceDetails: http://janzen.sas.upenn.edu; catalogNumber: DHJPAR0011712; recordedBy: D.H. Janzen, W. Hallwachs & Manuel Rios; individualID: DHJPAR0011712; individualCount: 1; sex: M; lifeStage: adult; preparations: pinned; otherCatalogNumbers: 04-SRNP-31283, BOLD:AAD9853, ASTAS438-06; **Taxon:** scientificName: Calolydella renemalaisei; phylum: Arthropoda; class: Insecta; order: Diptera; family: Tachinidae; genus: Calolydella; specificEpithet: renemalaisei; scientificNameAuthorship: Fleming & Wood, 2016; **Location:** continent: Central America; country: Costa Rica; countryCode: CR; stateProvince: Guanacaste; county: Sector Pitilla; locality: Area de Conservacion Guanacaste; verbatimLocality: Sendero Mismo; verbatimElevation: 680; verbatimLatitude: 10.9876; verbatimLongitude: -85.4197; verbatimCoordinateSystem: Decimal; **Identification:** identifiedBy: AJ Fleming; dateIdentified: 2016; **Event:** samplingProtocol: Reared from the larva of the erebid moth, Eucereon aeolum; verbatimEventDate: 10-Apr-2004; **Record Level:** language: en; institutionCode: CNC; collectionCode: Insects; basisOfRecord: Pinned Specimen
- **Type status:** Paratype. **Occurrence:** occurrenceDetails: http://janzen.sas.upenn.edu; catalogNumber: DHJPAR0017809; recordedBy: D.H. Janzen, W. Hallwachs & Elieth Cantillano; individualID: DHJPAR0017809; individualCount: 1; sex: M; lifeStage: adult; preparations: pinned; otherCatalogNumbers: 03-SRNP-2236, BOLD:AAD9853, ASTAR520-07; **Taxon:** scientificName: Calolydella renemalaisei; phylum: Arthropoda; class: Insecta; order: Diptera; family: Tachinidae; genus: Calolydella; specificEpithet: renemalaisei; scientificNameAuthorship: Fleming & Wood, 2016; **Location:** continent: Central America; country: Costa Rica; countryCode: CR; stateProvince: Guanacaste; county: Sector Del Oro; locality: Area de Conservacion Guanacaste; verbatimLocality: Puente Mena; verbatimElevation: 280; verbatimLatitude: 11.0456; verbatimLongitude: -85.4574; verbatimCoordinateSystem: Decimal; **Identification:** identifiedBy: AJ Fleming; dateIdentified: 2016; **Event:** samplingProtocol: Reared from the larva of the erebid moth, Leucotmemis nexa; verbatimEventDate: 20-Jun-2003; **Record Level:** language: en; institutionCode: CNC; collectionCode: Insects; basisOfRecord: Pinned Specimen

#### Description

**Male** (Fig. 30a, b, c). Length: 5–8mm. **Head** (Fig. 30b): frontal setae extending to base of postpedicel; fronto-orbital plate gold, sparsely setulose throughout; parafacial up to 50% silver pollinose or mostly gold (75-90%). **Thorax** (Fig. 30a, c): gold on both dorsal and lateral surfaces; with four regular thoracic vittae; postpronotum with three setae; 3:3 acrostichal setae; 3:3 dorsocentral setae; 1:3 intra-alar setae; 2:3 supra-alar setae; three katepisternal setae; anatergite with three or more hair-like setae, often in a small tuft; scutellar discal setae situated as wide apart as subapical scutellar setae or absent. Wing vein R_4+5_ with at most 2–3 setulae dorsally at base, not extending to crossvein R-M. **Abdomen** (Fig. 30a): ground color dark brown-orange with uninterrupted transverse marginal pollinose bands; gold dorsally, silver ventrally, and with an orange spot lateroventrally at base of ST1+2 or base of ST1+2 black lateroventrally; T3 with one pair of median marginal setae and one pair of discal setae; T4 with 1–2 pairs of discal setae. **Terminalia** (Fig. 31): sternite 5 (Fig. 31c) with two small lobes and a wide U-shaped median cleft, 0.47X the length of the sternite from lobe to apex; inner margin covered by dense pollinosity, appearing darker than surrounding cuticle; entire lobe of sternite with short setae, all of equal length. Cerci (Fig. 31b), in dorsal view, separated by a narrow gap widening at apex; each cercus long, slender and straight, very slightly tapered from its already narrow base; setose along basal half. Surstylus (Fig. 31a) subequal to length of cercus, broad and digitiform when viewed laterally; with short setae along entire length; tip of surstylus not curved inwards when viewed dorsally.

**Female**: not known at this time.

#### Diagnosis

*Calolydella renemalaisei* can be distinguished from all other other species of *Calolydella* by the following combination of characters: frontal setae extending to base of postpedicel, fronto-orbital plate sparsely setose througout, with four thoracic vittae, the outermost two twice as wide as the innermost two, anatergite with three or more hair-like setae arranged in a small tuft, and up to two pairs of discal setae on T4.

#### Etymology

The specific epithet is in honor of René Edmond Malaise (1892-1978) of Stockholm, Sweden, in recognition of his invention of the Malaise trap, which has captured so many ACG Tachinidae and other insects, as explained by Fredrik Sjöberg in his wonderful book "The Fly Trap" (Sjöberg 2014, translation by Thomas Teal), a book that explains much about Malaise traps.

#### Distribution

Costa Rica, ACG, Guanacaste Province, 280–680m.

#### Ecology

*Calolydella renemalaisei* has been reared six times from two species of Arctiinae: *Euceron aeolum* Hampson, 1898, and *Leucotmemis nexa* Herrich-Schäffer, 1854 (Lepidoptera: Erebidae), in rain forest and dry-rain lowland intergrade ecosystems.

### Calolydella susanaroibasae

Fleming & Wood sp. n.

urn:lsid:zoobank.org:act:A67E6DB2-D3C1-47BB-B098-B7373D8BEBC4

#### Materials

- **Type status:** Holotype. **Occurrence:** occurrenceDetails: http://janzen.sas.upenn.edu; catalogNumber: DHJPAR0057699; recordedBy: D.H. Janzen, W. Hallwachs & Carolina Cano; individualID: DHJPAR0057699; individualCount: 1; sex: M; lifeStage: adult; preparations: pinned; otherCatalogNumbers: 15-SRNP-1269, BOLD:ACW6315, ASTAX072-15; **Taxon:** scientificName: Calolydella susanaroibasae; phylum: Arthropoda; class: Insecta; order: Diptera; family: Tachinidae; genus: Calolydella; specificEpithet: susanaroibasae; scientificNameAuthorship: Fleming & Wood, 2016; **Location:** continent: Central America; country: Costa Rica; countryCode: CR; stateProvince: Alajuela; county: Sector San Cristobal; locality: Area de Conservacion Guanacaste; verbatimLocality: Rio Blanco Abajo; verbatimElevation: 500; verbatimLatitude: 10.9004; verbatimLongitude: -85.3725; verbatimCoordinateSystem: Decimal; **Identification:** identifiedBy: AJ Fleming; dateIdentified: 2016; **Event:** samplingProtocol: Reared from the larva of the erebid moth, Isanthrene echemon; verbatimEventDate: 20-May-2015; **Record Level:** language: en; institutionCode: CNC; collectionCode: Insects; basisOfRecord: Pinned Specimen
- **Type status:** Paratype. **Occurrence:** occurrenceDetails: http://janzen.sas.upenn.edu; catalogNumber: 15-SRNP-1269; recordedBy: D.H. Janzen, W. Hallwachs & Carolina Cano; individualID: 15-SRNP-1269; individualCount: 2; sex: 2F, siblings to holotype; lifeStage: adult; preparations: pinned; otherCatalogNumbers: 15-SRNP-1269; **Taxon:** scientificName: Calolydella susanaroibasae; phylum: Arthropoda; class: Insecta; order: Diptera; family: Tachinidae; genus: Calolydella; specificEpithet: susanaroibasae; scientificNameAuthorship: Fleming & Wood, 2016; **Location:** continent: Central America; country: Costa Rica; countryCode: CR; stateProvince: Alajuela; county: Sector San Cristobal; locality: Area de Conservacion Guanacaste; verbatimLocality: Rio Blanco Abajo; verbatimElevation: 500; verbatimLatitude: 10.9004; verbatimLongitude: -85.3725; verbatimCoordinateSystem: Decimal; **Identification:** identifiedBy: AJ Fleming; dateIdentified: 2016; **Event:** samplingProtocol: Reared from the larva of the erebid moth, Isanthrene echemon; verbatimEventDate: 20-May-2015; **Record Level:** language: en; institutionCode: CNC; collectionCode: Insects; basisOfRecord: Pinned Specimen

#### Description

**Male** (Fig. 32a, b, c). Length: 9mm. **Head** (Fig. 32b): frontal setae extending to base of postpedicel; fronto-orbital plate gold, with a single row of fine setulae outside of frontal setae; parafacial silver along lower half. **Thorax** (Fig. 32a, c): gold on dorsal surface, silver laterally (>50% coverage); outermost two vittae twice as wide as innermost two; postpronotum with three setae; 3:3 acrostichal setae; 3:3 dorsocentral setae; 2:3 intra-alar setae; 2:3 supra-alar setae 2:3; three katepisternal setae; anatergite with three or more hair-like setae, often in a small tuft, or rarely bare; scutellar discal setae situated as wide apart as subapical scutellar setae. Wing vein R_4+5_ with 5–7 setulae dorsally, reaching from base almost to crossvein R-M. **Abdomen** (Fig. 32a): ground color dark brown-orange, with transverse marginal pollinose bands; gold pollinosity dorsally, silver ventrally; base of ST1+2 black lateroventrally; T3 with one pair of median marginal setae and one pair of discal setae; T4 with 1–2 pairs of discal setae. **Terminalia**: not examined.

**Female** (Fig. 32d, e, f) . Length: 6mm. Fronto-orbital plate 1.7X as wide as in male.

#### Diagnosis

*Calolydella susanaroibasae* can be distinguished from all other species of *Calolydella* by the following combination of characters: fronto-orbital plate with a single row of fine setulae outside of frontal setae, thoracic pollinosity gold dorsally and on 50% of lateral surface, with outer two thoracic vittae twice as wide as inner two, anatergite with three or more hair-like setae arranged in a small tuft, and base of ST1+2 black lateroventrally.

#### Etymology

The specific epithet is in honor of Susana Roibas of Lexington, Kentucky, in recognition of her company "Sante-Traps" (http://www.santetraps.com), which has long supplied the world with Malaise traps and other kinds of insect traps, many of which have been used to collect ACG Tachinidae and other insects.

#### Distribution

Costa Rica, ACG, Alajuela Province, Rio Blanco Abajo, 500m.

#### Ecology

*Calolydella susanaroibasae* has been reared once from *Isanthrene echemon* Druce, 1884 (Lepidoptera: Erebidae: Arctiinae), in rain forest.

### Calolydella tanyadapkeyae

Fleming & Wood sp. n.

urn:lsid:zoobank.org:act:C7F91FFF-F269-4D02-BAA6-903F6B5BBB28

#### Materials

- **Type status:** Holotype. **Occurrence:** occurrenceDetails: http://janzen.sas.upenn.edu; catalogNumber: DHJPAR0011701; recordedBy: D.H. Janzen, W. Hallwachs & Jose Perez; individualID: DHJPAR0011701; individualCount: 1; sex: F; lifeStage: adult; preparations: pinned; otherCatalogNumbers: 05-SRNP-41436, BOLD:AAW8655, ASTAS427-06; **Taxon:** scientificName: Calolydella tanyadapkeyae; phylum: Arthropoda; class: Insecta; order: Diptera; family: Tachinidae; genus: Calolydella; specificEpithet: tanyadapkeyae; scientificNameAuthorship: Fleming & Wood, 2016; **Location:** continent: Central America; country: Costa Rica; countryCode: CR; stateProvince: Alajuela; county: Sector Rincon Rain Forest; locality: Area de Conservacion Guanacaste; verbatimLocality: Camino Rio Francia; verbatimElevation: 410; verbatimLatitude: 10.9043; verbatimCoordinateSystem: Decimal; **Identification:** identifiedBy: AJ Fleming; dateIdentified: 2016; **Event:** samplingProtocol: Reared from the larva of the erebid moth, Napata flaviceps; verbatimEventDate: 25-Jun-2005; **Record Level:** language: en; institutionCode: CNC; collectionCode: Insects; basisOfRecord: Pinned Specimen

#### Description

**Female** (Fig. 33a, b, c). Length: 6mm. **Head** (Fig. 33b): frontal setae extending to base of postpedicel; fronto-orbital plate gold throughout, and sparsely setulose; parafacial silver throughout. **Thorax** (Fig. 33a, c): gold on dorsal surface, silver laterally (>50% coverage); with four regular thoracic vittae; postpronotum with two setae (inner basal seta absent); 3:3 acrostichal setae; 2:3 dorsocentral setae; 2:3 intra-alar setae; 2:3 supra-alar setae; three katepisternal setae; anatergite bare; scutellar discal setae situated as wide apart as subapical scutellar setae. Wing vein R4+5 with 6–7 setulae dorsally, extending to crossvein R-M. **Abdomen** (Fig. 33a): ground color black, with uninterrupted transverse marginal pollinose bands and with both dorsal and ventral surfaces concolorous; base of ST1+2 black lateroventrally; T3 with one pair of median marginal setae and one pair of discal setae; T4 with one pair of discal setae.

**Male**: not known at this time.

#### Diagnosis

*Calolydella tanyadapkeyae* can be distinguished from all other other species of *Calolydella* by the following combination of traits: parafacial all silver, frontal setae extending to base of postpedicel, thoracic pollinosity gold dorsally and over 50% of lateral surfaces, postpronotum with only two setae (inner basal seta absent), anatergite bare, and scutellar discal setae situated as wide apart as subapical scutellar setae.

#### Etymology

The specific epithet is in honor of Tanya Dapkey James of Levittown, Pennsylvania, in recognition of her efforts curating and preparing ACG parasitoid flies for DNA barcoding.

#### Distribution

Costa Rica, ACG, Alajuela Province, Camino Rio Francia, 410m.

#### Ecology

*Calolydella tanyadapkeyae* has been reared once from *Uranophora flaviceps* (Hampson, 1901) (Lepidoptera: Erebidae), in rain forest.

### Calolydella tenebrosa

Fleming & Wood sp. n.

urn:lsid:zoobank.org:act:1D33CB25-2145-45FC-B696-5E25C44CD4BD

#### Materials

- **Type status:** Holotype. **Occurrence:** occurrenceDetails: http://janzen.sas.upenn.edu; catalogNumber: DHJPAR0017810; recordedBy: D.H. Janzen, W. Hallwachs & Jose Perez; individualID: DHJPAR0017810; individualCount: 1; sex: M; lifeStage: adult; preparations: pinned; otherCatalogNumbers: 05-SRNP-40421, BOLD:AAG2405, ASTAR521-07; **Taxon:** scientificName: Calolydella tenebrosa; phylum: Arthropoda; class: Insecta; order: Diptera; family: Tachinidae; genus: Calolydella; specificEpithet: tenebrosa; scientificNameAuthorship: Fleming & Wood, 2016; **Location:** continent: Central America; country: Costa Rica; countryCode: CR; stateProvince: Alajuela; county: Sector Rincon Rain Forest; locality: Area de Conservacion Guanacaste; verbatimLocality: Vochysia; verbatimElevation: 320; verbatimLatitude: 10.8667; verbatimLongitude: -85.2453; verbatimCoordinateSystem: Decimal; **Identification:** identifiedBy: AJ Fleming; dateIdentified: 2016; **Event:** samplingProtocol: Reared from the larvae of the geometrid moth, Cyclophora Janzen14; verbatimEventDate: 01-Mar-2005; **Record Level:** language: en; institutionCode: CNC; collectionCode: Insects; basisOfRecord: Pinned Specimen
- **Type status:** Paratype. **Occurrence:** occurrenceDetails: http://janzen.sas.upenn.edu; catalogNumber: DHJPAR0053996; recordedBy: D.H. Janzen, W. Hallwachs & Ricardo Calero; individualID: DHJPAR0053996; individualCount: 1; sex: F; lifeStage: adult; preparations: pinned; otherCatalogNumbers: 13-SRNP-71761, BOLD:AAG2405, ASHYD3164-14; **Taxon:** scientificName: Calolydella tenebrosa; phylum: Arthropoda; class: Insecta; order: Diptera; family: Tachinidae; genus: Calolydella; specificEpithet: tenebrosa; scientificNameAuthorship: Fleming & Wood, 2016; **Location:** continent: Central America; country: Costa Rica; countryCode: CR; stateProvince: Guanacaste; county: Sector Pitilla; locality: Area de Conservacion Guanacaste; verbatimLocality: Medrano; verbatimElevation: 380; verbatimLatitude: 11.016; verbatimLongitude: -85.3805; verbatimCoordinateSystem: Decimal; **Identification:** identifiedBy: AJ Fleming; dateIdentified: 2016; **Event:** samplingProtocol: Reared from the larvae of the geometrid moth, Semaeopus Janzen08; verbatimEventDate: 27-Nov-2013; **Record Level:** language: en; institutionCode: CNC; collectionCode: Insects; basisOfRecord: Pinned Specimen

#### Description

**Male** (Fig. 34a, b, c). Length: 8mm. **Head** (Fig. 34b): frontal setae extending beyond base of postpedicel; fronto-orbital plate gold, sparsely setulose throughout; parafacial color all silver; or mostly gold (75-90%); palps black. **Thorax** (Fig. 34a, c): gold on both dorsal and lateral surfaces; with four regular thoracic vittae; postpronotum with three setae; 3:3 acrostichal setae; 3:3 dorsocentral setae; 2:3 intra-alar setae; 2:3 supra-alar setae; three katepisternal setae; anatergite bare; scutellar discal setae slightly closer together than subapical scutellar setae, or absent. Wing vein R_4+5_ with at most 2–3 setulae dorsally at base, not extending to crossvein R-M. **Abdomen** (Fig. 34a): ground color dark brown-orange, with uninterrupted transverse marginal pollinose bands and T5 black apically; pollinosity gold dorsally, silver ventrally; base of ST1+2 black lateroventrally; T3 with one pair of median marginal setae and two pairs of discal setae; T4 with two pairs of discal setae. **Terminalia**: not examined.

**Female** (Fig. 34d, e, f). Length: 6mm. As male, except for the following characters: fronto-orbital plate 1.41X as wide as in male; palps dark grayish-orange.

#### Diagnosis

*Calolydella tenebrosa* can be distinguished from all other species of *Calolydella* by the following combination of characters: frontal setae extending beyond base of postpedicel, palps black in males and dark grayish-orange in females, abdominal ground color dark brown-orange, anatergite bare, and abdominal T5 black apically in both sexes.

#### Etymology

The specific epithet *Calolydella tenebrosa* is derived from the Latin adjective "*tenebrosum*", meaning black or dark, in reference to the dark-colored palpi and dark-colored legs, character states found in both sexes of this species.

#### Distribution

Costa Rica, ACG, Alajuela and Guanacaste provinces, 320–380m.

#### Ecology

*Calolydella tenebrosa* has been reared once from *Cyclophora* Janzen14 and once from *Semaeopus* Janzen08 (Lepidoptera: Geometridae), in rain forest.

### Calolydella timjamesi

Fleming & Wood sp. n.

urn:lsid:zoobank.org:act:19044401-2286-439E-B788-B906DED6FBD5

#### Materials

- **Type status:** Holotype. **Occurrence:** occurrenceDetails: http://janzen.sas.upenn.edu; catalogNumber: DHJPAR0017781; recordedBy: D.H. Janzen, W. Hallwachs & Freddy Quesada; individualID: DHJPAR0017781; individualCount: 1; sex: M; lifeStage: adult; preparations: pinned; otherCatalogNumbers: 99-SRNP-12851, BOLD:AAA1932, ASTAR492-07; **Taxon:** scientificName: Calolydella timjamesi; phylum: Arthropoda; class: Insecta; order: Diptera; family: Tachinidae; genus: Calolydella; specificEpithet: timjamesi; scientificNameAuthorship: Fleming & Wood, 2016; **Location:** continent: Central America; country: Costa Rica; countryCode: CR; stateProvince: Alajuela; county: Sector San Cristobal; locality: Area de Conservacion Guanacaste; verbatimLocality: Sendero Corredor; verbatimElevation: 620; verbatimLatitude: 10.8787; verbatimLongitude: -85.3896; verbatimCoordinateSystem: Decimal; **Identification:** identifiedBy: AJ Fleming; dateIdentified: 2016; **Event:** samplingProtocol: Reared from the larva of the notodontid moth, Dunama jessiehillae; verbatimEventDate: 09-Dec-1999; **Record Level:** language: en; institutionCode: CNC; collectionCode: Insects; basisOfRecord: Pinned Specimen
- **Type status:** Paratype. **Occurrence:** occurrenceDetails: http://janzen.sas.upenn.edu; catalogNumber: DHJPAR0016130; recordedBy: D.H. Janzen, W. Hallwachs & Petrona Rios; individualID: DHJPAR0016130; individualCount: 1; sex: M; lifeStage: adult; preparations: pinned; otherCatalogNumbers: 06-SRNP-33809, BOLD:AAA1932, ASTAP159-06; **Taxon:** scientificName: Calolydella timjamesi; phylum: Arthropoda; class: Insecta; order: Diptera; family: Tachinidae; genus: Calolydella; specificEpithet: timjamesi; scientificNameAuthorship: Fleming & Wood, 2016; **Location:** continent: Central America; country: Costa Rica; countryCode: CR; stateProvince: Guanacaste; county: Sector Pitilla; locality: Area de Conservacion Guanacaste; verbatimLocality: Sendero Laguna; verbatimElevation: 680; verbatimLatitude: 10.9888; verbatimLongitude: -85.4234; verbatimCoordinateSystem: Decimal; **Identification:** identifiedBy: AJ Fleming; dateIdentified: 2016; **Event:** samplingProtocol: Reared from the larva of the notodontid moth, Tithraustes lambertae; verbatimEventDate: 11-Sep-2006; **Record Level:** language: en; institutionCode: CNC; collectionCode: Insects; basisOfRecord: Pinned Specimen
- **Type status:** Paratype. **Occurrence:** occurrenceDetails: http://janzen.sas.upenn.edu; catalogNumber: DHJPAR0016143; recordedBy: D.H. Janzen, W. Hallwachs & Calixto Moraga; individualID: DHJPAR0016143; individualCount: 1; sex: F; lifeStage: adult; preparations: pinned; otherCatalogNumbers: 06-SRNP-33843, BOLD:AAA1932, ASTAP172-06; **Taxon:** scientificName: Calolydella timjamesi; phylum: Arthropoda; class: Insecta; order: Diptera; family: Tachinidae; genus: Calolydella; specificEpithet: timjamesi; scientificNameAuthorship: Fleming & Wood, 2016; **Location:** continent: Central America; country: Costa Rica; countryCode: CR; stateProvince: Guanacaste; county: Sector Pitilla; locality: Area de Conservacion Guanacaste; verbatimLocality: Sendero Laguna; verbatimElevation: 680; verbatimLatitude: 10.9888; verbatimLongitude: -85.4234; verbatimCoordinateSystem: Decimal; **Identification:** identifiedBy: AJ Fleming; dateIdentified: 2016; **Event:** samplingProtocol: Reared from the larva of the notodontid moth, Dunama janewaldronae; verbatimEventDate: 19-Sep-2006; **Record Level:** language: en; institutionCode: CNC; collectionCode: Insects; basisOfRecord: Pinned Specimen
- **Type status:** Paratype. **Occurrence:** occurrenceDetails: http://janzen.sas.upenn.edu; catalogNumber: DHJPAR0036611; recordedBy: D.H. Janzen, W. Hallwachs & Calixto Moraga; individualID: DHJPAR0036611; individualCount: 1; sex: F; lifeStage: adult; preparations: pinned; otherCatalogNumbers: 09-SRNP-32524, BOLD:AAA1932, ASHYE1522-09; **Taxon:** scientificName: Calolydella timjamesi; phylum: Arthropoda; class: Insecta; order: Diptera; family: Tachinidae; genus: Calolydella; specificEpithet: timjamesi; scientificNameAuthorship: Fleming & Wood, 2016; **Location:** continent: Central America; country: Costa Rica; countryCode: CR; stateProvince: Guanacaste; county: Sector Pitilla; locality: Area de Conservacion Guanacaste; verbatimLocality: Sendero Rotulo; verbatimElevation: 510; verbatimLatitude: 11.0135; verbatimLongitude: -85.4241; verbatimCoordinateSystem: Decimal; **Identification:** identifiedBy: AJ Fleming; dateIdentified: 2016; **Event:** samplingProtocol: Reared from the larva of the notodontid moth, Dioptis longipennis; verbatimEventDate: 16-Sep-2009; **Record Level:** language: en; institutionCode: CNC; collectionCode: Insects; basisOfRecord: Pinned Specimen
- **Type status:** Paratype. **Occurrence:** occurrenceDetails: http://janzen.sas.upenn.edu; catalogNumber: DHJPAR0016132; recordedBy: D.H. Janzen, W. Hallwachs & Calixto Moraga; individualID: DHJPAR0016132; individualCount: 1; sex: F; lifeStage: adult; preparations: pinned; otherCatalogNumbers: 06-SRNP-33839, BOLD:AAA1932, ASTAP161-06; **Taxon:** scientificName: Calolydella timjamesi; phylum: Arthropoda; class: Insecta; order: Diptera; family: Tachinidae; genus: Calolydella; specificEpithet: timjamesi; scientificNameAuthorship: Fleming & Wood, 2016; **Location:** continent: Central America; country: Costa Rica; countryCode: CR; stateProvince: Guanacaste; county: Sector Pitilla; locality: Area de Conservacion Guanacaste; verbatimLocality: Sendero Laguna; verbatimElevation: 680; verbatimLatitude: 10.9888; verbatimLongitude: -85.4234; verbatimCoordinateSystem: Decimal; **Identification:** identifiedBy: AJ Fleming; dateIdentified: 2016; **Event:** samplingProtocol: Reared from the larva of the notodontid moth, Dunama janewaldronae; verbatimEventDate: 25-Sep-2006; **Record Level:** language: en; institutionCode: CNC; collectionCode: Insects; basisOfRecord: Pinned Specimen
- **Type status:** Paratype. **Occurrence:** occurrenceDetails: http://janzen.sas.upenn.edu; catalogNumber: DHJPAR0044914; recordedBy: D.H. Janzen, W. Hallwachs & Freddy Quesada; individualID: DHJPAR0044914; individualCount: 1; sex: F; lifeStage: adult; preparations: pinned; otherCatalogNumbers: 11-SRNP-71657, BOLD:AAA1932, ACGAZ138-11; **Taxon:** scientificName: Calolydella timjamesi; phylum: Arthropoda; class: Insecta; order: Diptera; family: Tachinidae; genus: Calolydella; specificEpithet: timjamesi; scientificNameAuthorship: Fleming & Wood, 2016; **Location:** continent: Central America; country: Costa Rica; countryCode: CR; stateProvince: Guanacaste; county: Sector Pitilla; locality: Area de Conservacion Guanacaste; verbatimLocality: Charia; verbatimElevation: 530; verbatimLatitude: 10.9934; verbatimLongitude: -85.4027; verbatimCoordinateSystem: Decimal; **Identification:** identifiedBy: AJ Fleming; dateIdentified: 2016; **Event:** samplingProtocol: Reared from the larva of the notodontid moth, Dottia viridifusca; verbatimEventDate: 26-Aug-2011; **Record Level:** language: en; institutionCode: CNC; collectionCode: Insects; basisOfRecord: Pinned Specimen
- **Type status:** Paratype. **Occurrence:** occurrenceDetails: http://janzen.sas.upenn.edu; catalogNumber: DHJPAR0017778; recordedBy: D.H. Janzen, W. Hallwachs & Freddy Quesada; individualID: DHJPAR0017778; individualCount: 1; sex: F; lifeStage: adult; preparations: pinned; otherCatalogNumbers: 99-SRNP-12846, BOLD:AAA1932, ASTAR489-07; **Taxon:** scientificName: Calolydella timjamesi; phylum: Arthropoda; class: Insecta; order: Diptera; family: Tachinidae; genus: Calolydella; specificEpithet: timjamesi; scientificNameAuthorship: Fleming & Wood, 2016; **Location:** continent: Central America; country: Costa Rica; countryCode: CR; stateProvince: Alajuela; county: Sector San Cristobal; locality: Area de Conservacion Guanacaste; verbatimLocality: Sendero Corredor; verbatimElevation: 620; verbatimLatitude: 10.8787; verbatimLongitude: -85.3896; verbatimCoordinateSystem: Decimal; **Identification:** identifiedBy: AJ Fleming; dateIdentified: 2016; **Event:** samplingProtocol: Reared from the larva of the notodontid moth, Dunama jessiehillae; verbatimEventDate: 09-Nov-1999; **Record Level:** language: en; institutionCode: CNC; collectionCode: Insects; basisOfRecord: Pinned Specimen
- **Type status:** Paratype. **Occurrence:** occurrenceDetails: http://janzen.sas.upenn.edu; catalogNumber: DHJPAR0053460; recordedBy: D.H. Janzen, W. Hallwachs & Elda Araya; individualID: DHJPAR0053460; individualCount: 1; sex: F; lifeStage: adult; preparations: pinned; otherCatalogNumbers: 13-SRNP-4277, BOLD:AAA1932, ASHYM2814-13; **Taxon:** scientificName: Calolydella timjamesi; phylum: Arthropoda; class: Insecta; order: Diptera; family: Tachinidae; genus: Calolydella; specificEpithet: timjamesi; scientificNameAuthorship: Fleming & Wood, 2016; **Location:** continent: Central America; country: Costa Rica; countryCode: CR; stateProvince: Alajuela; county: Sector San Cristobal; locality: Area de Conservacion Guanacaste; verbatimLocality: Rio Blanco Abajo; verbatimElevation: 500; verbatimLatitude: 10.9004; verbatimLongitude: -85.3725; verbatimCoordinateSystem: Decimal; **Identification:** identifiedBy: AJ Fleming; dateIdentified: 2016; **Event:** samplingProtocol: Reared from the larva of the notodontid moth, Tithraustes noctilucesICG02; verbatimEventDate: 06-Sep-2013; **Record Level:** language: en; institutionCode: CNC; collectionCode: Insects; basisOfRecord: Pinned Specimen
- **Type status:** Paratype. **Occurrence:** occurrenceDetails: http://janzen.sas.upenn.edu; catalogNumber: DHJPAR0035882; recordedBy: D.H. Janzen, W. Hallwachs & Manuel Rios; individualID: DHJPAR0035882; individualCount: 1; sex: F; lifeStage: adult; preparations: pinned; otherCatalogNumbers: 09-SRNP-31957, BOLD:AAA1932, ASHYD1263-09; **Taxon:** scientificName: Calolydella timjamesi; phylum: Arthropoda; class: Insecta; order: Diptera; family: Tachinidae; genus: Calolydella; specificEpithet: timjamesi; scientificNameAuthorship: Fleming & Wood, 2016; **Location:** continent: Central America; country: Costa Rica; countryCode: CR; stateProvince: Guanacaste; county: Sector Pitilla; locality: Area de Conservacion Guanacaste; verbatimLocality: Sendero Memos; verbatimElevation: 740; verbatimLatitude: 10.9817; verbatimLongitude: -85.4278; verbatimCoordinateSystem: Decimal; **Identification:** identifiedBy: AJ Fleming; dateIdentified: 2016; **Event:** samplingProtocol: Reared from the larva of the notodontid moth, Dunama jessiehillae; verbatimEventDate: 11-Jul-2009; **Record Level:** language: en; institutionCode: CNC; collectionCode: Insects; basisOfRecord: Pinned Specimen
- **Type status:** Paratype. **Occurrence:** occurrenceDetails: http://janzen.sas.upenn.edu; catalogNumber: DHJPAR0016152; recordedBy: D.H. Janzen, W. Hallwachs & Petrona Rios; individualID: DHJPAR0016152; individualCount: 1; sex: F; lifeStage: adult; preparations: pinned; otherCatalogNumbers: 06-SRNP-33804, BOLD:AAA1932, ASTAP181-06; **Taxon:** scientificName: Calolydella timjamesi; phylum: Arthropoda; class: Insecta; order: Diptera; family: Tachinidae; genus: Calolydella; specificEpithet: timjamesi; scientificNameAuthorship: Fleming & Wood, 2016; **Location:** continent: Central America; country: Costa Rica; countryCode: CR; stateProvince: Guanacaste; county: Sector Pitilla; locality: Area de Conservacion Guanacaste; verbatimLocality: Sendero Laguna; verbatimElevation: 680; verbatimLatitude: 10.9888; verbatimLongitude: -85.4234; verbatimCoordinateSystem: Decimal; **Identification:** identifiedBy: AJ Fleming; dateIdentified: 2016; **Event:** samplingProtocol: Reared from the larva of the notodontid moth, Tithraustes lambertae; verbatimEventDate: 21-Sep-2006; **Record Level:** language: en; institutionCode: CNC; collectionCode: Insects; basisOfRecord: Pinned Specimen
- **Type status:** Paratype. **Occurrence:** occurrenceDetails: http://janzen.sas.upenn.edu; catalogNumber: DHJPAR0017783; recordedBy: D.H. Janzen, W. Hallwachs & Freddy Quesada; individualID: DHJPAR0017783; individualCount: 1; sex: M; lifeStage: adult; preparations: pinned; otherCatalogNumbers: 99-SRNP-5596, BOLD:AAA1932, ASTAR494-07; **Taxon:** scientificName: Calolydella timjamesi; phylum: Arthropoda; class: Insecta; order: Diptera; family: Tachinidae; genus: Calolydella; specificEpithet: timjamesi; scientificNameAuthorship: Fleming & Wood, 2016; **Location:** continent: Central America; country: Costa Rica; countryCode: CR; stateProvince: Alajuela; county: Sector San Cristobal; locality: Area de Conservacion Guanacaste; verbatimLocality: Sendero Carmona; verbatimElevation: 670; verbatimLatitude: 10.8762; verbatimLongitude: -85.3863; verbatimCoordinateSystem: Decimal; **Identification:** identifiedBy: AJ Fleming; dateIdentified: 2016; **Event:** samplingProtocol: Reared from the larva of the notodontid moth, Dunama jessiehillae; verbatimEventDate: 07-Mar-1999; **Record Level:** language: en; institutionCode: CNC; collectionCode: Insects; basisOfRecord: Pinned Specimen
- **Type status:** Paratype. **Occurrence:** occurrenceDetails: http://janzen.sas.upenn.edu; catalogNumber: DHJPAR0017780; recordedBy: D.H. Janzen, W. Hallwachs & Freddy Quesada; individualID: DHJPAR0017780; individualCount: 1; sex: F; lifeStage: adult; preparations: pinned; otherCatalogNumbers: 99-SRNP-12853, BOLD:AAA1932, ASTAR491-07; **Taxon:** scientificName: Calolydella timjamesi; phylum: Arthropoda; class: Insecta; order: Diptera; family: Tachinidae; genus: Calolydella; specificEpithet: timjamesi; scientificNameAuthorship: Fleming & Wood, 2016; **Location:** continent: Central America; country: Costa Rica; countryCode: CR; stateProvince: Alajuela; county: Sector San Cristobal; locality: Area de Conservacion Guanacaste; verbatimLocality: Sendero Corredor; verbatimElevation: 620; verbatimLatitude: 10.8787; verbatimLongitude: -85.3896; verbatimCoordinateSystem: Decimal; **Identification:** identifiedBy: AJ Fleming; dateIdentified: 2016; **Event:** samplingProtocol: Reared from the larva of the notodontid moth, Dunama jessiehillae; verbatimEventDate: 09-Dec-1999; **Record Level:** language: en; institutionCode: CNC; collectionCode: Insects; basisOfRecord: Pinned Specimen
- **Type status:** Paratype. **Occurrence:** occurrenceDetails: http://janzen.sas.upenn.edu; catalogNumber: DHJPAR0016139; recordedBy: D.H. Janzen, W. Hallwachs & Calixto Moraga; individualID: DHJPAR0016139; individualCount: 1; sex: M; lifeStage: adult; preparations: pinned; otherCatalogNumbers: 06-SRNP-33840, BOLD:AAA1932, ASTAP168-06; **Taxon:** scientificName: Calolydella timjamesi; phylum: Arthropoda; class: Insecta; order: Diptera; family: Tachinidae; genus: Calolydella; specificEpithet: timjamesi; scientificNameAuthorship: Fleming & Wood, 2016; **Location:** continent: Central America; country: Costa Rica; countryCode: CR; stateProvince: Guanacaste; county: Sector Pitilla; locality: Area de Conservacion Guanacaste; verbatimLocality: Sendero Laguna; verbatimElevation: 680; verbatimLatitude: 10.9888; verbatimLongitude: -85.4234; verbatimCoordinateSystem: Decimal; **Identification:** identifiedBy: AJ Fleming; dateIdentified: 2016; **Event:** samplingProtocol: Reared from the larva of the notodontid moth, Dunama janewaldronae; verbatimEventDate: 19-Sep-2006; **Record Level:** language: en; institutionCode: CNC; collectionCode: Insects; basisOfRecord: Pinned Specimen
- **Type status:** Paratype. **Occurrence:** occurrenceDetails: http://janzen.sas.upenn.edu; catalogNumber: DHJPAR0011690; recordedBy: D.H. Janzen, W. Hallwachs & Yessenia Mendoza; individualID: DHJPAR0011690; individualCount: 1; sex: F; lifeStage: adult; preparations: pinned; otherCatalogNumbers: 04-SRNP-3964, BOLD:AAA1932, ASTAS416-06; **Taxon:** scientificName: Calolydella timjamesi; phylum: Arthropoda; class: Insecta; order: Diptera; family: Tachinidae; genus: Calolydella; specificEpithet: timjamesi; scientificNameAuthorship: Fleming & Wood, 2016; **Location:** continent: Central America; country: Costa Rica; countryCode: CR; stateProvince: Alajuela; county: Sector San Cristobal; locality: Area de Conservacion Guanacaste; verbatimLocality: Sendero Corredor; verbatimElevation: 620; verbatimLatitude: 10.8787; verbatimLongitude: -85.3896; verbatimCoordinateSystem: Decimal; **Identification:** identifiedBy: AJ Fleming; dateIdentified: 2016; **Event:** samplingProtocol: Reared from the larva of the notodontid moth, Dottia viridifusca; verbatimEventDate: 05-Sep-2004; **Record Level:** language: en; institutionCode: CNC; collectionCode: Insects; basisOfRecord: Pinned Specimen
- **Type status:** Paratype. **Occurrence:** occurrenceDetails: http://janzen.sas.upenn.edu; catalogNumber: DHJPAR0016137; recordedBy: D.H. Janzen, W. Hallwachs & Petrona Rios; individualID: DHJPAR0016137; individualCount: 1; sex: F; lifeStage: adult; preparations: pinned; otherCatalogNumbers: 06-SRNP-33659, BOLD:AAA1932, ASTAP166-06; **Taxon:** scientificName: Calolydella timjamesi; phylum: Arthropoda; class: Insecta; order: Diptera; family: Tachinidae; genus: Calolydella; specificEpithet: timjamesi; scientificNameAuthorship: Fleming & Wood, 2016; **Location:** continent: Central America; country: Costa Rica; countryCode: CR; stateProvince: Guanacaste; county: Sector Pitilla; locality: Area de Conservacion Guanacaste; verbatimLocality: Sendero Evangelista; verbatimElevation: 660; verbatimLatitude: 10.9868; verbatimLongitude: -85.4208; verbatimCoordinateSystem: Decimal; **Identification:** identifiedBy: AJ Fleming; dateIdentified: 2016; **Event:** samplingProtocol: Reared from the larva of the notodontid moth, Dioptis longipennis; verbatimEventDate: 07-Sep-2006; **Record Level:** language: en; institutionCode: CNC; collectionCode: Insects; basisOfRecord: Pinned Specimen
- **Type status:** Paratype. **Occurrence:** occurrenceDetails: http://janzen.sas.upenn.edu; catalogNumber: DHJPAR0016129; recordedBy: D.H. Janzen, W. Hallwachs & Calixto Moraga; individualID: DHJPAR0016129; individualCount: 1; sex: F; lifeStage: adult; preparations: pinned; otherCatalogNumbers: 06-SRNP-33813, BOLD:AAA1932, ASTAP158-06; **Taxon:** scientificName: Calolydella timjamesi; phylum: Arthropoda; class: Insecta; order: Diptera; family: Tachinidae; genus: Calolydella; specificEpithet: timjamesi; scientificNameAuthorship: Fleming & Wood, 2016; **Location:** continent: Central America; country: Costa Rica; countryCode: CR; stateProvince: Guanacaste; county: Sector Pitilla; locality: Area de Conservacion Guanacaste; verbatimLocality: Sendero Laguna; verbatimElevation: 680; verbatimLatitude: 10.9888; verbatimLongitude: -85.4234; verbatimCoordinateSystem: Decimal; **Identification:** identifiedBy: AJ Fleming; dateIdentified: 2016; **Event:** samplingProtocol: Reared from the larva of the notodontid moth, Tithraustes lambertae; verbatimEventDate: 25-Sep-2006; **Record Level:** language: en; institutionCode: CNC; collectionCode: Insects; basisOfRecord: Pinned Specimen
- **Type status:** Paratype. **Occurrence:** occurrenceDetails: http://janzen.sas.upenn.edu; catalogNumber: DHJPAR0017779; recordedBy: D.H. Janzen, W. Hallwachs & Freddy Quesada; individualID: DHJPAR0017779; individualCount: 1; sex: M; lifeStage: adult; preparations: pinned; otherCatalogNumbers: 99-SRNP-12844, BOLD:AAA1932, ASTAR490-07; **Taxon:** scientificName: Calolydella timjamesi; phylum: Arthropoda; class: Insecta; order: Diptera; family: Tachinidae; genus: Calolydella; specificEpithet: timjamesi; scientificNameAuthorship: Fleming & Wood, 2016; **Location:** continent: Central America; country: Costa Rica; countryCode: CR; stateProvince: Alajuela; county: Sector San Cristobal; locality: Area de Conservacion Guanacaste; verbatimLocality: Sendero Corredor; verbatimElevation: 620; verbatimLatitude: 10.8787; verbatimLongitude: -85.3896; verbatimCoordinateSystem: Decimal; **Identification:** identifiedBy: AJ Fleming; dateIdentified: 2016; **Event:** samplingProtocol: Reared from the larva of the notodontid moth, Dunama jessiehillae; verbatimEventDate: 09-Oct-1999; **Record Level:** language: en; institutionCode: CNC; collectionCode: Insects; basisOfRecord: Pinned Specimen
- **Type status:** Paratype. **Occurrence:** occurrenceDetails: http://janzen.sas.upenn.edu; catalogNumber: DHJPAR0017772; recordedBy: D.H. Janzen, W. Hallwachs & Carolina Cano; individualID: DHJPAR0017772; individualCount: 1; sex: M; lifeStage: adult; preparations: pinned; otherCatalogNumbers: 03-SRNP-8648, BOLD:AAA1932, ASTAR483-07; **Taxon:** scientificName: Calolydella timjamesi; phylum: Arthropoda; class: Insecta; order: Diptera; family: Tachinidae; genus: Calolydella; specificEpithet: timjamesi; scientificNameAuthorship: Fleming & Wood, 2016; **Location:** continent: Central America; country: Costa Rica; countryCode: CR; stateProvince: Alajuela; county: Sector San Cristobal; locality: Area de Conservacion Guanacaste; verbatimLocality: Sendero Corredor; verbatimElevation: 620; verbatimLatitude: 10.8787; verbatimLongitude: -85.3896; verbatimCoordinateSystem: Decimal; **Identification:** identifiedBy: AJ Fleming; dateIdentified: 2016; **Event:** samplingProtocol: Reared from the larva of the notodontid moth, Dunama jessiehillae; verbatimEventDate: 19-Oct-2003; **Record Level:** language: en; institutionCode: CNC; collectionCode: Insects; basisOfRecord: Pinned Specimen
- **Type status:** Paratype. **Occurrence:** occurrenceDetails: http://janzen.sas.upenn.edu; catalogNumber: DHJPAR0017777; recordedBy: D.H. Janzen, W. Hallwachs & Freddy Quesada; individualID: DHJPAR0017777; individualCount: 1; sex: M; lifeStage: adult; preparations: pinned; otherCatalogNumbers: 99-SRNP-12847, BOLD:AAA1932, ASTAR488-07; **Taxon:** scientificName: Calolydella timjamesi; phylum: Arthropoda; class: Insecta; order: Diptera; family: Tachinidae; genus: Calolydella; specificEpithet: timjamesi; scientificNameAuthorship: Fleming & Wood, 2016; **Location:** continent: Central America; country: Costa Rica; countryCode: CR; stateProvince: Alajuela; county: Sector San Cristobal; locality: Area de Conservacion Guanacaste; verbatimLocality: Sendero Corredor; verbatimElevation: 620; verbatimLatitude: 10.8787; verbatimLongitude: -85.3896; verbatimCoordinateSystem: Decimal; **Identification:** identifiedBy: AJ Fleming; dateIdentified: 2016; **Event:** samplingProtocol: Reared from the larva of the notodontid moth, Dunama jessiehillae; verbatimEventDate: 09-Dec-1999; **Record Level:** language: en; institutionCode: CNC; collectionCode: Insects; basisOfRecord: Pinned Specimen
- **Type status:** Paratype. **Occurrence:** occurrenceDetails: http://janzen.sas.upenn.edu; catalogNumber: DHJPAR0007021; recordedBy: D.H. Janzen, W. Hallwachs & Manuel Rios; individualID: DHJPAR0007021; individualCount: 1; sex: M; lifeStage: adult; preparations: pinned; otherCatalogNumbers: 05-SRNP-70526, BOLD:AAA1932, ASTAV263-06; **Taxon:** scientificName: Calolydella timjamesi; phylum: Arthropoda; class: Insecta; order: Diptera; family: Tachinidae; genus: Calolydella; specificEpithet: timjamesi; scientificNameAuthorship: Fleming & Wood, 2016; **Location:** continent: Central America; country: Costa Rica; countryCode: CR; stateProvince: Guanacaste; county: Sector Pitilla; locality: Area de Conservacion Guanacaste; verbatimLocality: Colocho; verbatimElevation: 375; verbatimLatitude: 11.0237; verbatimLongitude: -85.4188; verbatimCoordinateSystem: Decimal; **Identification:** identifiedBy: AJ Fleming; dateIdentified: 2016; **Event:** samplingProtocol: Reared from the larva of the notodontid moth, Dunama jessiehillae; verbatimEventDate: 05-Feb-2006; **Record Level:** language: en; institutionCode: CNC; collectionCode: Insects; basisOfRecord: Pinned Specimen
- **Type status:** Paratype. **Occurrence:** occurrenceDetails: http://janzen.sas.upenn.edu; catalogNumber: DHJPAR0016128; recordedBy: D.H. Janzen, W. Hallwachs & Calixto Moraga; individualID: DHJPAR0016128; individualCount: 1; sex: F; lifeStage: adult; preparations: pinned; otherCatalogNumbers: 06-SRNP-33929, BOLD:AAA1932, ASTAP157-06; **Taxon:** scientificName: Calolydella timjamesi; phylum: Arthropoda; class: Insecta; order: Diptera; family: Tachinidae; genus: Calolydella; specificEpithet: timjamesi; scientificNameAuthorship: Fleming & Wood, 2016; **Location:** continent: Central America; country: Costa Rica; countryCode: CR; stateProvince: Guanacaste; county: Sector Pitilla; locality: Area de Conservacion Guanacaste; verbatimLocality: Sendero Laguna; verbatimElevation: 680; verbatimLatitude: 10.9888; verbatimLongitude: -85.4234; verbatimCoordinateSystem: Decimal; **Identification:** identifiedBy: AJ Fleming; dateIdentified: 2016; **Event:** samplingProtocol: Reared from the larva of the notodontid moth, Dunama jessiehillae; verbatimEventDate: 25-Sep-2006; **Record Level:** language: en; institutionCode: CNC; collectionCode: Insects; basisOfRecord: Pinned Specimen
- **Type status:** Paratype. **Occurrence:** occurrenceDetails: http://janzen.sas.upenn.edu; catalogNumber: DHJPAR0016576; recordedBy: D.H. Janzen, W. Hallwachs & Calixto Moraga; individualID: DHJPAR0016576; individualCount: 1; sex: F; lifeStage: adult; preparations: pinned; otherCatalogNumbers: 06-SRNP-34841, BOLD:AAA1932, ASTAP780-07; **Taxon:** scientificName: Calolydella timjamesi; phylum: Arthropoda; class: Insecta; order: Diptera; family: Tachinidae; genus: Calolydella; specificEpithet: timjamesi; scientificNameAuthorship: Fleming & Wood, 2016; **Location:** continent: Central America; country: Costa Rica; countryCode: CR; stateProvince: Guanacaste; county: Sector Pitilla; locality: Area de Conservacion Guanacaste; verbatimLocality: Sendero Laguna; verbatimElevation: 680; verbatimLatitude: 10.9888; verbatimLongitude: -85.4234; verbatimCoordinateSystem: Decimal; **Identification:** identifiedBy: AJ Fleming; dateIdentified: 2016; **Event:** samplingProtocol: Reared from the larva of the notodontid moth, Tithraustes lambertae; verbatimEventDate: 10-Nov-2006; **Record Level:** language: en; institutionCode: CNC; collectionCode: Insects; basisOfRecord: Pinned Specimen
- **Type status:** Paratype. **Occurrence:** occurrenceDetails: http://janzen.sas.upenn.edu; catalogNumber: DHJPAR0036621; recordedBy: D.H. Janzen, W. Hallwachs & Petrona Rios; individualID: DHJPAR0036621; individualCount: 1; sex: M; lifeStage: adult; preparations: pinned; otherCatalogNumbers: 09-SRNP-32404, BOLD:AAA1932, ASHYE1532-09; **Taxon:** scientificName: Calolydella timjamesi; phylum: Arthropoda; class: Insecta; order: Diptera; family: Tachinidae; genus: Calolydella; specificEpithet: timjamesi; scientificNameAuthorship: Fleming & Wood, 2016; **Location:** continent: Central America; country: Costa Rica; countryCode: CR; stateProvince: Guanacaste; county: Sector Pitilla; locality: Area de Conservacion Guanacaste; verbatimLocality: Sendero Cuestona; verbatimElevation: 640; verbatimLatitude: 10.9945; verbatimLongitude: -85.4146; verbatimCoordinateSystem: Decimal; **Identification:** identifiedBy: AJ Fleming; dateIdentified: 2016; **Event:** samplingProtocol: Reared from the larva of the notodontid moth, Dioptis longipennis; verbatimEventDate: 15-Sep-2009; **Record Level:** language: en; institutionCode: CNC; collectionCode: Insects; basisOfRecord: Pinned Specimen
- **Type status:** Paratype. **Occurrence:** occurrenceDetails: http://janzen.sas.upenn.edu; catalogNumber: DHJPAR0016131; recordedBy: D.H. Janzen, W. Hallwachs & Calixto Moraga; individualID: DHJPAR0016131; individualCount: 1; sex: F; lifeStage: adult; preparations: pinned; otherCatalogNumbers: 06-SRNP-33838, BOLD:AAA1932, ASTAP160-06; **Taxon:** scientificName: Calolydella timjamesi; phylum: Arthropoda; class: Insecta; order: Diptera; family: Tachinidae; genus: Calolydella; specificEpithet: timjamesi; scientificNameAuthorship: Fleming & Wood, 2016; **Location:** continent: Central America; country: Costa Rica; countryCode: CR; stateProvince: Guanacaste; county: Sector Pitilla; locality: Area de Conservacion Guanacaste; verbatimLocality: Sendero Laguna; verbatimElevation: 680; verbatimLatitude: 10.9888; verbatimLongitude: -85.4234; verbatimCoordinateSystem: Decimal; **Identification:** identifiedBy: AJ Fleming; dateIdentified: 2016; **Event:** samplingProtocol: Reared from the larva of the notodontid moth, Dunama janewaldronae; verbatimEventDate: 21-Sep-2006; **Record Level:** language: en; institutionCode: CNC; collectionCode: Insects; basisOfRecord: Pinned Specimen
- **Type status:** Paratype. **Occurrence:** occurrenceDetails: http://janzen.sas.upenn.edu; catalogNumber: DHJPAR0027866; recordedBy: D.H. Janzen, W. Hallwachs & Jose Perez; individualID: DHJPAR0027866; individualCount: 1; sex: F; lifeStage: adult; preparations: pinned; otherCatalogNumbers: 08-SRNP-41163, BOLD:AAA1932, ASHYE103-08; **Taxon:** scientificName: Calolydella timjamesi; phylum: Arthropoda; class: Insecta; order: Diptera; family: Tachinidae; genus: Calolydella; specificEpithet: timjamesi; scientificNameAuthorship: Fleming & Wood, 2016; **Location:** continent: Central America; country: Costa Rica; countryCode: CR; stateProvince: Alajuela; county: Sector Rincon Rain Forest; locality: Area de Conservacion Guanacaste; verbatimLocality: Sendero Anonas; verbatimElevation: 405; verbatimLatitude: 10.9053; verbatimLongitude: -85.2788; verbatimCoordinateSystem: Decimal; **Identification:** identifiedBy: AJ Fleming; dateIdentified: 2016; **Event:** samplingProtocol: Reared from the larva of the notodontid moth, Dunama jessiehillae; verbatimEventDate: 30-Jun-2008; **Record Level:** language: en; institutionCode: CNC; collectionCode: Insects; basisOfRecord: Pinned Specimen
- **Type status:** Paratype. **Occurrence:** occurrenceDetails: http://janzen.sas.upenn.edu; catalogNumber: DHJPAR0016192; recordedBy: D.H. Janzen, W. Hallwachs & Roster Moraga; individualID: DHJPAR0016192; individualCount: 1; sex: F; lifeStage: adult; preparations: pinned; otherCatalogNumbers: 06-SRNP-22485, BOLD:AAA1932, ASTAP221-06; **Taxon:** scientificName: Calolydella timjamesi; phylum: Arthropoda; class: Insecta; order: Diptera; family: Tachinidae; genus: Calolydella; specificEpithet: timjamesi; scientificNameAuthorship: Fleming & Wood, 2016; **Location:** continent: Central America; country: Costa Rica; countryCode: CR; stateProvince: Guanacaste; county: Sector Del Oro; locality: Area de Conservacion Guanacaste; verbatimLocality: San Antonio; verbatimElevation: 335; verbatimLatitude: 11.0353; verbatimLongitude: -85.4453; verbatimCoordinateSystem: Decimal; **Identification:** identifiedBy: AJ Fleming; dateIdentified: 2016; **Event:** samplingProtocol: Reared from the larva of the notodontid moth, Dunama jessiehillae; verbatimEventDate: 13-Sep-2006; **Record Level:** language: en; institutionCode: CNC; collectionCode: Insects; basisOfRecord: Pinned Specimen

#### Description

**Male** (Fig. 35a, b, c). Length: 5–7mm. **Head** (Fig. 35b): frontal setae extending beyond base of postpedicel; fronto-orbital plate gold, sparsely setulose, these setulae being confined to the upper half; parafacial silver throughout. **Thorax** (Fig. 35a, c): gold on dorsal surface, silver laterally (>50% coverage); with four regular thoracic vittae presuturally, these fusing together postsuturally; postpronotum with two setae (inner basal seta absent); 3:3 acrostichal setae; 3:3 dorsocentral setae; 1:3 intra-alar setae; 1:3 supra-alar setae; three katepisternal setae; anatergite with three or more hair-like setae, often in a small tuft; scutellar discal setae slightly closer together than subapical scutellar setae. Wing vein R_4+5_ with 6–7 setulae dorsally from base to crossvein R-M. **Abdomen** (Fig. 35a): ground color black, with uninterrupted transverse marginal pollinose bands; abdomen gold dorsally, silver ventrally; base of ST1+2 black lateroventrally; T3 with one pair of median marginal setae and one pair of discal setae; T4 with one pair of discal setae. **Terminalia** (Fig. 36): sternite 5 (Fig. 36c) with two small lobes and a wide U-shaped median cleft; 0.47X the length of the sternite from lobe to apex; inner margin covered by dense pollinosity, appearing darker than surrounding cuticle; entire lobe of sternite with short stout setae, longer along margins of sternite. Cerci (Fig. 36b), in dorsal view, separated by a gap widening at apex; each cercus long, slender and pointed, very slightly tapered from its already narrow base; cercus slightly apically lobed, setose along its basal half. Surstylus (Fig. 36a) subequal to length of cercus, stout and spatulate, appearing rounded at apex when viewed laterally; apical half with short setulae; tip of surstylus angled (almost hooked) inwards when viewed dorsally.

**Female** (Fig. 35d, e, f). Length: 4–6mm. Fronto-orbital plate 1.73X wider than in male.

#### Diagnosis

*Calolydella timjamesi* can be distinguished from all other species of *Calolydella* by the following combination of traits: fronto-orbital plate sparsely setulose to bare, frontal setae extending beyond base of postpedicel, abdominal ground color black, anatergite with three or more setae often in a small tuft, and scutellar discal setae slightly closer togetherthan subapical scutellar setae.

#### Etymology

The specific epithet is in honor of Tim James of Levittown, Pennsylvania, in recognition of the moral and family support of Tanya Dapkey in her efforts curating and preparing ACG parasitoid flies for DNA barcoding.

#### Distribution

Costa Rica, ACG, Alajuela and Guanacaste provinces, 335–740m.

#### Ecology

*Calolydella timjamesi* has been reared 26 times from six species of Notodontidae: *Dunama janewaldronae* Chacon, 2013, *Dunama jessiehillae* Chacon, 2013, *Tithraustes* noctilucesICG02, *Tithraustes lambertae* Miller, 2008, *Dioptis longipennis* (Schaus, 1913), and *Dottia viridifusca* (Schaus, 1906), in rain forest and dry-rain lowland intergrade.

### Calolydella virginiajamesae

Fleming & Wood sp. n.

urn:lsid:zoobank.org:act:58B44ECE-D7A6-49F6-B050-D118409D499A

#### Materials

- **Type status:** Holotype. **Occurrence:** occurrenceDetails: http://janzen.sas.upenn.edu; catalogNumber: DHJPAR0017770; recordedBy: D.H. Janzen, W. Hallwachs & Gloria Sihezar; individualID: DHJPAR0017770; individualCount: 1; sex: M; lifeStage: adult; preparations: pinned; otherCatalogNumbers: 99-SRNP-13019, BOLD:ABY7242, ASTAR481-07; **Taxon:** scientificName: Calolydella virginiajamesae; phylum: Arthropoda; class: Insecta; order: Diptera; family: Tachinidae; genus: Calolydella; specificEpithet: virginiajamesae; scientificNameAuthorship: Fleming & Wood, 2016; **Location:** continent: Central America; country: Costa Rica; countryCode: CR; stateProvince: Alajuela; county: Sector San Cristobal; locality: Area de Conservacion Guanacaste; verbatimLocality: Quebrada Cementerio; verbatimElevation: 700; verbatimLatitude: 10.8712; verbatimLongitude: -85.3875; verbatimCoordinateSystem: Decimal; **Identification:** identifiedBy: AJ Fleming; dateIdentified: 2016; **Event:** samplingProtocol: Reared from the larva of the sphingid moth, Manduca sextaDHJ03; verbatimEventDate: 13-Oct-1999; **Record Level:** language: en; institutionCode: CNC; collectionCode: Insects; basisOfRecord: Pinned Specimen
- **Type status:** Paratype. **Occurrence:** occurrenceDetails: http://janzen.sas.upenn.edu; catalogNumber: DHJPAR0054010; recordedBy: D.H. Janzen, W. Hallwachs & Keiner Aragon; individualID: DHJPAR0054010; individualCount: 1; sex: M; lifeStage: adult; preparations: pinned; otherCatalogNumbers: 13-SRNP-45063, BOLD:ABY7242, ASHYD3178-14; **Taxon:** scientificName: Calolydella virginiajamesae; phylum: Arthropoda; class: Insecta; order: Diptera; family: Tachinidae; genus: Calolydella; specificEpithet: virginiajamesae; scientificNameAuthorship: Fleming & Wood, 2016; **Location:** continent: Central America; country: Costa Rica; countryCode: CR; stateProvince: Alajuela; county: Sector Rincon Rain Forest; locality: Area de Conservacion Guanacaste; verbatimLocality: Palomo; verbatimElevation: 96; verbatimLatitude: 10.9619; verbatimLongitude: -85.2804; verbatimCoordinateSystem: Decimal; **Identification:** identifiedBy: AJ Fleming; dateIdentified: 2016; **Event:** samplingProtocol: Reared from the larva of the gelechiid, gelJanzen01 Janzen11; verbatimEventDate: 03-Dec-2013; **Record Level:** language: en; institutionCode: CNC; collectionCode: Insects; basisOfRecord: Pinned Specimen
- **Type status:** Paratype. **Occurrence:** occurrenceDetails: http://janzen.sas.upenn.edu; catalogNumber: DHJPAR0034486; recordedBy: D.H. Janzen, W. Hallwachs & Duvalier Briceno; individualID: DHJPAR0034486; individualCount: 1; sex: F; lifeStage: adult; preparations: pinned; otherCatalogNumbers: 09-SRNP-65345, BOLD:ABY7242, ASHYC1138-09; **Taxon:** scientificName: Calolydella virginiajamesae; phylum: Arthropoda; class: Insecta; order: Diptera; family: Tachinidae; genus: Calolydella; specificEpithet: virginiajamesae; scientificNameAuthorship: Fleming & Wood, 2016; **Location:** continent: Central America; country: Costa Rica; countryCode: CR; stateProvince: Alajuela; county: Brasilia; locality: Area de Conservacion Guanacaste; verbatimLocality: Moga; verbatimElevation: 320; verbatimLatitude: 11.0123; verbatimLongitude: -85.3493; verbatimCoordinateSystem: Decimal; **Identification:** identifiedBy: AJ Fleming; dateIdentified: 2016; **Event:** samplingProtocol: Reared from the larva of the hesperiid moth, Saliana antoninus; verbatimEventDate: 12-Mar-2009; **Record Level:** language: en; institutionCode: CNC; collectionCode: Insects; basisOfRecord: Pinned Specimen
- **Type status:** Paratype. **Occurrence:** occurrenceDetails: http://janzen.sas.upenn.edu; catalogNumber: DHJPAR0039336; recordedBy: D.H. Janzen, W. Hallwachs & Calixto Moraga; individualID: DHJPAR0039336; individualCount: 1; sex: F; lifeStage: adult; preparations: pinned; otherCatalogNumbers: 10-SRNP-30911, BOLD:ABY7242, ASTAV899-10; **Taxon:** scientificName: Calolydella virginiajamesae; phylum: Arthropoda; class: Insecta; order: Diptera; family: Tachinidae; genus: Calolydella; specificEpithet: virginiajamesae; scientificNameAuthorship: Fleming & Wood, 2016; **Location:** continent: Central America; country: Costa Rica; countryCode: CR; stateProvince: Guanacaste; county: Sector Pitilla; locality: Area de Conservacion Guanacaste; verbatimLocality: Estacion Pitilla; verbatimElevation: 675; verbatimLatitude: 10.9893; verbatimLongitude: -85.4258; verbatimCoordinateSystem: Decimal; **Identification:** identifiedBy: AJ Fleming; dateIdentified: 2016; **Event:** samplingProtocol: Reared from the larva of the crambid moth, Herpetogramma phaeopteralis; verbatimEventDate: 26-Apr-2010; **Record Level:** language: en; institutionCode: CNC; collectionCode: Insects; basisOfRecord: Pinned Specimen
- **Type status:** Paratype. **Occurrence:** occurrenceDetails: http://janzen.sas.upenn.edu; catalogNumber: DHJPAR0053994; recordedBy: D.H. Janzen, W. Hallwachs & Ricardo Calero; individualID: DHJPAR0053994; individualCount: 1; sex: M; lifeStage: adult; preparations: pinned; otherCatalogNumbers: 13-SRNP-71701, BOLD:ABY7242, ASHYD3162-14; **Taxon:** scientificName: Calolydella virginiajamesae; phylum: Arthropoda; class: Insecta; order: Diptera; family: Tachinidae; genus: Calolydella; specificEpithet: virginiajamesae; scientificNameAuthorship: Fleming & Wood, 2016; **Location:** continent: Central America; country: Costa Rica; countryCode: CR; stateProvince: Guanacaste; county: Sector Pitilla; locality: Area de Conservacion Guanacaste; verbatimLocality: Medrano; verbatimElevation: 380; verbatimLatitude: 11.016; verbatimLongitude: -85.3805; verbatimCoordinateSystem: Decimal; **Identification:** identifiedBy: AJ Fleming; dateIdentified: 2016; **Event:** samplingProtocol: Reared from the larva of the riodinid butterfly Thisbe irenea; verbatimEventDate: 02-Nov-2013; **Record Level:** language: en; institutionCode: CNC; collectionCode: Insects; basisOfRecord: Pinned Specimen
- **Type status:** Paratype. **Occurrence:** occurrenceDetails: http://janzen.sas.upenn.edu; catalogNumber: DHJPAR0007071; recordedBy: D.H. Janzen, W. Hallwachs & Manuel Rios; individualID: DHJPAR0007071; individualCount: 1; sex: F; lifeStage: adult; preparations: pinned; otherCatalogNumbers: 06-SRNP-30377, BOLD:ABY7242, ASTAB408-10; **Taxon:** scientificName: Calolydella virginiajamesae; phylum: Arthropoda; class: Insecta; order: Diptera; family: Tachinidae; genus: Calolydella; specificEpithet: virginiajamesae; scientificNameAuthorship: Fleming & Wood, 2016; **Location:** continent: Central America; country: Costa Rica; countryCode: CR; stateProvince: Guanacaste; county: Sector Pitilla; locality: Area de Conservacion Guanacaste; verbatimLocality: Pasmompa; verbatimElevation: 440; verbatimLatitude: 11.0193; verbatimLongitude: -85.41; verbatimCoordinateSystem: Decimal; **Identification:** identifiedBy: AJ Fleming; dateIdentified: 2016; **Event:** samplingProtocol: Reared from the larva of the hesperiid butterfly, Perichares poaceaphaga; verbatimEventDate: 10-Feb-2006; **Record Level:** language: en; institutionCode: CNC; collectionCode: Insects; basisOfRecord: Pinned Specimen
- **Type status:** Paratype. **Occurrence:** occurrenceDetails: http://janzen.sas.upenn.edu; catalogNumber: DHJPAR0017786; recordedBy: D.H. Janzen, W. Hallwachs & Jose Perez; individualID: DHJPAR0017786; individualCount: 1; sex: F; lifeStage: adult; preparations: pinned; otherCatalogNumbers: 00-SRNP-20847, BOLD:ABY7242, ASTAR497-07; **Taxon:** scientificName: Calolydella virginiajamesae; phylum: Arthropoda; class: Insecta; order: Diptera; family: Tachinidae; genus: Calolydella; specificEpithet: virginiajamesae; scientificNameAuthorship: Fleming & Wood, 2016; **Location:** continent: Central America; country: Costa Rica; countryCode: CR; stateProvince: Alajuela; county: Sector Rincon Rain Forest; locality: Area de Conservacion Guanacaste; verbatimLocality: Sendero Rincon; verbatimElevation: 430; verbatimLatitude: 10.8962; verbatimLongitude: -85.2777; verbatimCoordinateSystem: Decimal; **Identification:** identifiedBy: AJ Fleming; dateIdentified: 2016; **Event:** samplingProtocol: Reared from the larva of the nymphalid butterfly, Danaus plexippus; verbatimEventDate: 28-Nov-2000; **Record Level:** language: en; institutionCode: CNC; collectionCode: Insects; basisOfRecord: Pinned Specimen
- **Type status:** Paratype. **Occurrence:** occurrenceDetails: http://janzen.sas.upenn.edu; catalogNumber: DHJPAR0017769; recordedBy: D.H. Janzen, W. Hallwachs & Dunia Garcia; individualID: DHJPAR0017769; individualCount: 1; sex: M; lifeStage: adult; preparations: pinned; otherCatalogNumbers: 02-SRNP-15501, BOLD:ABY7242, ASTAR480-07; **Taxon:** scientificName: Calolydella virginiajamesae; phylum: Arthropoda; class: Insecta; order: Diptera; family: Tachinidae; genus: Calolydella; specificEpithet: virginiajamesae; scientificNameAuthorship: Fleming & Wood, 2016; **Location:** continent: Central America; country: Costa Rica; countryCode: CR; stateProvince: Guanacaste; county: Sector El Hacha; locality: Area de Conservacion Guanacaste; verbatimLocality: Finca Araya; verbatimElevation: 295; verbatimLatitude: 11.0154; verbatimLongitude: -85.5113; verbatimCoordinateSystem: Decimal; **Identification:** identifiedBy: AJ Fleming; dateIdentified: 2016; **Event:** samplingProtocol: Reared from the larva of the nymphalid butterfly, Mechanitis isthmia; verbatimEventDate: 26-Jun-2002; **Record Level:** language: en; institutionCode: CNC; collectionCode: Insects; basisOfRecord: Pinned Specimen
- **Type status:** Paratype. **Occurrence:** occurrenceDetails: http://janzen.sas.upenn.edu; catalogNumber: DHJPAR0052421; recordedBy: D.H. Janzen, W. Hallwachs & Cirilo Umana; individualID: DHJPAR0052421; individualCount: 1; sex: M; lifeStage: adult; preparations: pinned; otherCatalogNumbers: 13-SRNP-77065, BOLD:ABY7242, ASHYM1775-13; **Taxon:** scientificName: Calolydella virginiajamesae; phylum: Arthropoda; class: Insecta; order: Diptera; family: Tachinidae; genus: Calolydella; specificEpithet: virginiajamesae; scientificNameAuthorship: Fleming & Wood, 2016; **Location:** continent: Central America; country: Costa Rica; countryCode: CR; stateProvince: Alajuela; county: Sector Rincon Rain Forest; locality: Area de Conservacion Guanacaste; verbatimLocality: Quebrada Bambu; verbatimElevation: 109; verbatimLatitude: 10.9301; verbatimLongitude: -85.2521; verbatimCoordinateSystem: Decimal; **Identification:** identifiedBy: AJ Fleming; dateIdentified: 2016; **Event:** samplingProtocol: Reared from the larva of the erebid moth, Anycles adusta; verbatimEventDate: 09-Aug-2013; **Record Level:** language: en; institutionCode: CNC; collectionCode: Insects; basisOfRecord: Pinned Specimen
- **Type status:** Paratype. **Occurrence:** occurrenceDetails: http://janzen.sas.upenn.edu; catalogNumber: DHJPAR0011696; recordedBy: D.H. Janzen, W. Hallwachs & Eilyn Camacho; individualID: DHJPAR0011696; individualCount: 1; sex: F; lifeStage: adult; preparations: pinned; otherCatalogNumbers: 05-SRNP-60644, BOLD:ABY7242, ASTAS422-06; **Taxon:** scientificName: Calolydella virginiajamesae; phylum: Arthropoda; class: Insecta; order: Diptera; family: Tachinidae; genus: Calolydella; specificEpithet: virginiajamesae; scientificNameAuthorship: Fleming & Wood, 2016; **Location:** continent: Central America; country: Costa Rica; countryCode: CR; stateProvince: Guanacaste; county: Sector Santa Rosa; locality: Area de Conservacion Guanacaste; verbatimLocality: Quebrada Puercos; verbatimElevation: 155; verbatimLatitude: 10.8591; verbatimLongitude: -85.5709; verbatimCoordinateSystem: Decimal; **Identification:** identifiedBy: AJ Fleming; dateIdentified: 2016; **Event:** samplingProtocol: Reared from the larva of the noctuid moth, Cropia cedica; verbatimEventDate: 21-Sep-2005; **Record Level:** language: en; institutionCode: CNC; collectionCode: Insects; basisOfRecord: Pinned Specimen
- **Type status:** Paratype. **Occurrence:** occurrenceDetails: http://janzen.sas.upenn.edu; catalogNumber: DHJPAR0045648; recordedBy: D.H. Janzen, W. Hallwachs & Minor Carmona; individualID: DHJPAR0045648; individualCount: 1; sex: F; lifeStage: adult; preparations: pinned; otherCatalogNumbers: 11-SRNP-66015, BOLD:ABY7242, ACGAZ837-11; **Taxon:** scientificName: Calolydella virginiajamesae; phylum: Arthropoda; class: Insecta; order: Diptera; family: Tachinidae; genus: Calolydella; specificEpithet: virginiajamesae; scientificNameAuthorship: Fleming & Wood, 2016; **Location:** continent: Central America; country: Costa Rica; countryCode: CR; stateProvince: Alajuela; county: Brasilia; locality: Area de Conservacion Guanacaste; verbatimLocality: Gallinazo; verbatimElevation: 360; verbatimLatitude: 11.0183; verbatimLongitude: -85.372; verbatimCoordinateSystem: Decimal; **Identification:** identifiedBy: AJ Fleming; dateIdentified: 2016; **Event:** samplingProtocol: Reared from the larva of the noctuid moth, Perigea agnonia; verbatimEventDate: 01-Oct-2011; **Record Level:** language: en; institutionCode: CNC; collectionCode: Insects; basisOfRecord: Pinned Specimen
- **Type status:** Paratype. **Occurrence:** occurrenceDetails: http://janzen.sas.upenn.edu; catalogNumber: DHJPAR0017767; recordedBy: D.H. Janzen, W. Hallwachs & Ruth Franco; individualID: DHJPAR0017767; individualCount: 1; sex: M; lifeStage: adult; preparations: pinned; otherCatalogNumbers: 02-SRNP-32494, BOLD:ABY7242, ASTAR478-07; **Taxon:** scientificName: Calolydella virginiajamesae; phylum: Arthropoda; class: Insecta; order: Diptera; family: Tachinidae; genus: Calolydella; specificEpithet: virginiajamesae; scientificNameAuthorship: Fleming & Wood, 2016; **Location:** continent: Central America; country: Costa Rica; countryCode: CR; stateProvince: Guanacaste; county: Sector Santa Rosa; locality: Area de Conservacion Guanacaste; verbatimLocality: Quebrada Puercos; verbatimElevation: 155; verbatimLatitude: 10.8591; verbatimLongitude: -85.5709; verbatimCoordinateSystem: Decimal; **Identification:** identifiedBy: AJ Fleming; dateIdentified: 2016; **Event:** samplingProtocol: Reared from the larva of the pierid butterfly Colias cesonia; verbatimEventDate: 24-Nov-2002; **Record Level:** language: en; institutionCode: CNC; collectionCode: Insects; basisOfRecord: Pinned Specimen
- **Type status:** Paratype. **Occurrence:** occurrenceDetails: http://janzen.sas.upenn.edu; catalogNumber: DHJPAR0057673; recordedBy: D.H. Janzen, W. Hallwachs & Elissa Arroyo; individualID: DHJPAR0057673; individualCount: 1; sex: M; lifeStage: adult; preparations: pinned; otherCatalogNumbers: 15-SRNP-30814, BOLD:ABY7242, ASTAX046-15; **Taxon:** scientificName: Calolydella virginiajamesae; phylum: Arthropoda; class: Insecta; order: Diptera; family: Tachinidae; genus: Calolydella; specificEpithet: virginiajamesae; scientificNameAuthorship: Fleming & Wood, 2016; **Location:** continent: Central America; country: Costa Rica; countryCode: CR; stateProvince: Guanacaste; county: Sector Pitilla; locality: Area de Conservacion Guanacaste; verbatimLocality: Ingas; verbatimElevation: 580; verbatimLatitude: 11.0031; verbatimLongitude: -85.4204; verbatimCoordinateSystem: Decimal; **Identification:** identifiedBy: AJ Fleming; dateIdentified: 2016; **Event:** samplingProtocol: Reared from the larva of the crambid moth, Rhectocraspeda periusalis; verbatimEventDate: 25-May-2015; **Record Level:** language: en; institutionCode: CNC; collectionCode: Insects; basisOfRecord: Pinned Specimen
- **Type status:** Paratype. **Occurrence:** occurrenceDetails: http://janzen.sas.upenn.edu; catalogNumber: DHJPAR0007075; recordedBy: D.H. Janzen, W. Hallwachs & Manuel Rios; individualID: DHJPAR0007075; individualCount: 1; sex: M; lifeStage: adult; preparations: pinned; otherCatalogNumbers: 06-SRNP-31177, , ASTAV317-06; **Taxon:** scientificName: Calolydella virginiajamesae; phylum: Arthropoda; class: Insecta; order: Diptera; family: Tachinidae; genus: Calolydella; specificEpithet: virginiajamesae; scientificNameAuthorship: Fleming & Wood, 2016; **Location:** continent: Central America; country: Costa Rica; countryCode: CR; stateProvince: Guanacaste; county: Sector Pitilla; locality: Area de Conservacion Guanacaste; verbatimLocality: Pasmompa; verbatimElevation: 440; verbatimLatitude: 11.0193; verbatimLongitude: -85.41; verbatimCoordinateSystem: Decimal; **Identification:** identifiedBy: AJ Fleming; dateIdentified: 2016; **Event:** samplingProtocol: Reared from the larva of the lycaenid butterfly, Arawacus togarna; verbatimEventDate: 04-Apr-2006; **Record Level:** language: en; institutionCode: CNC; collectionCode: Insects; basisOfRecord: Pinned Specimen
- **Type status:** Paratype. **Occurrence:** occurrenceDetails: http://janzen.sas.upenn.edu; catalogNumber: DHJPAR0046613; recordedBy: D.H. Janzen, W. Hallwachs & Ricardo Calero; individualID: DHJPAR0046613; individualCount: 1; sex: M; lifeStage: adult; preparations: pinned; otherCatalogNumbers: 11-SRNP-72723, BOLD:ABY7242, ACGBA786-12; **Taxon:** scientificName: Calolydella virginiajamesae; phylum: Arthropoda; class: Insecta; order: Diptera; family: Tachinidae; genus: Calolydella; specificEpithet: virginiajamesae; scientificNameAuthorship: Fleming & Wood, 2016; **Location:** continent: Central America; country: Costa Rica; countryCode: CR; stateProvince: Guanacaste; county: Sector Pitilla; locality: Area de Conservacion Guanacaste; verbatimLocality: Medrano; verbatimElevation: 380; verbatimLatitude: 11.016; verbatimLongitude: -85.3805; verbatimCoordinateSystem: Decimal; **Identification:** identifiedBy: AJ Fleming; dateIdentified: 2016; **Event:** samplingProtocol: Reared from the larva of the erebid moth, noctJanzen01 Janzen102; verbatimEventDate: 04-Jan-2012; **Record Level:** language: en; institutionCode: CNC; collectionCode: Insects; basisOfRecord: Pinned Specimen
- **Type status:** Paratype. **Occurrence:** occurrenceDetails: http://janzen.sas.upenn.edu; catalogNumber: DHJPAR0023071; recordedBy: D.H. Janzen, W. Hallwachs & Roster Moraga; individualID: DHJPAR0023071; individualCount: 1; sex: M; lifeStage: adult; preparations: pinned; otherCatalogNumbers: 07-SRNP-24114, BOLD:ABY7242, ASTAW235-08; **Taxon:** scientificName: Calolydella virginiajamesae; phylum: Arthropoda; class: Insecta; order: Diptera; family: Tachinidae; genus: Calolydella; specificEpithet: virginiajamesae; scientificNameAuthorship: Fleming & Wood, 2016; **Location:** continent: Central America; country: Costa Rica; countryCode: CR; stateProvince: Guanacaste; county: Sector Del Oro; locality: Area de Conservacion Guanacaste; verbatimLocality: Uncaria; verbatimElevation: 370; verbatimLatitude: 11.0175; verbatimLongitude: -85.4741; verbatimCoordinateSystem: Decimal; **Identification:** identifiedBy: AJ Fleming; dateIdentified: 2016; **Event:** samplingProtocol: Reared from the larva of the limacodid moth, Epiperola vafera; verbatimEventDate: 08-Nov-2007; **Record Level:** language: en; institutionCode: CNC; collectionCode: Insects; basisOfRecord: Pinned Specimen
- **Type status:** Paratype. **Occurrence:** occurrenceDetails: http://janzen.sas.upenn.edu; catalogNumber: DHJPAR0010317; recordedBy: D.H. Janzen, W. Hallwachs & Lucia Vargas; individualID: DHJPAR0010317; individualCount: 1; sex: M; lifeStage: adult; preparations: pinned; otherCatalogNumbers: 06-SRNP-17555, BOLD:ABY7242, ASTAS148-06; **Taxon:** scientificName: Calolydella virginiajamesae; phylum: Arthropoda; class: Insecta; order: Diptera; family: Tachinidae; genus: Calolydella; specificEpithet: virginiajamesae; scientificNameAuthorship: Fleming & Wood, 2016; **Location:** continent: Central America; country: Costa Rica; countryCode: CR; stateProvince: Guanacaste; county: Sector Horizontes; locality: Area de Conservacion Guanacaste; verbatimLocality: Vado Esperanza; verbatimElevation: 85; verbatimLatitude: 10.7894; verbatimLongitude: -85.551; verbatimCoordinateSystem: Decimal; **Identification:** identifiedBy: AJ Fleming; dateIdentified: 2016; **Event:** samplingProtocol: Reared from the larva of the crambid moth, Spoladea recurvalis; verbatimEventDate: 18-Jul-2006; **Record Level:** language: en; institutionCode: CNC; collectionCode: Insects; basisOfRecord: Pinned Specimen
- **Type status:** Paratype. **Occurrence:** occurrenceDetails: http://janzen.sas.upenn.edu; catalogNumber: DHJPAR0050664; recordedBy: D.H. Janzen, W. Hallwachs & Ricardo Calero; individualID: DHJPAR0050664; individualCount: 1; sex: F; lifeStage: adult; preparations: pinned; otherCatalogNumbers: 12-SRNP-72398, BOLD:ABY7242, ACGBA3256-13; **Taxon:** scientificName: Calolydella virginiajamesae; phylum: Arthropoda; class: Insecta; order: Diptera; family: Tachinidae; genus: Calolydella; specificEpithet: virginiajamesae; scientificNameAuthorship: Fleming & Wood, 2016; **Location:** continent: Central America; country: Costa Rica; countryCode: CR; stateProvince: Guanacaste; county: Sector Pitilla; locality: Area de Conservacion Guanacaste; verbatimLocality: Medrano; verbatimElevation: 380; verbatimLatitude: 11.016; verbatimLongitude: -85.3805; verbatimCoordinateSystem: Decimal; **Identification:** identifiedBy: AJ Fleming; dateIdentified: 2016; **Event:** samplingProtocol: Reared from the larva of the crambid moth, Hileithia Solis01DHJ02; verbatimEventDate: 08-Dec-2012; **Record Level:** language: en; institutionCode: CNC; collectionCode: Insects; basisOfRecord: Pinned Specimen
- **Type status:** Paratype. **Occurrence:** occurrenceDetails: http://janzen.sas.upenn.edu; catalogNumber: DHJPAR0021000; recordedBy: D.H. Janzen, W. Hallwachs & Jose Perez; individualID: DHJPAR0021000; individualCount: 1; sex: F; lifeStage: adult; preparations: pinned; otherCatalogNumbers: 07-SRNP-42208, BOLD:ABY7242, ASTA1343-07; **Taxon:** scientificName: Calolydella virginiajamesae; phylum: Arthropoda; class: Insecta; order: Diptera; family: Tachinidae; genus: Calolydella; specificEpithet: virginiajamesae; scientificNameAuthorship: Fleming & Wood, 2016; **Location:** continent: Central America; country: Costa Rica; countryCode: CR; stateProvince: Alajuela; county: Sector Rincon Rain Forest; locality: Area de Conservacion Guanacaste; verbatimLocality: Camino Porvenir; verbatimElevation: 383; verbatimLatitude: 10.9038; verbatimLongitude: -85.2596; verbatimCoordinateSystem: Decimal; **Identification:** identifiedBy: AJ Fleming; dateIdentified: 2016; **Event:** samplingProtocol: Reared from the larva of the riodinid butterfly, Euselasia cheles; verbatimEventDate: 18-Aug-2007; **Record Level:** language: en; institutionCode: CNC; collectionCode: Insects; basisOfRecord: Pinned Specimen
- **Type status:** Paratype. **Occurrence:** occurrenceDetails: http://janzen.sas.upenn.edu; catalogNumber: DHJPAR0035672; recordedBy: D.H. Janzen, W. Hallwachs & Mariano Pereira; individualID: DHJPAR0035672; individualCount: 1; sex: M; lifeStage: adult; preparations: pinned; otherCatalogNumbers: 09-SRNP-56981, BOLD:ABY7242, ASHYD1053-09; **Taxon:** scientificName: Calolydella virginiajamesae; phylum: Arthropoda; class: Insecta; order: Diptera; family: Tachinidae; genus: Calolydella; specificEpithet: virginiajamesae; scientificNameAuthorship: Fleming & Wood, 2016; **Location:** continent: Central America; country: Costa Rica; countryCode: CR; stateProvince: Guanacaste; county: Sector Mundo Nuevo; locality: Area de Conservacion Guanacaste; verbatimLocality: Camino Pozo Tres; verbatimElevation: 733; verbatimLatitude: 10.7708; verbatimLongitude: -85.3742; verbatimCoordinateSystem: Decimal; **Identification:** identifiedBy: AJ Fleming; dateIdentified: 2016; **Event:** samplingProtocol: Reared from the larva of the nymphalid butterfly, Chlosyne rosita; verbatimEventDate: 06-Aug-2009; **Record Level:** language: en; institutionCode: CNC; collectionCode: Insects; basisOfRecord: Pinned Specimen
- **Type status:** Paratype. **Occurrence:** occurrenceDetails: http://janzen.sas.upenn.edu; catalogNumber: DHJPAR0046436; recordedBy: D.H. Janzen, W. Hallwachs & Jose Perez; individualID: DHJPAR0046436; individualCount: 1; sex: F; lifeStage: adult; preparations: pinned; otherCatalogNumbers: 11-SRNP-26137, BOLD:ABY7242, ACGBA609-12; **Taxon:** scientificName: Calolydella virginiajamesae; phylum: Arthropoda; class: Insecta; order: Diptera; family: Tachinidae; genus: Calolydella; specificEpithet: virginiajamesae; scientificNameAuthorship: Fleming & Wood, 2016; **Location:** continent: Central America; country: Costa Rica; countryCode: CR; stateProvince: Alajuela; county: Sector Rincon Rain Forest; locality: Area de Conservacion Guanacaste; verbatimLocality: Sendero Venado; verbatimElevation: 420; verbatimLatitude: 10.8968; verbatimLongitude: -85.27; verbatimCoordinateSystem: Decimal; **Identification:** identifiedBy: AJ Fleming; dateIdentified: 2016; **Event:** samplingProtocol: Reared from the larva of the crambid moth, Undulambia BioLep248; verbatimEventDate: 12-Jan-2012; **Record Level:** language: en; institutionCode: CNC; collectionCode: Insects; basisOfRecord: Pinned Specimen
- **Type status:** Paratype. **Occurrence:** occurrenceDetails: http://janzen.sas.upenn.edu; catalogNumber: DHJPAR0017052; recordedBy: D.H. Janzen, W. Hallwachs & Calixto Moraga; individualID: DHJPAR0017052; individualCount: 1; sex: F; lifeStage: adult; preparations: pinned; otherCatalogNumbers: 07-SRNP-30522, BOLD:ABY7242, ASTAP490-07; **Taxon:** scientificName: Calolydella virginiajamesae; phylum: Arthropoda; class: Insecta; order: Diptera; family: Tachinidae; genus: Calolydella; specificEpithet: virginiajamesae; scientificNameAuthorship: Fleming & Wood, 2016; **Location:** continent: Central America; country: Costa Rica; countryCode: CR; stateProvince: Guanacaste; county: Sector Pitilla; locality: Area de Conservacion Guanacaste; verbatimLocality: Sendero Nacho; verbatimElevation: 710; verbatimLatitude: 10.9845; verbatimLongitude: -85.4248; verbatimCoordinateSystem: Decimal; **Identification:** identifiedBy: AJ Fleming; dateIdentified: 2016; **Event:** samplingProtocol: Reared from the larva of the erebid moth, Coreura phoenicides; verbatimEventDate: 15-Feb-2007; **Record Level:** language: en; institutionCode: CNC; collectionCode: Insects; basisOfRecord: Pinned Specimen
- **Type status:** Paratype. **Occurrence:** occurrenceDetails: http://janzen.sas.upenn.edu; catalogNumber: DHJPAR0046433; recordedBy: D.H. Janzen, W. Hallwachs & Jose Perez; individualID: DHJPAR0046433; individualCount: 1; sex: M; lifeStage: adult; preparations: pinned; otherCatalogNumbers: 11-SRNP-26139, BOLD:ABY7242, ACGBA606-12; **Taxon:** scientificName: Calolydella virginiajamesae; phylum: Arthropoda; class: Insecta; order: Diptera; family: Tachinidae; genus: Calolydella; specificEpithet: virginiajamesae; scientificNameAuthorship: Fleming & Wood, 2016; **Location:** continent: Central America; country: Costa Rica; countryCode: CR; stateProvince: Alajuela; county: Sector Rincon Rain Forest; locality: Area de Conservacion Guanacaste; verbatimLocality: Sendero Venado; verbatimElevation: 420; verbatimLatitude: 10.8968; verbatimLongitude: -85.27; verbatimCoordinateSystem: Decimal; **Identification:** identifiedBy: AJ Fleming; dateIdentified: 2016; **Event:** samplingProtocol: Reared from the larva of the noctuid moth, Callopistria floridensis; verbatimEventDate: 14-Jan-2012; **Record Level:** language: en; institutionCode: CNC; collectionCode: Insects; basisOfRecord: Pinned Specimen
- **Type status:** Paratype. **Occurrence:** occurrenceDetails: http://janzen.sas.upenn.edu; catalogNumber: DHJPAR0018404; recordedBy: D.H. Janzen, W. Hallwachs & Petrona Rios; individualID: DHJPAR0018404; individualCount: 1; sex: F; lifeStage: adult; preparations: pinned; otherCatalogNumbers: 05-SRNP-30122, BOLD:ABY7242, ASTAI1051-07; **Taxon:** scientificName: Calolydella virginiajamesae; phylum: Arthropoda; class: Insecta; order: Diptera; family: Tachinidae; genus: Calolydella; specificEpithet: virginiajamesae; scientificNameAuthorship: Fleming & Wood, 2016; **Location:** continent: Central America; country: Costa Rica; countryCode: CR; stateProvince: Guanacaste; county: Sector Pitilla; locality: Area de Conservacion Guanacaste; verbatimLocality: Pasmompa; verbatimElevation: 440; verbatimLatitude: 11.0193; verbatimLongitude: -85.41; verbatimCoordinateSystem: Decimal; **Identification:** identifiedBy: AJ Fleming; dateIdentified: 2016; **Event:** samplingProtocol: Reared from the larva of the sphingid moth, Manduca muscosa; verbatimEventDate: 10-Feb-2005; **Record Level:** language: en; institutionCode: CNC; collectionCode: Insects; basisOfRecord: Pinned Specimen
- **Type status:** Paratype. **Occurrence:** occurrenceDetails: http://janzen.sas.upenn.edu; catalogNumber: DHJPAR0011713; recordedBy: D.H. Janzen, W. Hallwachs & Guillermo Pereira; individualID: DHJPAR0011713; individualCount: 1; sex: M; lifeStage: adult; preparations: pinned; otherCatalogNumbers: 04-SRNP-13310, BOLD:ABY7242, ASTAS439-06; **Taxon:** scientificName: Calolydella virginiajamesae; phylum: Arthropoda; class: Insecta; order: Diptera; family: Tachinidae; genus: Calolydella; specificEpithet: virginiajamesae; scientificNameAuthorship: Fleming & Wood, 2016; **Location:** continent: Central America; country: Costa Rica; countryCode: CR; stateProvince: Guanacaste; county: Sector Santa Rosa; locality: Area de Conservacion Guanacaste; verbatimLocality: Quebrada San Pancho; verbatimElevation: 90; verbatimLatitude: 10.7477; verbatimLongitude: -85.5858; verbatimCoordinateSystem: Decimal; **Identification:** identifiedBy: AJ Fleming; dateIdentified: 2016; **Event:** samplingProtocol: Reared from the larva of the crambid moth, Omiodes cuniculalis; verbatimEventDate: 13-Aug-2004; **Record Level:** language: en; institutionCode: CNC; collectionCode: Insects; basisOfRecord: Pinned Specimen
- **Type status:** Paratype. **Occurrence:** occurrenceDetails: http://janzen.sas.upenn.edu; catalogNumber: DHJPAR0016740; recordedBy: D.H. Janzen, W. Hallwachs & Jose Alberto Sanchez; individualID: DHJPAR0016740; individualCount: 1; sex: F; lifeStage: adult; preparations: pinned; otherCatalogNumbers: 06-SRNP-59934, BOLD:ABY7242, ASTAP850-07; **Taxon:** scientificName: Calolydella virginiajamesae; phylum: Arthropoda; class: Insecta; order: Diptera; family: Tachinidae; genus: Calolydella; specificEpithet: virginiajamesae; scientificNameAuthorship: Fleming & Wood, 2016; **Location:** continent: Central America; country: Costa Rica; countryCode: CR; stateProvince: Guanacaste; county: Sector Mundo Nuevo; locality: Area de Conservacion Guanacaste; verbatimLocality: Porton Rivas; verbatimElevation: 570; verbatimLatitude: 10.7586; verbatimLongitude: -85.3727; verbatimCoordinateSystem: Decimal; **Identification:** identifiedBy: AJ Fleming; dateIdentified: 2016; **Event:** samplingProtocol: Reared from the larva of the crambid moth, Omiodes cuniculalis; verbatimEventDate: 06-Dec-2006; **Record Level:** language: en; institutionCode: CNC; collectionCode: Insects; basisOfRecord: Pinned Specimen
- **Type status:** Paratype. **Occurrence:** occurrenceDetails: http://janzen.sas.upenn.edu; catalogNumber: DHJPAR0046439; recordedBy: D.H. Janzen, W. Hallwachs & Jose Perez; individualID: DHJPAR0046439; individualCount: 1; sex: F; lifeStage: adult; preparations: pinned; otherCatalogNumbers: 11-SRNP-26138, BOLD:ABY7242, ACGBA612-12; **Taxon:** scientificName: Calolydella virginiajamesae; phylum: Arthropoda; class: Insecta; order: Diptera; family: Tachinidae; genus: Calolydella; specificEpithet: virginiajamesae; scientificNameAuthorship: Fleming & Wood, 2016; **Location:** continent: Central America; country: Costa Rica; countryCode: CR; stateProvince: Alajuela; county: Sector Rincon Rain Forest; locality: Area de Conservacion Guanacaste; verbatimLocality: Sendero Venado; verbatimElevation: 420; verbatimLatitude: 10.8968; verbatimLongitude: -85.27; verbatimCoordinateSystem: Decimal; **Identification:** identifiedBy: AJ Fleming; dateIdentified: 2016; **Event:** samplingProtocol: Reared from the larva of the noctuid moth, Callopistria floridensis; verbatimEventDate: 10-Jan-2012; **Record Level:** language: en; institutionCode: CNC; collectionCode: Insects; basisOfRecord: Pinned Specimen
- **Type status:** Paratype. **Occurrence:** occurrenceDetails: http://janzen.sas.upenn.edu; catalogNumber: DHJPAR0040787; recordedBy: D.H. Janzen, W. Hallwachs & Dunia Garcia; individualID: DHJPAR0040787; individualCount: 1; sex: M; lifeStage: adult; preparations: pinned; otherCatalogNumbers: 10-SRNP-36111, BOLD:ABY7242, ASHYE2923-11; **Taxon:** scientificName: Calolydella virginiajamesae; phylum: Arthropoda; class: Insecta; order: Diptera; family: Tachinidae; genus: Calolydella; specificEpithet: virginiajamesae; scientificNameAuthorship: Fleming & Wood, 2016; **Location:** continent: Central America; country: Costa Rica; countryCode: CR; stateProvince: Guanacaste; county: Sector Cacao; locality: Area de Conservacion Guanacaste; verbatimLocality: Sendero a Maritza, 1 km NW Estacion Cacao; verbatimElevation: 1150; verbatimLatitude: 10.9592; verbatimLongitude: -85.4682; verbatimCoordinateSystem: Decimal; **Identification:** identifiedBy: AJ Fleming; dateIdentified: 2016; **Event:** samplingProtocol: Reared from the larva of the hesperiid butterfly, Mylon lassia; verbatimEventDate: 22-Sep-2010; **Record Level:** language: en; institutionCode: CNC; collectionCode: Insects; basisOfRecord: Pinned Specimen
- **Type status:** Paratype. **Occurrence:** occurrenceDetails: http://janzen.sas.upenn.edu; catalogNumber: DHJPAR0003459; recordedBy: D.H. Janzen, W. Hallwachs & Petrona Rios; individualID: DHJPAR0003459; individualCount: 1; sex: F; lifeStage: adult; preparations: pinned; otherCatalogNumbers: 05-SRNP-30122, , ASTA366-05; **Taxon:** scientificName: Calolydella virginiajamesae; phylum: Arthropoda; class: Insecta; order: Diptera; family: Tachinidae; genus: Calolydella; specificEpithet: virginiajamesae; scientificNameAuthorship: Fleming & Wood, 2016; **Location:** continent: Central America; country: Costa Rica; countryCode: CR; stateProvince: Guanacaste; county: Sector Pitilla; locality: Area de Conservacion Guanacaste; verbatimLocality: Pasmompa; verbatimElevation: 440; verbatimLatitude: 11.0193; verbatimLongitude: -85.41; verbatimCoordinateSystem: Decimal; **Identification:** identifiedBy: AJ Fleming; dateIdentified: 2016; **Event:** samplingProtocol: Reared from the larva of the sphingid moth, Manduca muscosa; verbatimEventDate: 10-Feb-2005; **Record Level:** language: en; institutionCode: CNC; collectionCode: Insects; basisOfRecord: Pinned Specimen
- **Type status:** Paratype. **Occurrence:** occurrenceDetails: http://janzen.sas.upenn.edu; catalogNumber: DHJPAR0017785; recordedBy: D.H. Janzen, W. Hallwachs & Jose Perez; individualID: DHJPAR0017785; individualCount: 1; sex: F; lifeStage: adult; preparations: pinned; otherCatalogNumbers: 00-SRNP-20847, BOLD:ABY7242, ASTAR496-07; **Taxon:** scientificName: Calolydella virginiajamesae; phylum: Arthropoda; class: Insecta; order: Diptera; family: Tachinidae; genus: Calolydella; specificEpithet: virginiajamesae; scientificNameAuthorship: Fleming & Wood, 2016; **Location:** continent: Central America; country: Costa Rica; countryCode: CR; stateProvince: Alajuela; county: Sector Rincon Rain Forest; locality: Area de Conservacion Guanacaste; verbatimLocality: Sendero Rincon; verbatimElevation: 430; verbatimLatitude: 10.8962; verbatimLongitude: -85.2777; verbatimCoordinateSystem: Decimal; **Identification:** identifiedBy: AJ Fleming; dateIdentified: 2016; **Event:** samplingProtocol: Reared from the larva of the nymphalid butterfly, Danaus plexippus; verbatimEventDate: 28-Nov-2000; **Record Level:** language: en; institutionCode: CNC; collectionCode: Insects; basisOfRecord: Pinned Specimen
- **Type status:** Paratype. **Occurrence:** occurrenceDetails: http://janzen.sas.upenn.edu; catalogNumber: DHJPAR0029640; recordedBy: D.H. Janzen, W. Hallwachs & Gloria Sihezar; individualID: DHJPAR0029640; individualCount: 1; sex: F; lifeStage: adult; preparations: pinned; otherCatalogNumbers: 08-SRNP-5217, BOLD:ABY7242, ASHYM1061-09; **Taxon:** scientificName: Calolydella virginiajamesae; phylum: Arthropoda; class: Insecta; order: Diptera; family: Tachinidae; genus: Calolydella; specificEpithet: virginiajamesae; scientificNameAuthorship: Fleming & Wood, 2016; **Location:** continent: Central America; country: Costa Rica; countryCode: CR; stateProvince: Alajuela; county: Sector San Cristobal; locality: Area de Conservacion Guanacaste; verbatimLocality: Rio Areno; verbatimElevation: 460; verbatimLatitude: 10.9141; verbatimLongitude: -85.3817; verbatimCoordinateSystem: Decimal; **Identification:** identifiedBy: AJ Fleming; dateIdentified: 2016; **Event:** samplingProtocol: Reared from the larva of the erebid moth, Hypena coatilis; verbatimEventDate: 07-Oct-2008; **Record Level:** language: en; institutionCode: CNC; collectionCode: Insects; basisOfRecord: Pinned Specimen
- **Type status:** Paratype. **Occurrence:** occurrenceDetails: http://janzen.sas.upenn.edu; catalogNumber: DHJPAR0030167; recordedBy: D.H. Janzen, W. Hallwachs & Petrona Rios; individualID: DHJPAR0030167; individualCount: 1; sex: M; lifeStage: adult; preparations: pinned; otherCatalogNumbers: 08-SRNP-32592, BOLD:ABY7242, ASHYB911-09; **Taxon:** scientificName: Calolydella virginiajamesae; phylum: Arthropoda; class: Insecta; order: Diptera; family: Tachinidae; genus: Calolydella; specificEpithet: virginiajamesae; scientificNameAuthorship: Fleming & Wood, 2016; **Location:** continent: Central America; country: Costa Rica; countryCode: CR; stateProvince: Guanacaste; county: Sector Pitilla; locality: Area de Conservacion Guanacaste; verbatimLocality: Amonias; verbatimElevation: 390; verbatimLatitude: 11.0425; verbatimLongitude: -85.4034; verbatimCoordinateSystem: Decimal; **Identification:** identifiedBy: AJ Fleming; dateIdentified: 2016; **Event:** samplingProtocol: Reared from the larva of the geometrid moth, Eois Janzen04; verbatimEventDate: 05-Nov-2008; **Record Level:** language: en; institutionCode: CNC; collectionCode: Insects; basisOfRecord: Pinned Specimen
- **Type status:** Paratype. **Occurrence:** occurrenceDetails: http://janzen.sas.upenn.edu; catalogNumber: DHJPAR0016580; recordedBy: D.H. Janzen, W. Hallwachs & Manuel Rios; individualID: DHJPAR0016580; individualCount: 1; sex: F; lifeStage: adult; preparations: pinned; otherCatalogNumbers: 06-SRNP-65089, BOLD:ABY7242, ASTAP784-07; **Taxon:** scientificName: Calolydella virginiajamesae; phylum: Arthropoda; class: Insecta; order: Diptera; family: Tachinidae; genus: Calolydella; specificEpithet: virginiajamesae; scientificNameAuthorship: Fleming & Wood, 2016; **Location:** continent: Central America; country: Costa Rica; countryCode: CR; stateProvince: Guanacaste; county: Sector Pitilla; locality: Area de Conservacion Guanacaste; verbatimLocality: Coneja; verbatimElevation: 415; verbatimLatitude: 11.0153; verbatimLongitude: -85.3977; verbatimCoordinateSystem: Decimal; **Identification:** identifiedBy: AJ Fleming; dateIdentified: 2016; **Event:** samplingProtocol: Reared from the larva of the riodinid butterfly, Ancyluris inca; verbatimEventDate: 22-Nov-2006; **Record Level:** language: en; institutionCode: CNC; collectionCode: Insects; basisOfRecord: Pinned Specimen

#### Description

**Male** (Fig. 37a, b, c). Length: 5–8mm. **Head** (Fig. 37b): frontal setae extending beyond base of postpedicel; fronto-orbital plate gold, with a single row of fine setulae outside of frontal setae; parafacial silver throughout. **Thorax** (Fig. 37a, c): gold on both dorsal and lateral surfaces; outermost two vittae twice as wide as innermost two; postpronotum with three setae; 3:3 acrostichal setae; 3:3 dorsocentral setae; 2:3 intra-alar setae; 2:3 supra-alar setae; three katepisternal setae; anatergite with three or more hair-like setae, often in a small tuft; scutellar discal setae situated as wide apart as subapical scutellar setae. Wing vein R4+5 with 7–9 setulae from base to crossvein R-M. **Abdomen** (Fig. 37a): with uninterrupted transverse marginal pollinose bands, and with an orange spot lateroventrally at base of ST1+2; T3 with one pair of median marginal setae and one pair of discal setae; T4 with one pair of discal setae. **Terminalia** (Fig. 38): sternite 5 (Fig. 38c) with two small lobes and a wide U-shaped median cleft, 0.48X the length of the sternite from lobe to apex; inner margin covered by dense pollinosity, appearing darker than surrounding cuticle; entire lobe of sternite with short setae, of varying lengths. Cerci (Fig. 38b), in dorsal view, separated by a narrow gap widening at apex, and with separated tips; each cercus long, slender and straight, very slightly tapered from its already narrow base; setose along its entire length. Surstylus (Fig. 38a) slender and digitiform, appearing 0.2X shorter than cercus when viewed laterally; with short setae along entire length; tip of surstylus curved inwards when viewed dorsally.

**Female** (Fig. 37d, e, f). Length: 4–7mm. Fronto-orbital plate 2.1X wider than in male.

#### Diagnosis

*Calolydella virginiajamesae* can be distinguished from all other species of *Calolydella* by the following combination of traits: fronto-orbital plate with a single row of small black setulae outside of frontal setae, parafacial silver-pollinose, anatergite with three or more setae arranged in a small tuft, and T3–T4 each with a single pair of discal setae.

#### Etymology

The specific epithet is in honor of Virginia James of Levittown, Pennsylvania, in recognition of the moral and family support of her mother, Tanya Dapkey, in her efforts curating and preparing ACG parasitoid flies for DNA barcoding.

#### Distribution

Costa Rica, ACG, Alajuela and Guanacaste provinces, 85–1150m.

#### Ecology

*Calolydella virginiajamesae* has been reared 34 times from various species of Lepidoptera from several families (Nymphalidae; Hesperiidae; Lycaenidae; Geometridae; Crambidae; Erebidae; Riodinidae; Sphingidae; and Pieridae), in rain forest, cloud forest, dry forest, and dry-rain lowland intergrade ecosystems.

## Identification Keys

### Key to the species of *Calolydella* of the Mesoamerican region

**Table:** 

1	Fronto-orbital plate with gold pollinosity; thorax with some gold on both lateral and dorsal surfaces	2
–	Fronto-orbital plate entirely silver; thorax silver on both lateral and dorsal surfaces	25
2	Fronto-orbital plate entirely gold	3
–	Fronto-orbital plate with both silver and gold pollinosity (Fig. 15b, d)	*Calolydella erasmocoronadoi* **sp. n.**
3	Abdomen with transverse marginal pollinose bands interrupted by a median dark stripe	4
–	Abdomen with uninterrupted transverse marginal pollinose bands	8
4	Parafacial pollinosity either entirely silver or up to 50% silver pollinose	5
–	Parafacial pollinosity entirely gold	*Calolydella gentica* (Walker, 1860)
5	Fronto-orbital plate with setulae outside of frontal seta; thorax with four distinct dorsal vittae; anatergite with up to two fine hair-like seta; T3 with one pair of median marginal setae	6
–	Fronto-orbital plate bare; thorax with two, prominent, dark dorsal vittae; anatergite with three or more hair-like setae; T3 with a complete row of marginal setae and one pair of discal setae	7
6	Fronto-orbital plate sparsely setulose; frontal setae not extending beyond base of postpedicel; anatergite bare; wing vein R_4+5_ with 4–5 small setulae dorsally, near base; T3 with one pair of discal setae (Fig. 9a)	*Calolydella crocata* **sp. n.**
–	Fronto-orbital plate with a row of fine setulae outside of frontal setae; frontal setae extending beyond base of postpedicel; anatergite with up to two hair-like setae; wing vein R_4+5_ with at most three small setulae dorsally, near base; T3 with two pairs of discal setae	*Calolydella trifasciata* (Walker, 1837)
7	Thorax with two presutural acrostichal setae; abdominal ST1+2 mostly black dorsally, with small spots of gold pollinosity laterally (Fig. 23a, d)	*Calolydella interrupta* **sp. n.**
–	Thorax with three presutural acrostichal setae; abdominal ST1+2 with a thick median black stripe but still mostly gold dorsally (Fig. 6a, c)	*Calolydella bicolor* **sp. n.**
8	T3 with at least one pair of median marginal setae	9
–	T3 without median marginal setae (Fig. 11a, d)	*Calolydella destituta* **sp. n.**
9	T3 with at least one pair of discal setae	10
–	T3 without discal setae (Fig. 26a, d)	*Calolydella omissa* **sp. n.**
10	Palpus dark gray to black	11
–	Palpus yellow-orange	12
11	Frontal setae extending to base of postpedicel but not beyond; thoracic vittae only faintly visible; abdominal ground color black (Fig. 24a, b)	*Calolydella nigripalpis* **sp. n.**
–	Frontal setae extending beyond base of postpedicel; thoracic vittae prominent and easily visible; abdominal ground color dark brown-orange (Fig. 34a, b, d, e)	*Calolydella tenebrosa* **sp. n.**
12	One or more pairs of discal setae on abdominal T4	13
–	A complete row of discal setae on abdominal T4 (Fig. 28a, b, d, e)	*Calolydella ordinalis* **sp. n.**
13	Frontal setae extending beyond base of postpedicel	14
–	Frontal setae extending to base of postpedicel	17
14	Parafacial silver; palpus normal; T3 and T4 each with one pair of discal setae	15
–	Parafacial gold; palpus normal in male but significantly enlarged in female (Fig. 21b, e); T3 and T4 each with two pairs of discal setae (Fig. 21c, f)	*Calolydella inflatipalpis* **sp. n.**
15	Four distinct thoracic vittae postsuturally	16
–	Thoracic vittae fused postsuturally (Fig. 35a, d)	*Calolydella timjamesi* **sp. n.**
16	Anatergite with up to two hair-like setae or, rarely, bare (Fig. 17a, d)	*Calolydella felipechavarriai* **sp. n.**
–	Anatergite with a small tuft of three or more hair-like setae (Fig. 37a, d)	*Calolydella virginiajamesae* **sp. n.**
17	Abdominal pollinosity concolorous on both ventral and dorsal surfaces	18
–	Abdominal pollinosity gold dorsally, silver ventrally	19
18	Parafacial all gold pollinose; palpus black basally; thorax with four regular thoracic vittae; scutellum without discal setae; abdomen silver pollinose on both dorsal and ventral surfaces (Fig. 5a, b)	*Calolydella aureofacies* **sp. n.**
–	Parafacial with some silver pollinosity; palpus orange; thorax with two bold and prominent thoracic vittae; scutellum with discal setae; abdomen gold pollinose on both dorsal and ventral surfaces (Fig. 7a, d)	*Calolydella bifissus* **sp. n.**
19	Base of ST1+2 with an orange spot lateroventrally	20
–	Base of ST1+2 black lateroventrally (Fig. 33a, b)	*Calolydella tanyadapkeyae* **sp. n.**
20	Parafacial silver-pollinose throughout	21
–	Parafacial with both silver and gold pollinosity	22
21	Anatergite with three or more hair-like setae arranged in a tuft; thoracic chaetotaxy as follows: 3:3 acrostichal setae; 3:3 dorsocentral setae; 2:3 intra-alar setae; 2:3 supra-alar setae; abdominal T4 with one pair of discal setae (Fig. 19a, b, d, e)	*Calolydella fredriksjobergi* **sp. n.**
–	Anatergite bare; thoracic chaetotaxy as follows: 3:3 acrostichal setae (2:3 in females); 3:4 dorsocentral setae; 1:3 intra-alar setae; 1:3 supra-alar setae; abdominal T4 with two pairs of discal setae(Fig. 1a, b, d, e)	*Calolydella adelinamoralesae* **sp. n.**
22	Outermost thoracic vittae 2X as wide as innermost, these becoming fused postsuturally	23
–	Four thoracic vittae of sub-equal width, not fused postsuturally	24
23	Fronto-orbital plate with a single row of setulae outside of frontal setae; postpronotum with three setae; wing vein R_4+5_ with 5–7 setulae dorsally, reaching from base almost to crossvein R-M; abdominal T4 with two pairs of discal setae (Fig. 32a, b, d, e)	*Calolydella susanaroibasae* **sp. n.**
–	Fronto-orbital plate with setulae interspersed among frontal setae; postpronotum with two setae, inner basal seta absent; wing vein R_4+5_ with at most three small setulae dorsally at base; T4 with one pair of discal setae (Fig. 3a, b)	*Calolydella alexanderjamesi* **sp. n.**
24	Fronto-orbital plate with a single row of setulae outside of frontal setae; thoracic chaetotaxy as follows: 3:3 acrostichal setae; 2:3 dorsocentral setae; 3:3 intra-alar setae; 2:3 supra-alar setae; wing vein R_4+5_ with more than 4–5 setulae dorsally, which may or may not extend to crossvein R-M; T3 and T4 both with up to two pairs of discal setae (Fig. 13a, b)	*Calolydella discalis* **sp. n.**
–	Fronto-orbital plate with setulae interspersed among frontal setae; thoracic chaetotaxy as follows: 3:3 acrostichal setae; 3:3 dorsocentral setae; 1:3 intra-alar setae; 2:3 supra-alar setae; wing vein R_4+5_ with at most 2–3 setulae dorsally at base, never extending to crossvein R-M; T3 with 1 pair of discal setae; T4 with up to two pairs of discal setae (Fig. 30a, b)	*Calolydella renemalaisei* **sp. n.**
25	Thorax with two longitudinal vittae	26
–	Thorax with four distinct longitudinal vittae	28
26	T3 with only one pair of discal setae; abdominal pollinosity appearing as thick uninterrupted bands across tergites	27
–	T3 with two pairs of discal setae; abdominal pollinosity across tergites interrupted by a dark median stripe	*Calolydella summatis* Reinhard, 1974
27	Frontal setae extending to base of postpedicel; three supravibrissal setae; 3:3 acrostichal setae; anatergite with three or more hair-like setae in a small tuft; anterior 2/3 of abdominal tergites silver pollinose; wing vein R_4+5_ with 6–7 setulae dorsally, extending almost to crossvein R-M (Fig. 4a, b)	*Calolydella argentea* **sp. n.**
–	Frontal setae extending beyond base of postpedicel; six or more supravibrissal setae; 2:3 acrostichal setae; anatergite bare; anterior 1/3 of abdominal tergites silver pollinose; wing vein R_4+5_ with two setulae dorsally, not extending to crossvein R-M	*Calolydella cylindriventris* (Wulp, 1890)
28	Fronto-orbital plate bare; frontal setae reaching base of postpedicel; thoracic pollinosity silver throughout; T3 with one pair of discal setae and one pair of median marginal setae	*Calolydella triangulifera* (Bigot, 1889)
–	Fronto-orbital plate with sparse setulae interspersed among frontal setae; frontal setae reaching beyond base of postpedicel; thoracic pollinosity gold throughout; T3 with two pairs of discal setae and a row of marginal setae	*Calolydella blandita* (Wulp, 1890)

This key is confined to species of *Calolydella* occurring in the Mesoamerican biogeographical region. It is applicable to both males and females.

## Analysis

### DNA Barcoding

**Figure 39.**
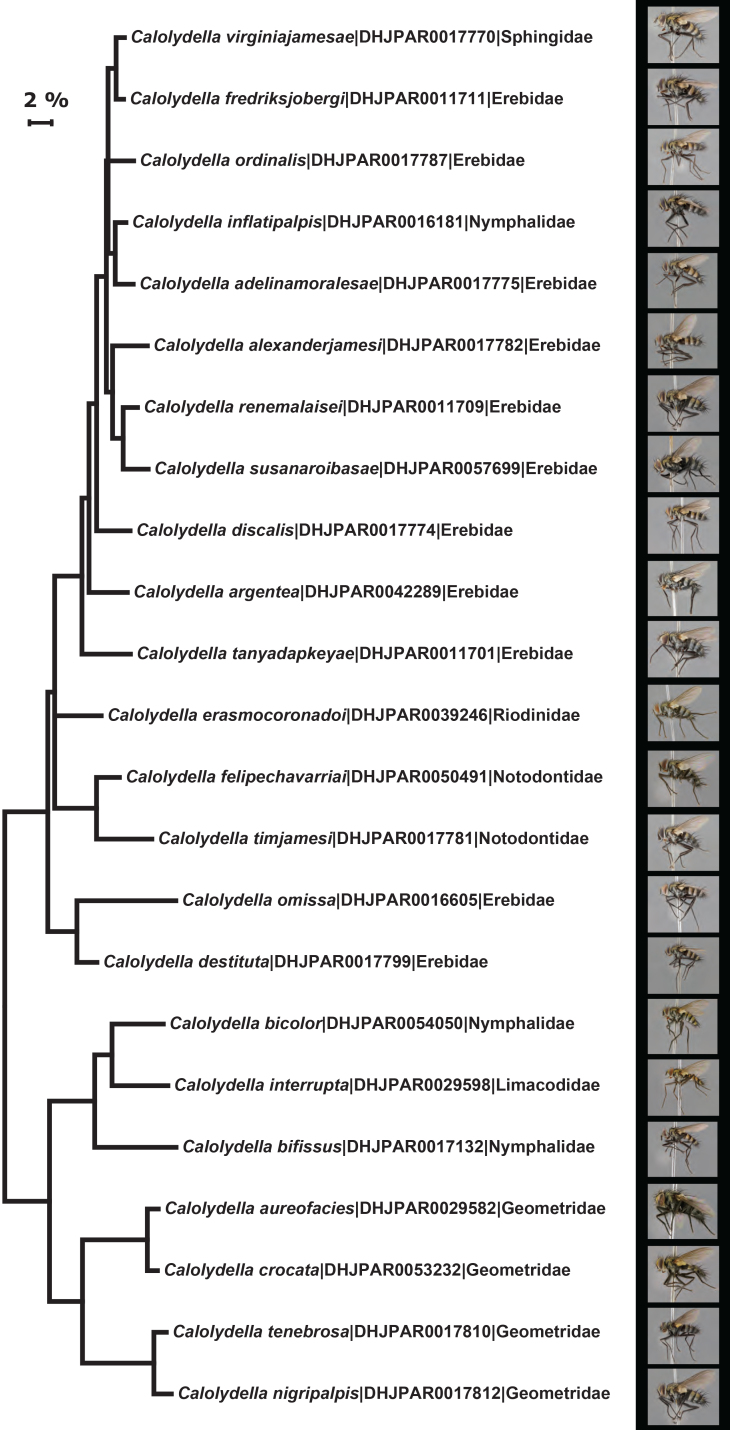
**Maximum Likelihood tree showing DNA barcode variation within ACG species of *Calolydella*.** The inter- and intra-specific variation within the DNA barcode region for the 23 species of *Calolydella* from ACG is illustrated using a Maximum Likelihood tree based on the General Time Reversible model (GTR) (Nei and Kumar 2000). GTR was selected due to the lowest BIC scores (Bayesian Information Criterion) of the Maximum Likelihood fits of 23 different nucleotide substitution models run in MEGA6 (Tamura et al. 2013). Tip labels include species name|specimen code|host family of each species. A habitus image in lateral view of each holotype is presented with the tree.

A phylogenetic tree based on DNA barcodes was used to visually demonstrate the variation within and between species, and is presented in Fig. 39. Interested readers can consult the Barcode of Life Data System (BOLD) (Ratnasingham and Hebert 2007) for all information associated with each sequence (including GenBank accession numbers), derived from each individual specimen using the persistent DOI: dx.doi.org/10.5883/DS-ASCALOL.

## Supplementary Material

XML Treatment for Calolydella

XML Treatment for Calolydella adelinamoralesae

XML Treatment for Calolydella alexanderjamesi

XML Treatment for Calolydella argentea

XML Treatment for Calolydella aureofacies

XML Treatment for Calolydella bicolor

XML Treatment for Calolydella bifissus

XML Treatment for Calolydella crocata

XML Treatment for Calolydella destituta

XML Treatment for Calolydella discalis

XML Treatment for Calolydella erasmocoronadoi

XML Treatment for Calolydella felipechavarriai

XML Treatment for Calolydella fredriksjobergi

XML Treatment for Calolydella inflatipalpis

XML Treatment for Calolydella interrupta

XML Treatment for Calolydella nigripalpis

XML Treatment for Calolydella omissa

XML Treatment for Calolydella ordinalis

XML Treatment for Calolydella renemalaisei

XML Treatment for Calolydella susanaroibasae

XML Treatment for Calolydella tanyadapkeyae

XML Treatment for Calolydella tenebrosa

XML Treatment for Calolydella timjamesi

XML Treatment for Calolydella virginiajamesae

## Figures and Tables

**Figure 1a.**
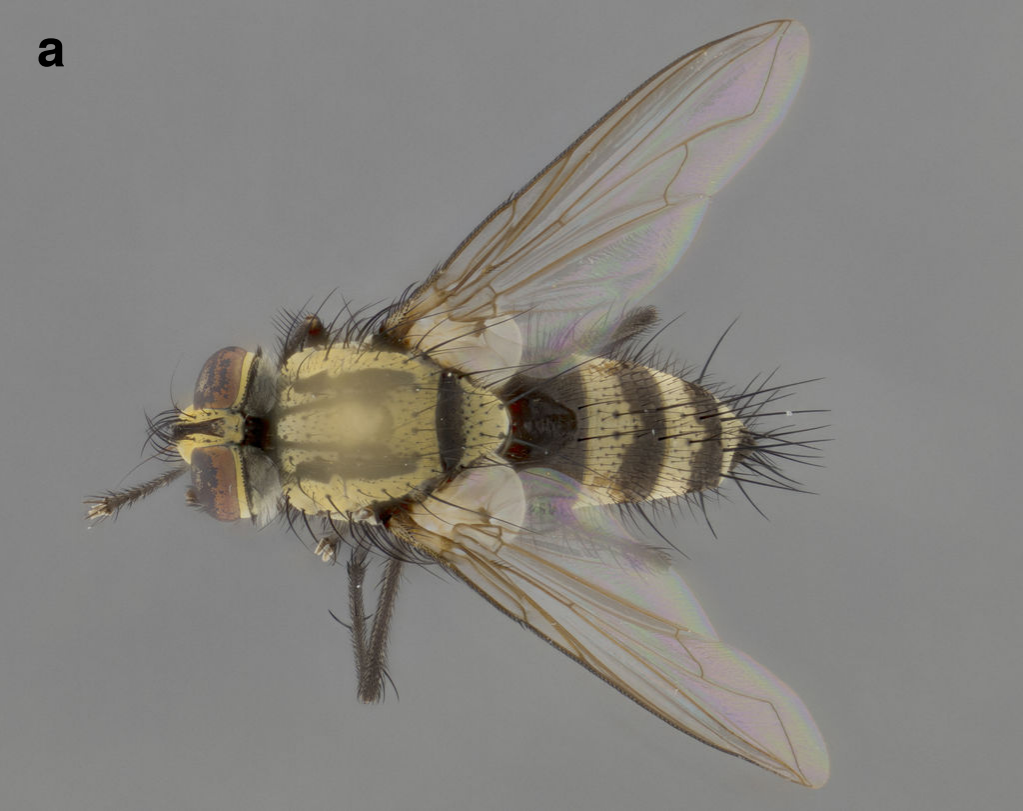
habitus in dorsal view

**Figure 1b.**
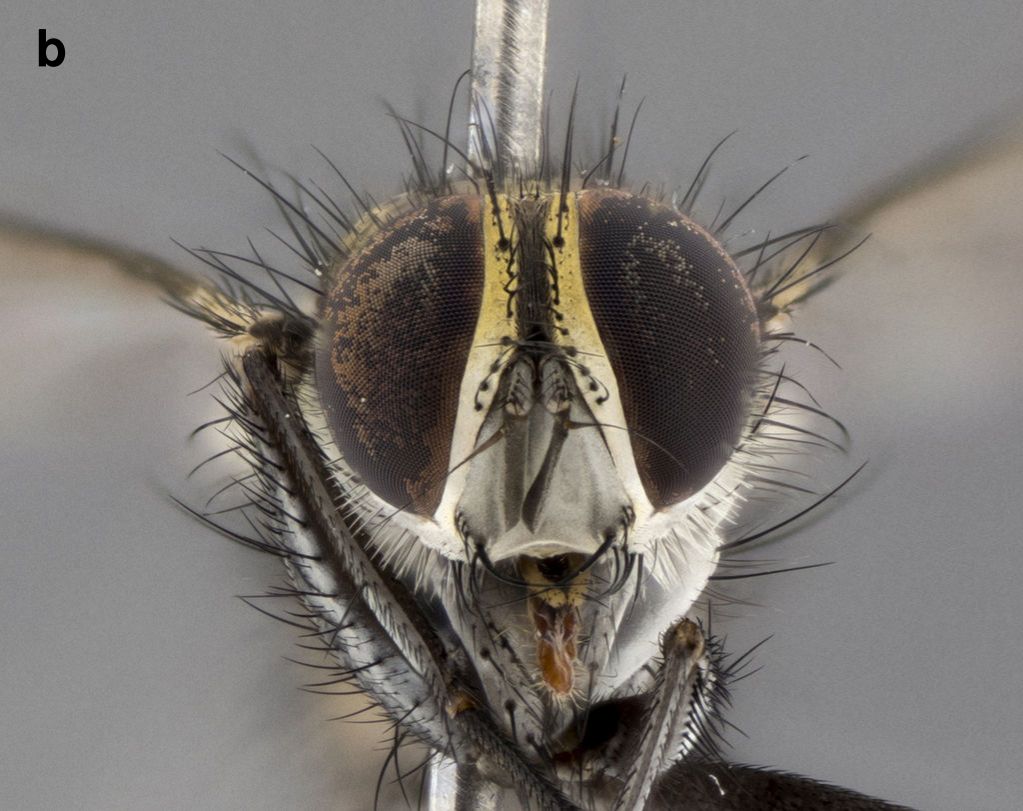
head in frontal view

**Figure 1c.**
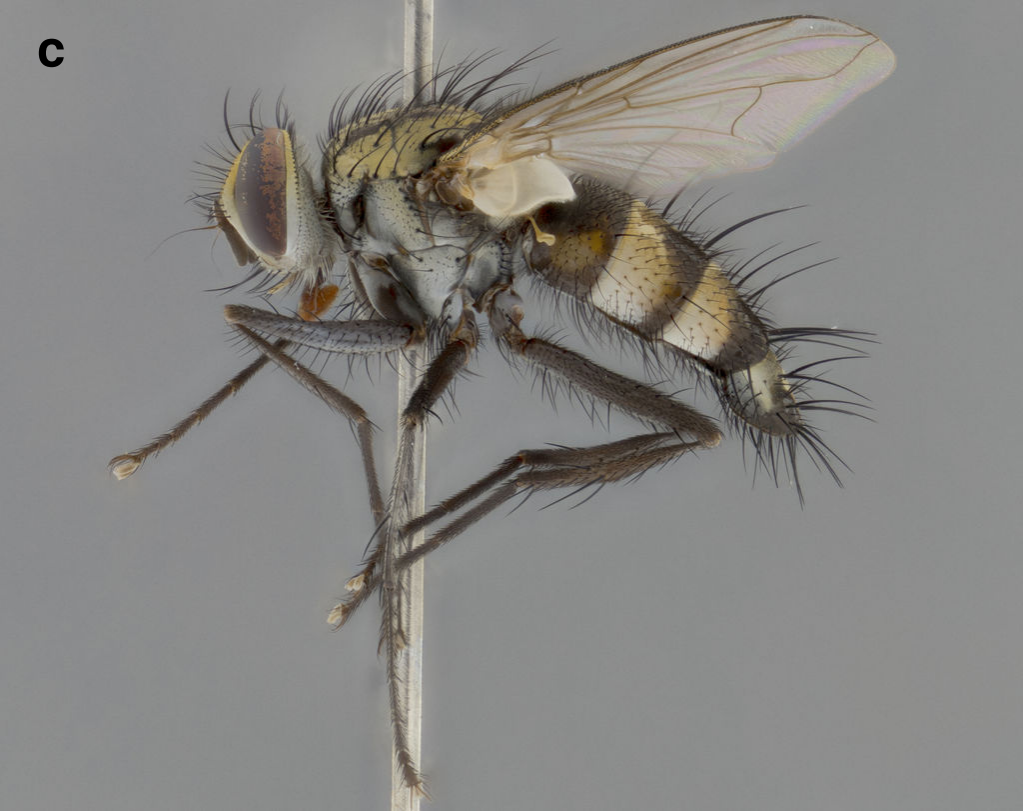
habitus in lateral view

**Figure 1d.**
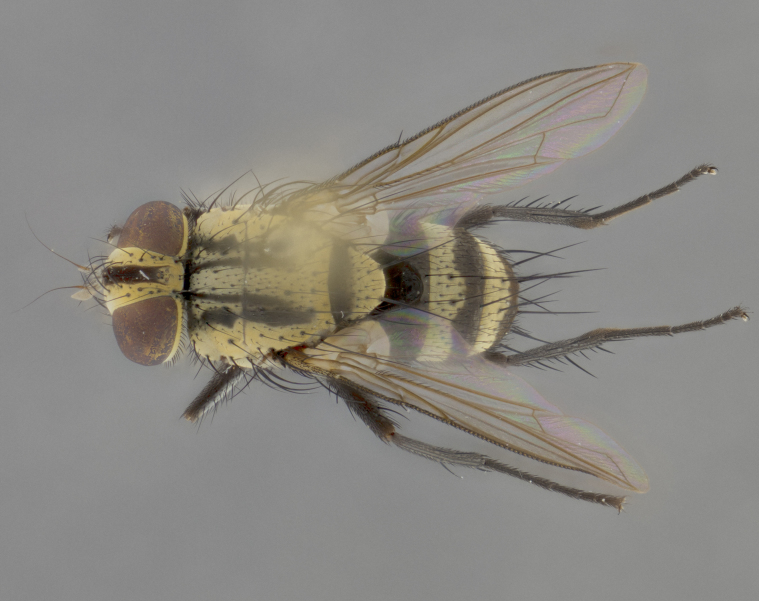
habitus in dorsal view

**Figure 1e.**
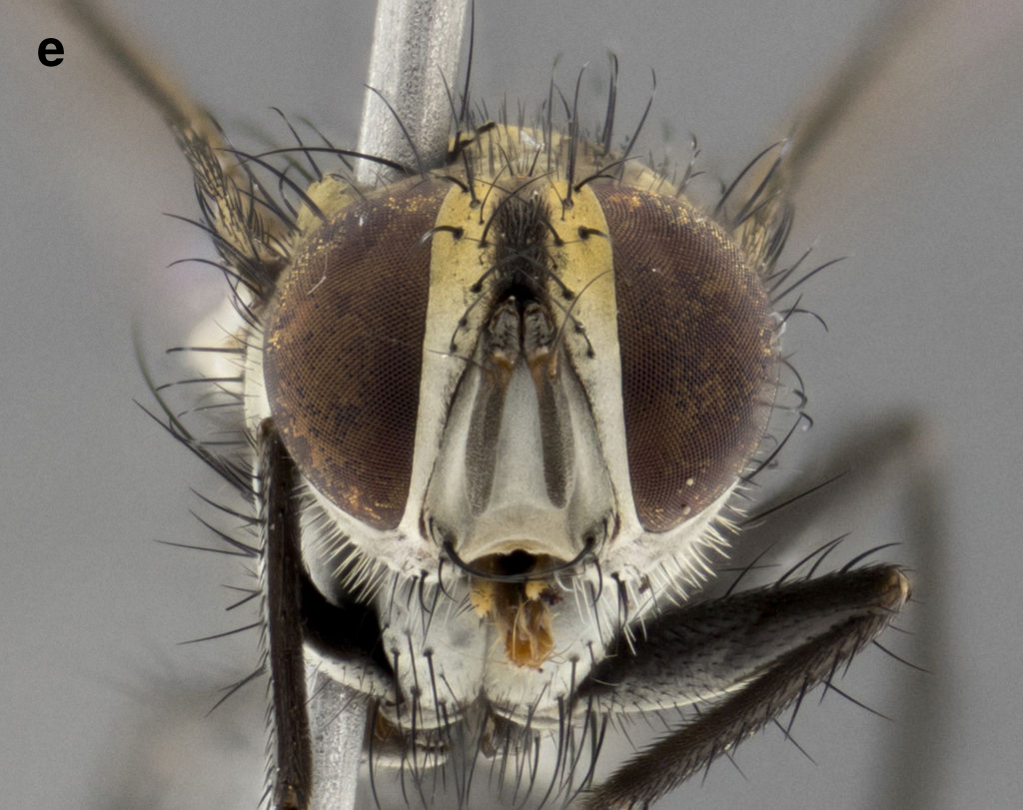
head in frontal view

**Figure 1f.**
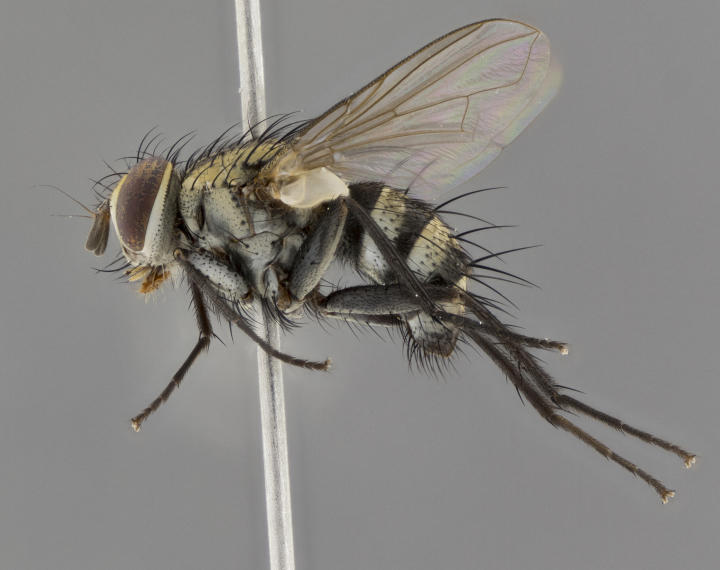
habitus in lateral view

**Figure 2a.**
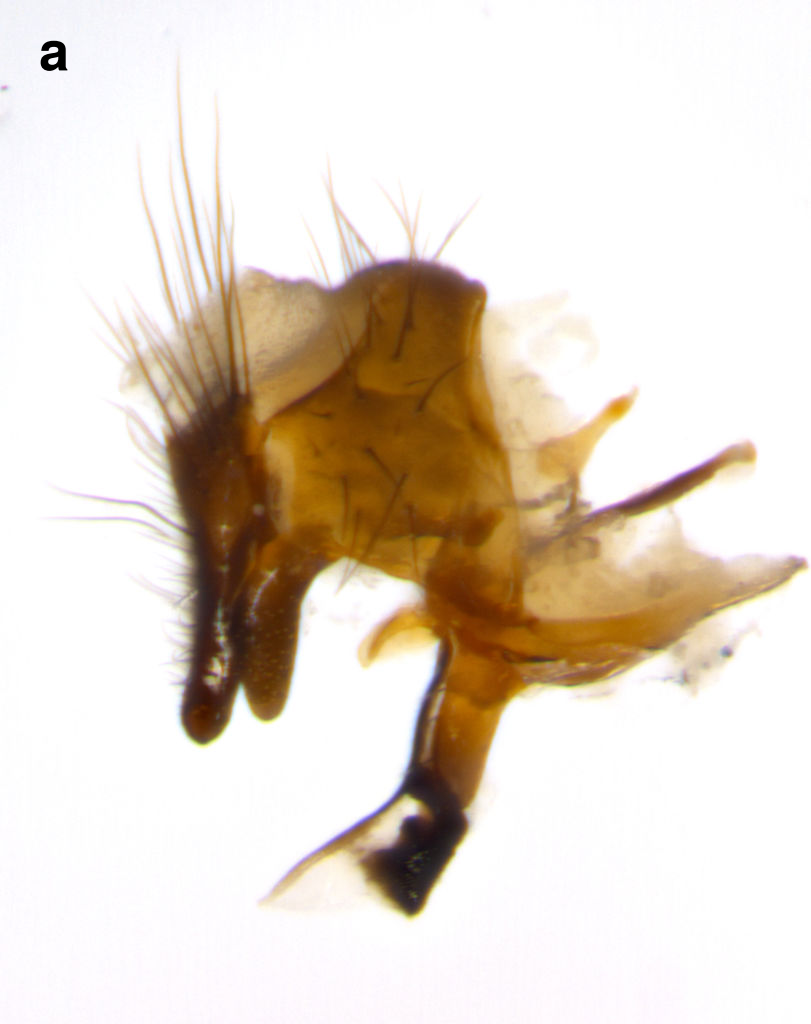
lateral view

**Figure 2b.**
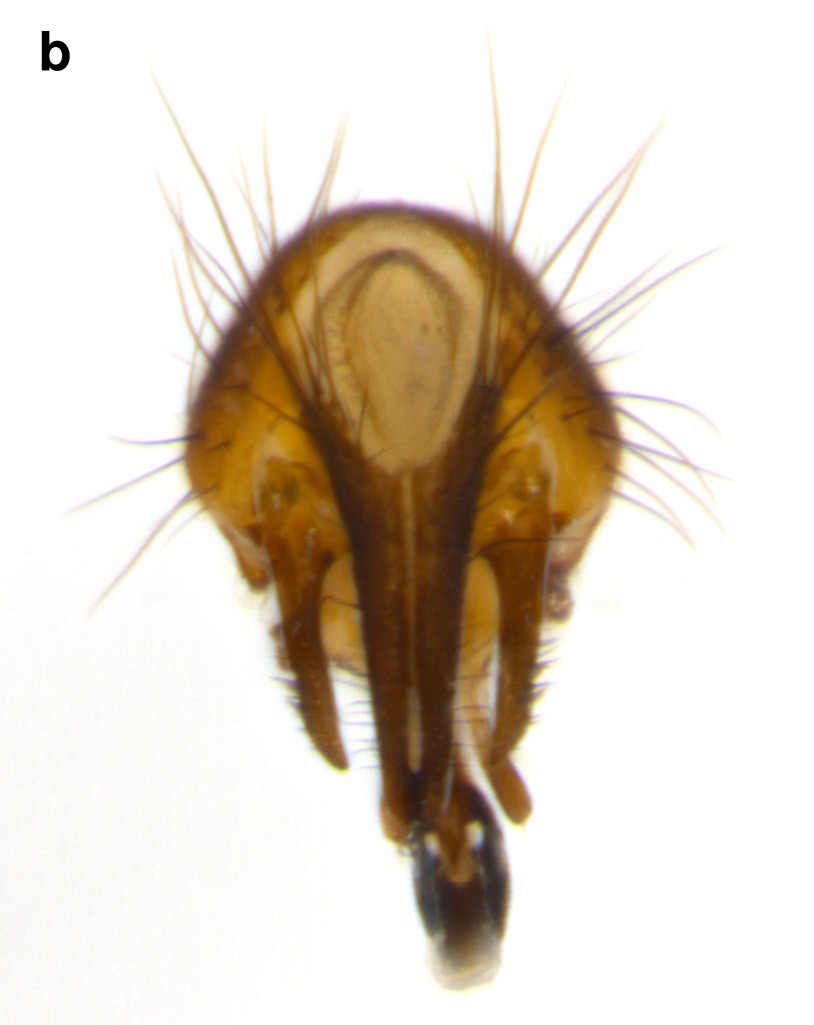
dorsal view

**Figure 2c.**
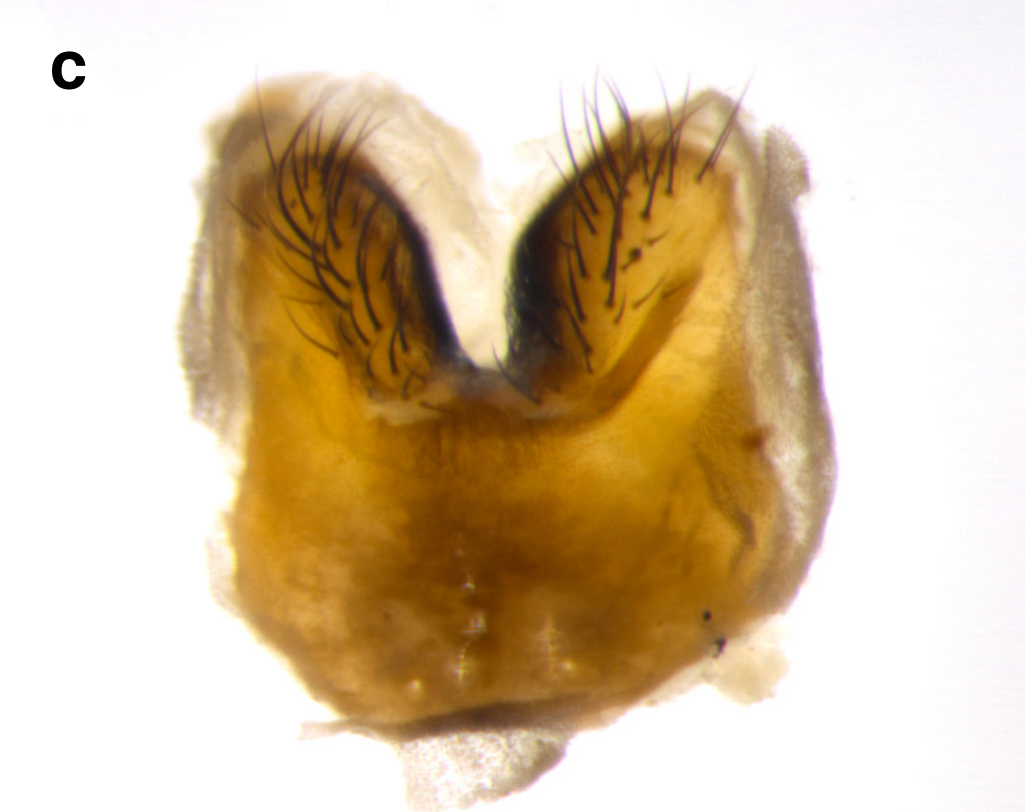
sternite 5 in ventral view

**Figure 3a.**
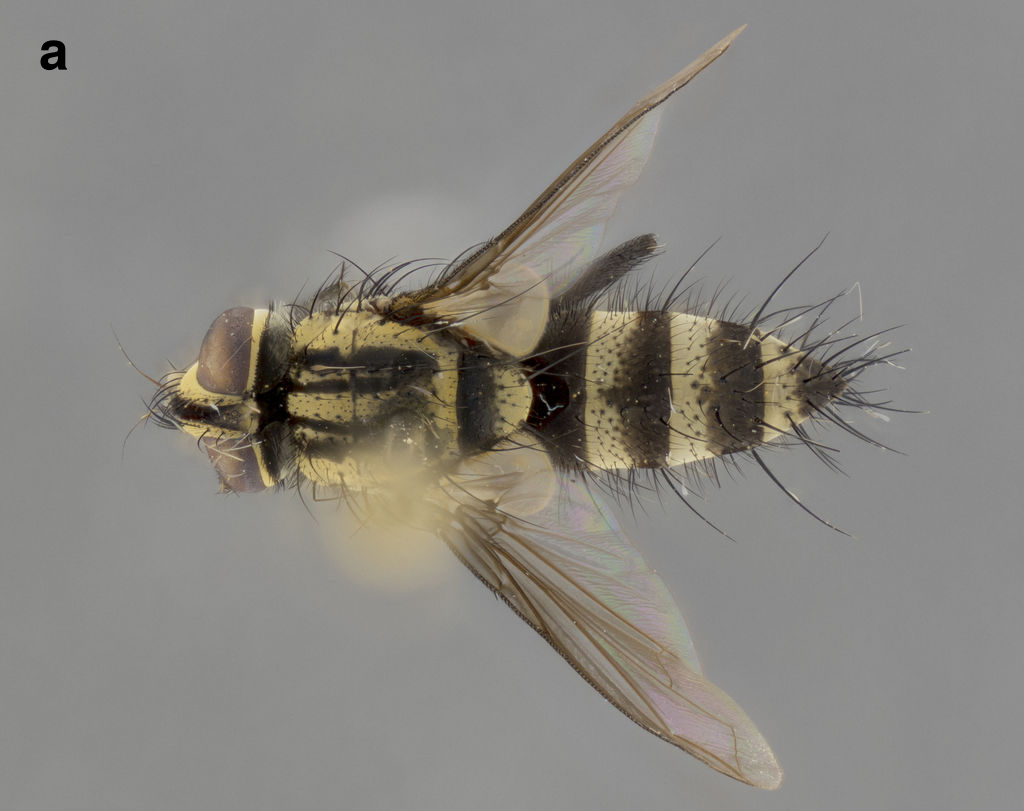
habitus in dorsal view

**Figure 3b.**
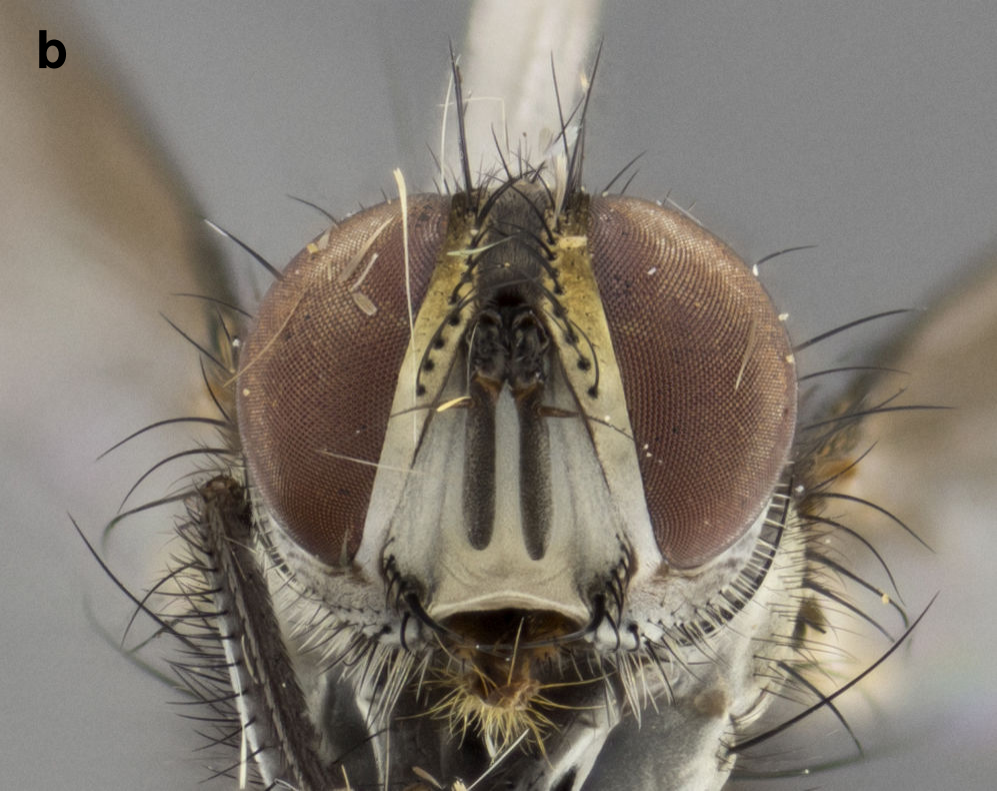
head in frontal view

**Figure 3c.**
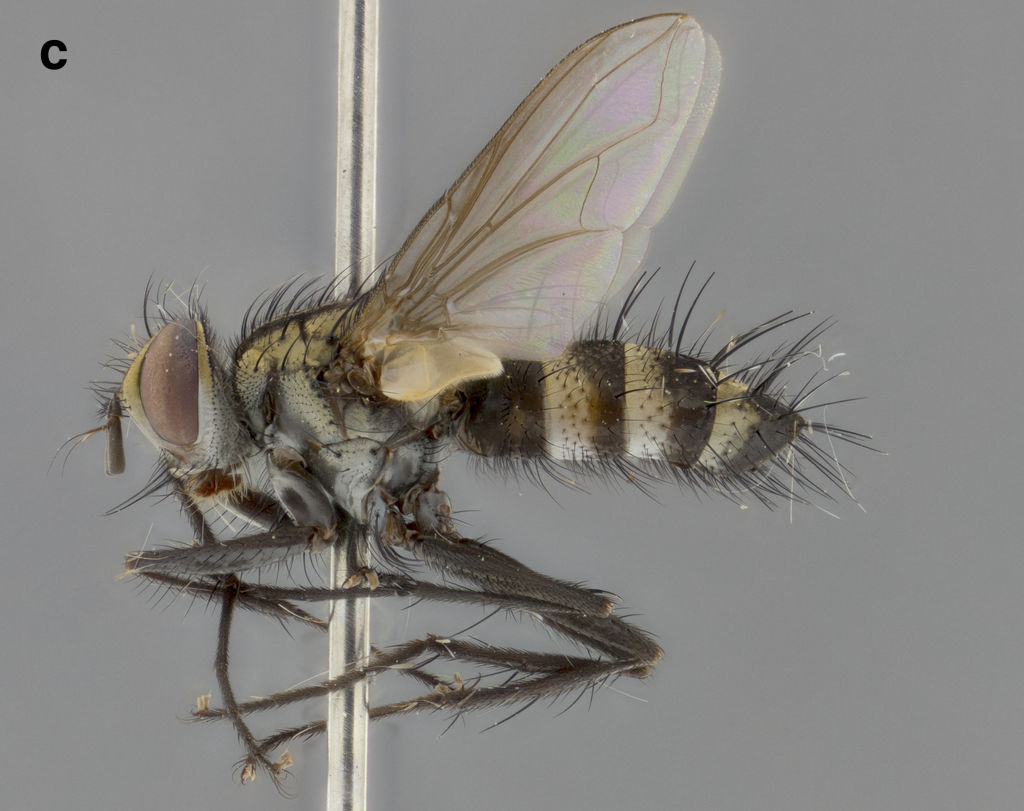
habitus in lateral view

**Figure 4a.**
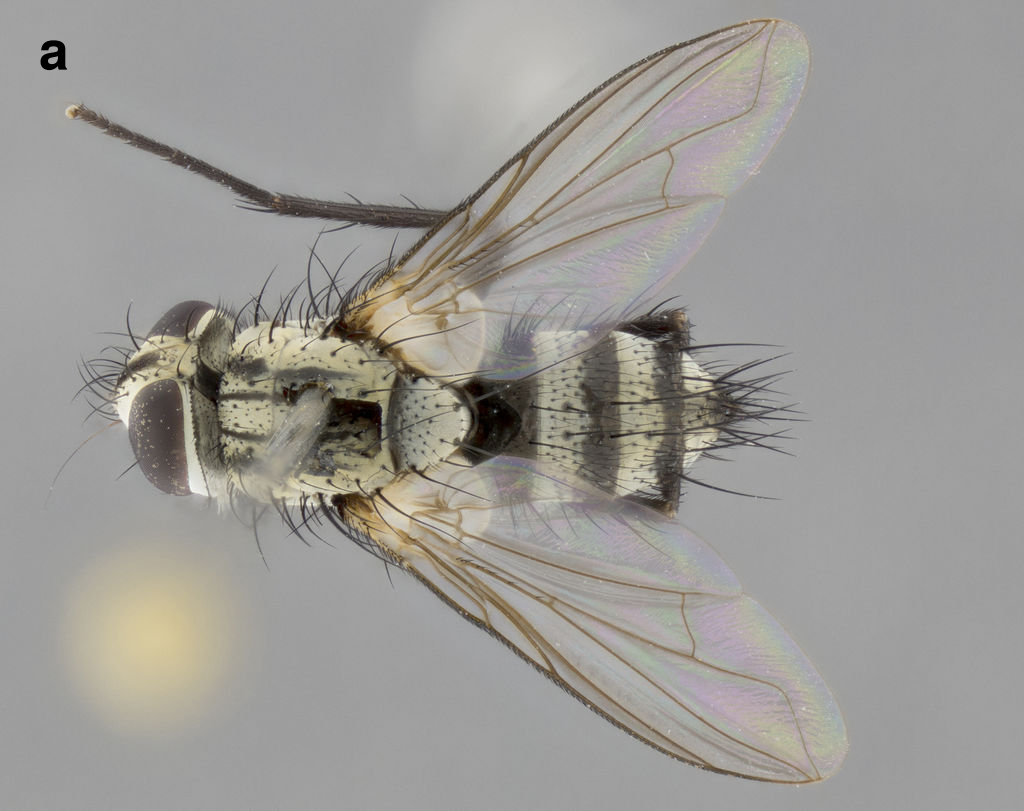
habitus in dorsal view

**Figure 4b.**
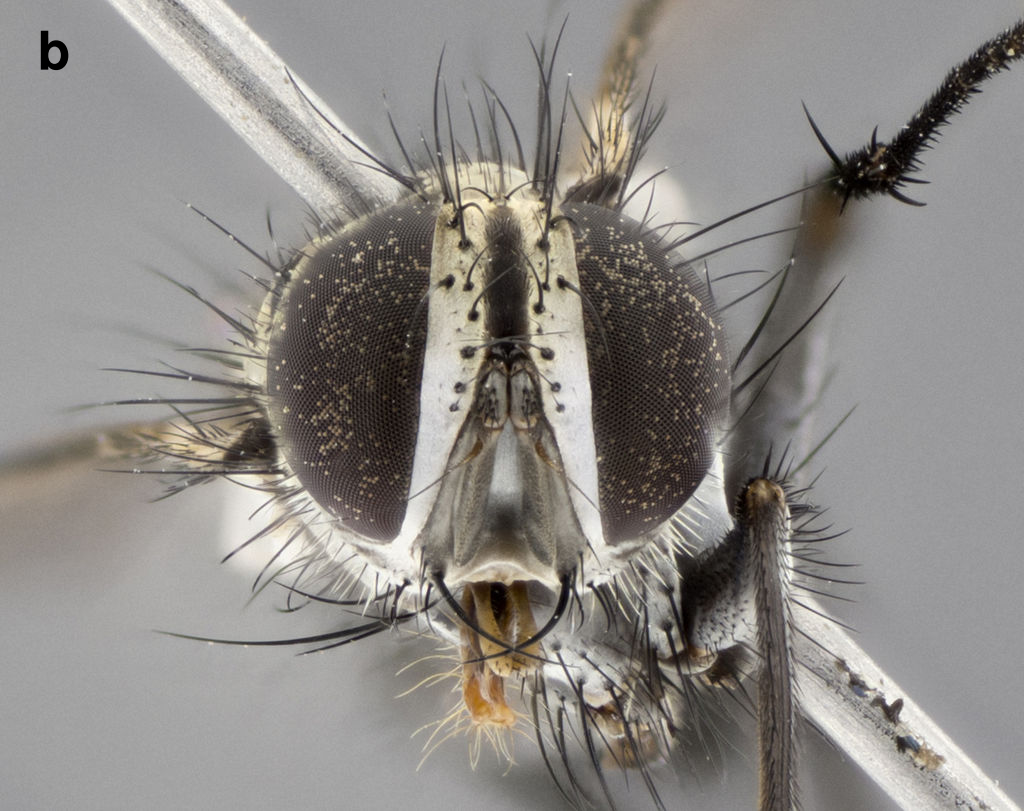
head in frontal view

**Figure 4c.**
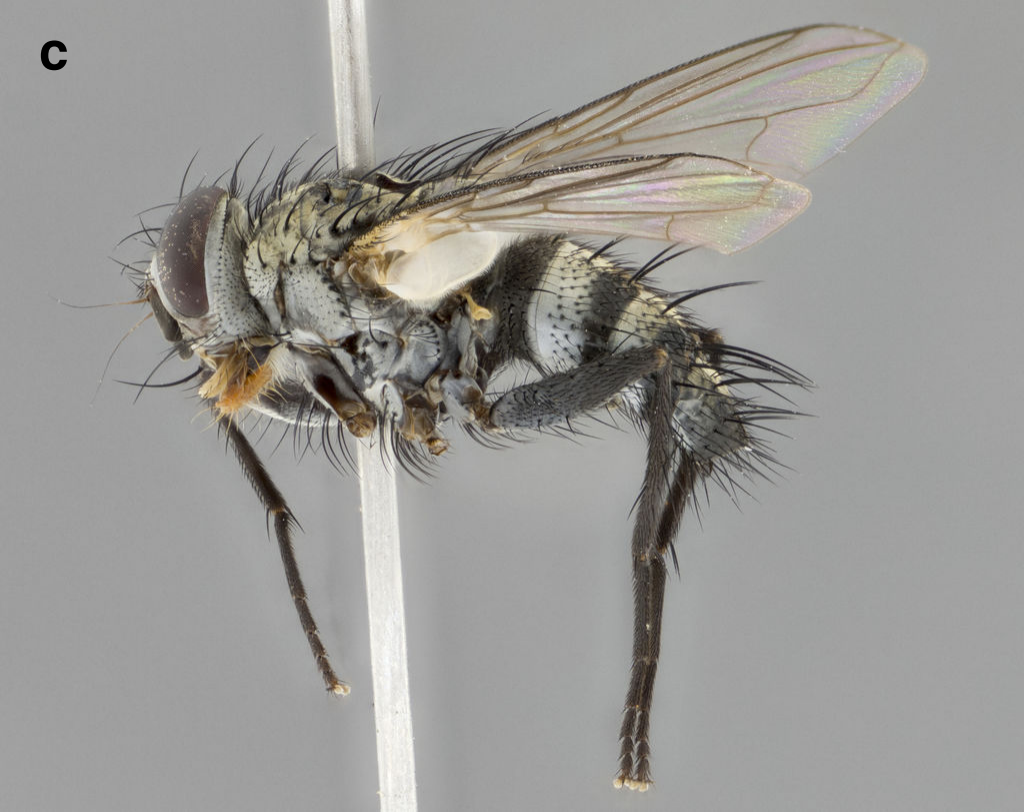
habitus in lateral view

**Figure 5a.**
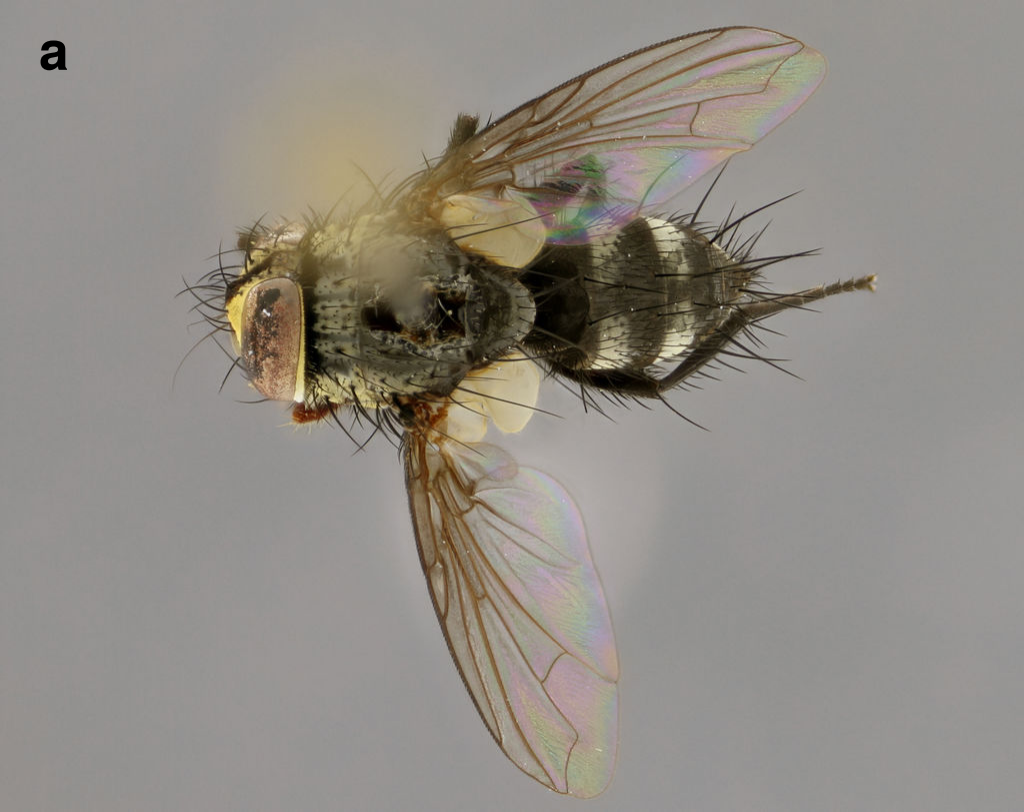
habitus in dorsal view

**Figure 5b.**
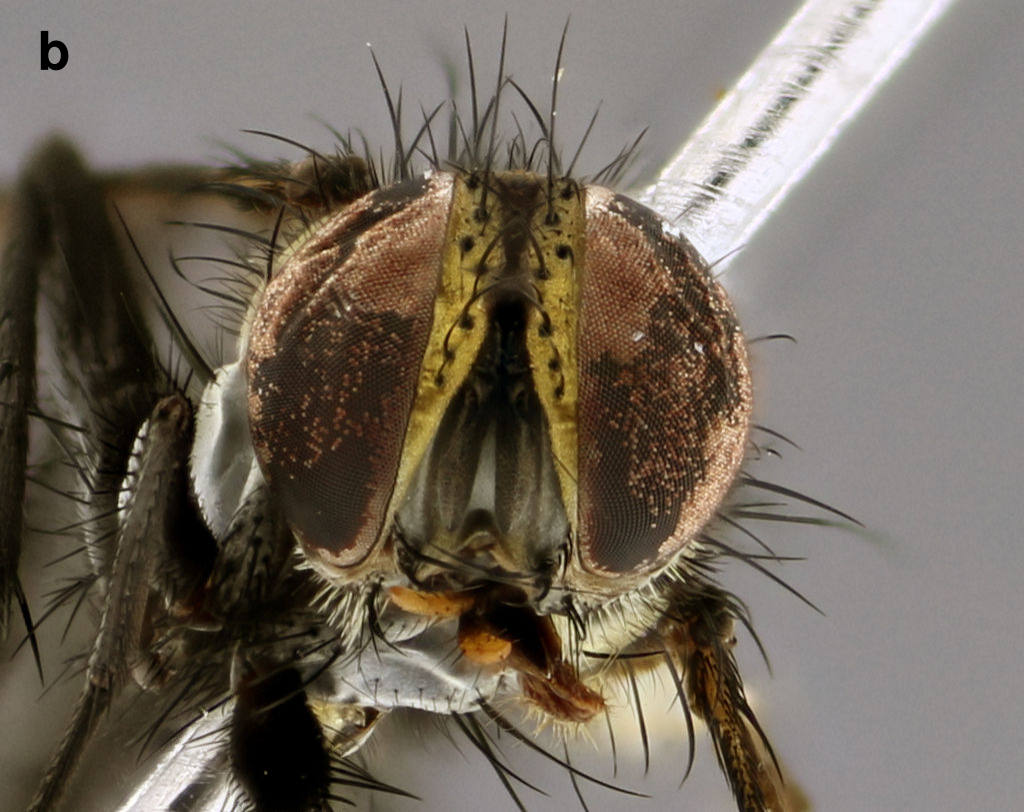
head in frontal view

**Figure 5c.**
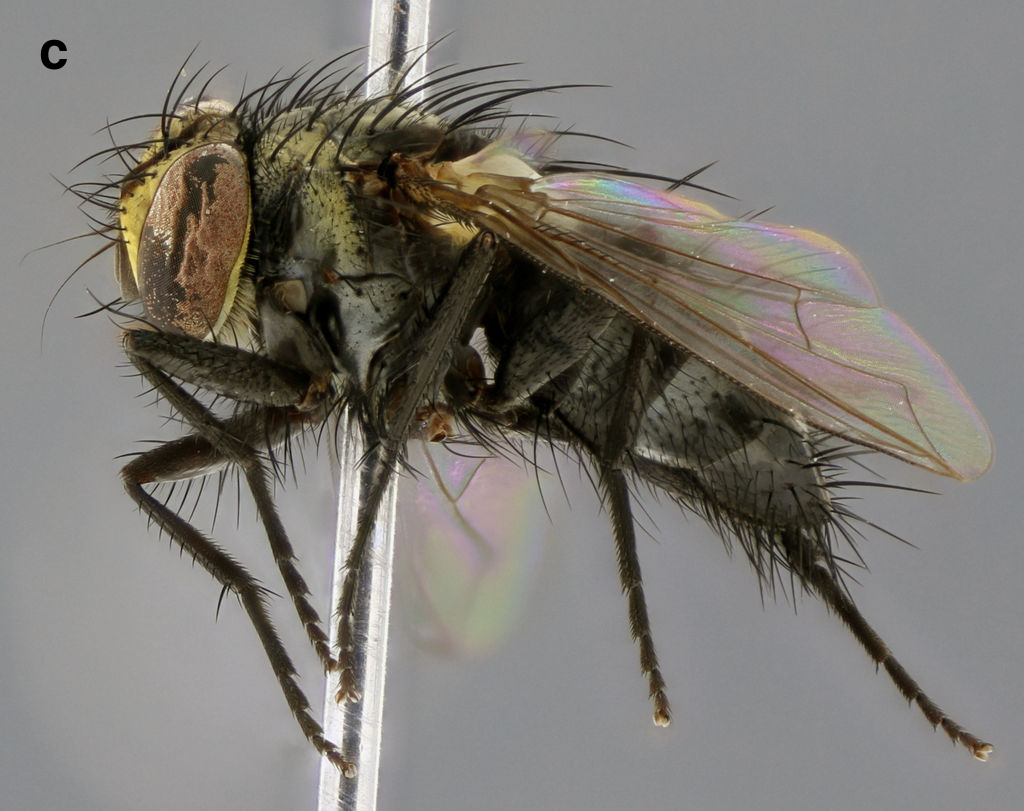
habitus in lateral view

**Figure 6a.**
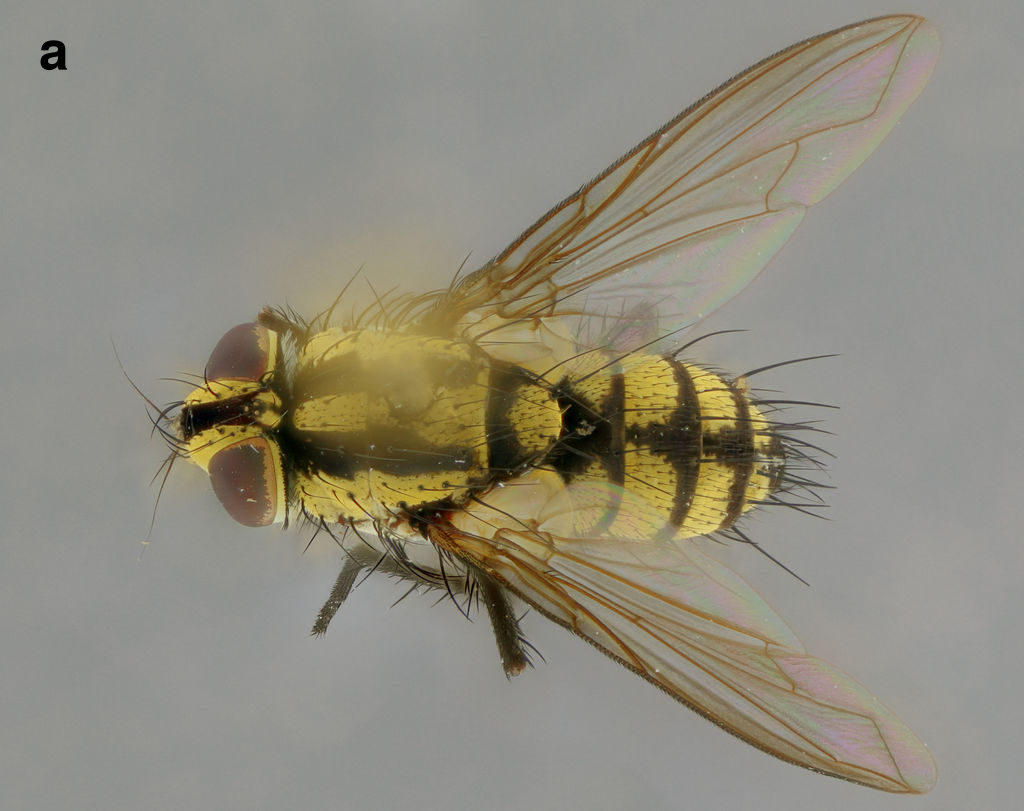
habitus in dorsal view

**Figure 6b.**
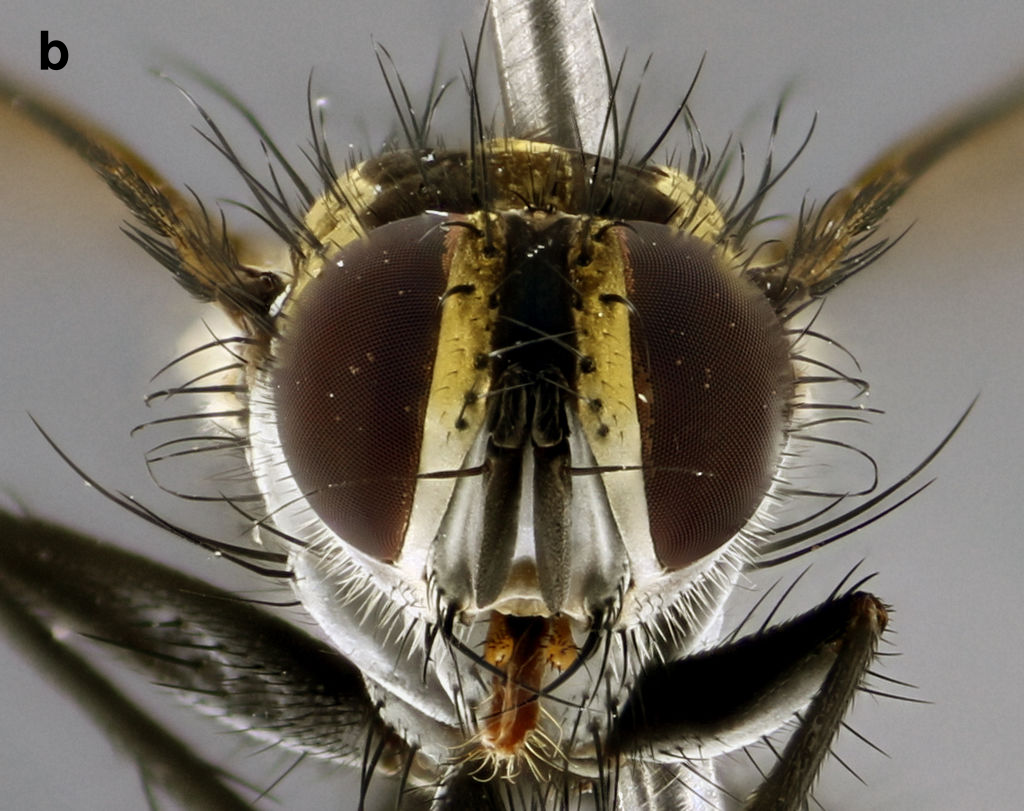
head in frontal view

**Figure 6c.**
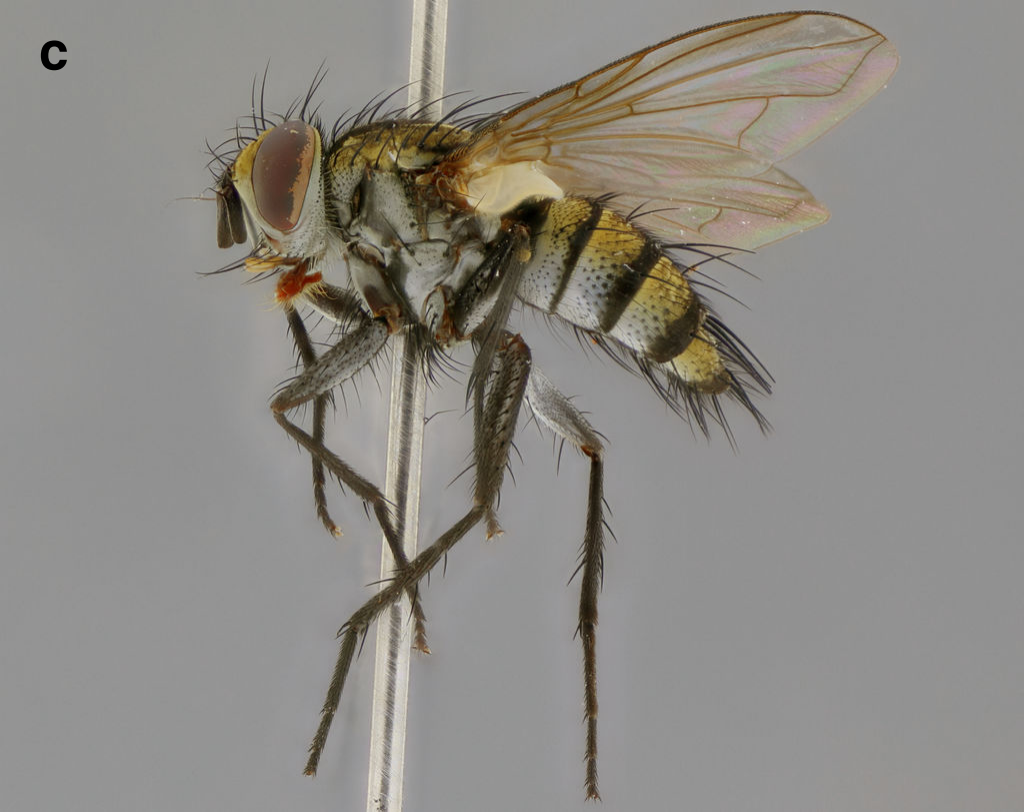
habitus in lateral view

**Figure 7a.**
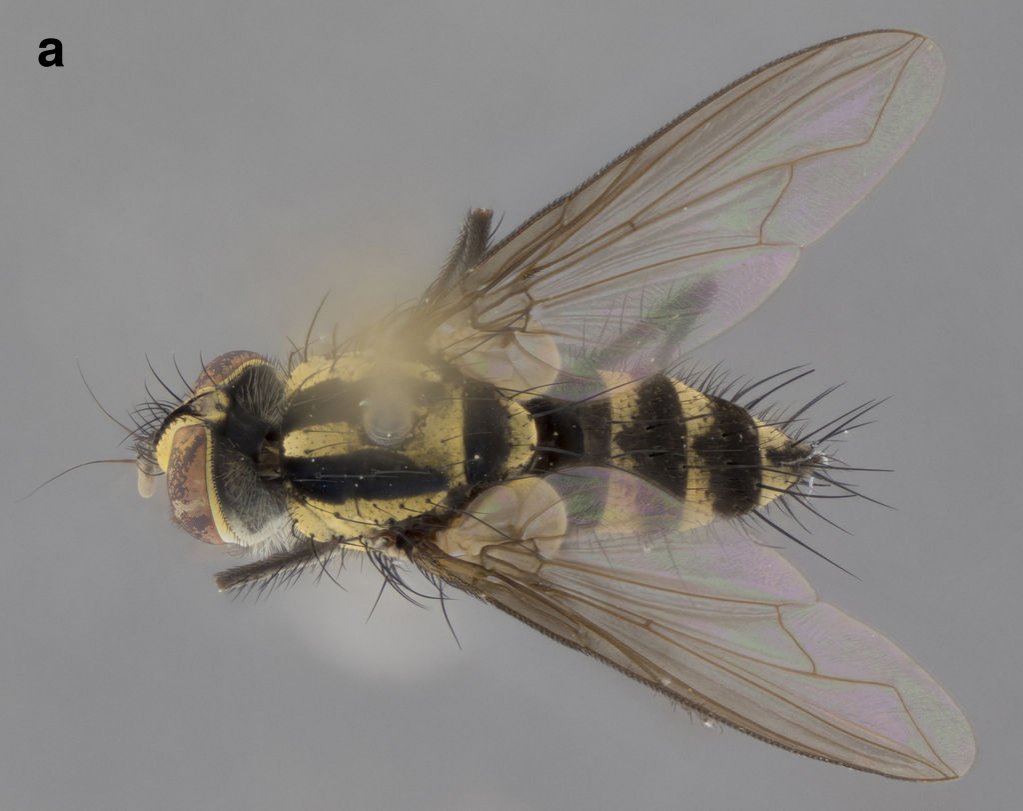
habitus in dorsal view

**Figure 7b.**
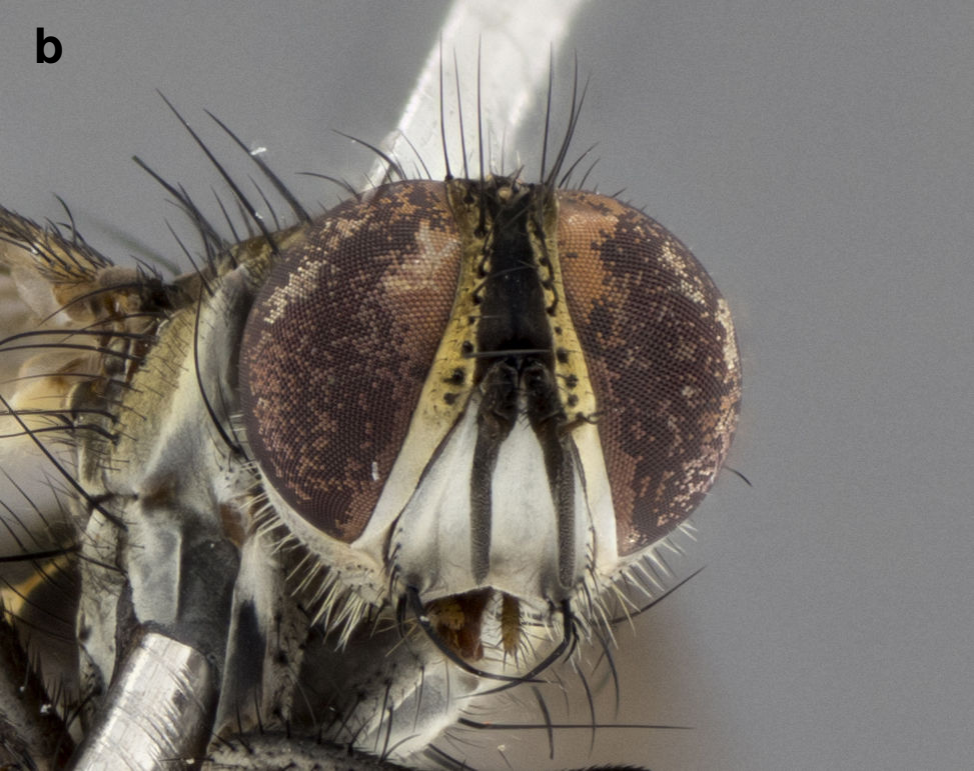
head in frontal view

**Figure 7c.**
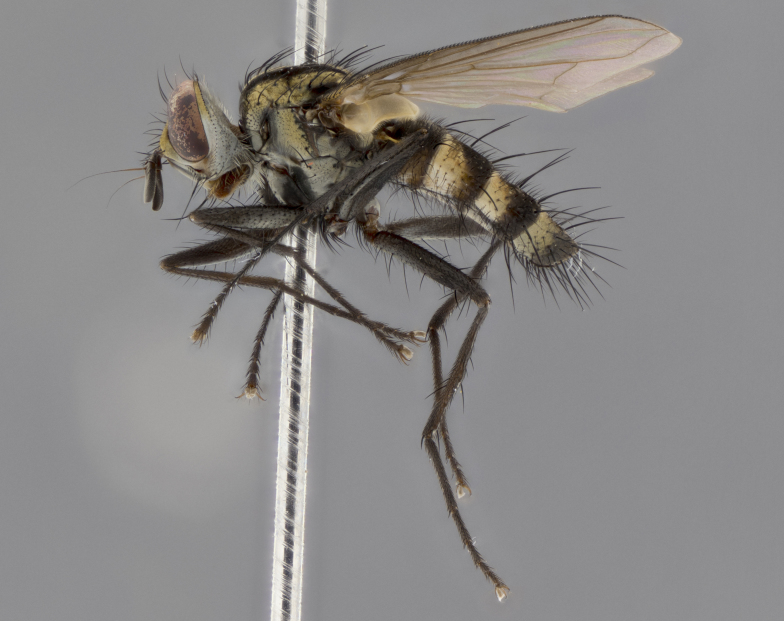
habitus in lateral view

**Figure 7d.**
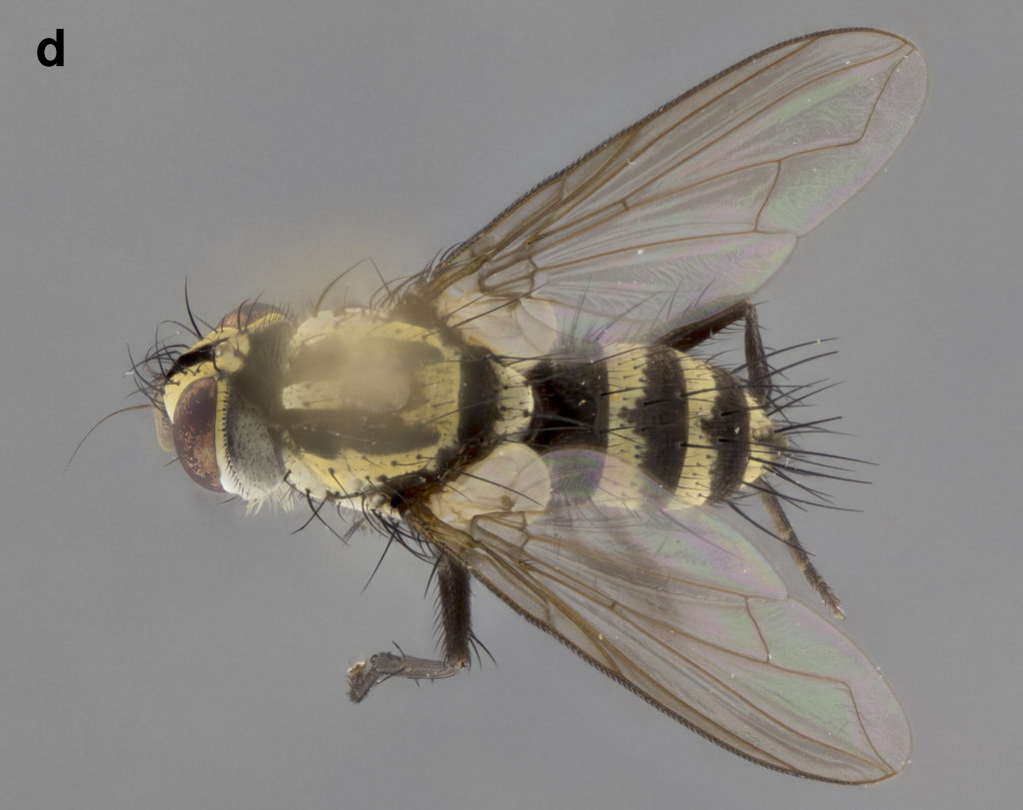
habitus in dorsal view

**Figure 7e.**
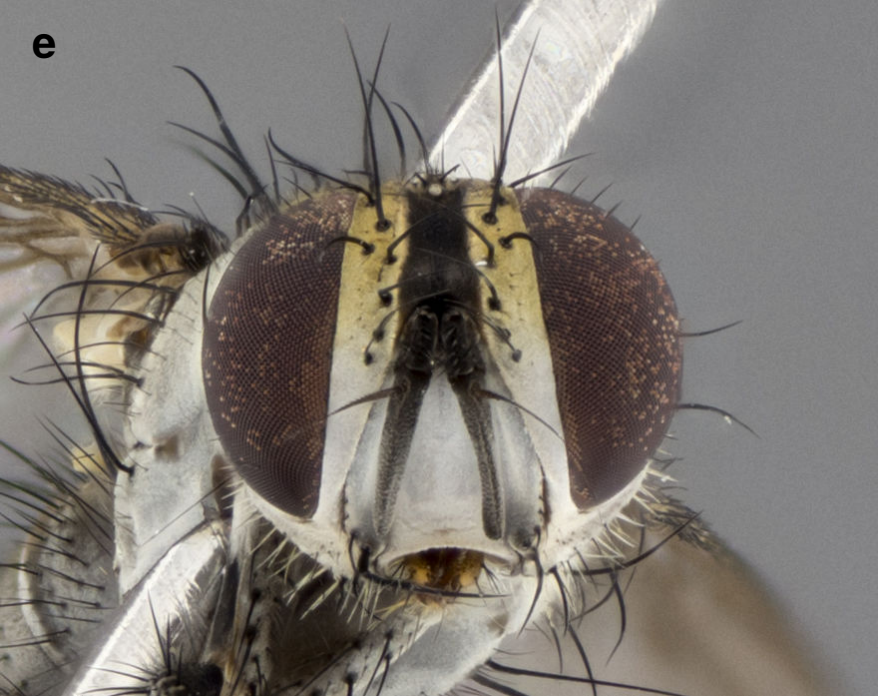
head in frontal view

**Figure 7f.**
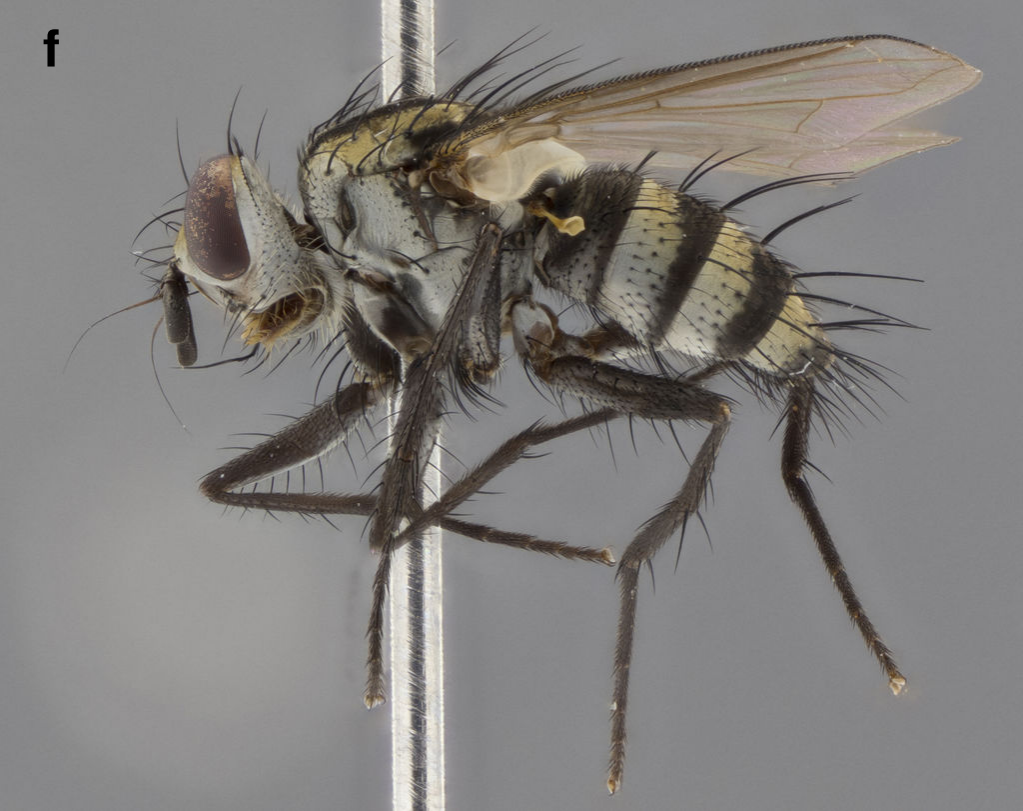
habitus in lateral view

**Figure 8a.**
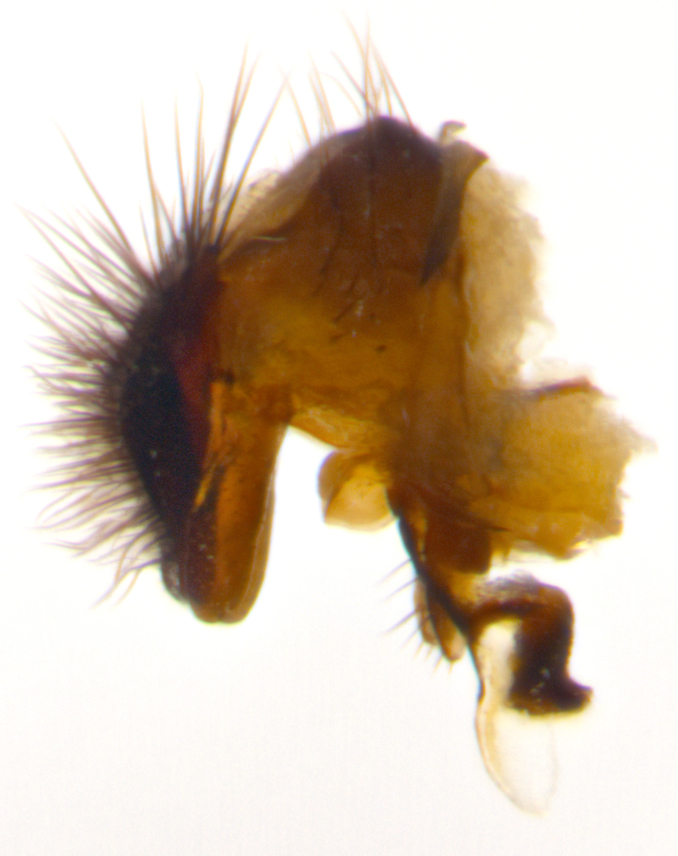
lateral view

**Figure 8b.**
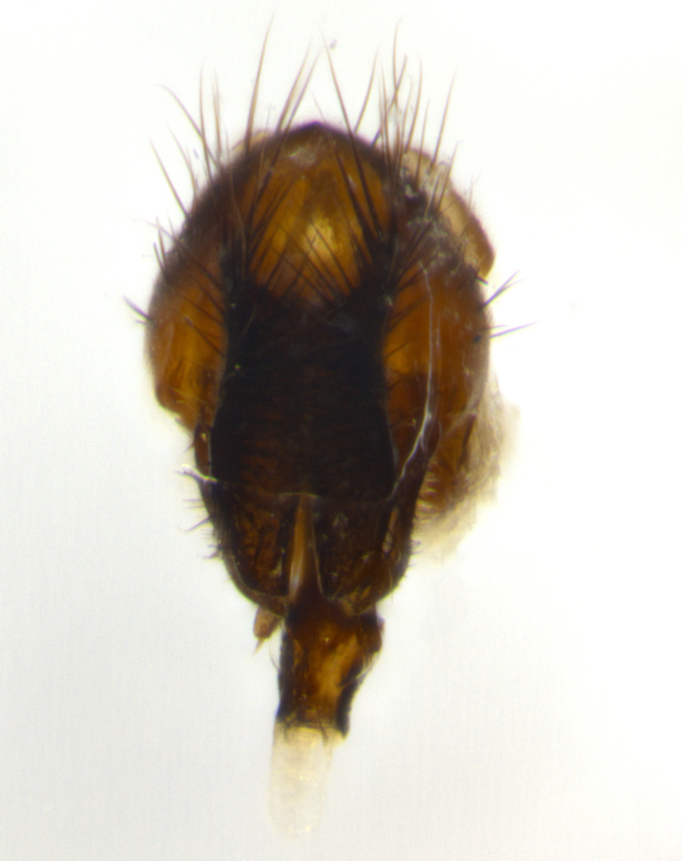
dorsal view

**Figure 8c.**
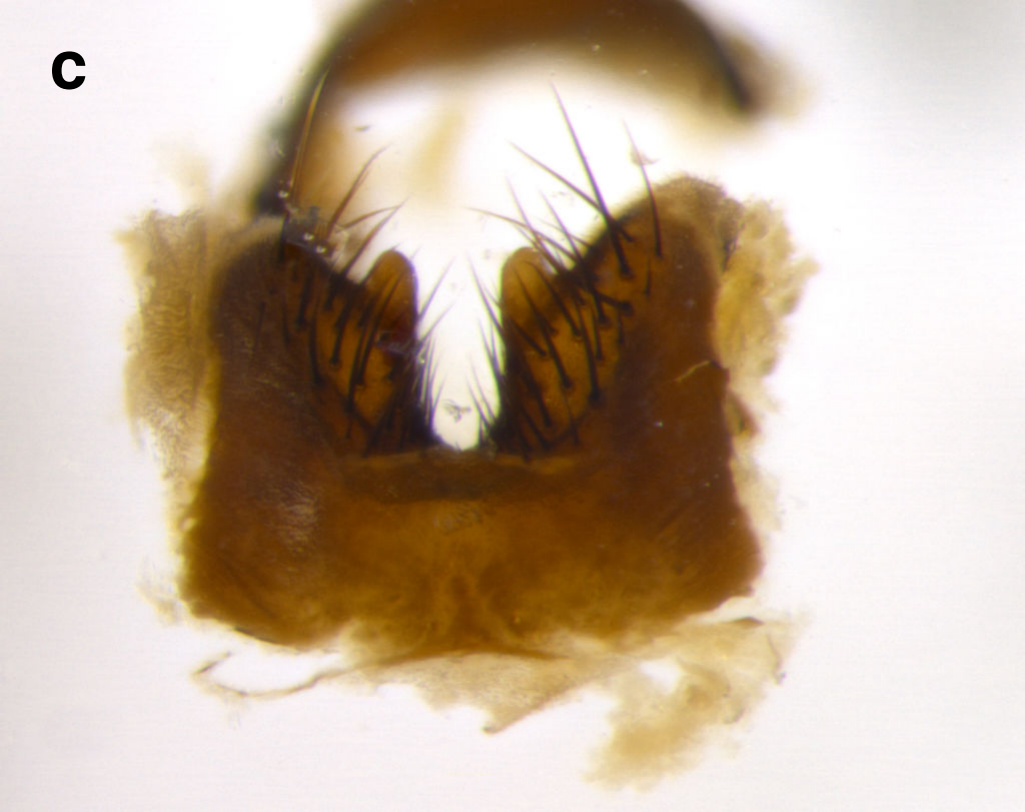
sternite 5 in ventral view

**Figure 9a.**
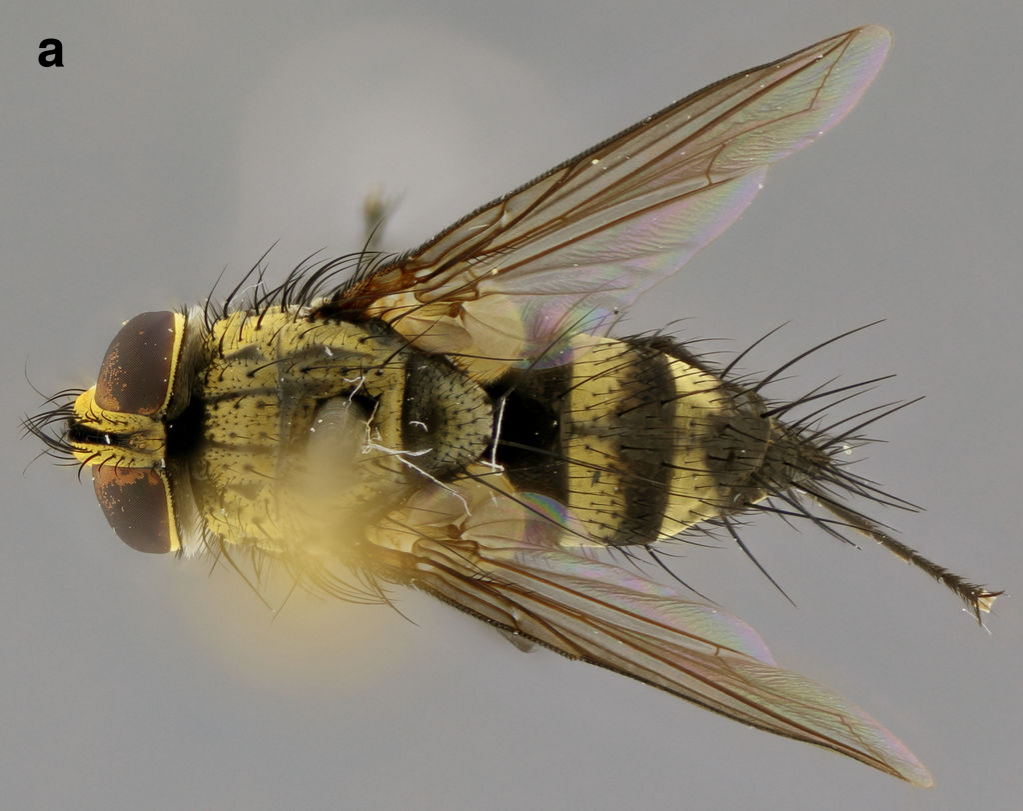
habitus in dorsal view

**Figure 9b.**
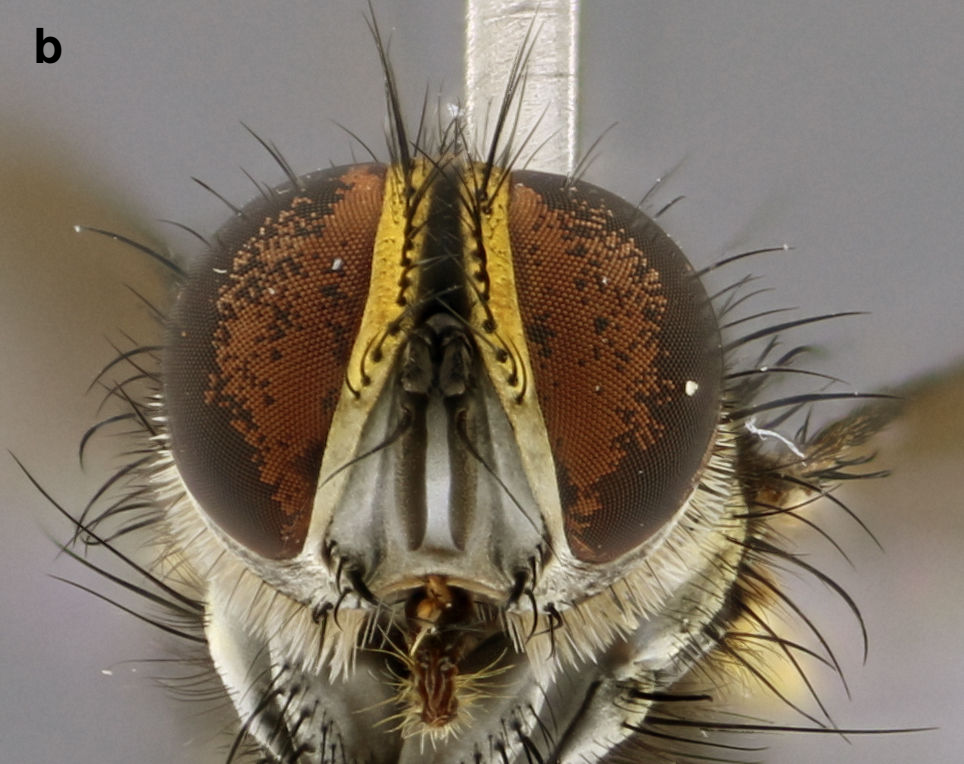
head in frontal view

**Figure 9c.**
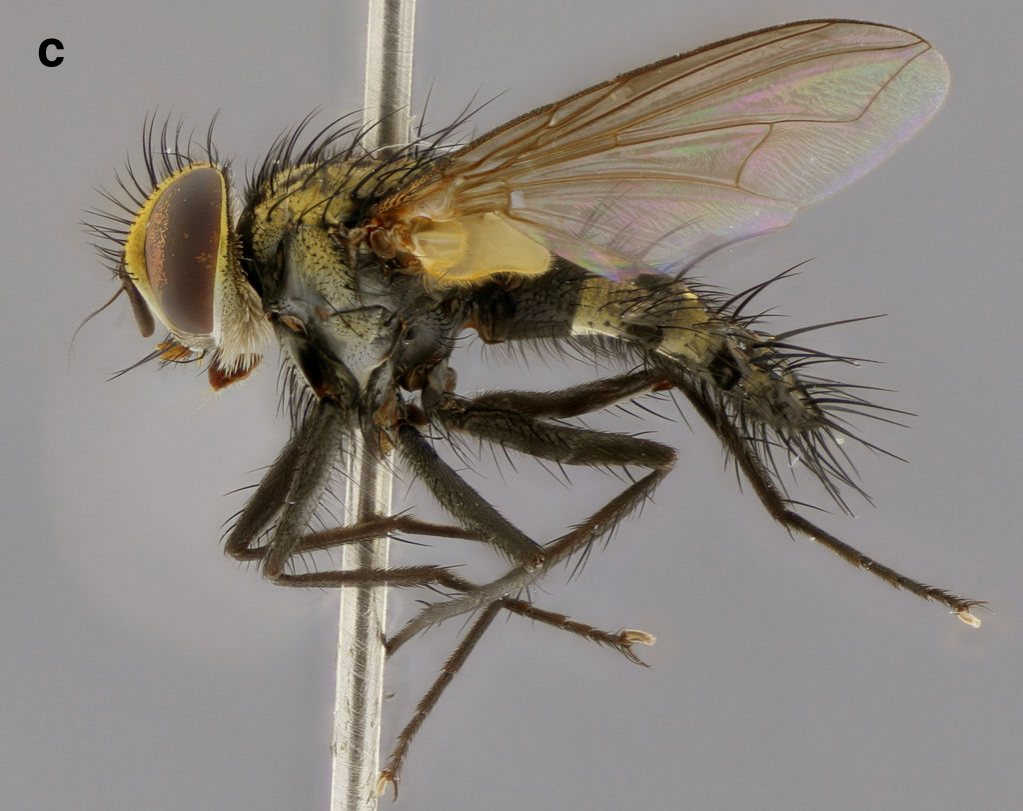
habitus in lateral view

**Figure 10a.**
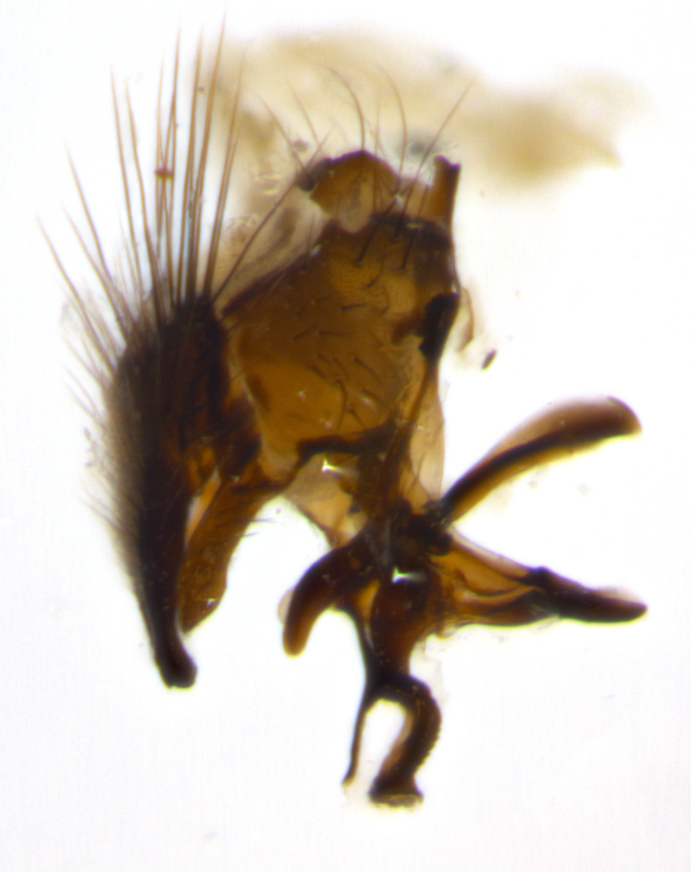
lateral view

**Figure 10b.**
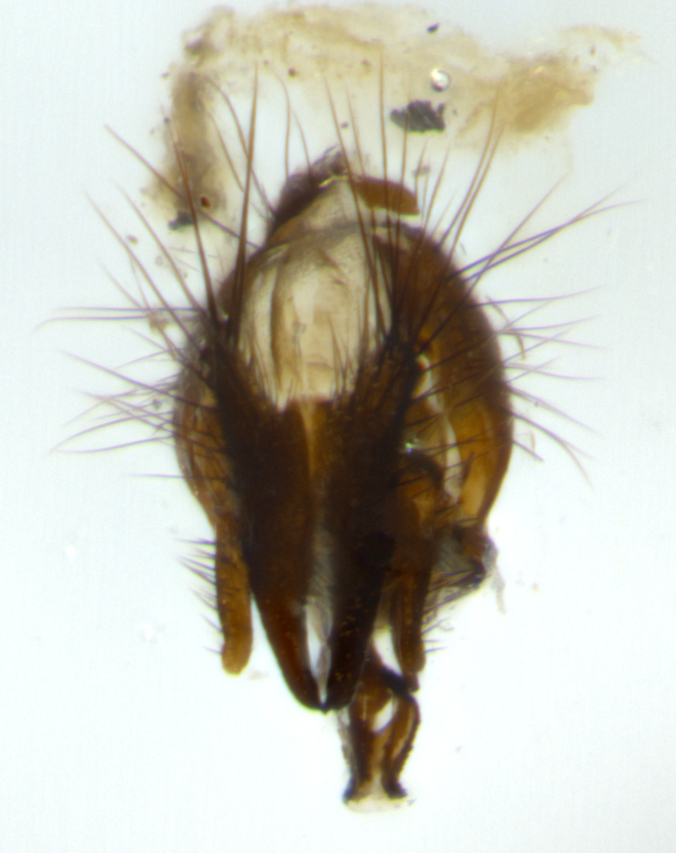
dorsal view

**Figure 10c.**
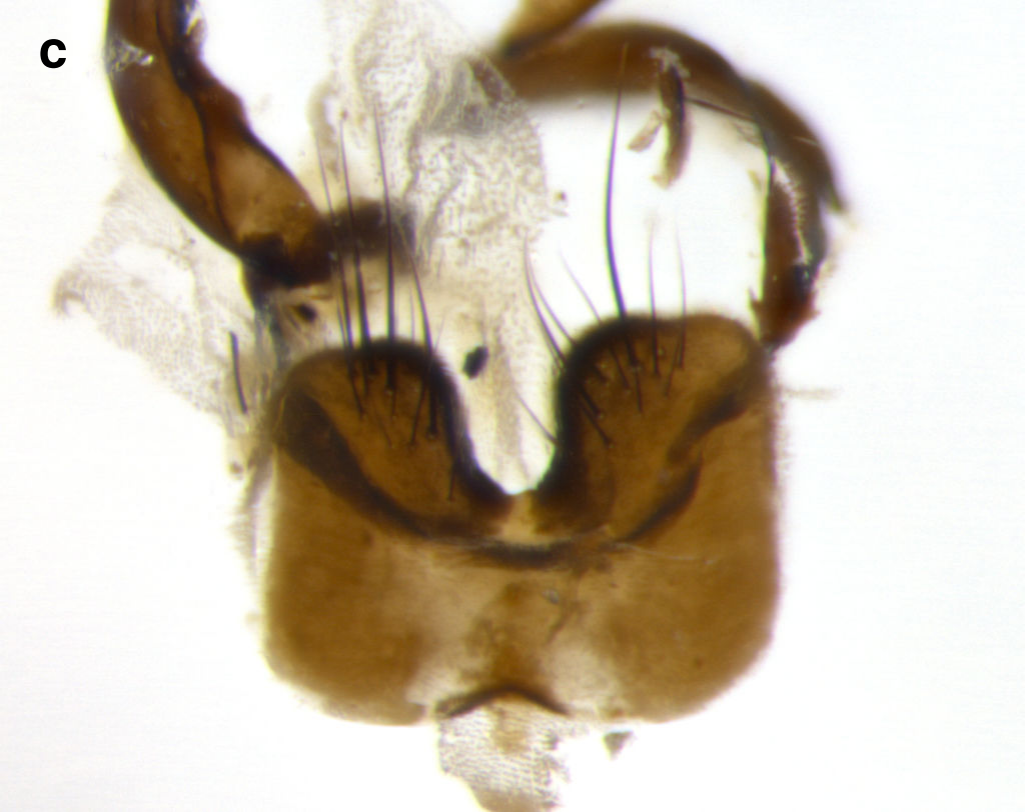
sternite 5 in ventral view

**Figure 11a.**
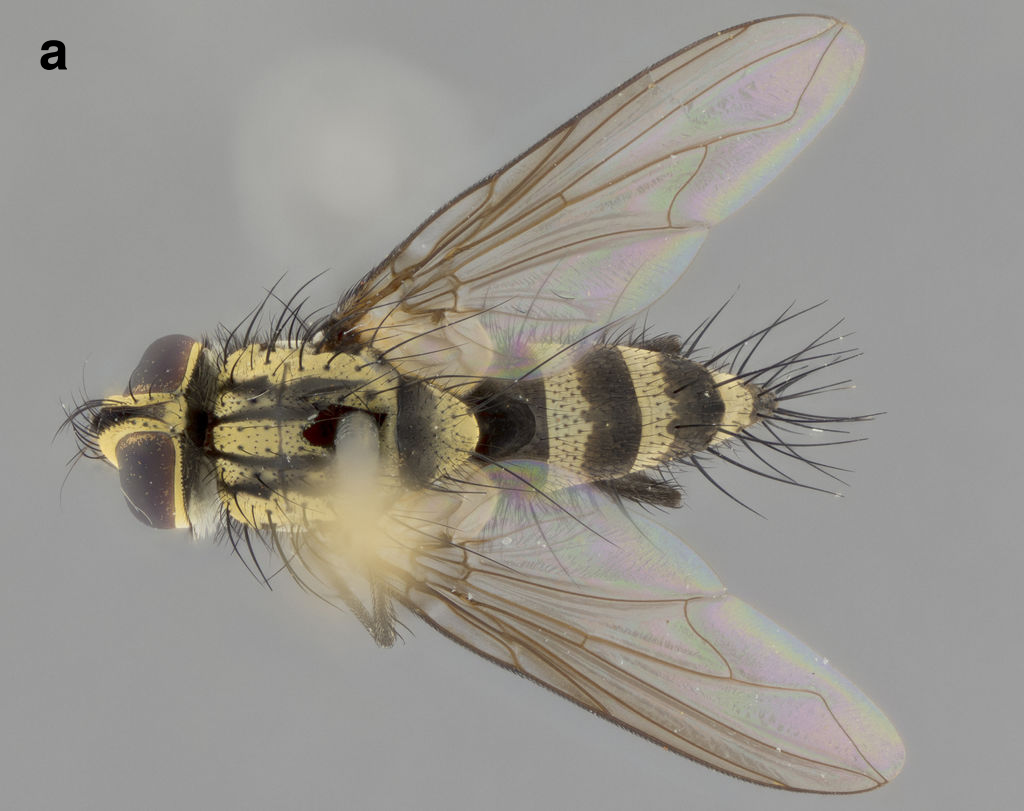
habitus in dorsal view

**Figure 11b.**
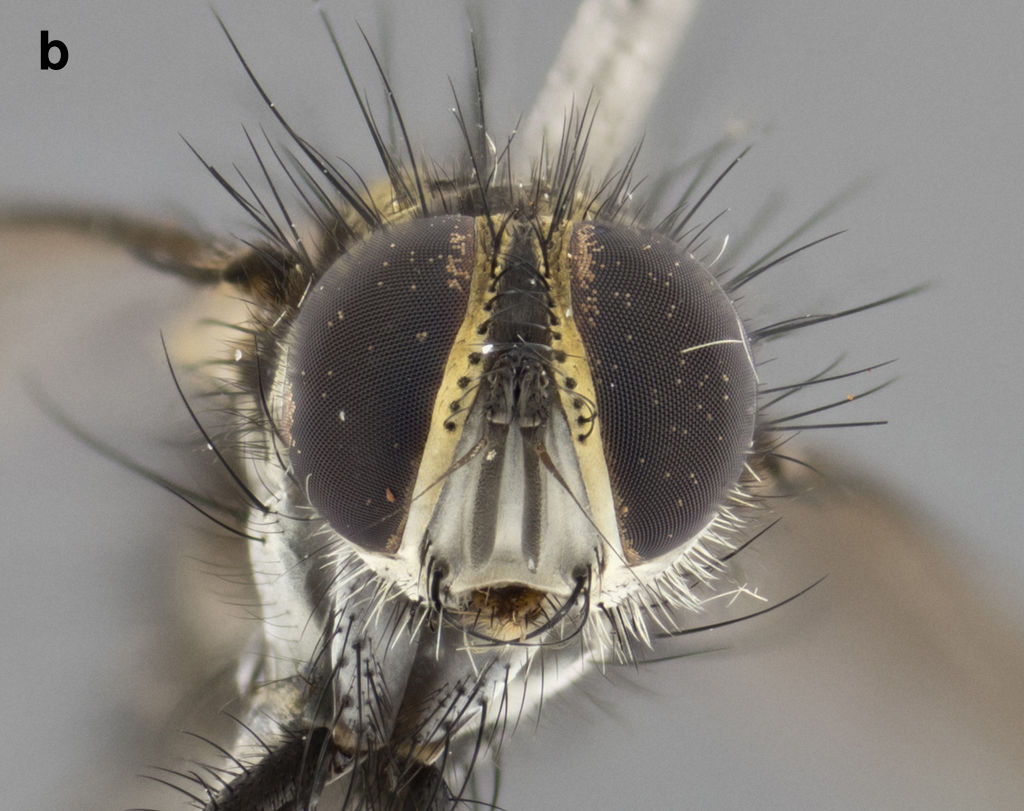
head in frontal view

**Figure 11c.**
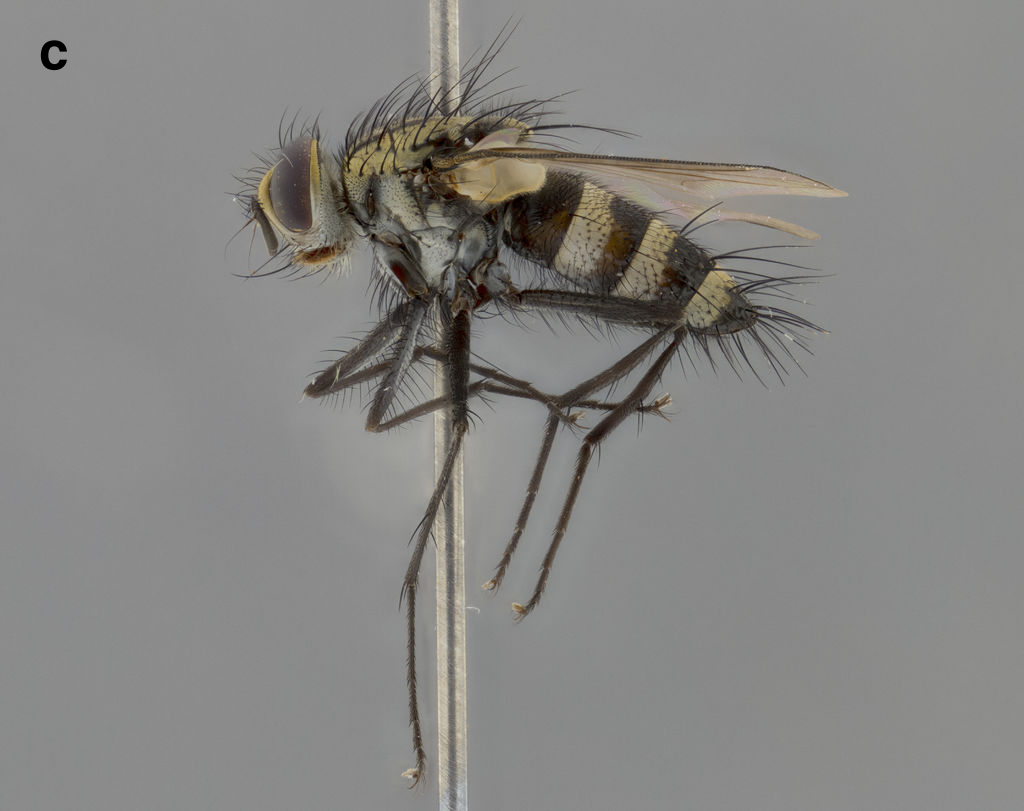
habitus in lateal view

**Figure 11d.**
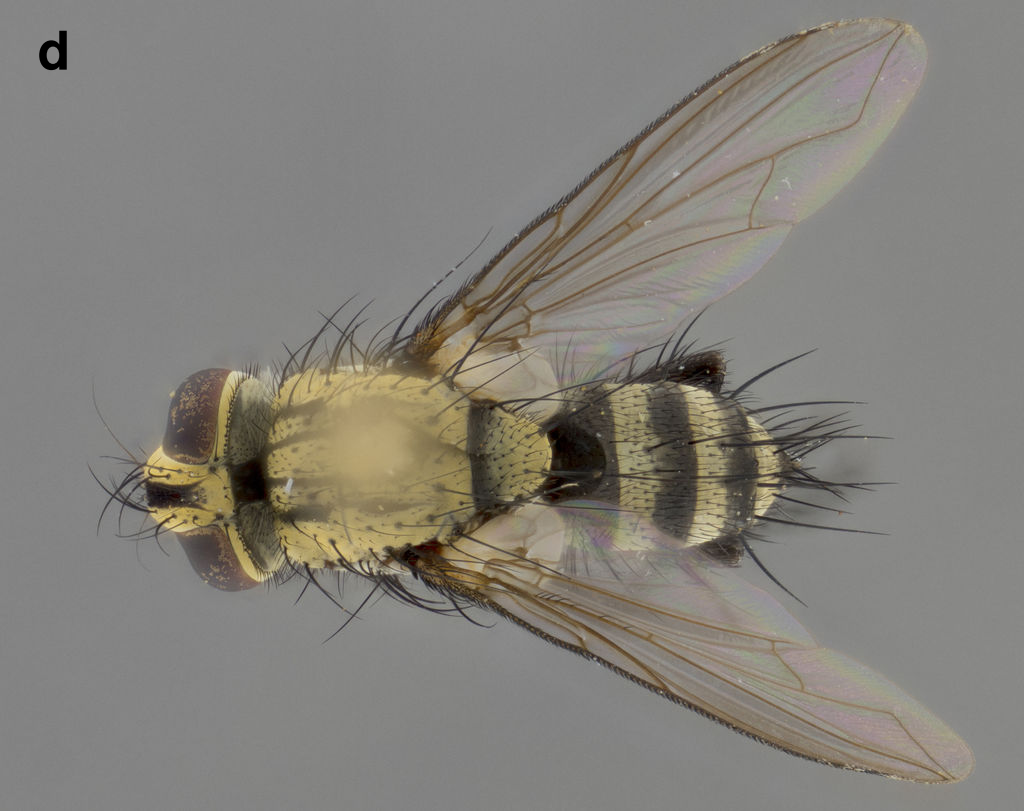
habitus in dorsal view

**Figure 11e.**
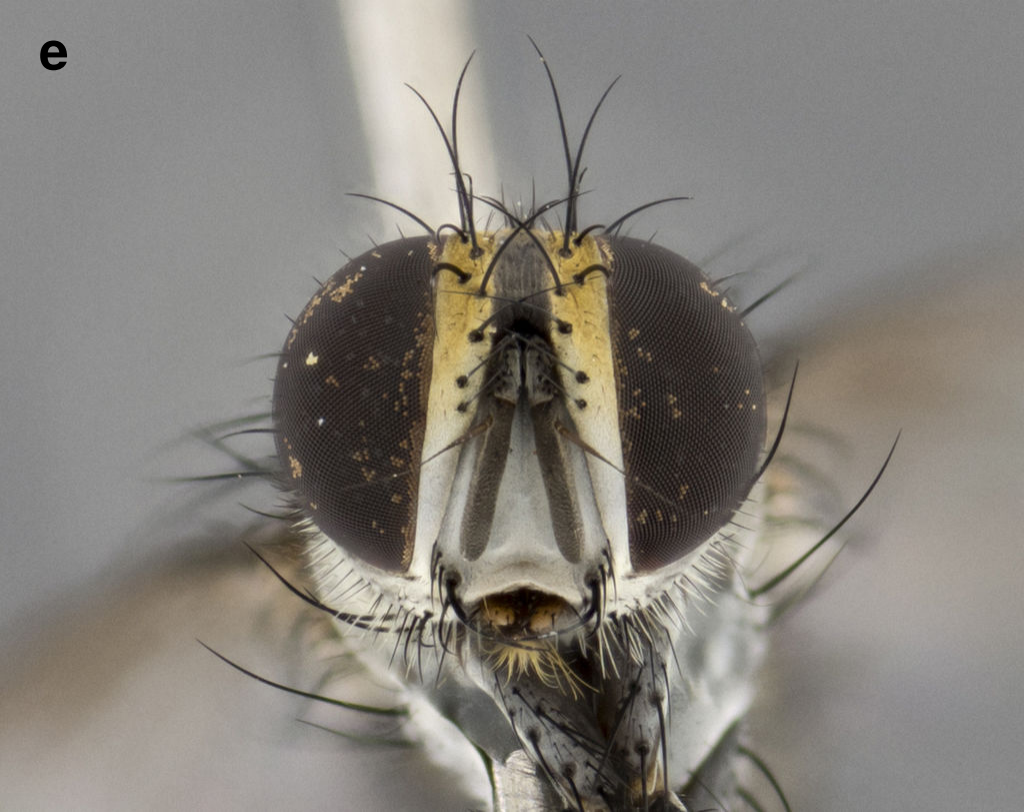
head in frontal view

**Figure 11f.**
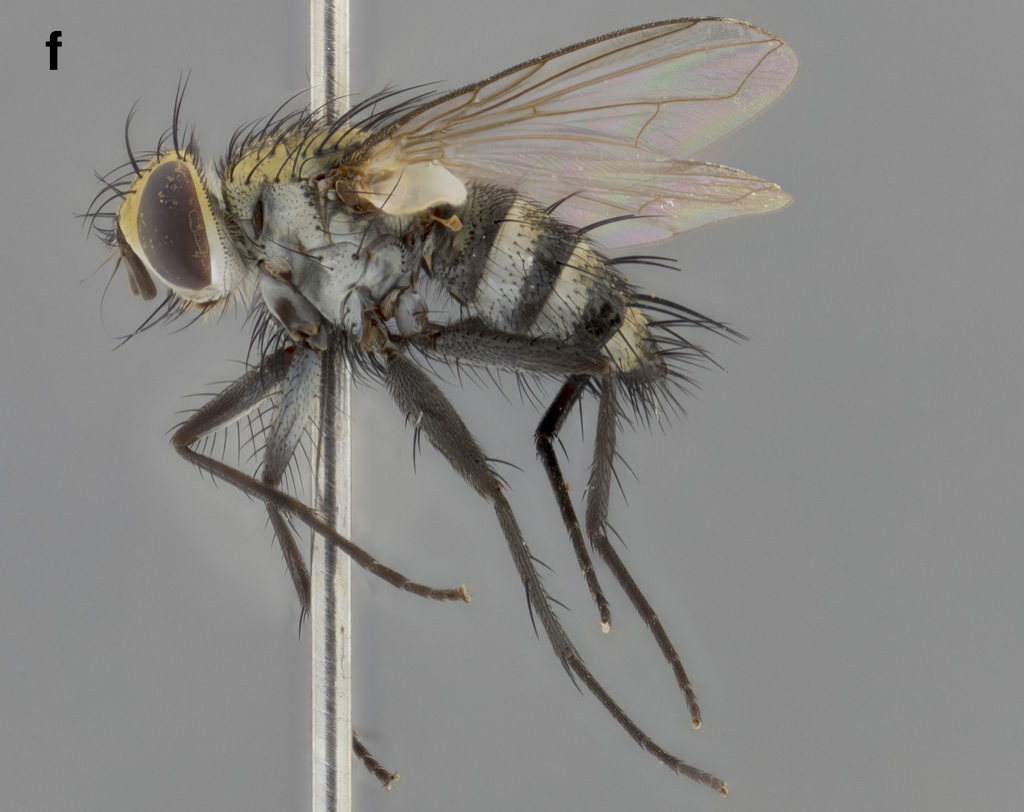
habitus in lateral view

**Figure 12a.**
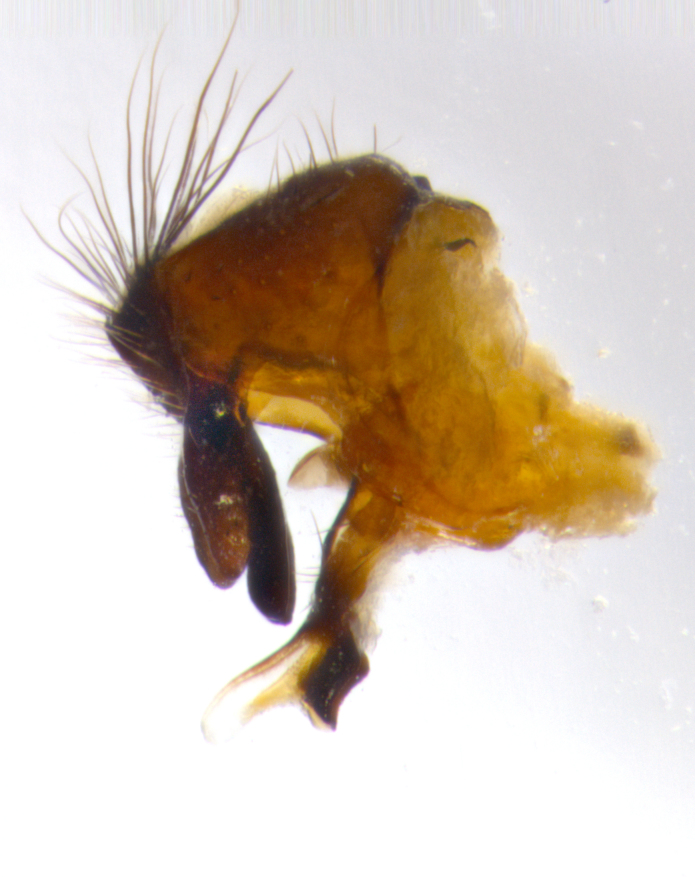
lateral view

**Figure 12b.**
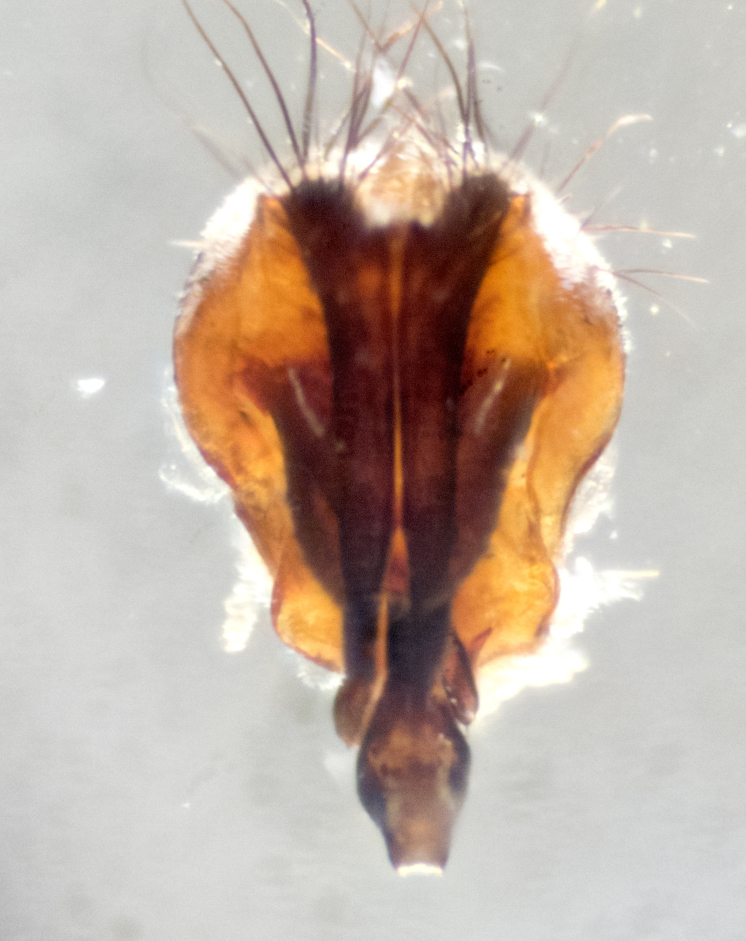
dorsal view

**Figure 12c.**
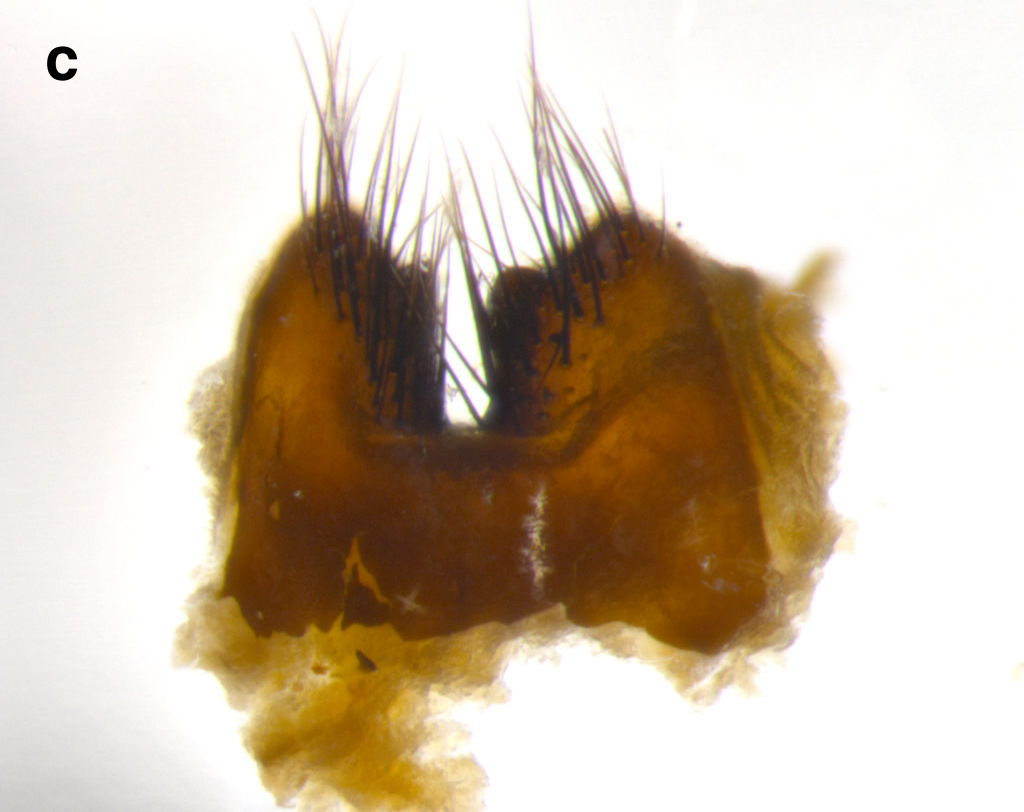
sternite 5 in ventral view

**Figure 13a.**
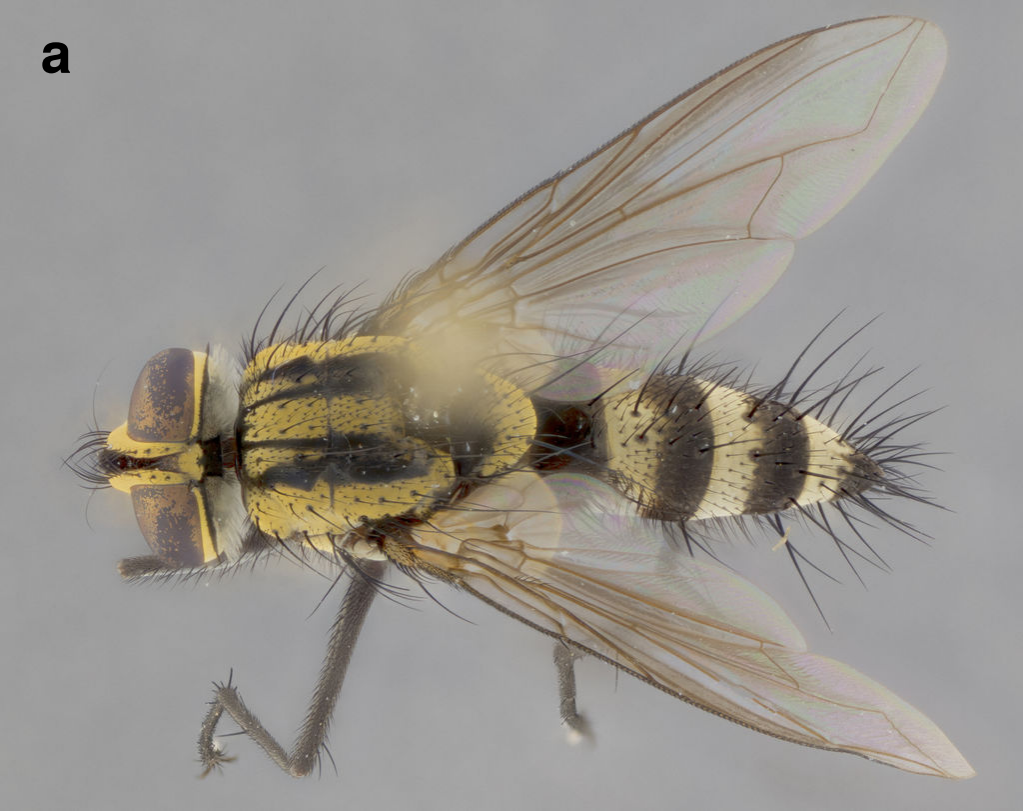
habitus in dorsal view

**Figure 13b.**
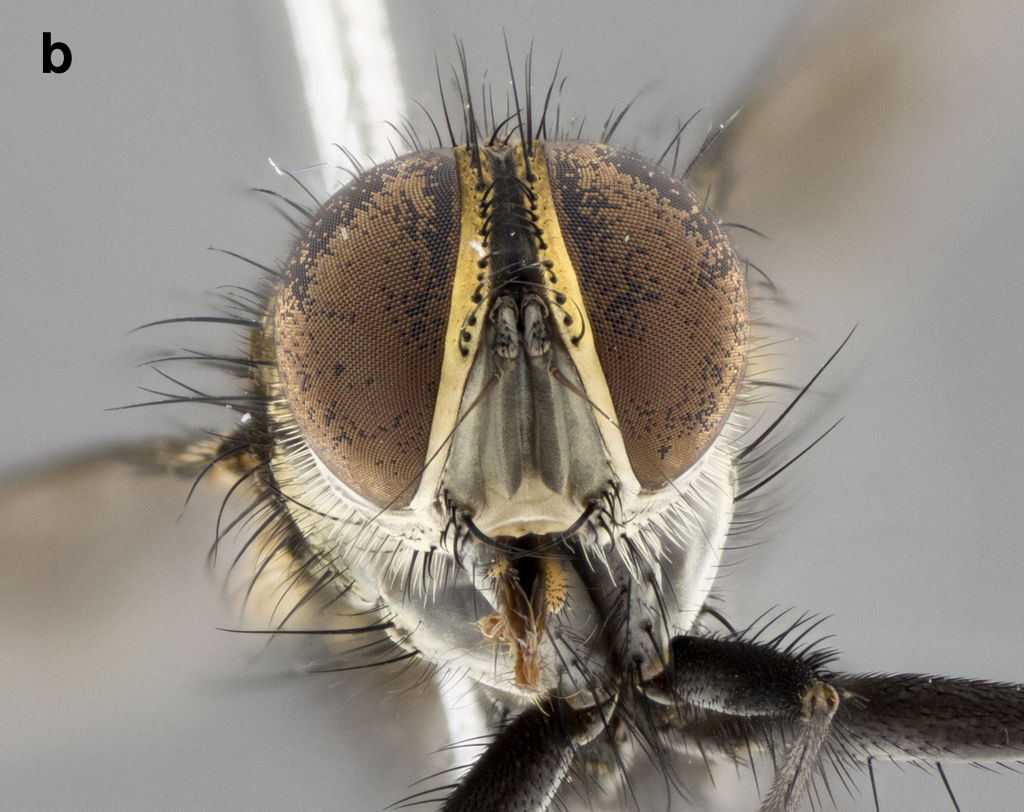
head in frontal view

**Figure 13c.**
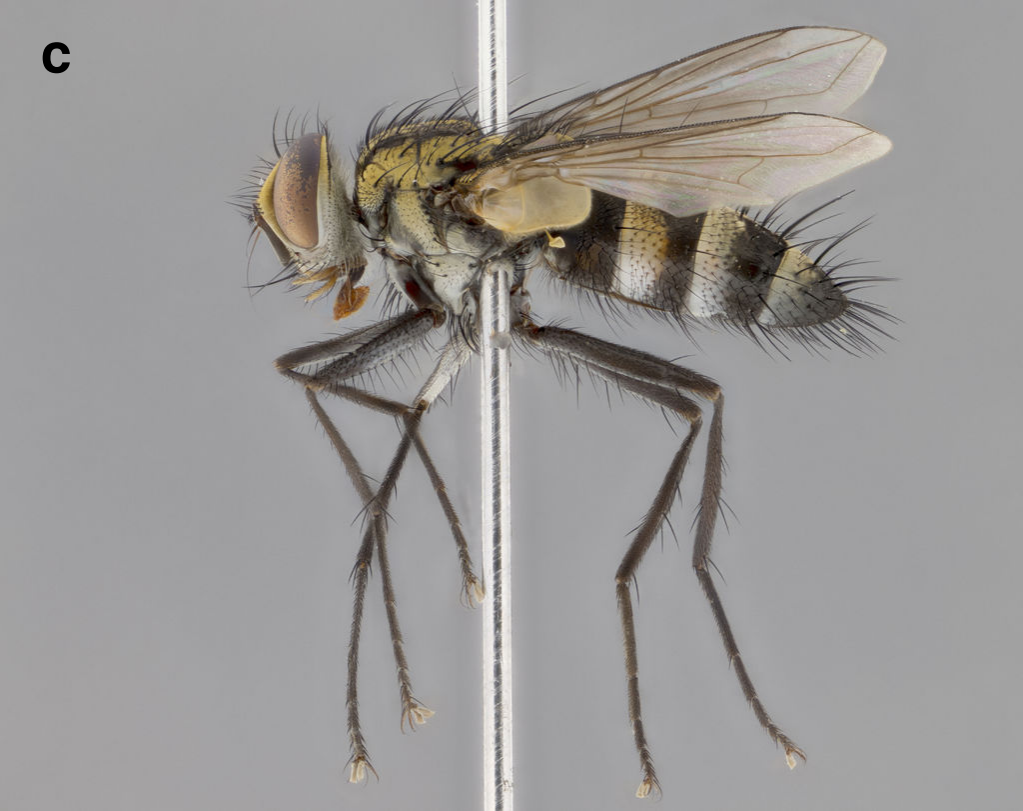
habitus in lateral view

**Figure 14a.**
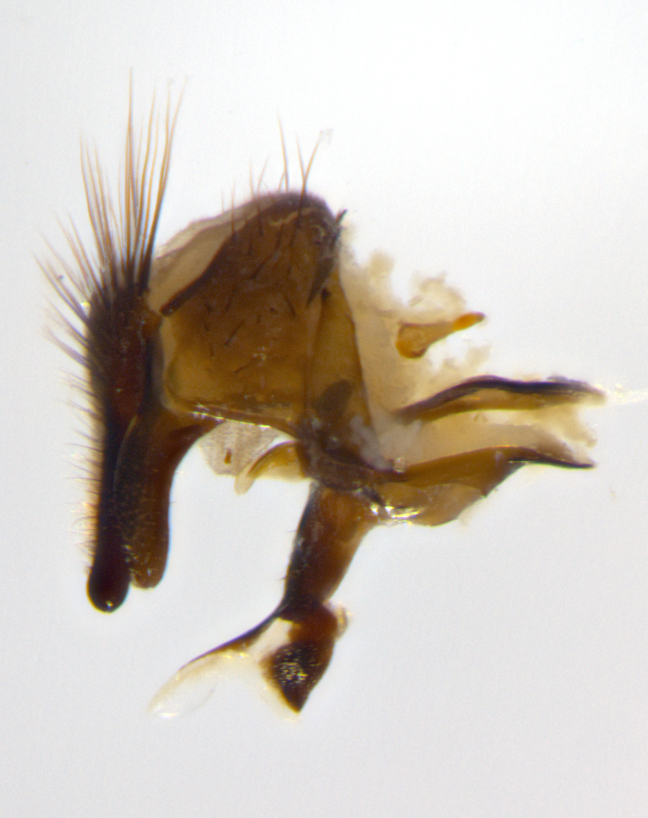
lateral view

**Figure 14b.**
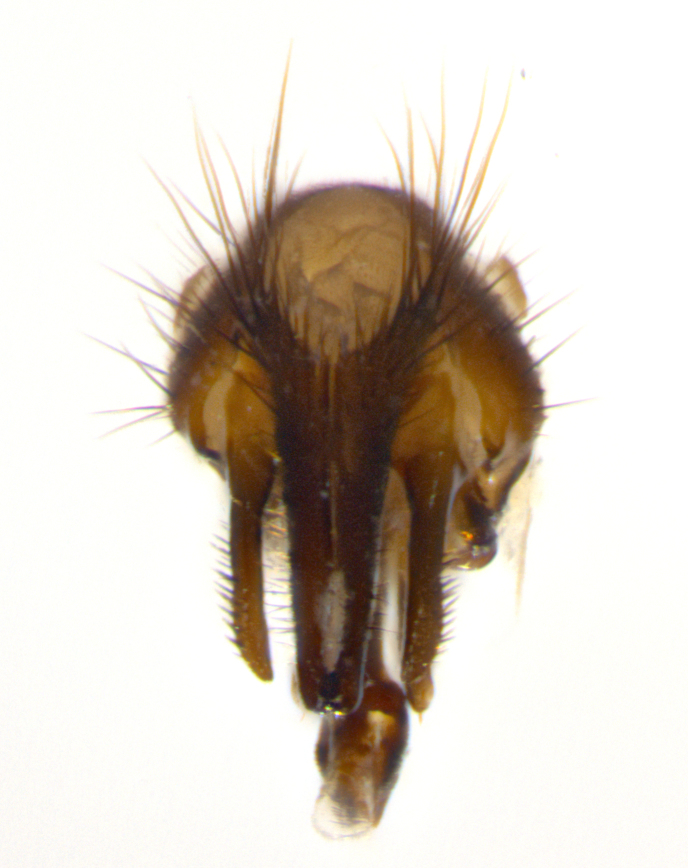
dorsal view

**Figure 14c.**
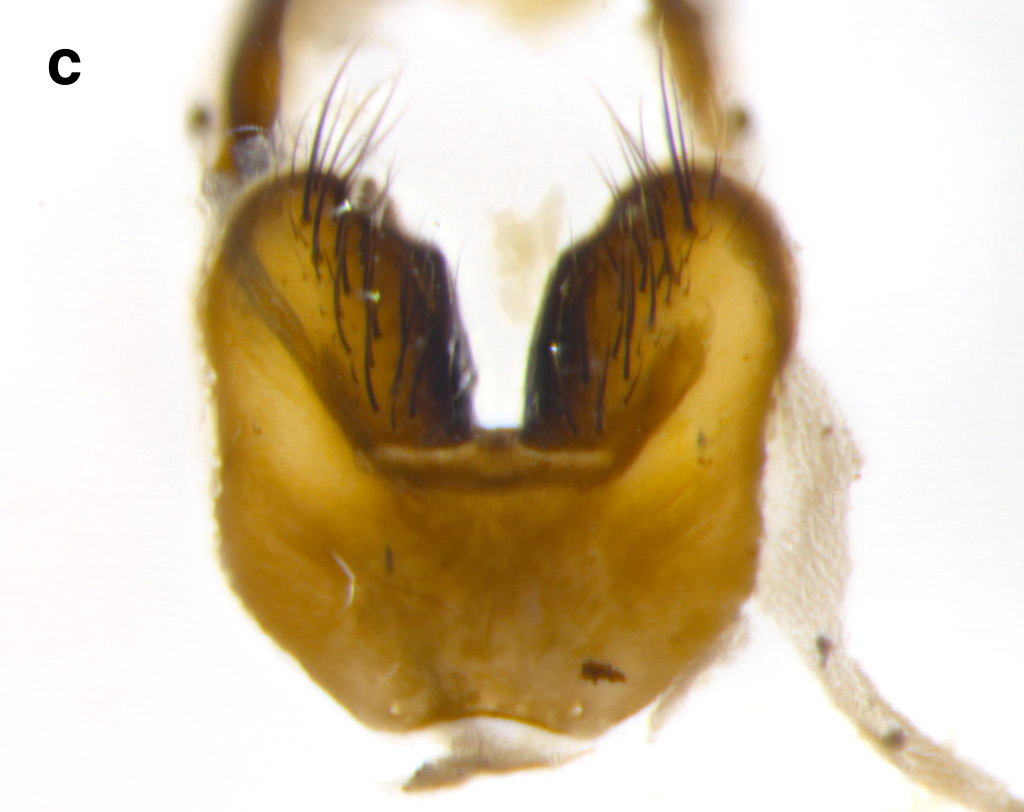
sternite 5 in ventral view

**Figure 15a.**
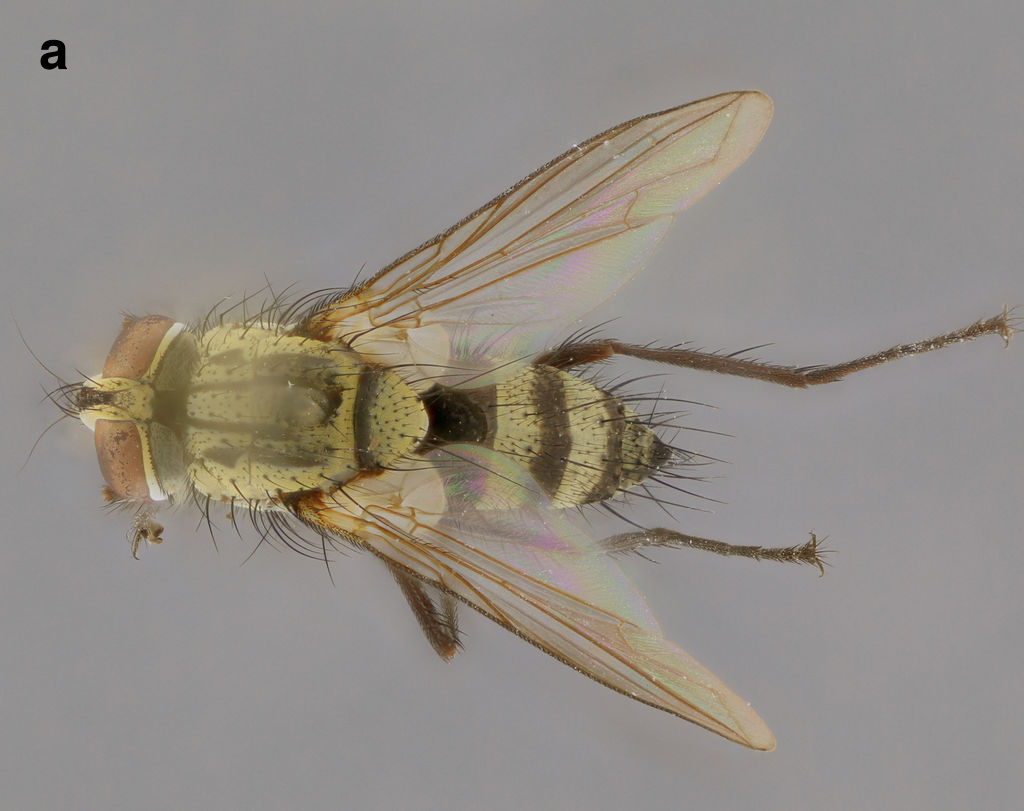
habitus in dorsal view

**Figure 15b.**
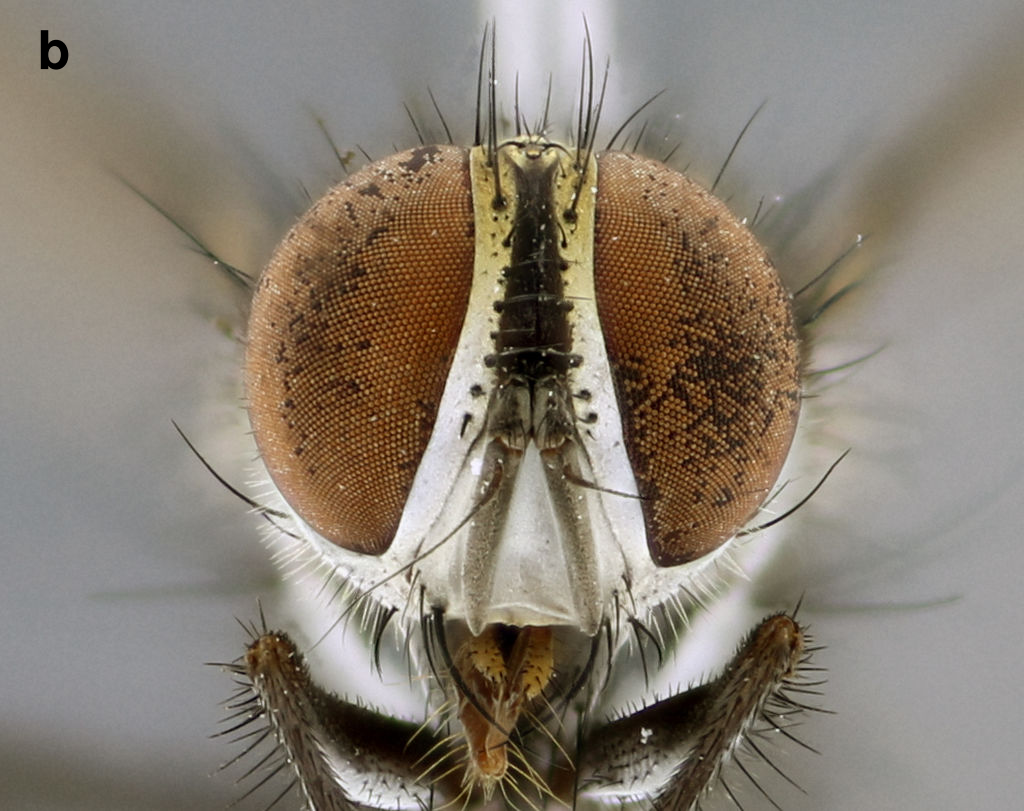
head in frontal view

**Figure 15c.**
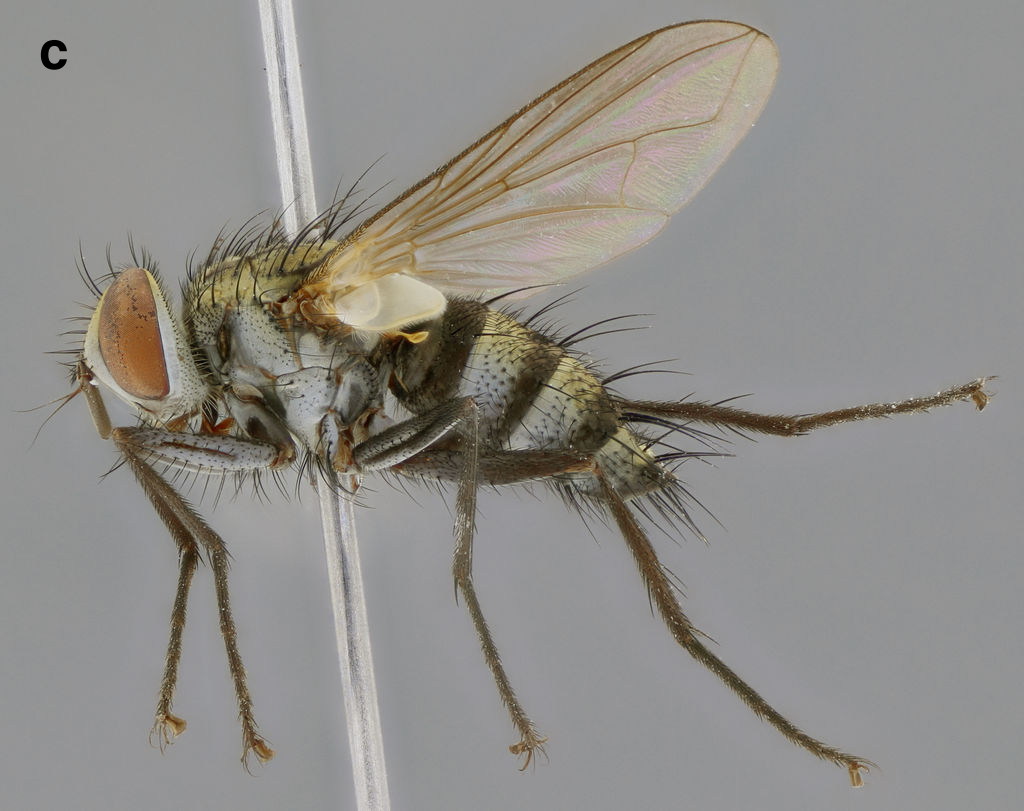
habitus in lateral view

**Figure 15d.**
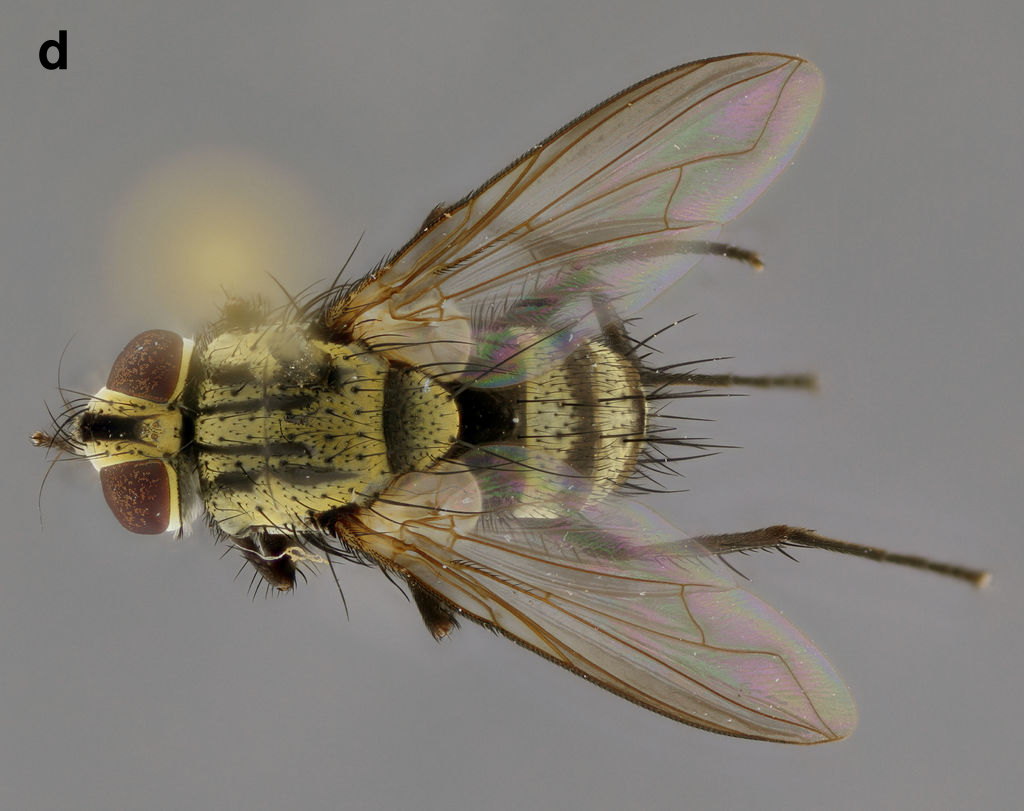
habitus in dorsal view

**Figure 15e.**
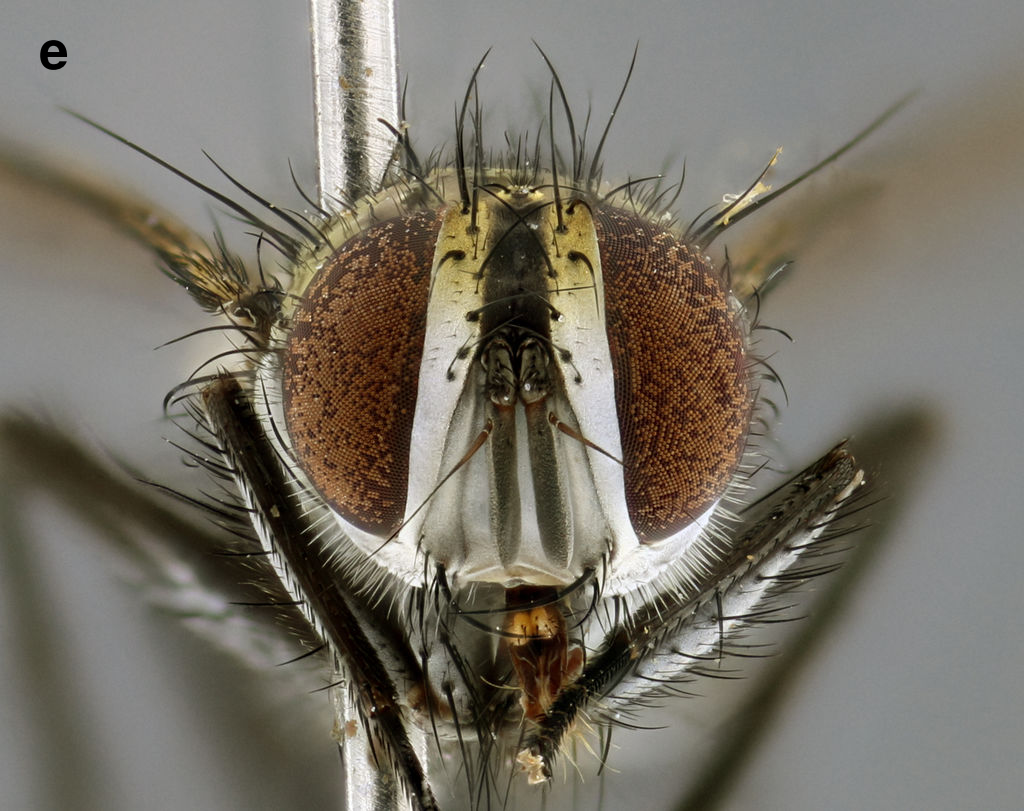
head in frontal view

**Figure 15f.**
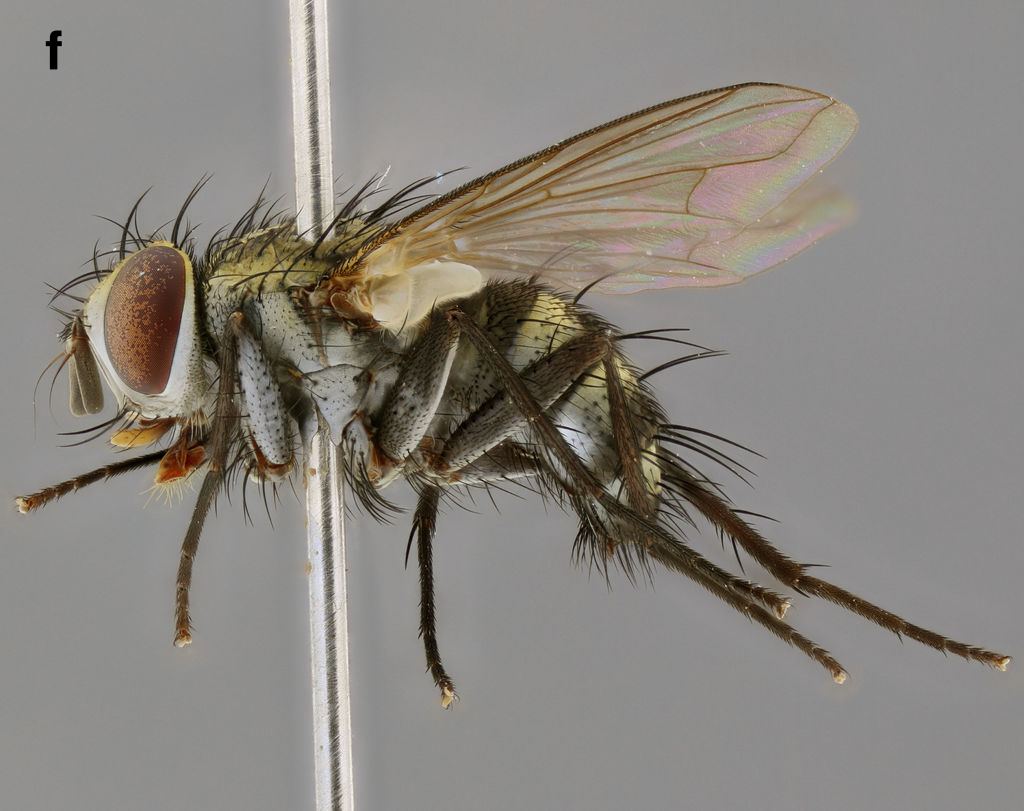
habitus in lateral view

**Figure 16a.**
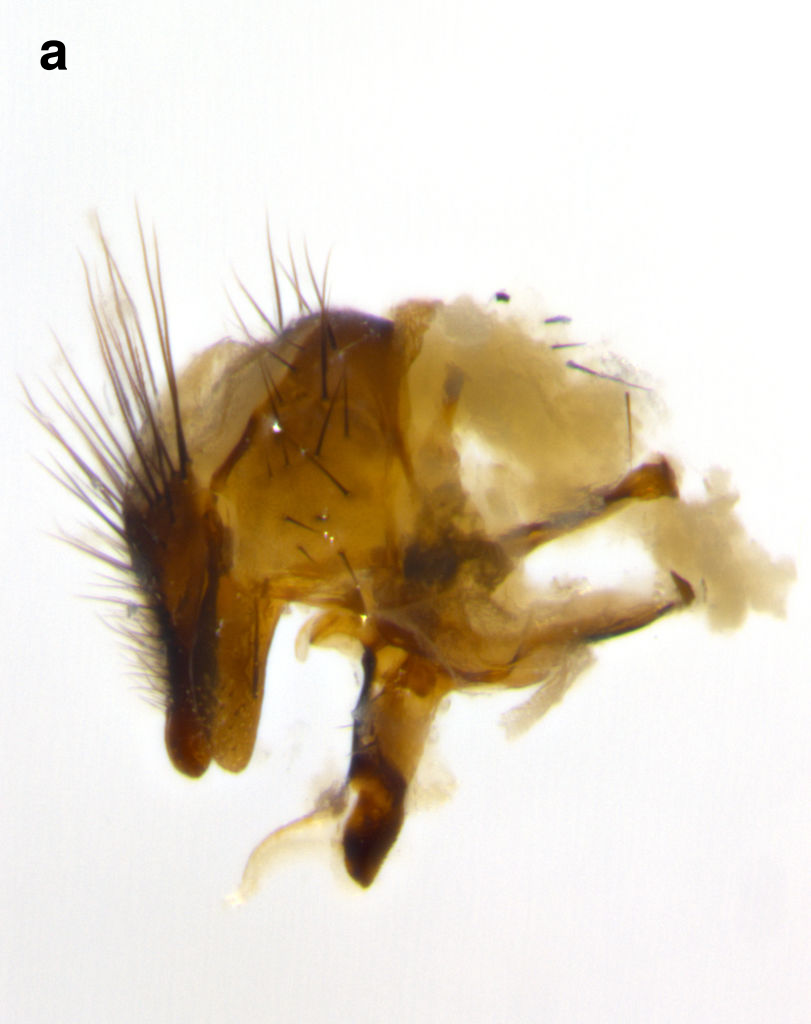
lateral view

**Figure 16b.**
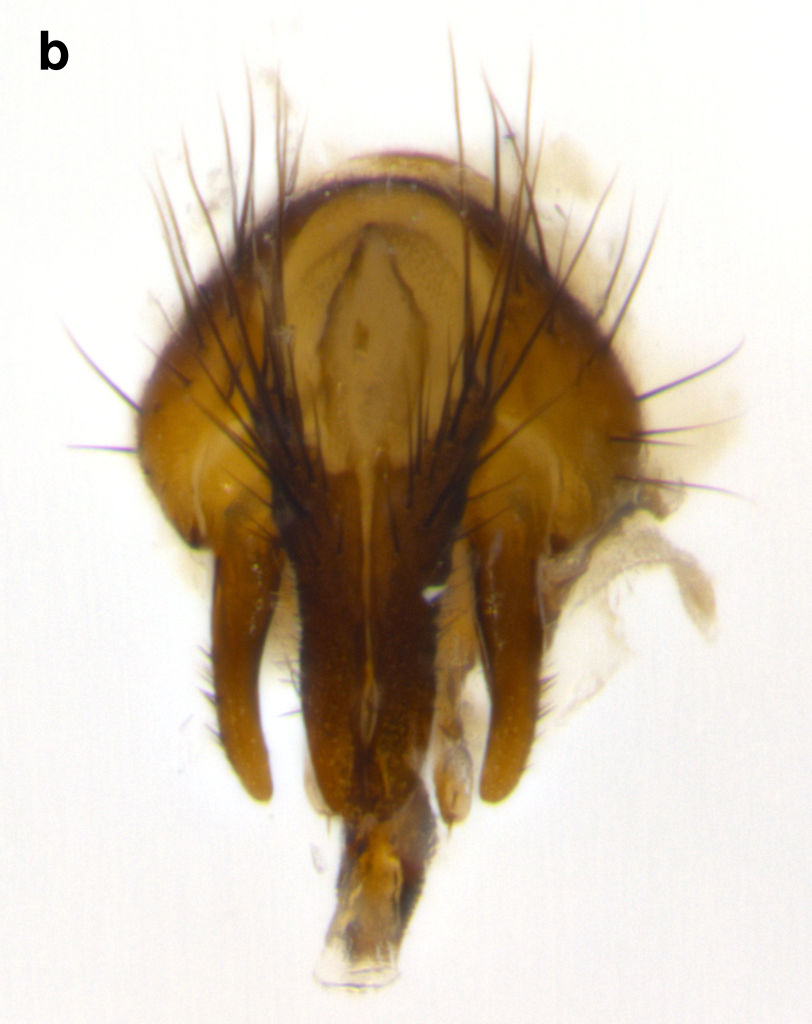
dorsal view

**Figure 16c.**
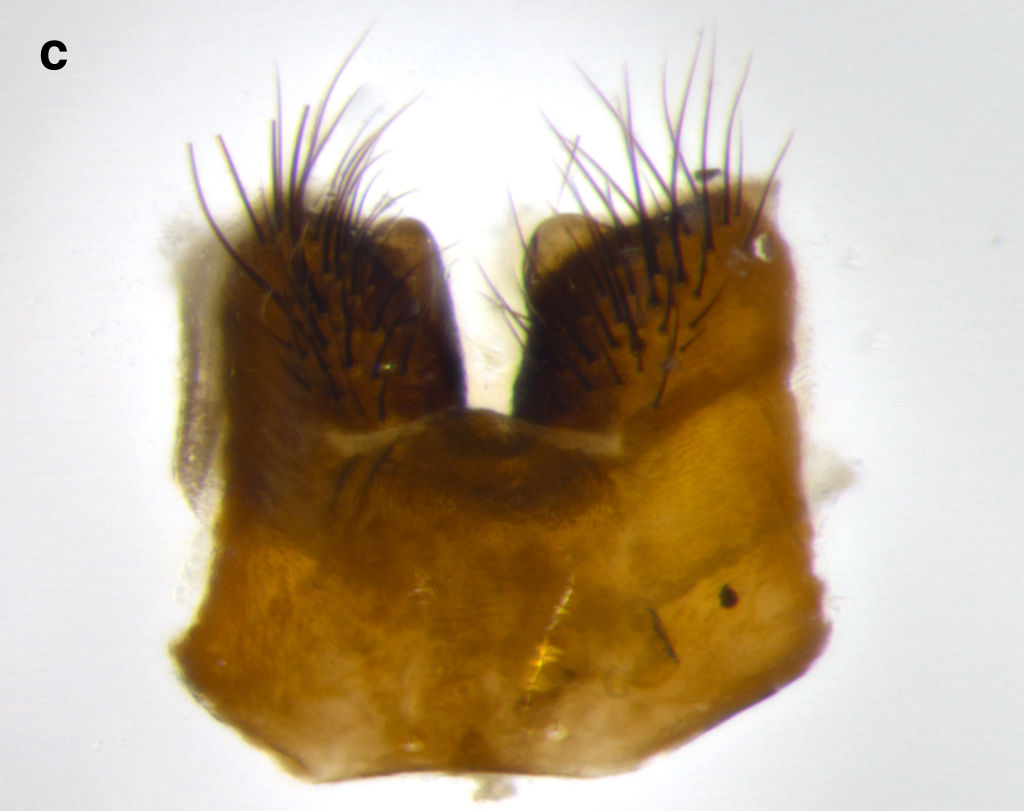
sternite 5 in ventral view

**Figure 16d.**
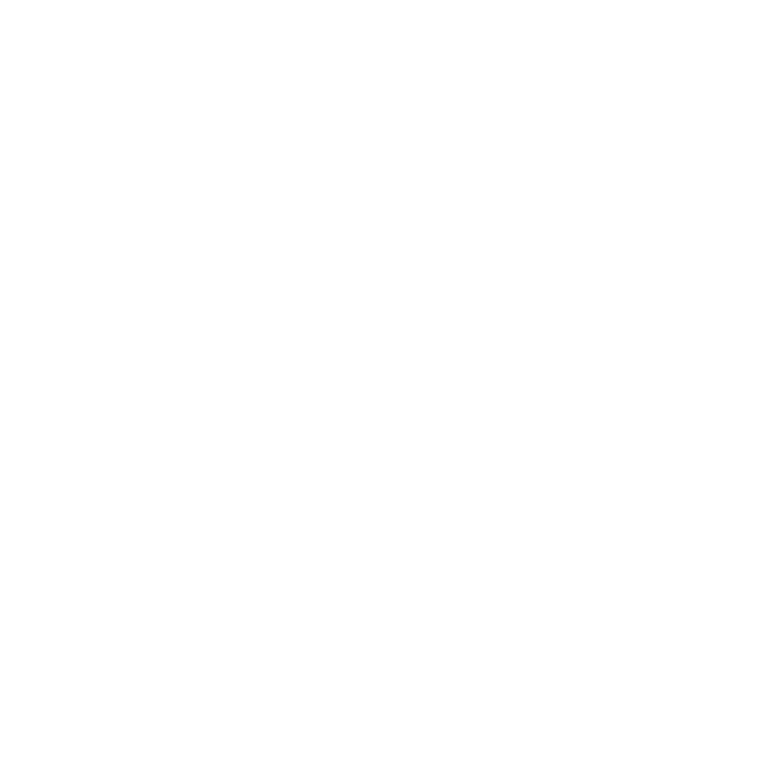


**Figure 17a.**
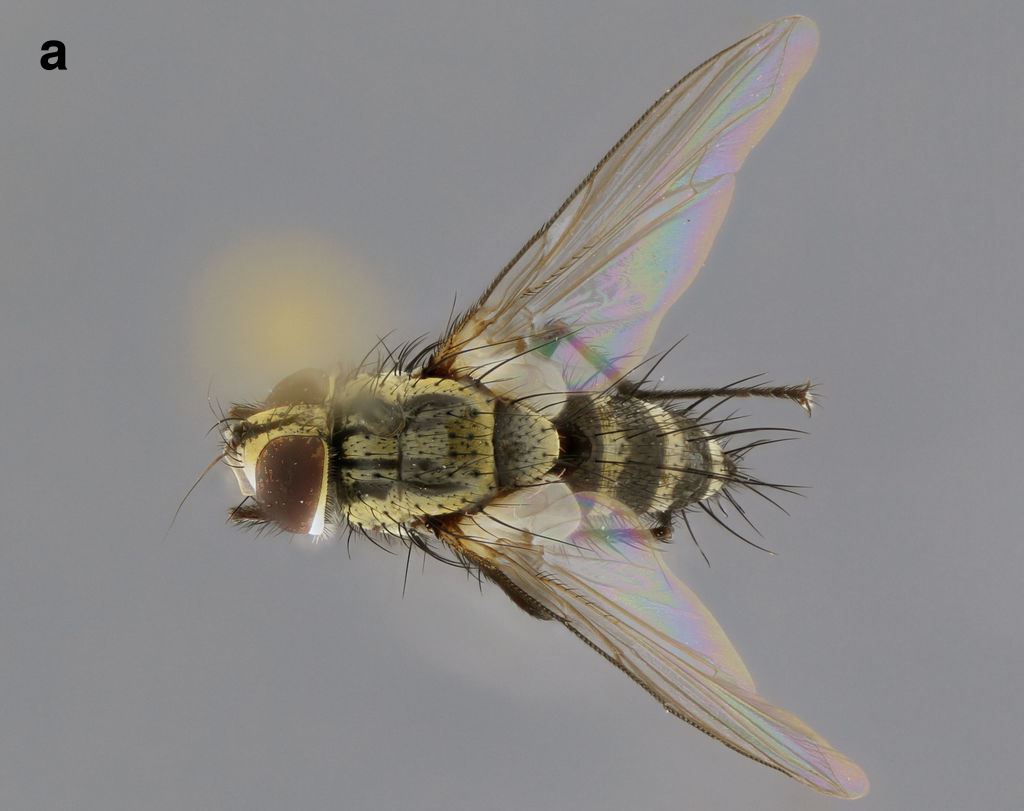
habitus in dorsal view

**Figure 17b.**
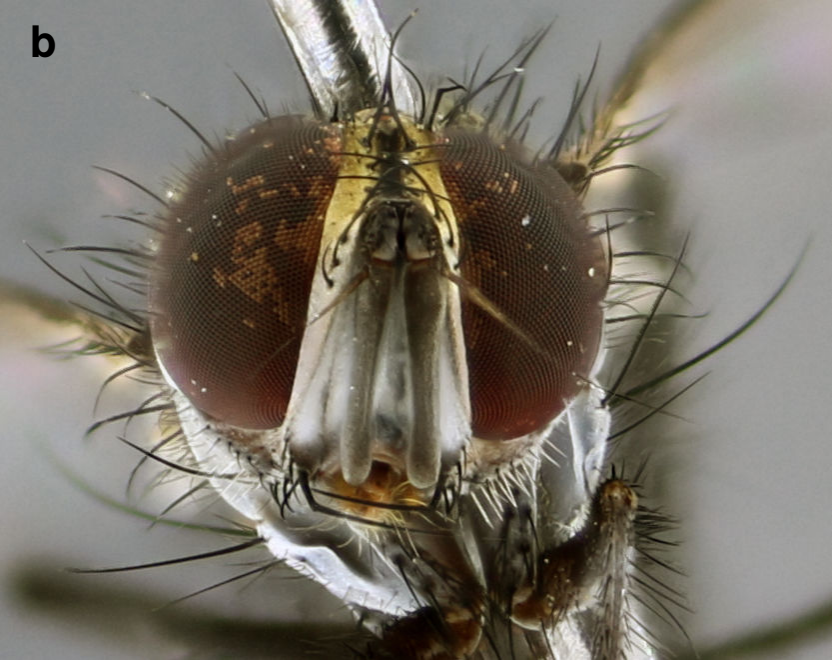
head in frontal view

**Figure 17c.**
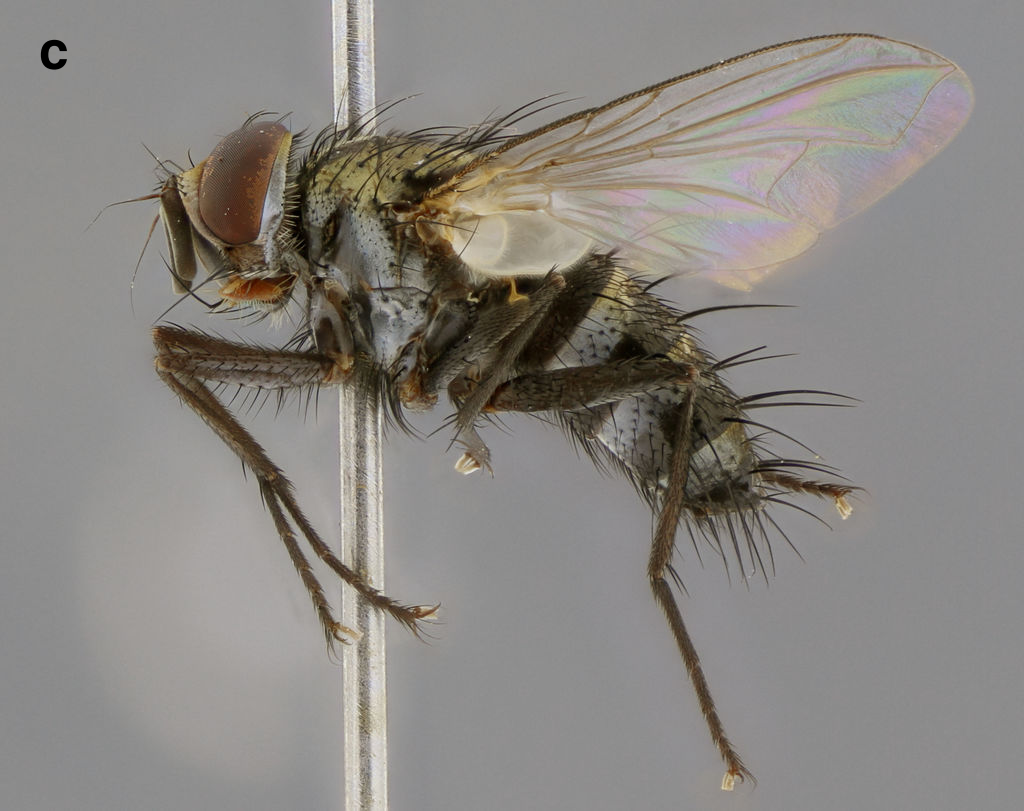
habitus in lateral view

**Figure 17d.**
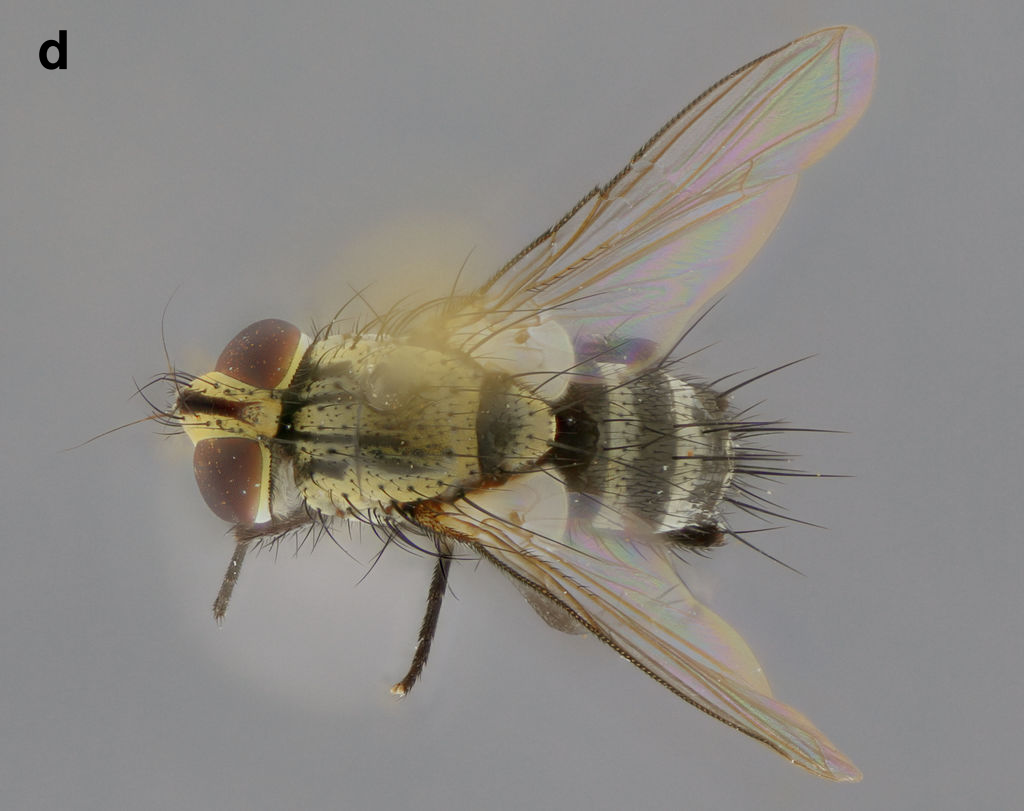
habitus in dorsal view

**Figure 17e.**
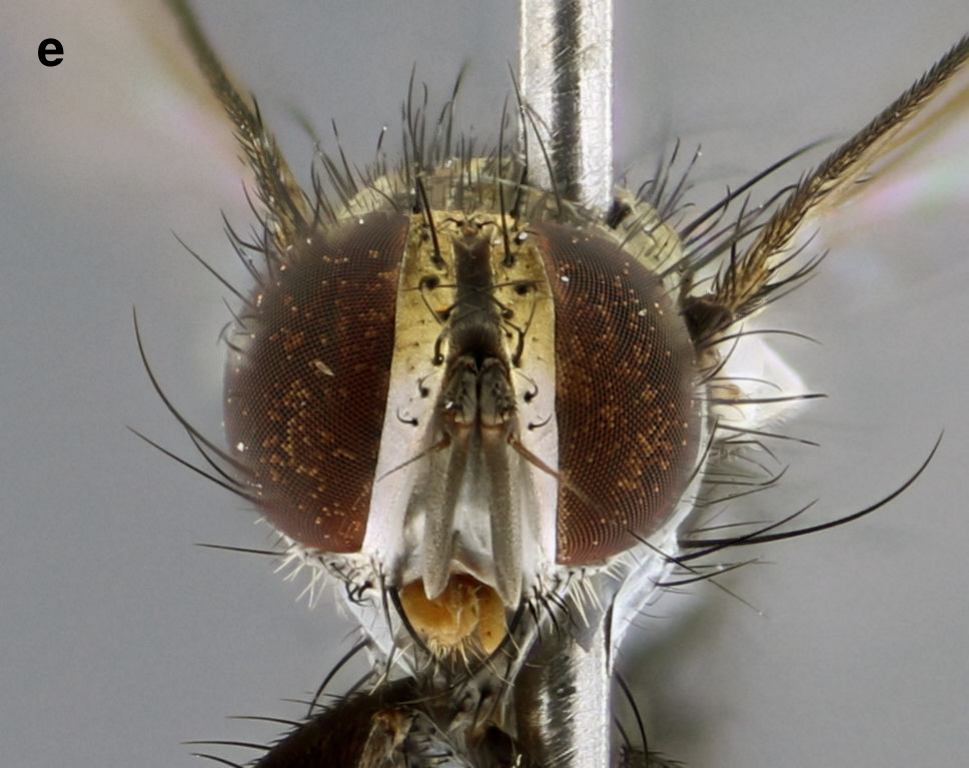
head in frontal view

**Figure 17f.**
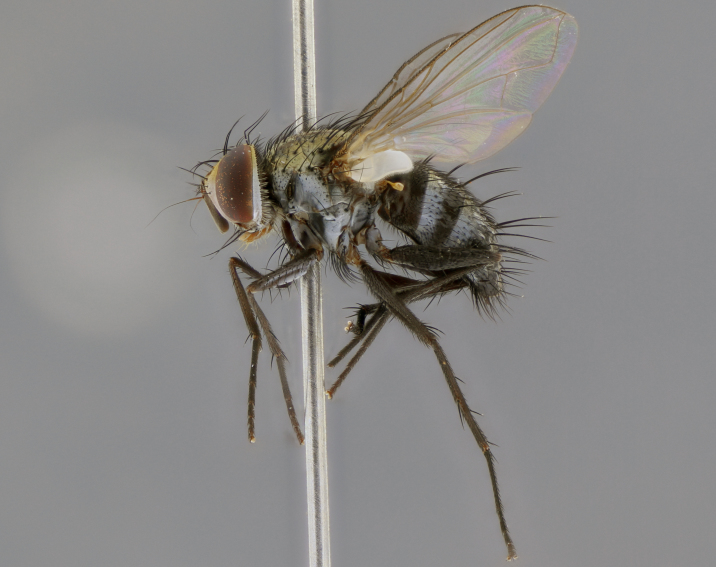
habitus in lateral view

**Figure 18a.**
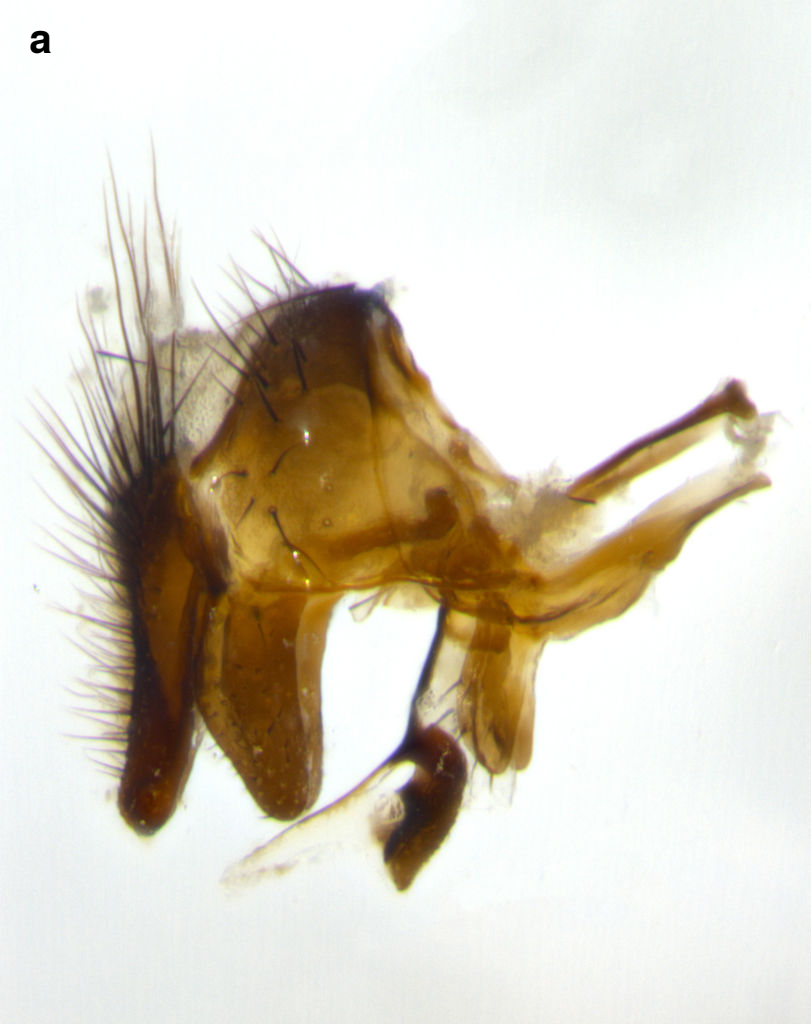
lateral view

**Figure 18b.**
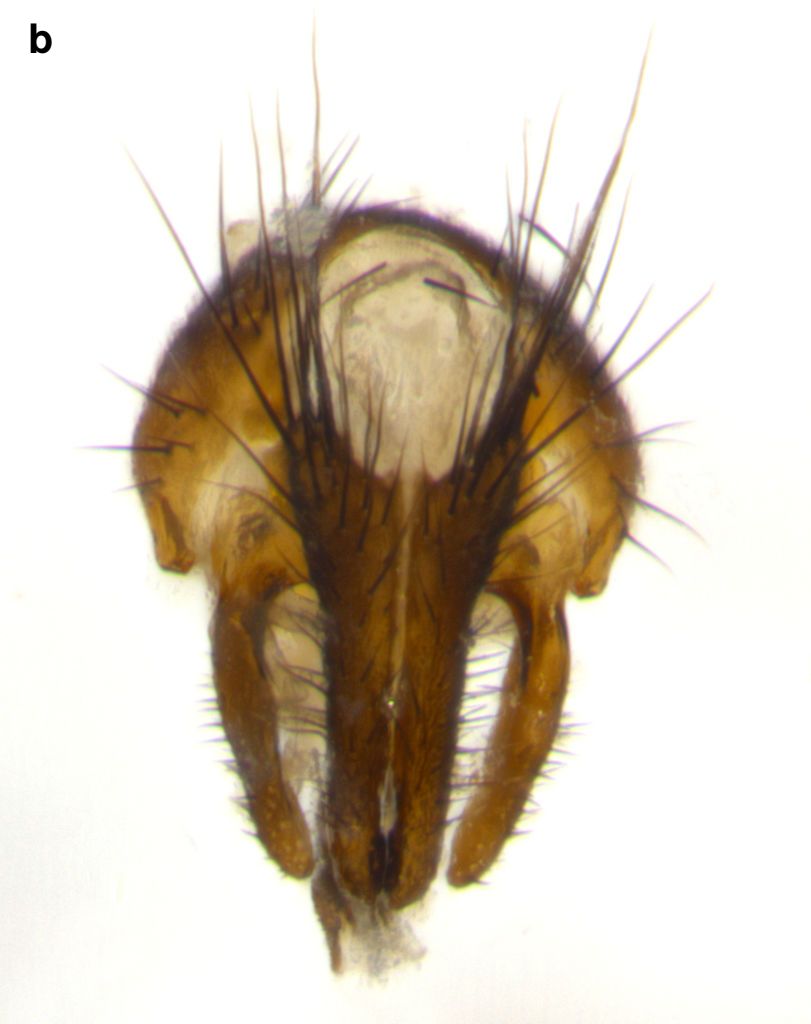
dorsal view

**Figure 18c.**
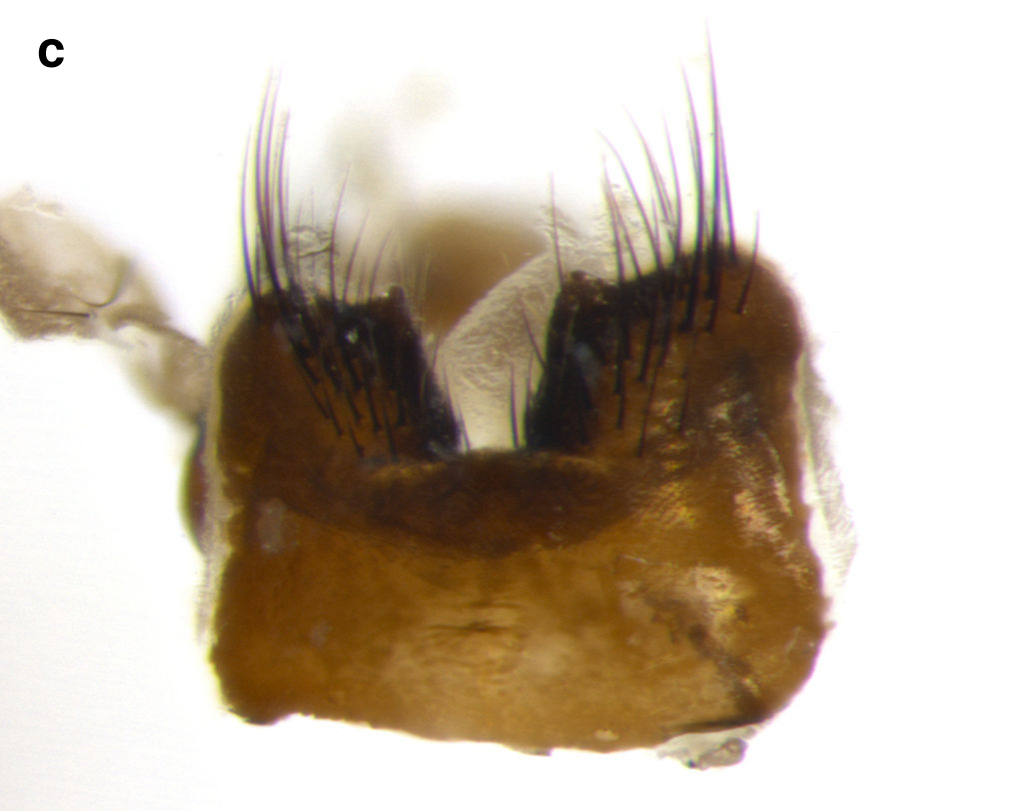
sternite 5 in ventral view

**Figure 19a.**
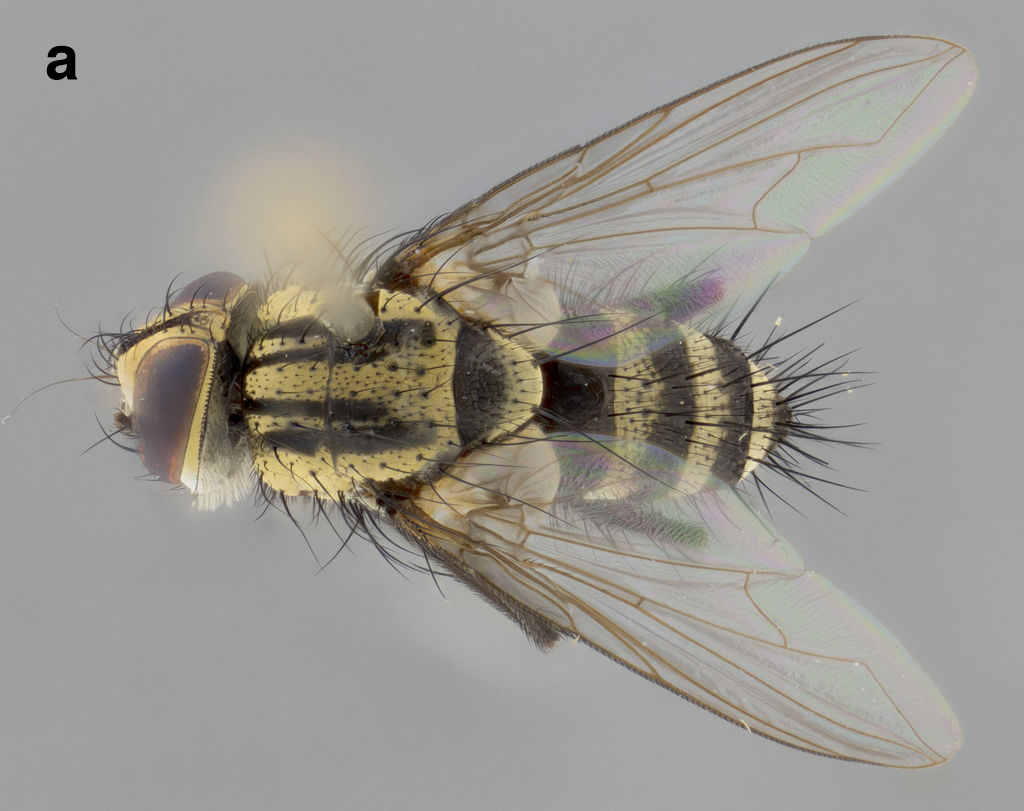
habitus in dorsal view

**Figure 19b.**
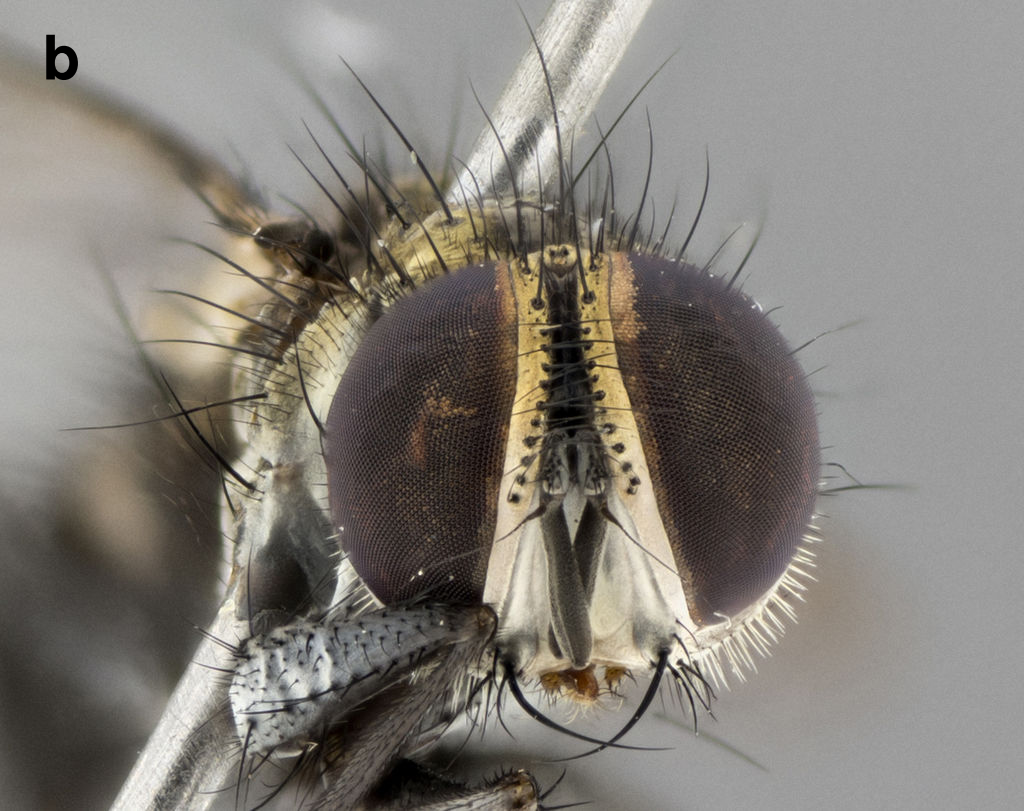
head in frontal view

**Figure 19c.**
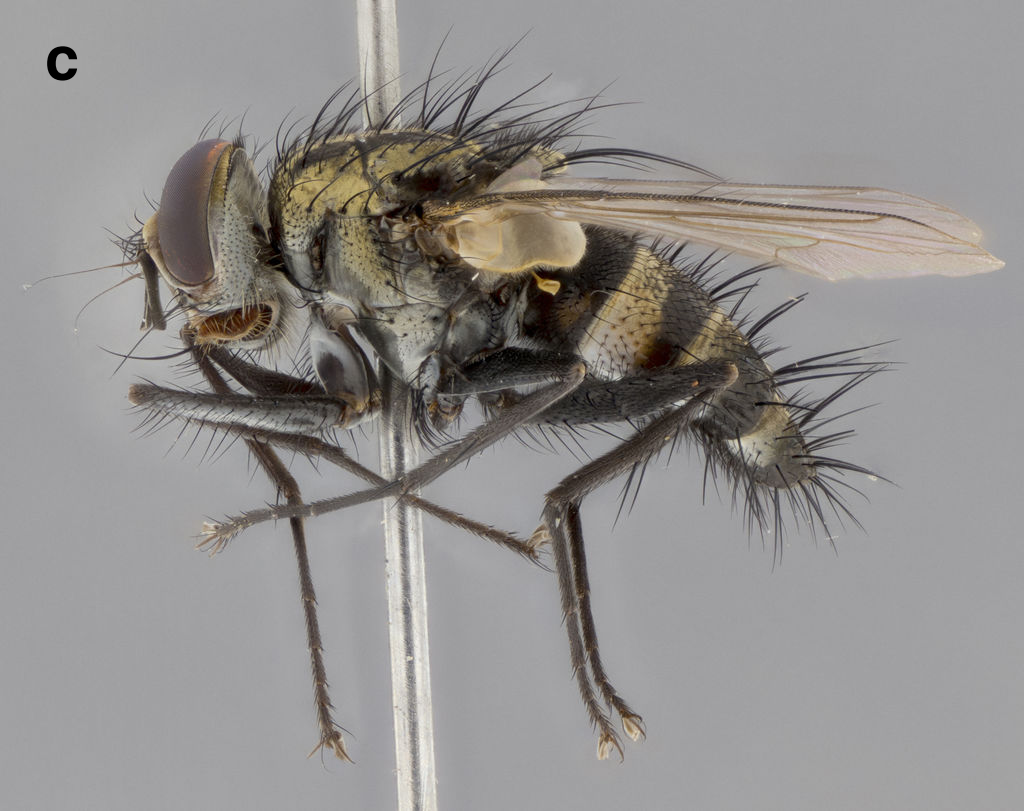
habitus in lateral view

**Figure 19d.**
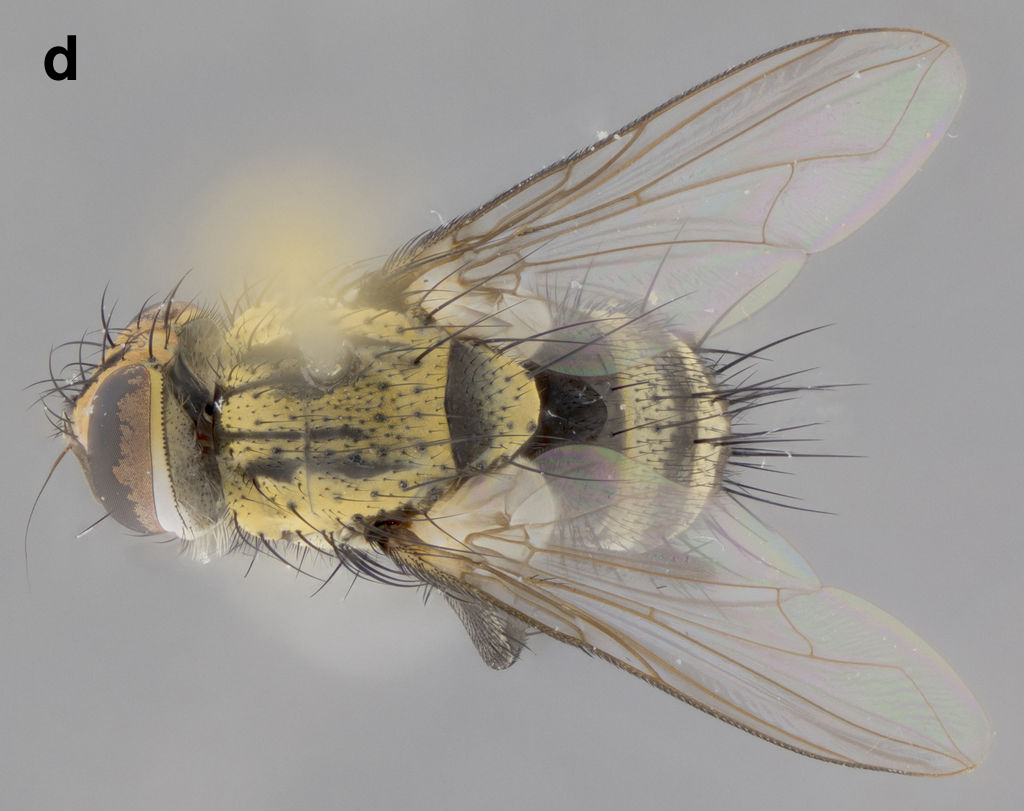
habitus in dorsal view

**Figure 19e.**
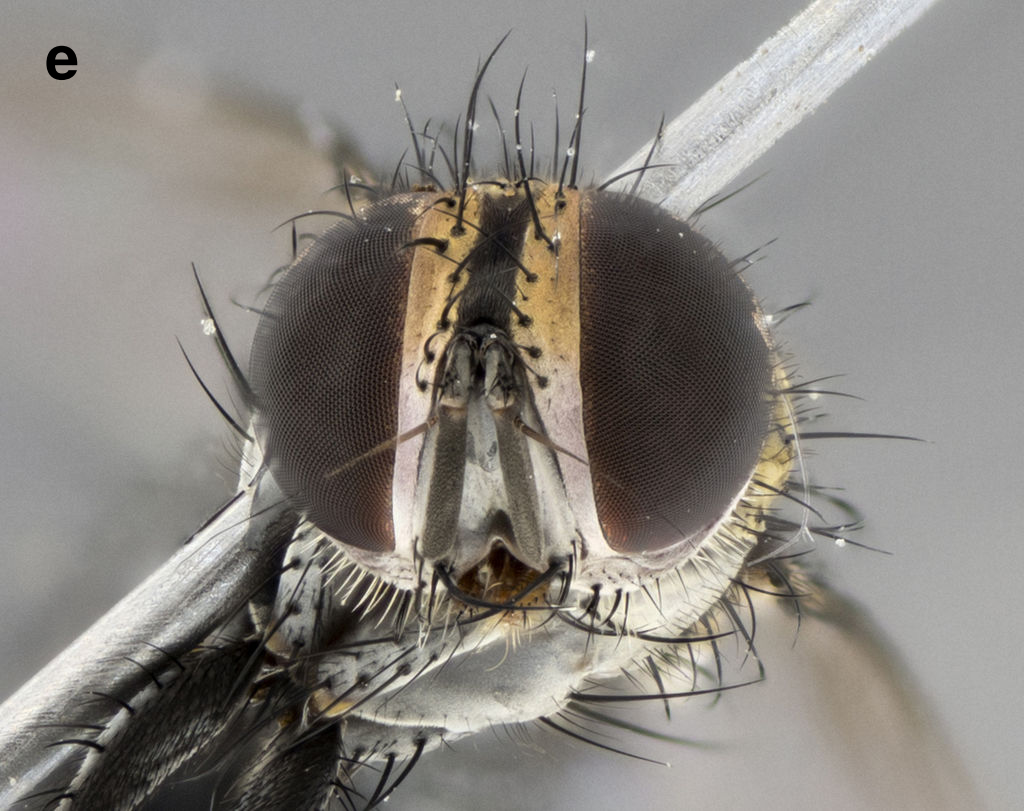
head in frontal view

**Figure 19f.**
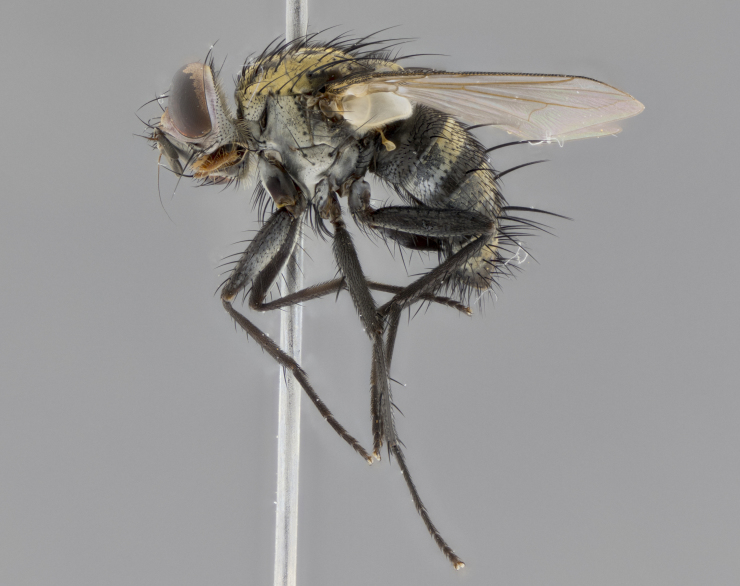
habitus in lateral view

**Figure 20a.**
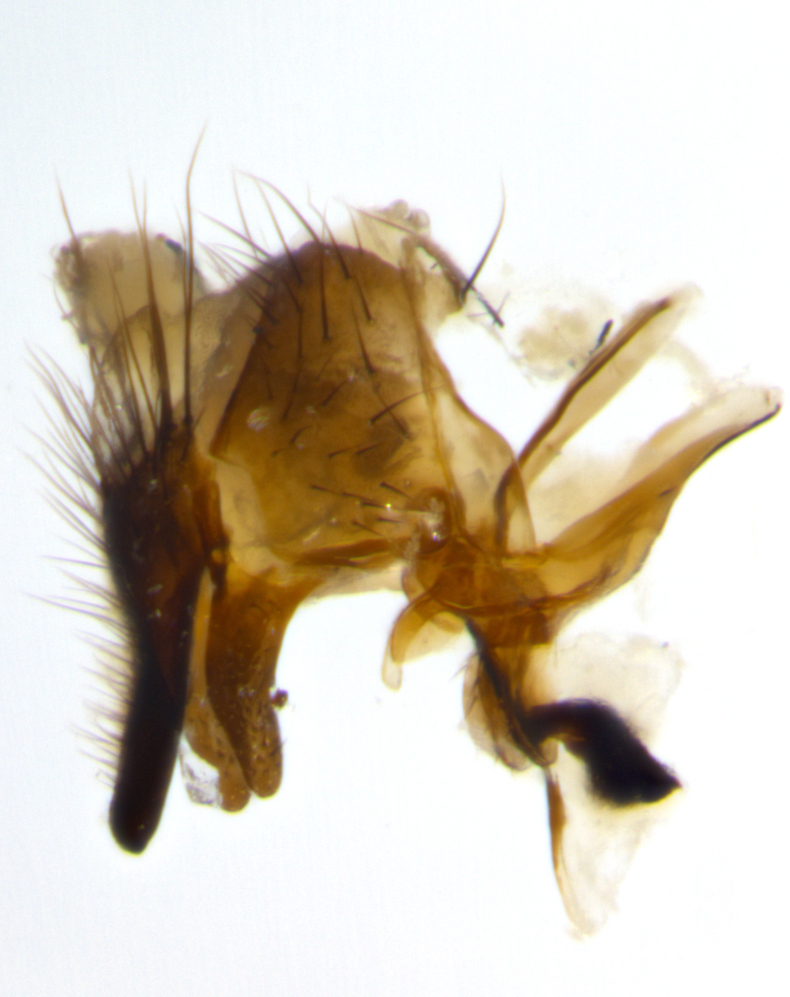
lateral view

**Figure 20b.**
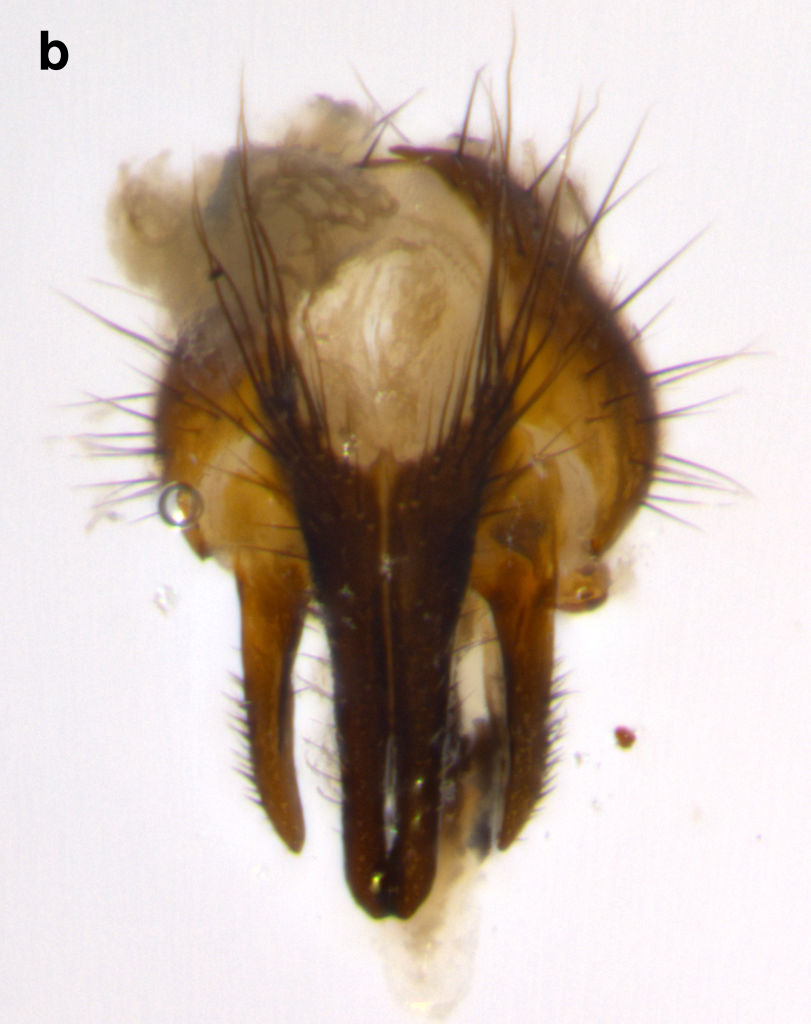
dorsal view

**Figure 20c.**
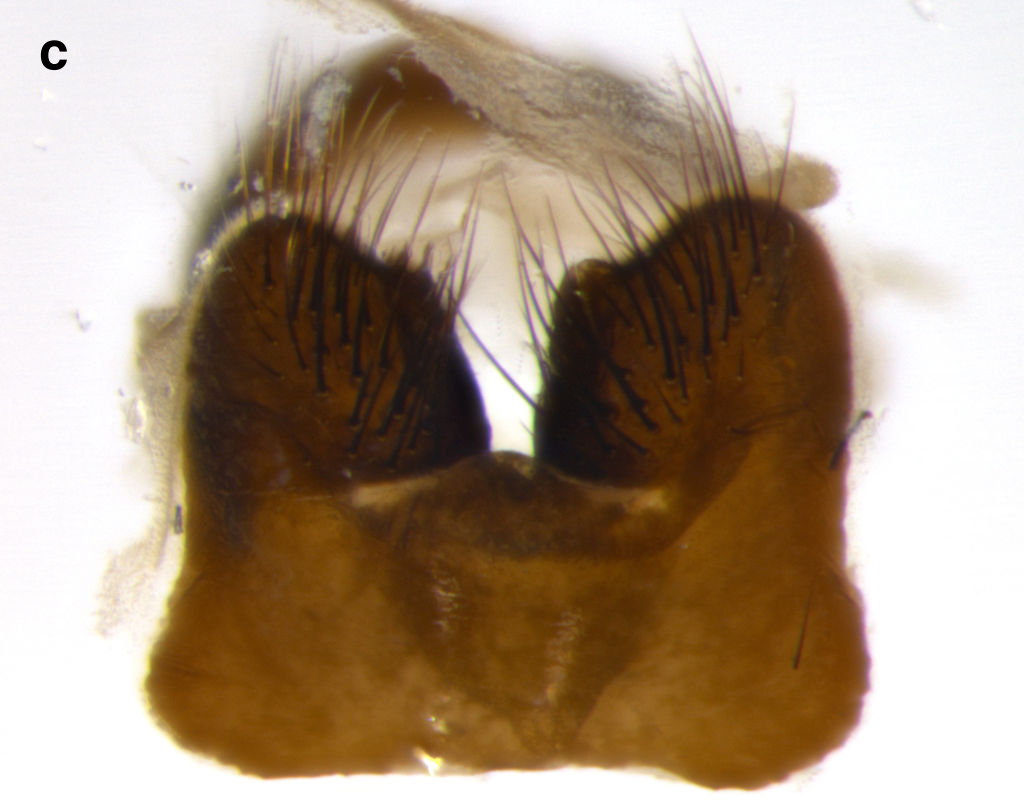
sternite 5 in ventral view

**Figure 21a.**
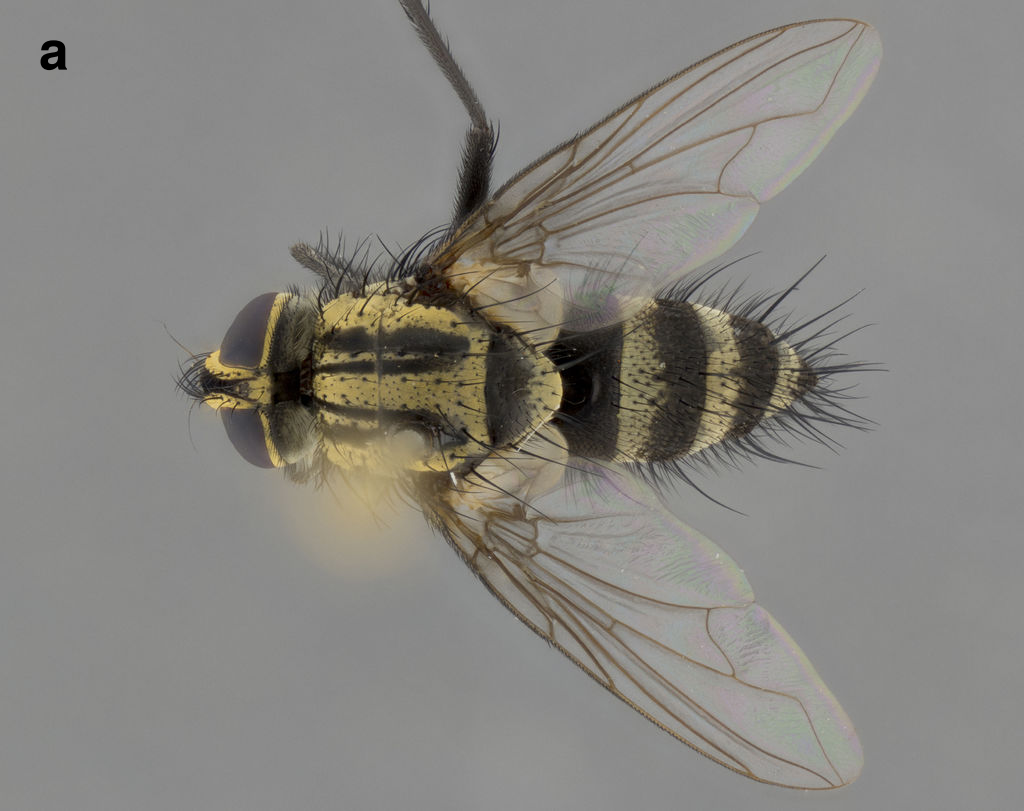
habitus in dorsal view

**Figure 21b.**
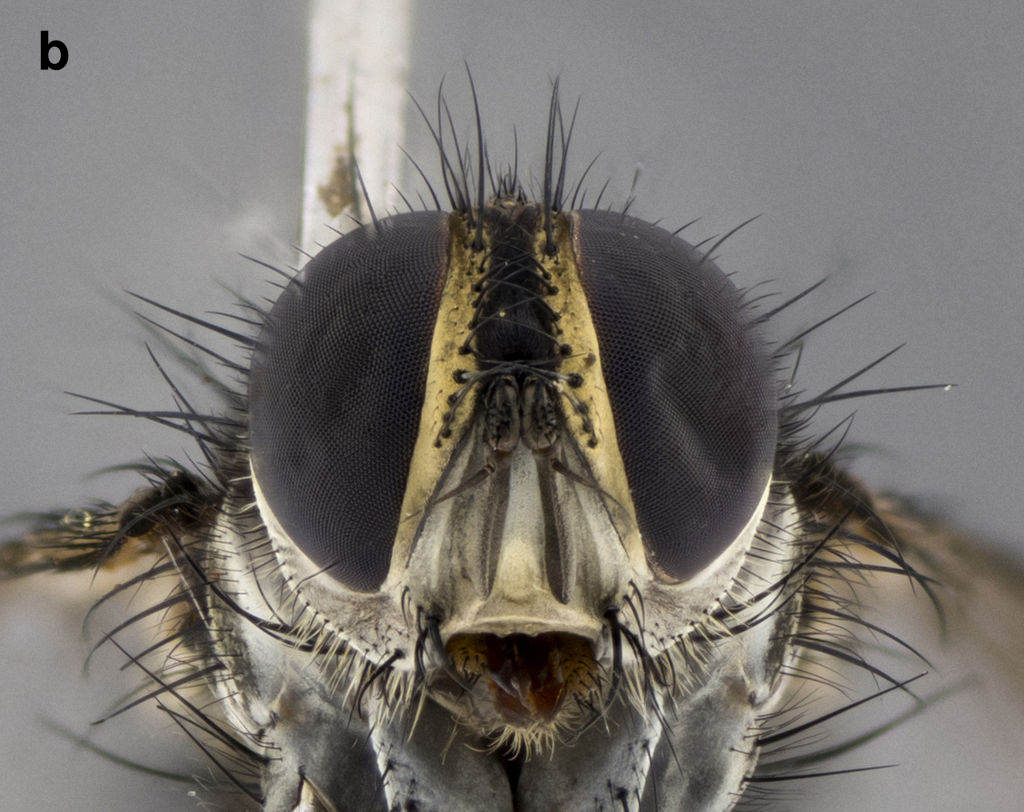
head in frontal view

**Figure 21c.**
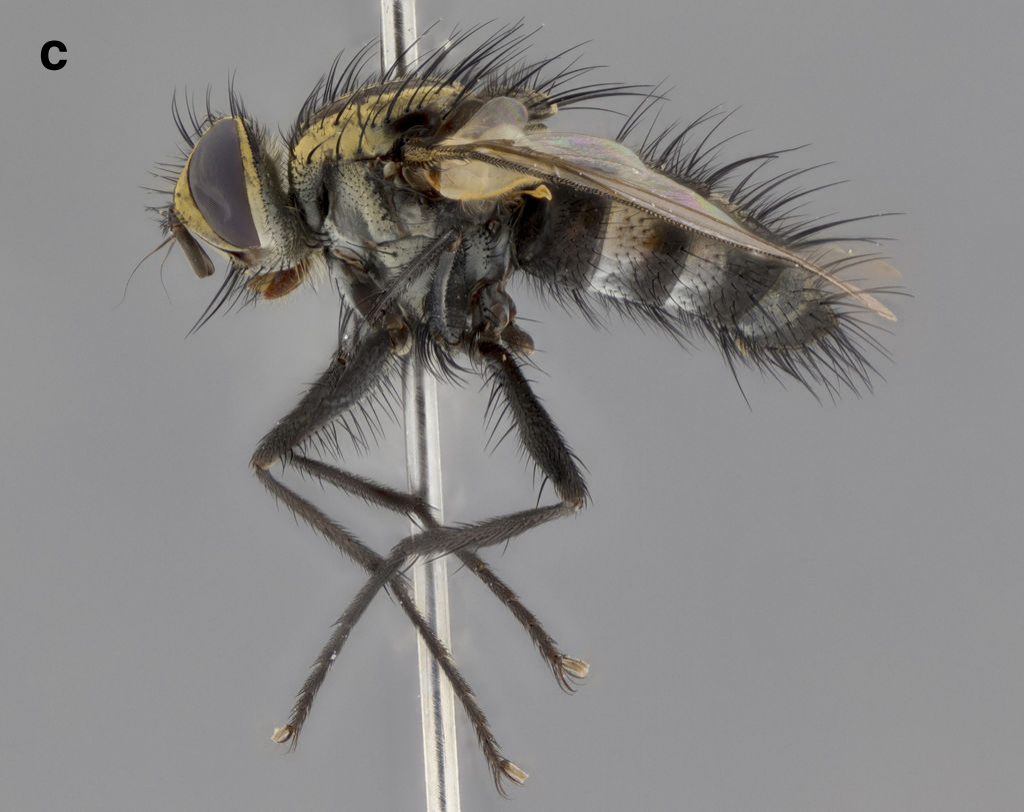
habitus in lateral view

**Figure 21d.**
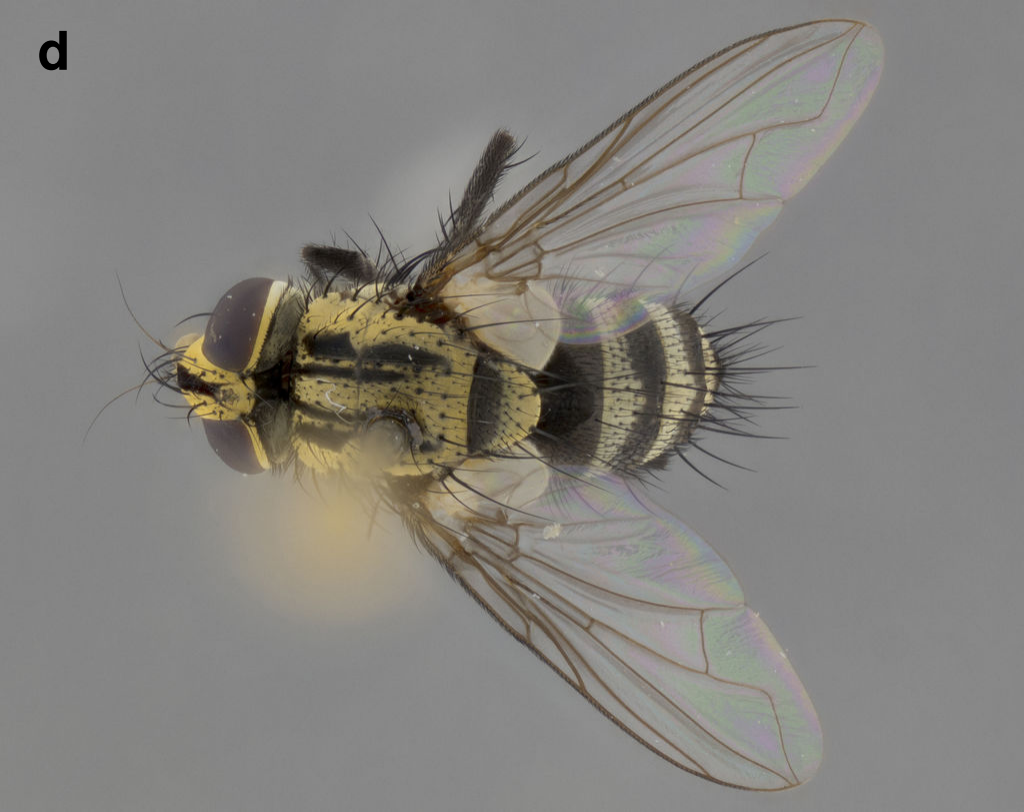
habitus in dorsal view

**Figure 21e.**
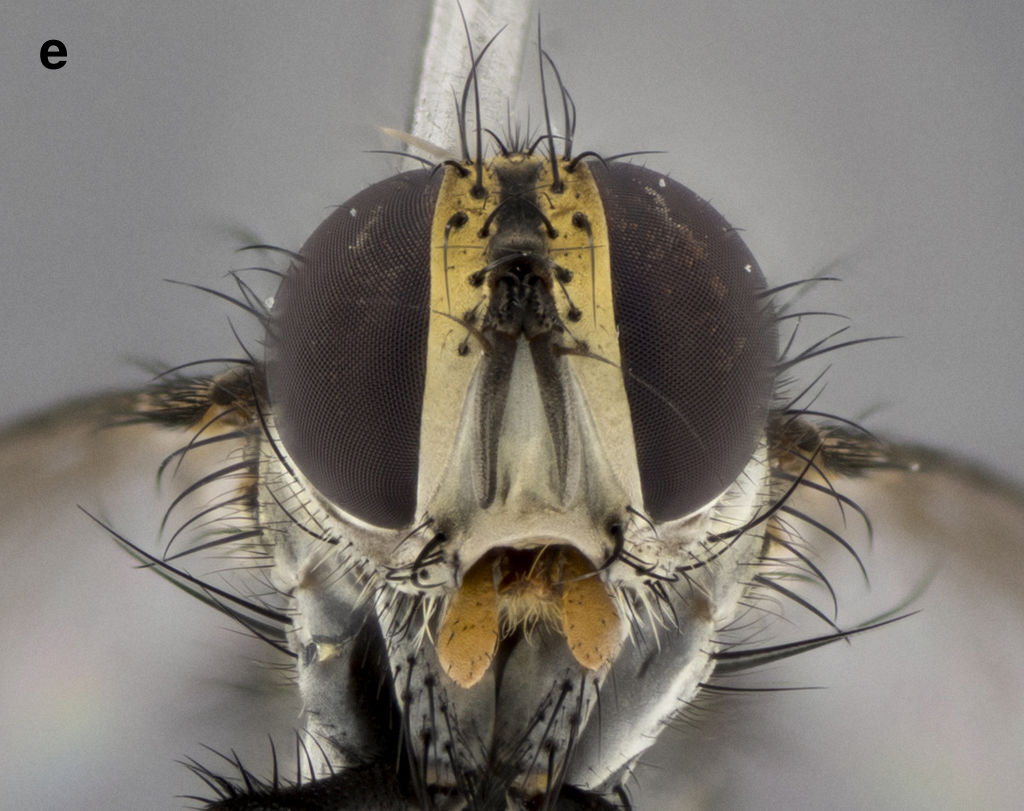
head in frontal view

**Figure 21f.**
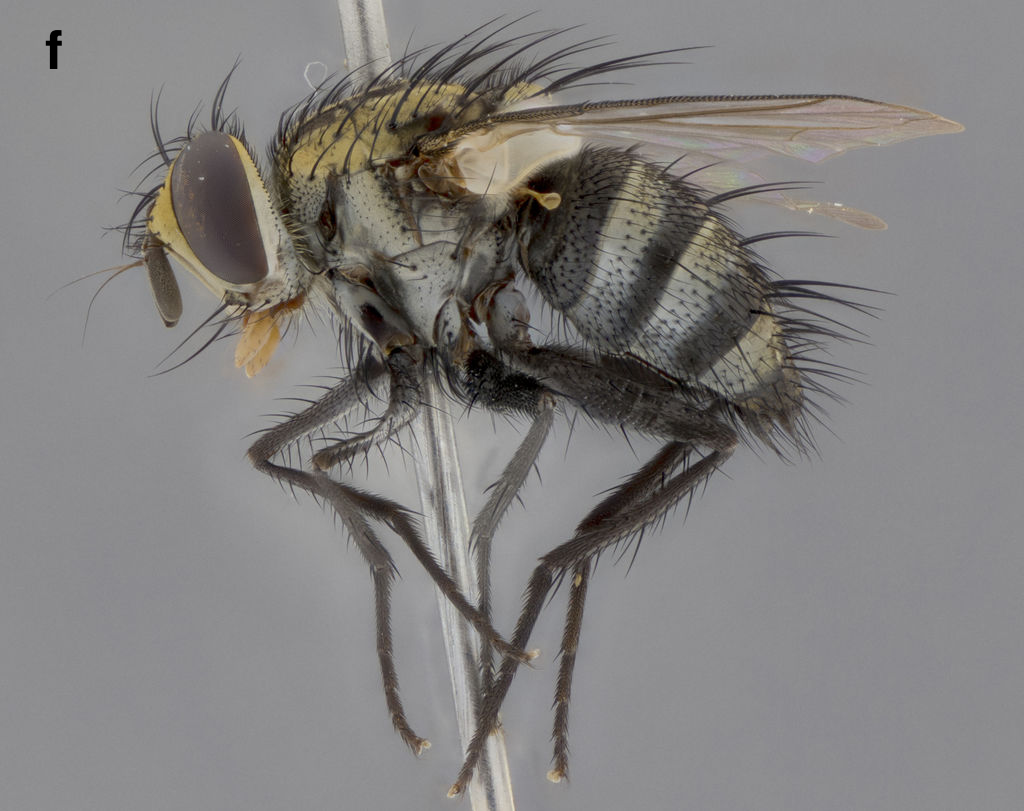
habitus in lateral view

**Figure 22a.**
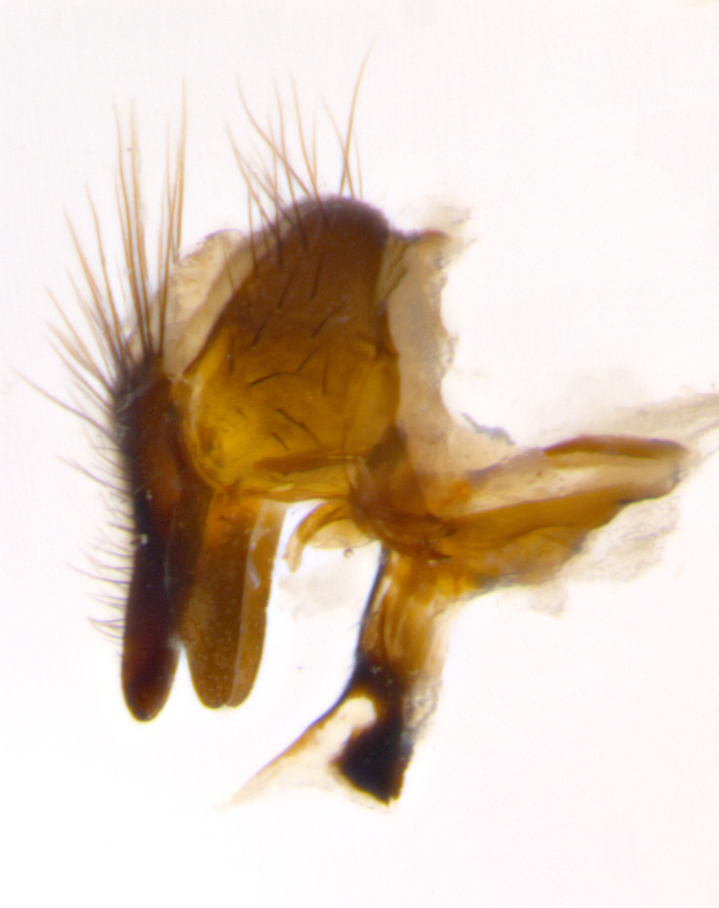
lateral view

**Figure 22b.**
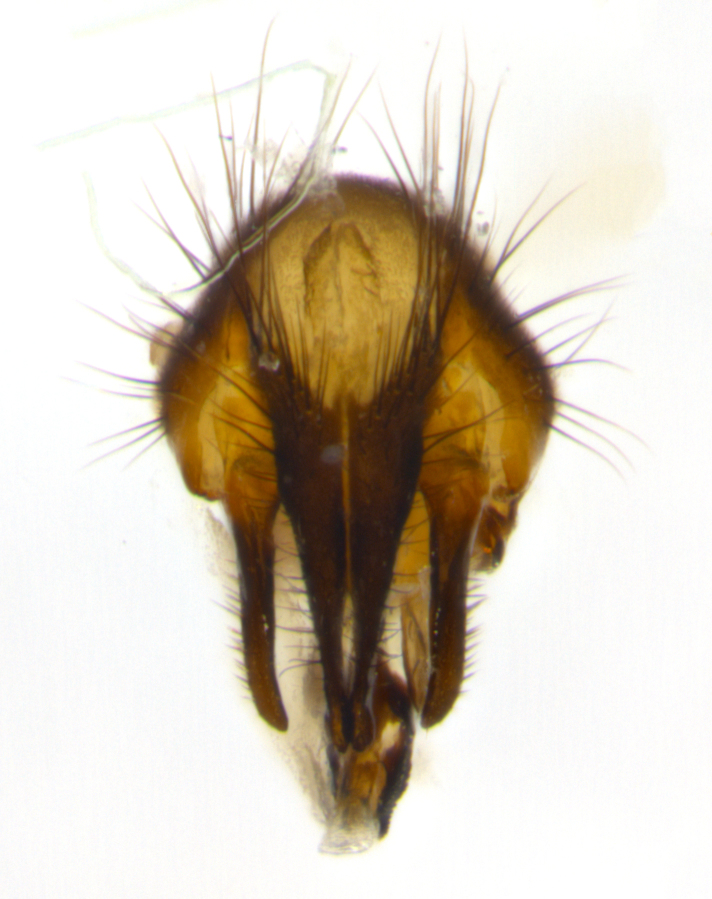
dorsal view

**Figure 22c.**
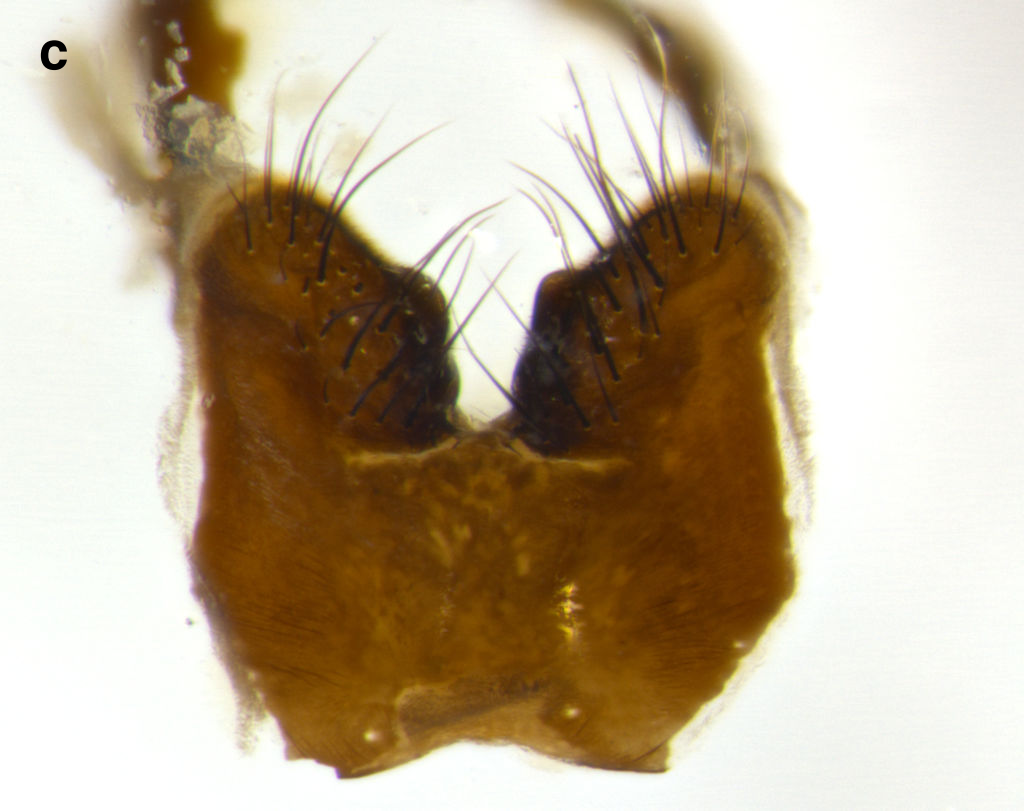
sternite 5 in ventral view

**Figure 23a.**
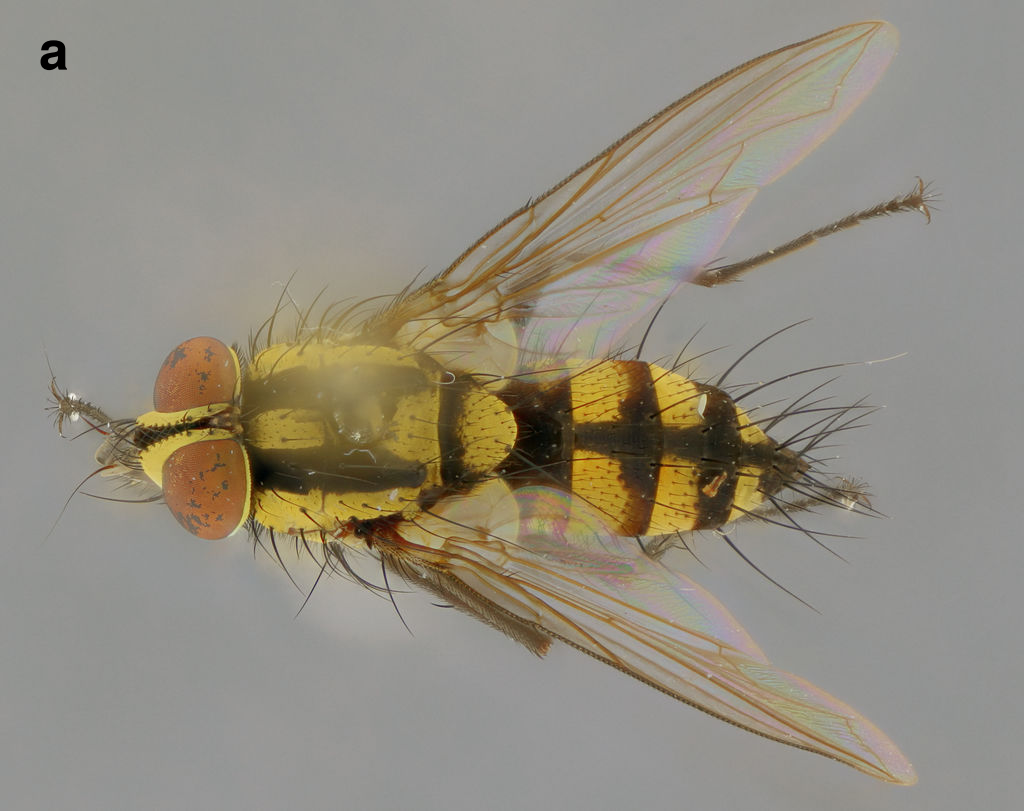
habitus in dorsal view

**Figure 23b.**
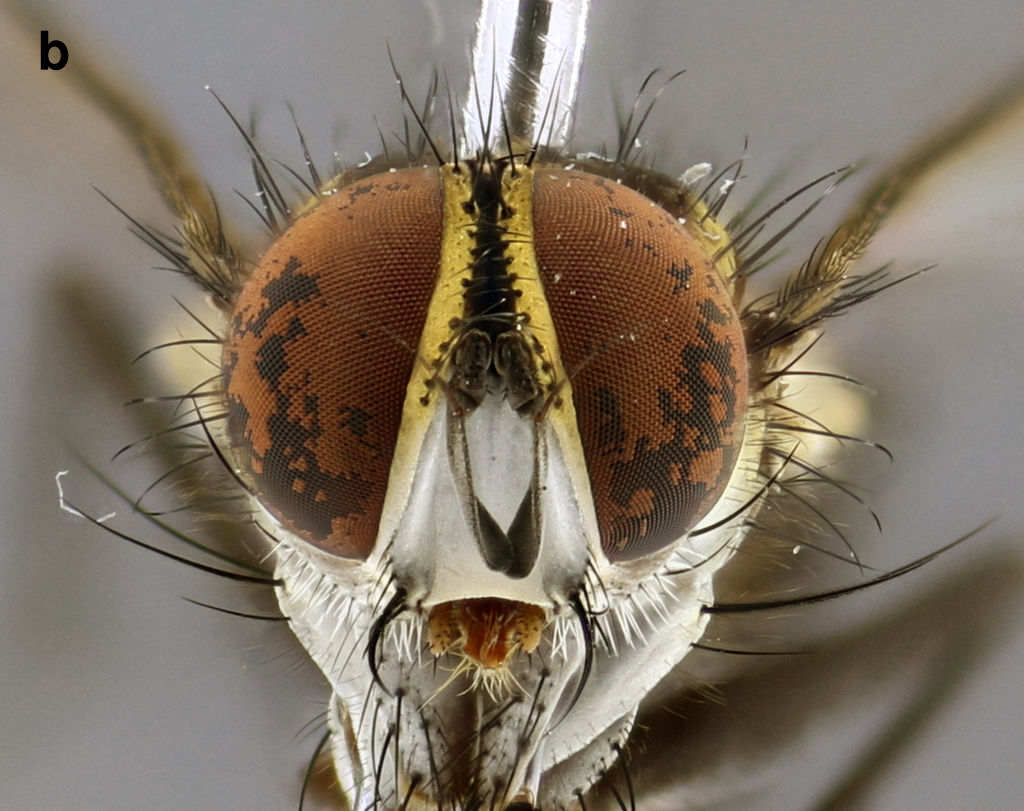
head in frontal view

**Figure 23c.**
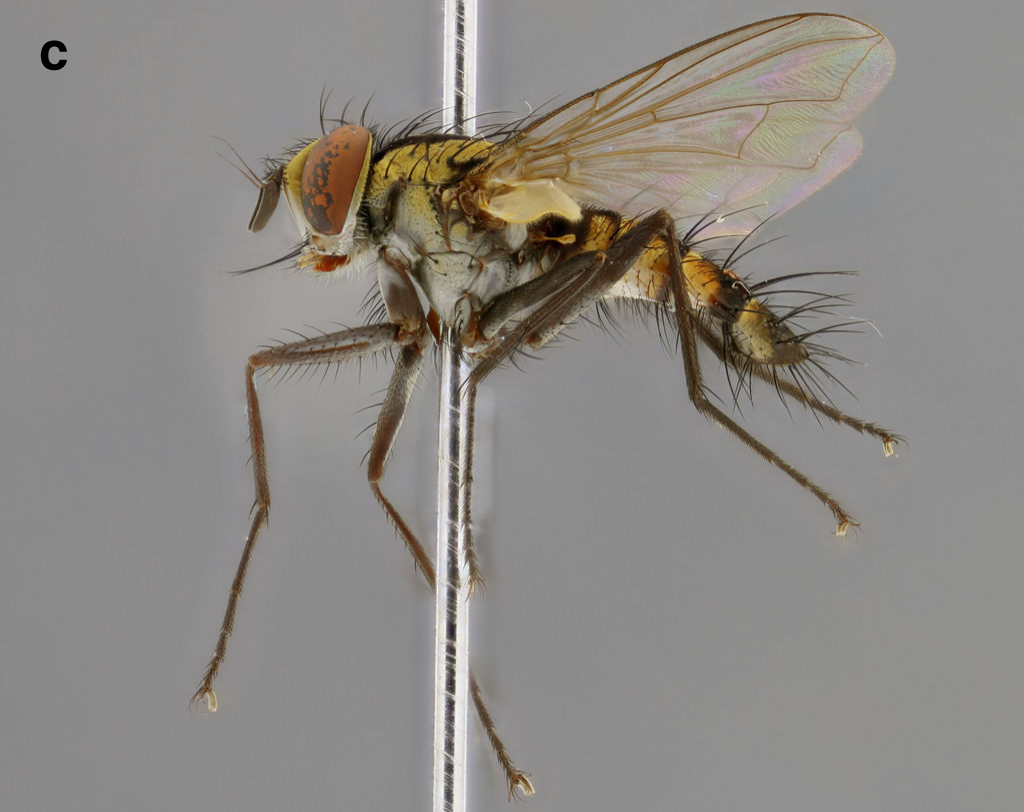
habitus in lateral view

**Figure 23d.**
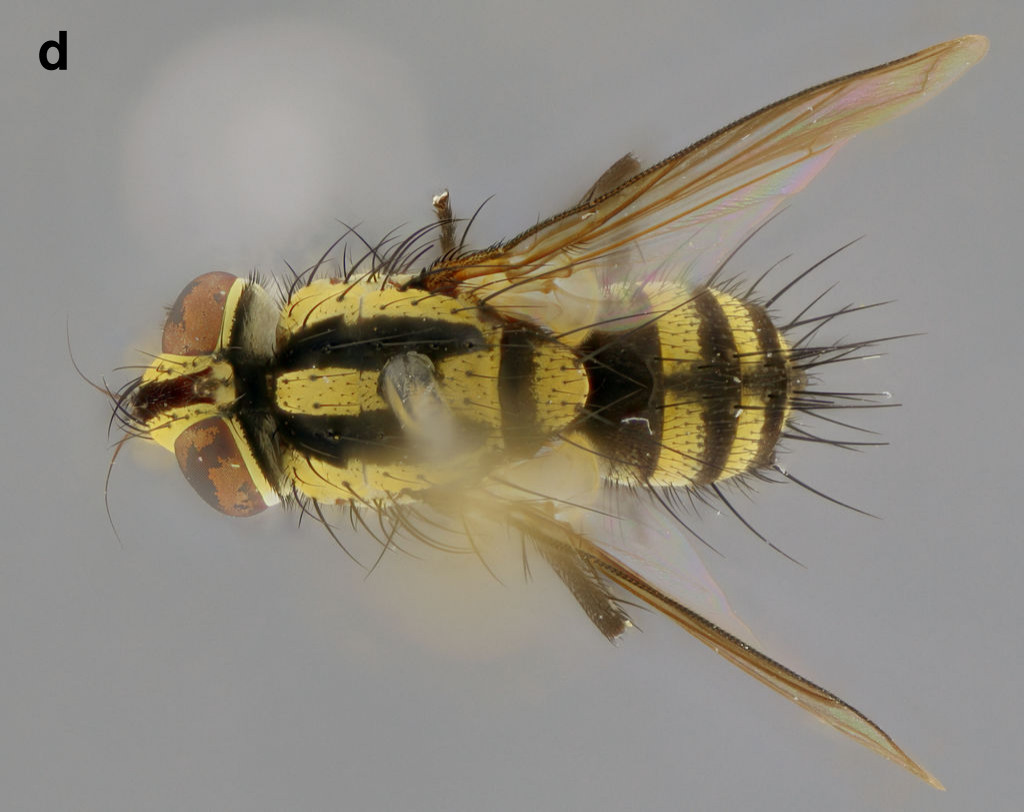
habitus in frontal view

**Figure 23e.**
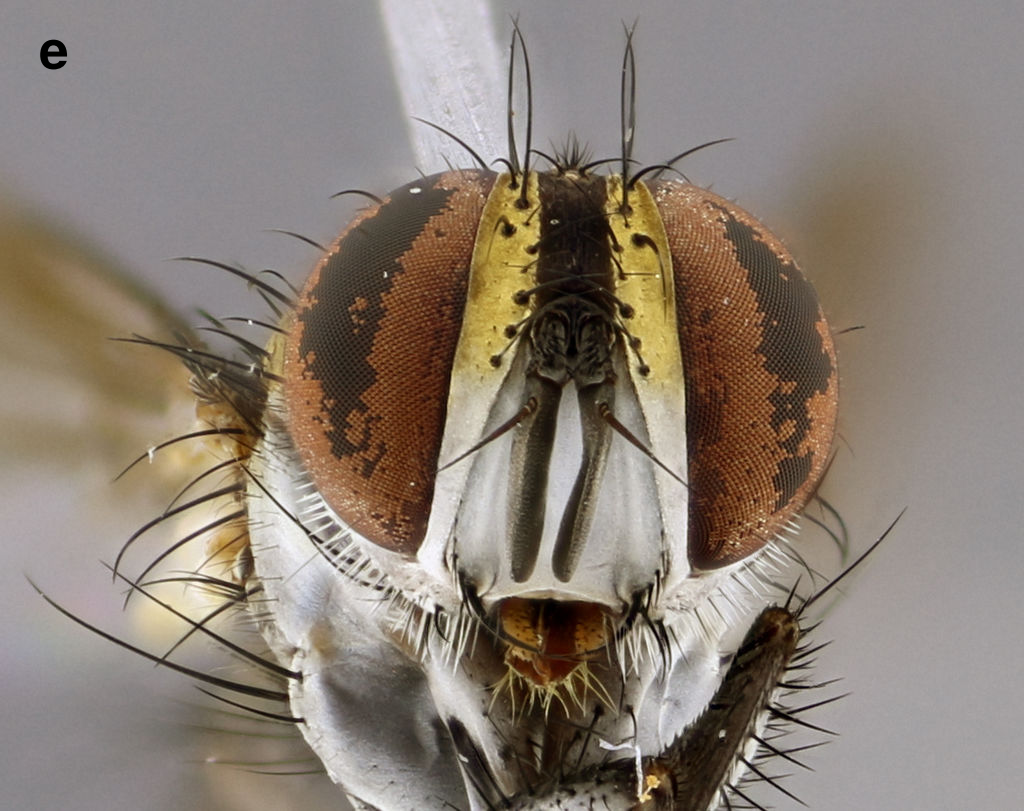
head in frontal view

**Figure 23f.**
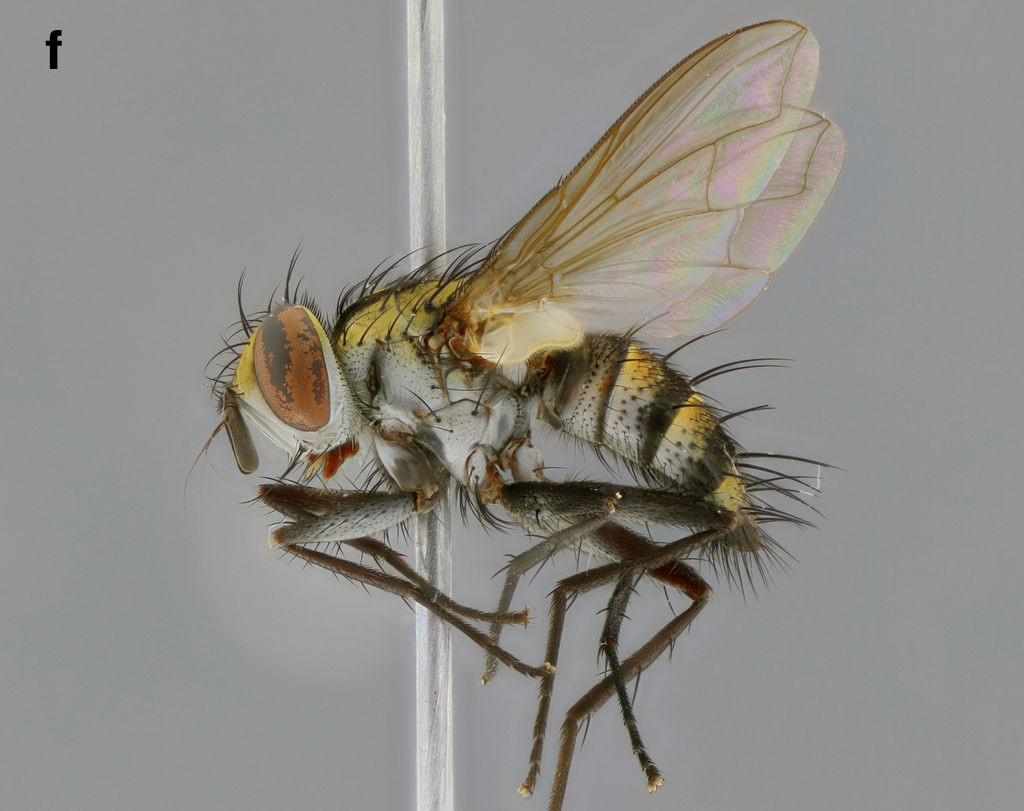
habitus in lateral view

**Figure 24a.**
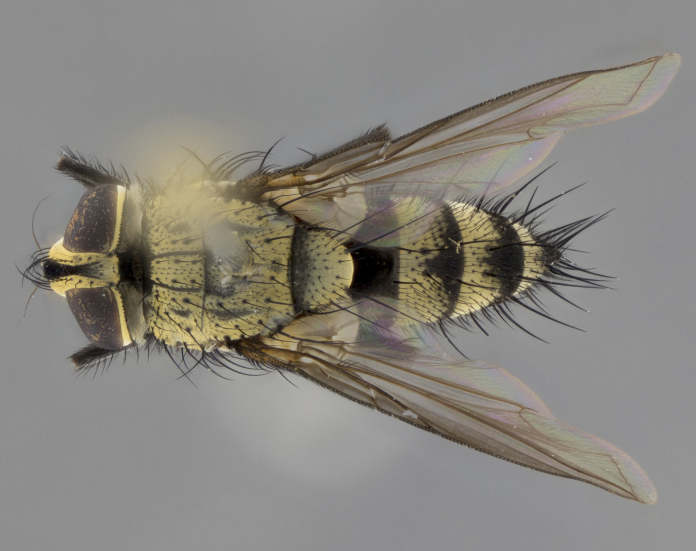
habitus in dorsal view

**Figure 24b.**
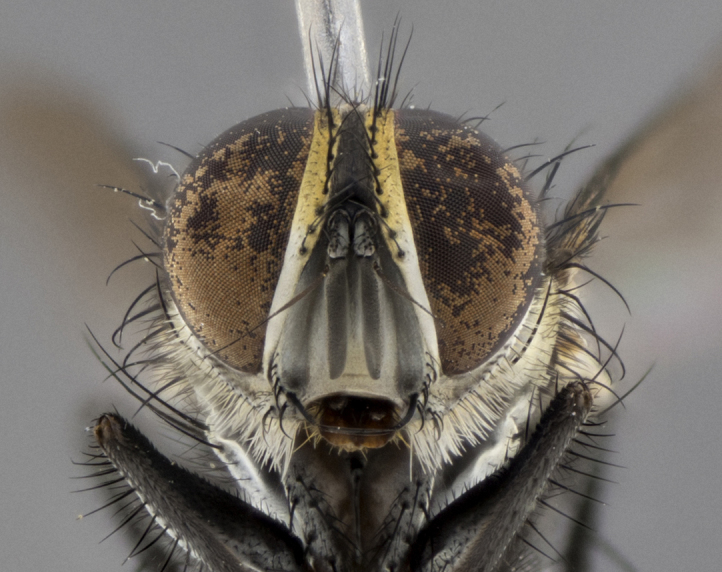
head in frontal view

**Figure 24c.**
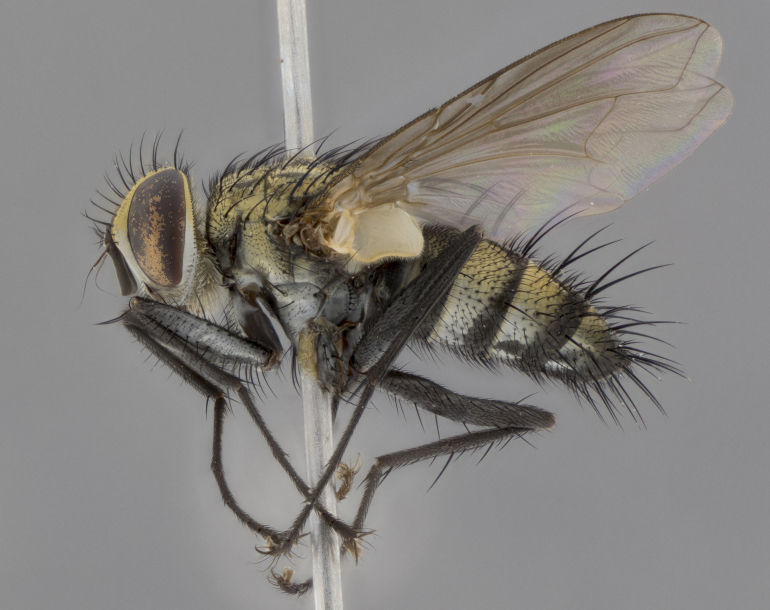
habitus in lateral view

**Figure 25a.**
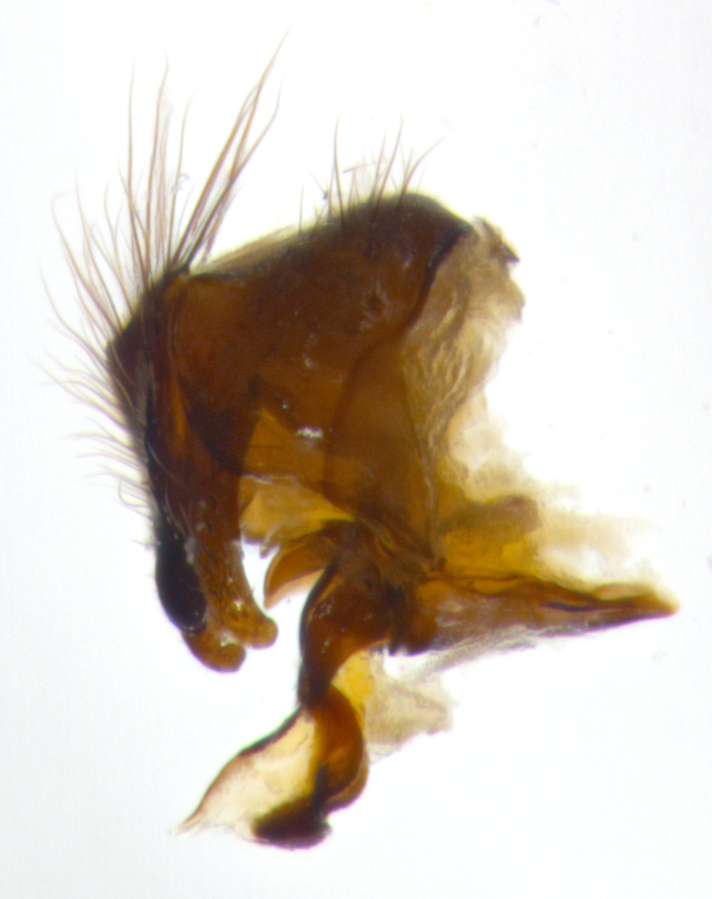
lateral view

**Figure 25b.**
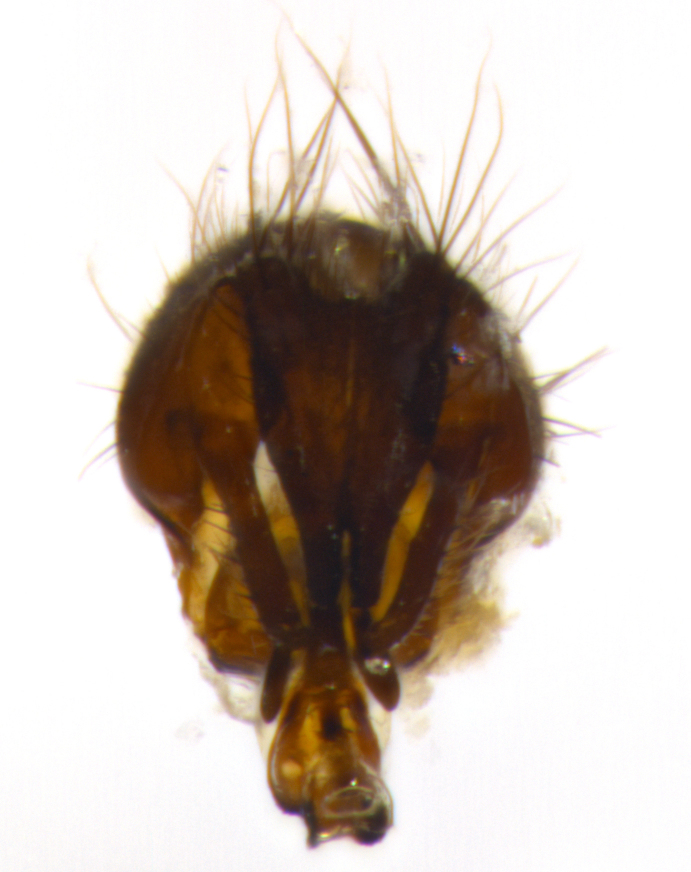
dorsal view

**Figure 25c.**
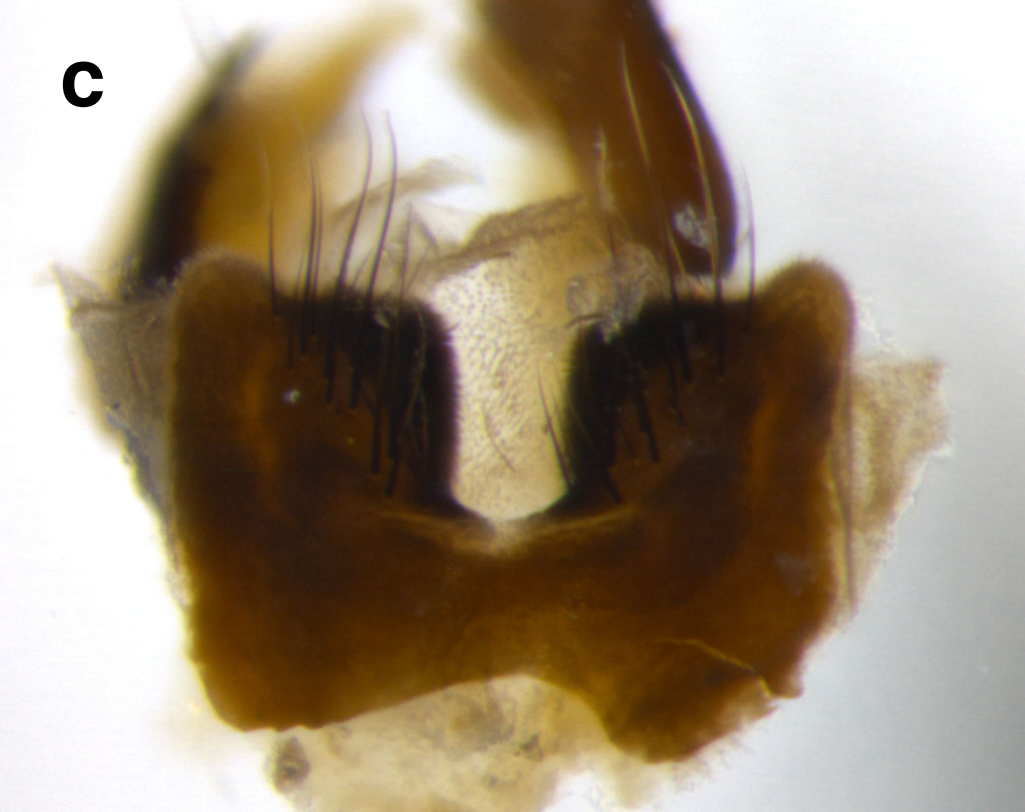
sternite 5 in ventral view

**Figure 26a.**
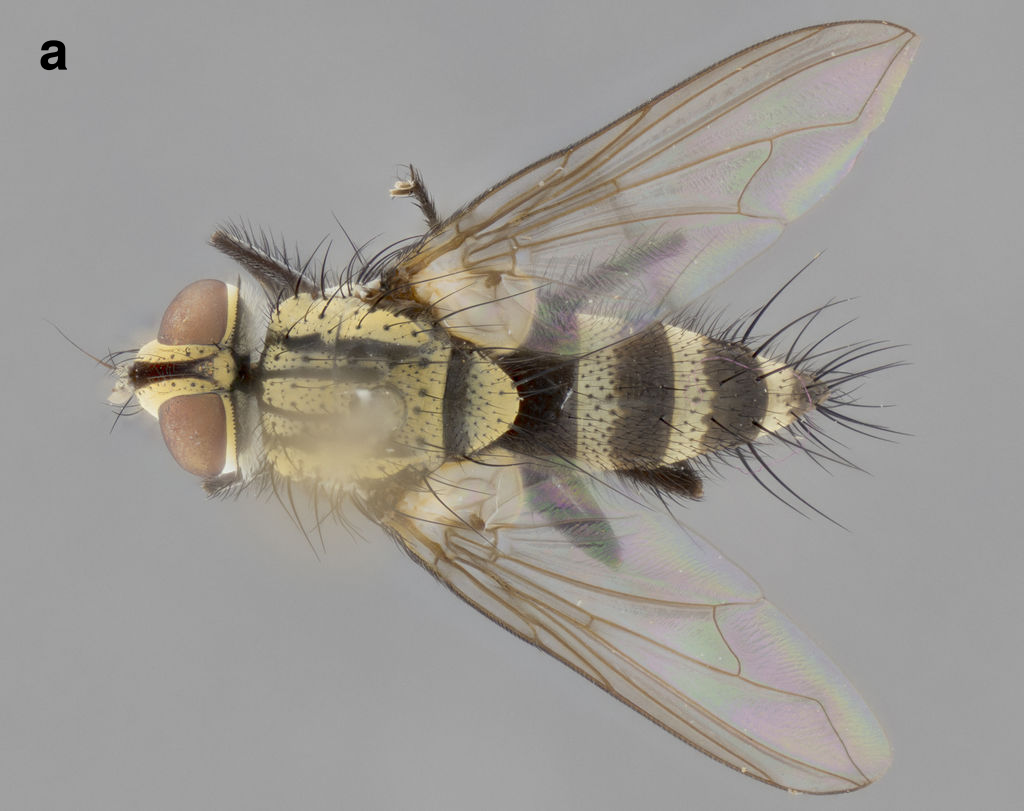
habitus in dorsal view

**Figure 26b.**
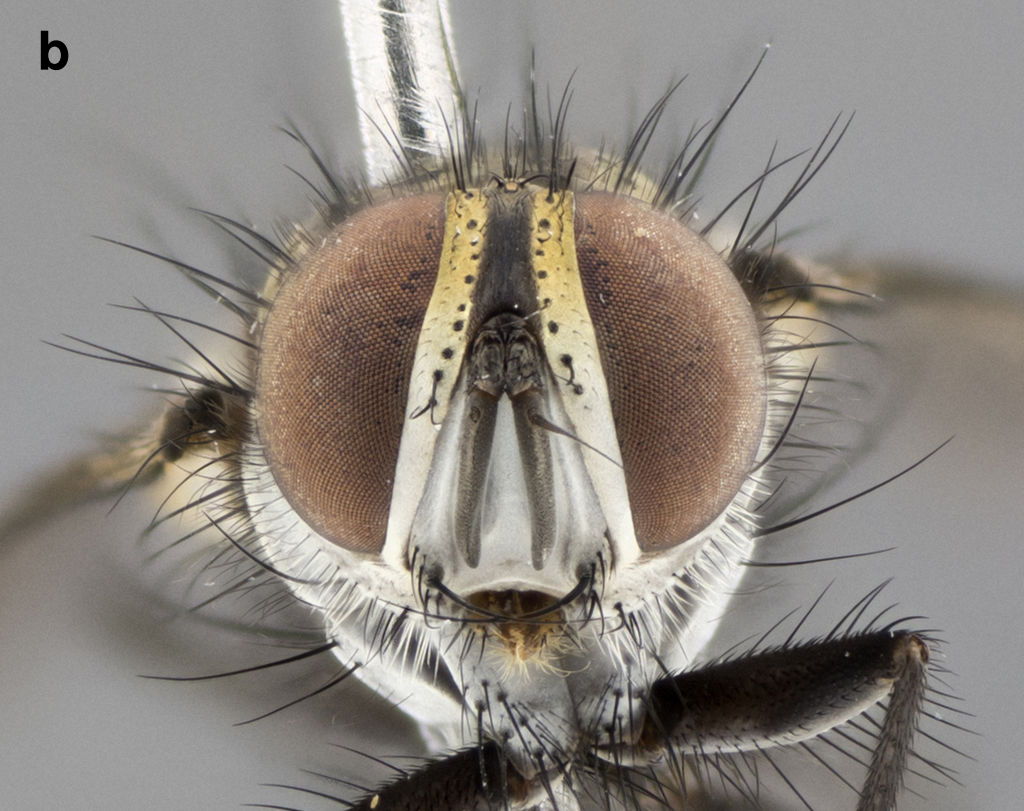
head in frontal view

**Figure 26c.**
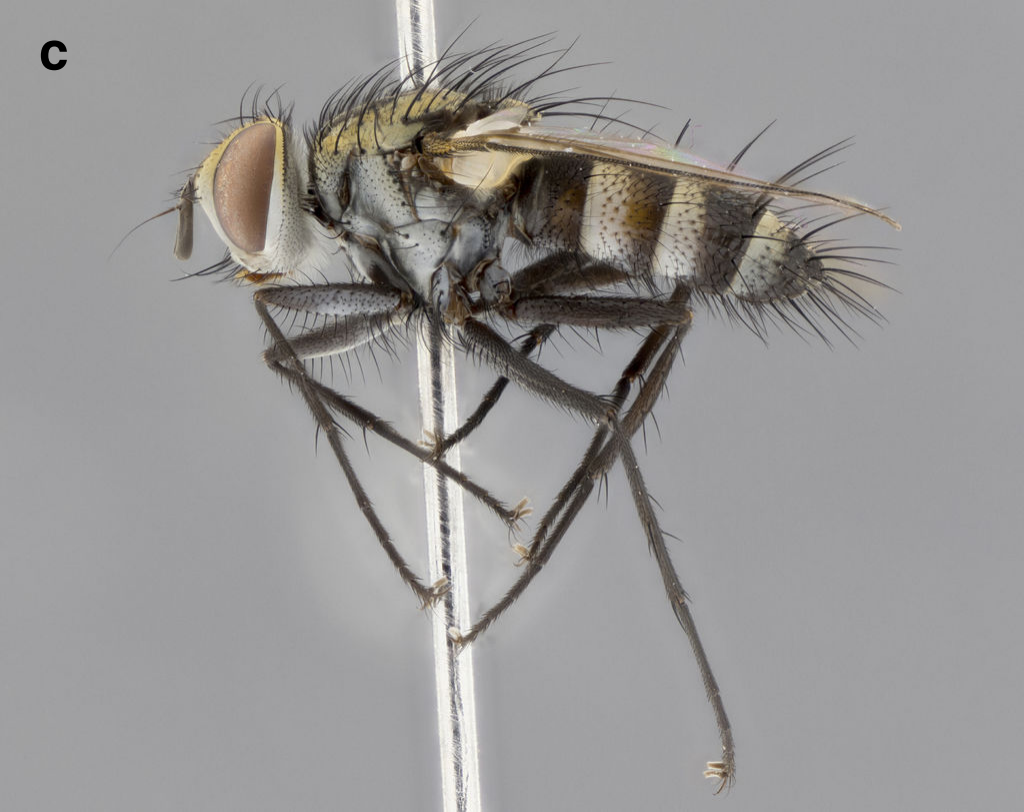
habitus in lateral view

**Figure 26d.**
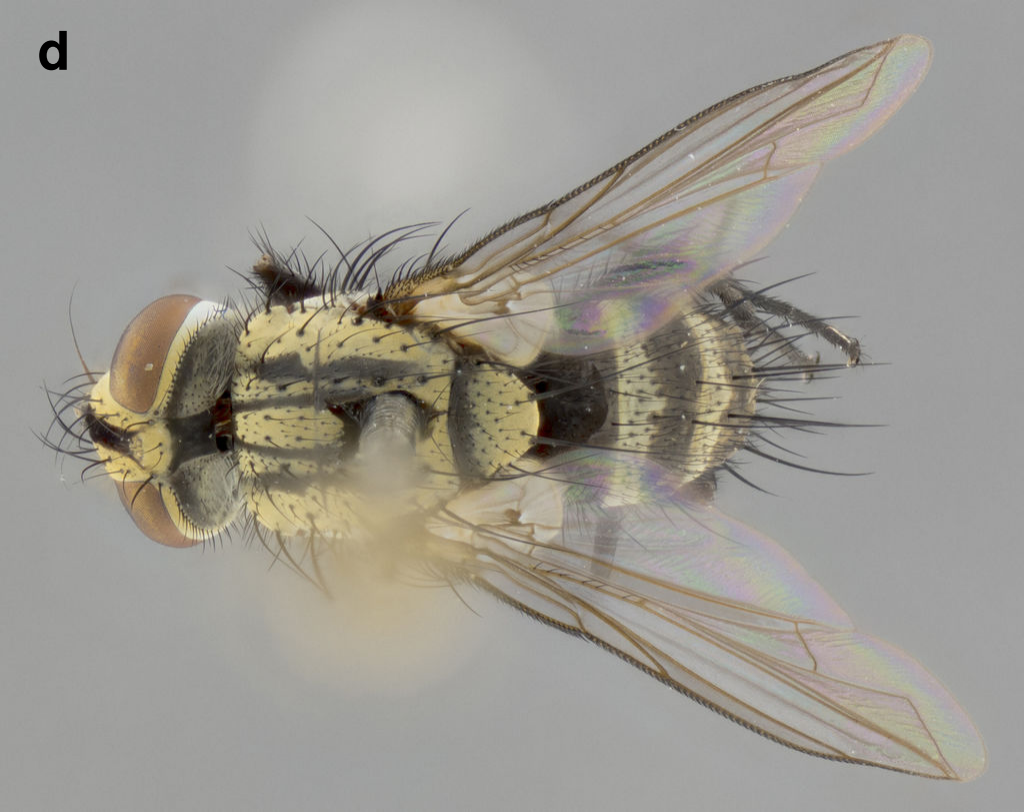
habitus in dorsal view

**Figure 26e.**
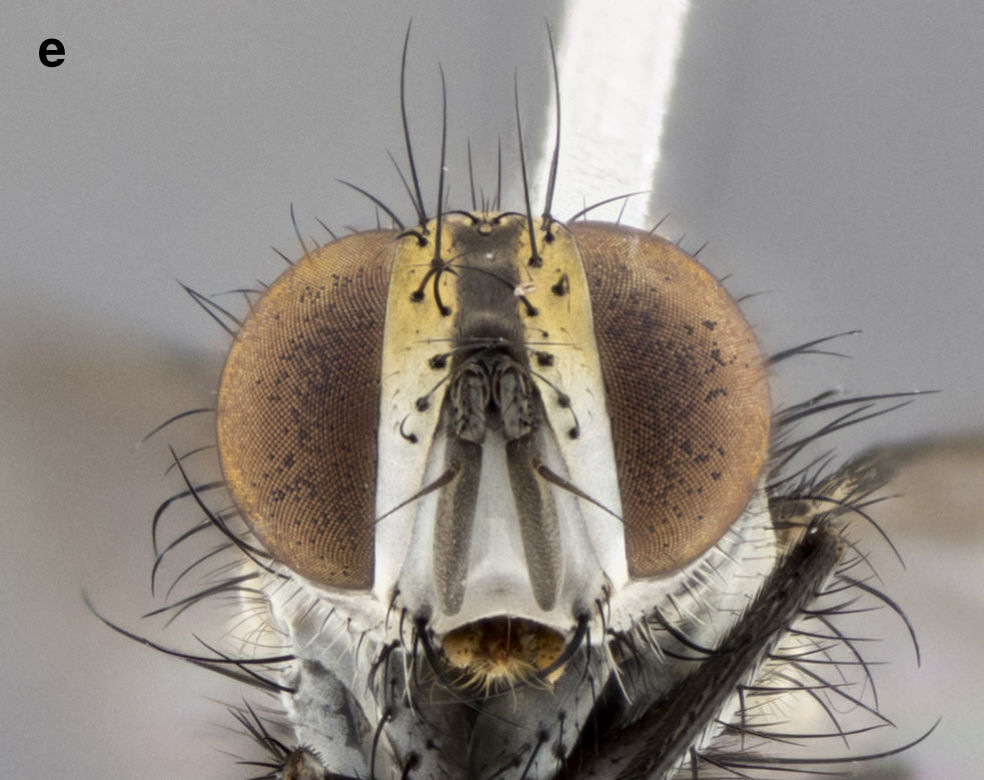
head in frontal view

**Figure 26f.**
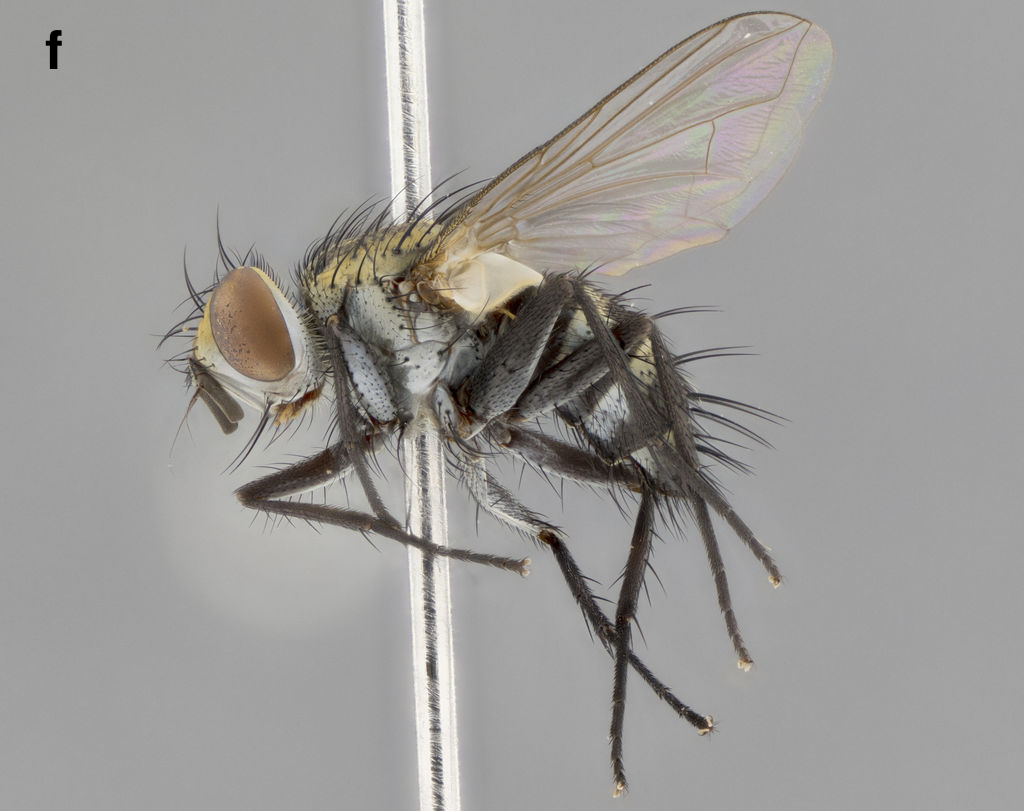
habitus in lateral view

**Figure 27a.**
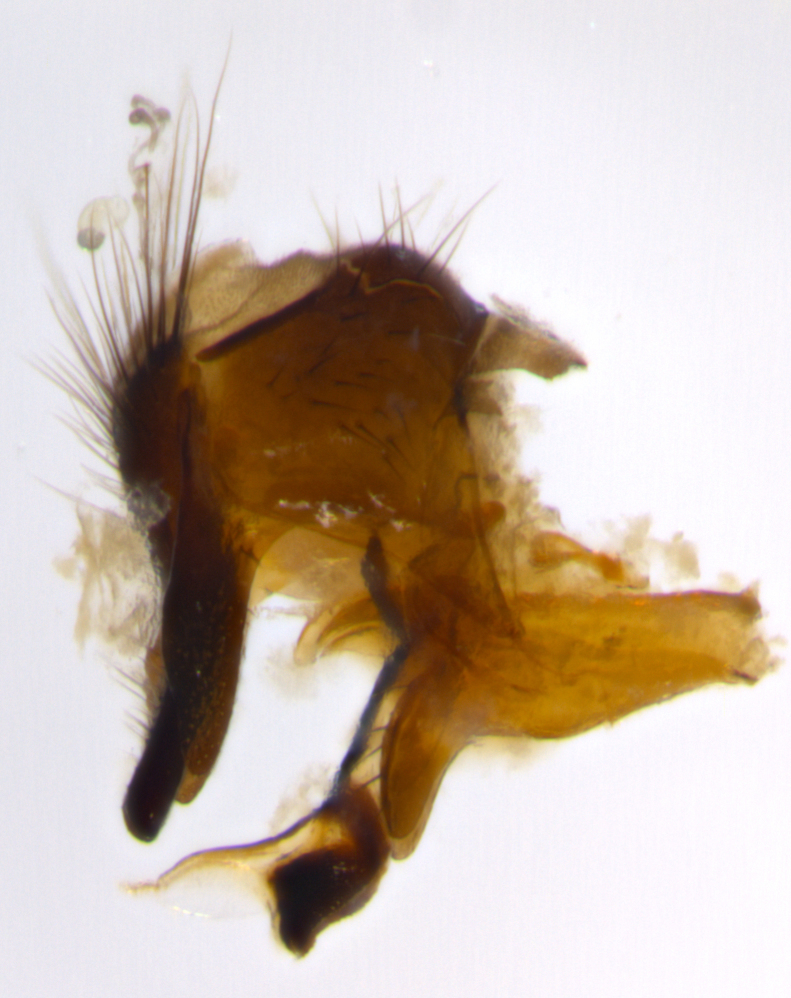
lateral view

**Figure 27b.**
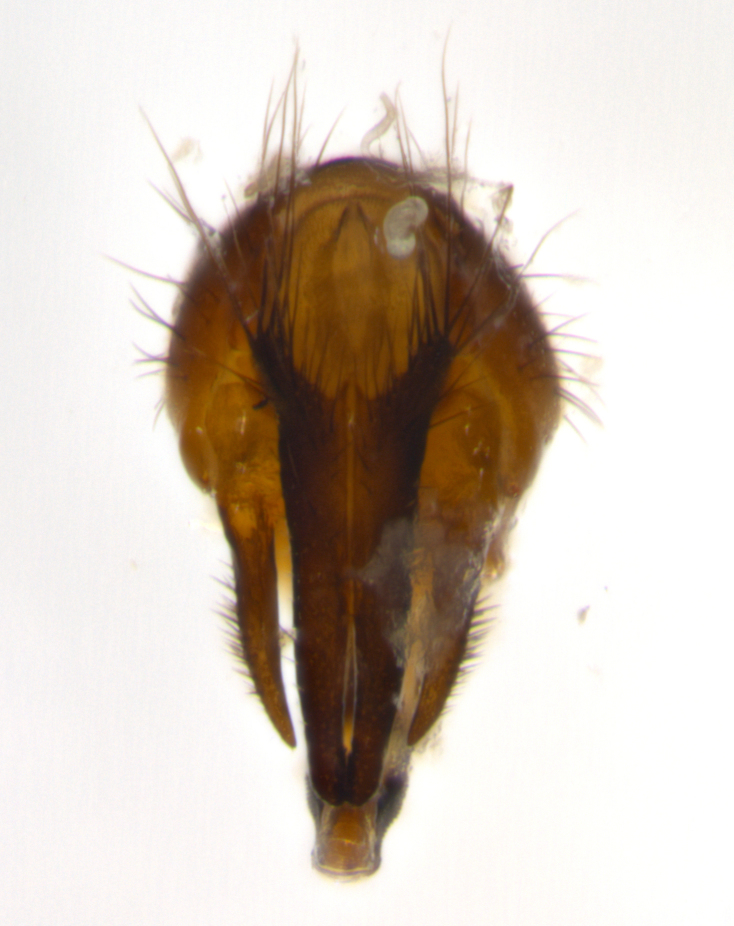
dorsal view

**Figure 27c.**
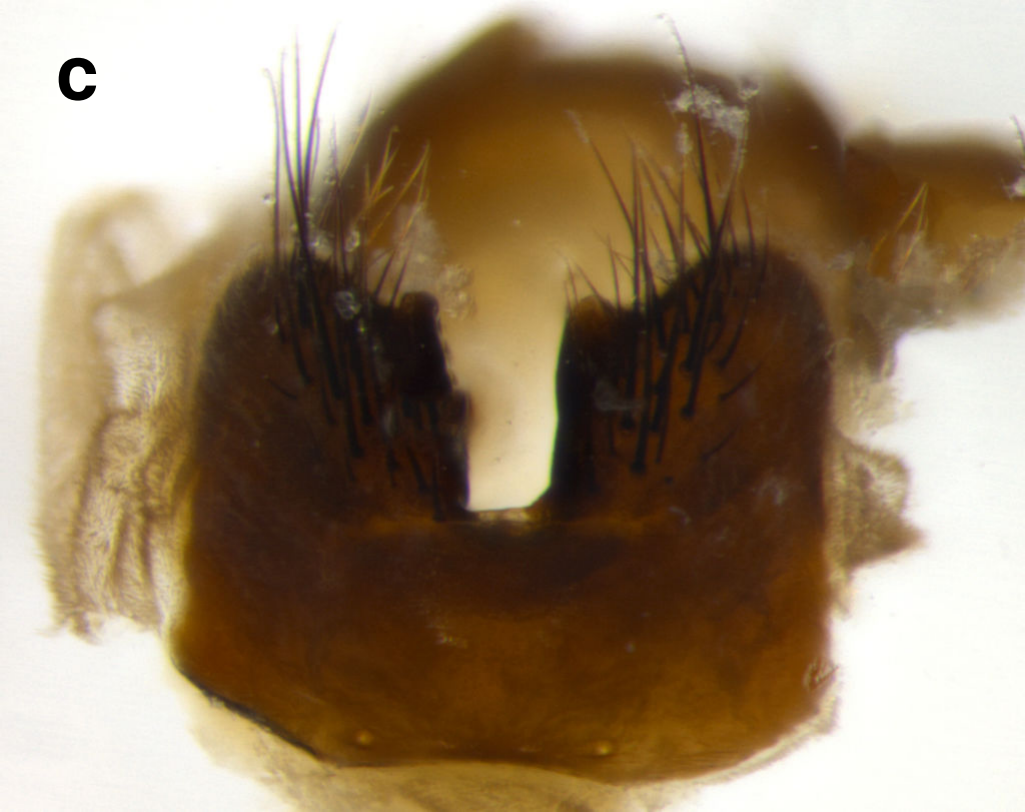
sternite 5 in ventral view

**Figure 28a.**
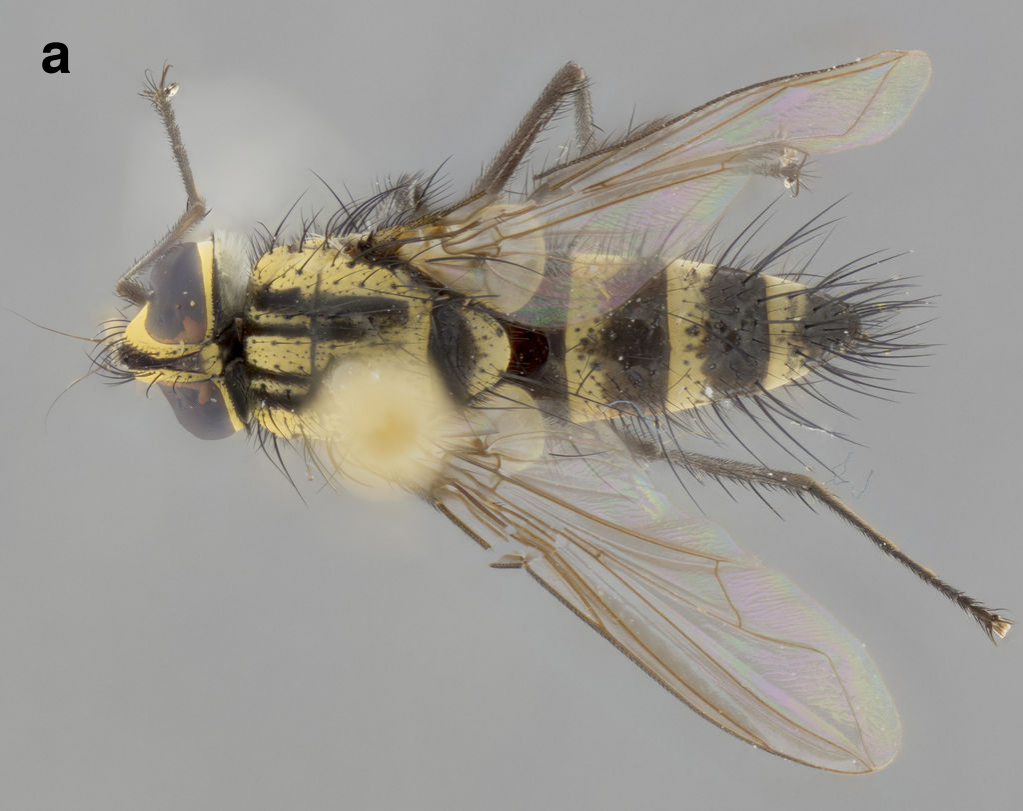
habitus in dorsal view

**Figure 28b.**
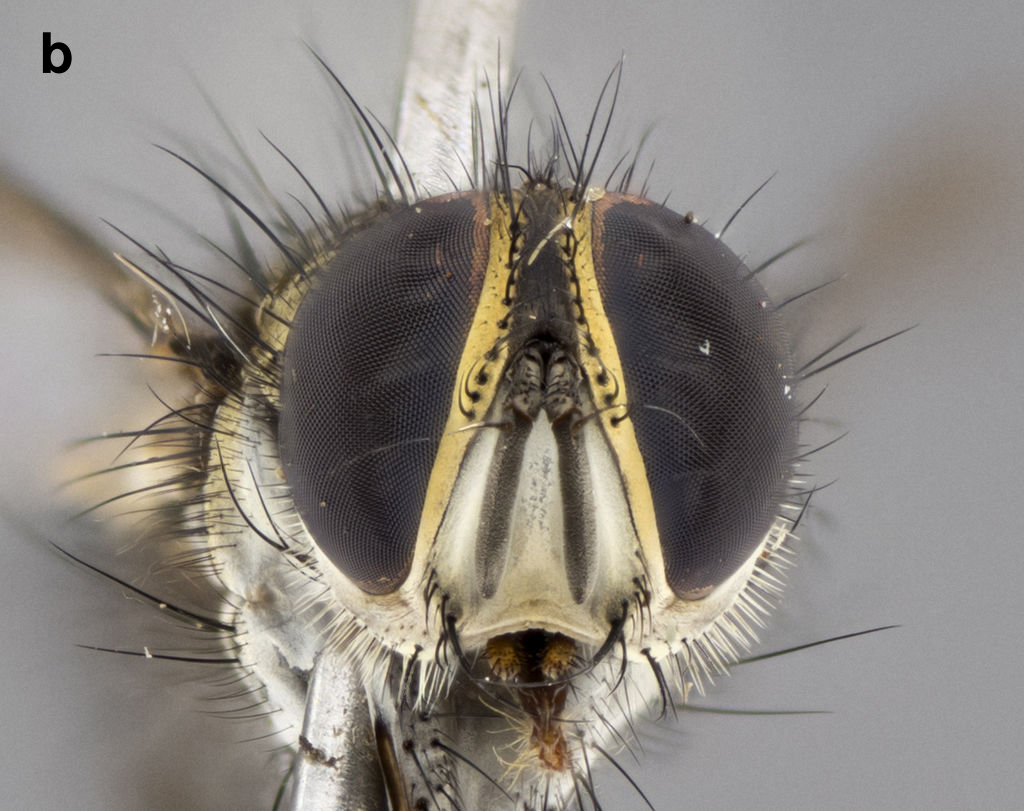
head in frontal view

**Figure 28c.**
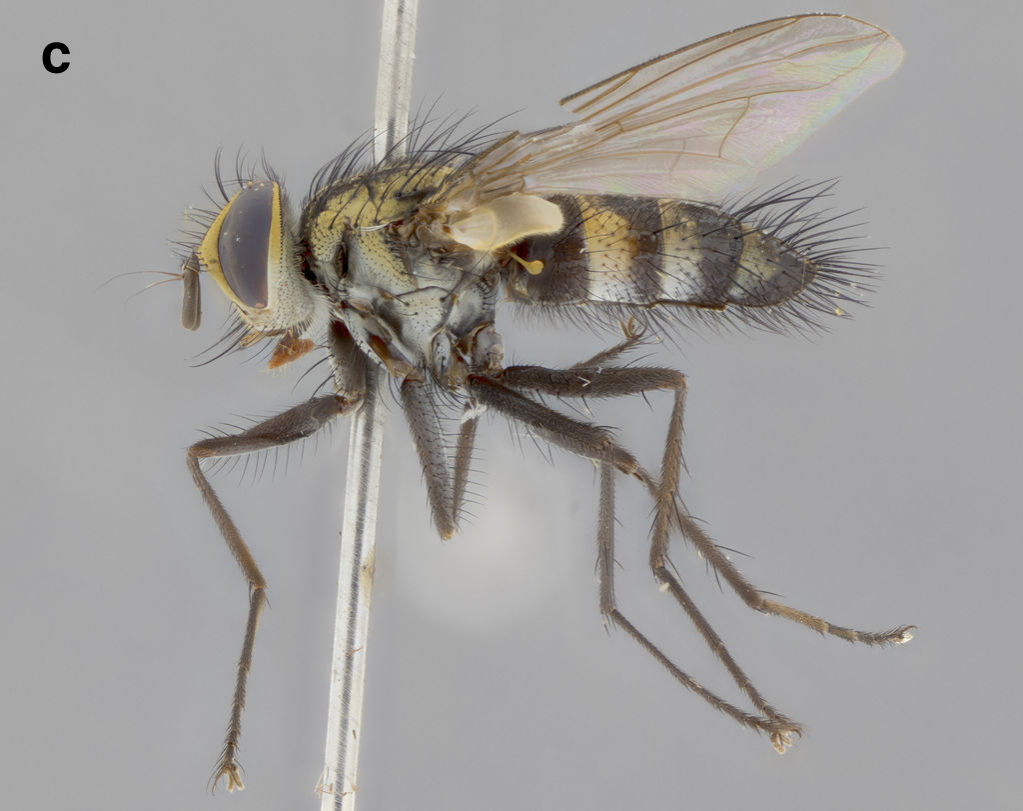
habitus in lateral view

**Figure 28d.**
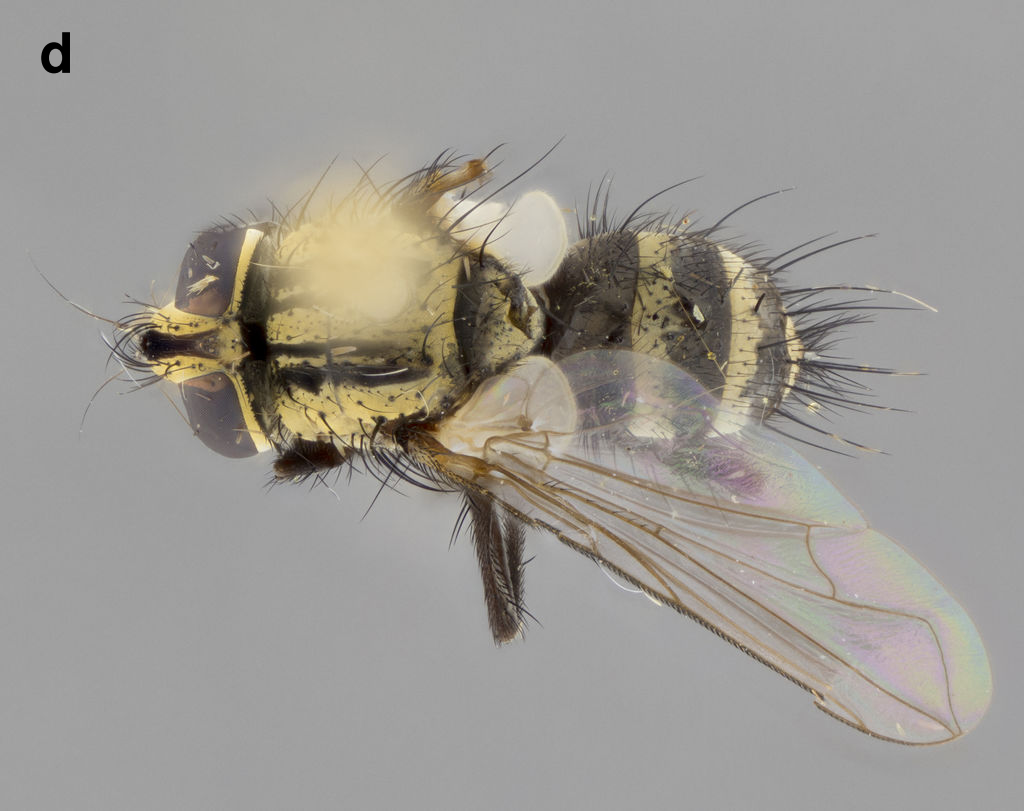
habitus in dorsal view

**Figure 28e.**
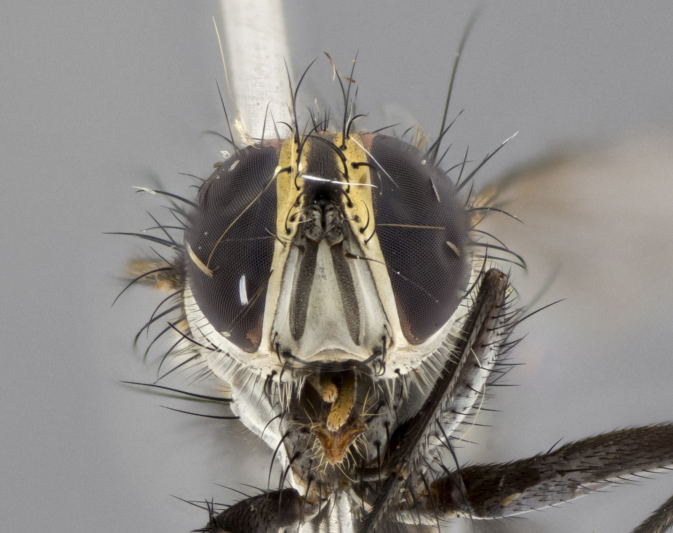
head in frontal view

**Figure 28f.**
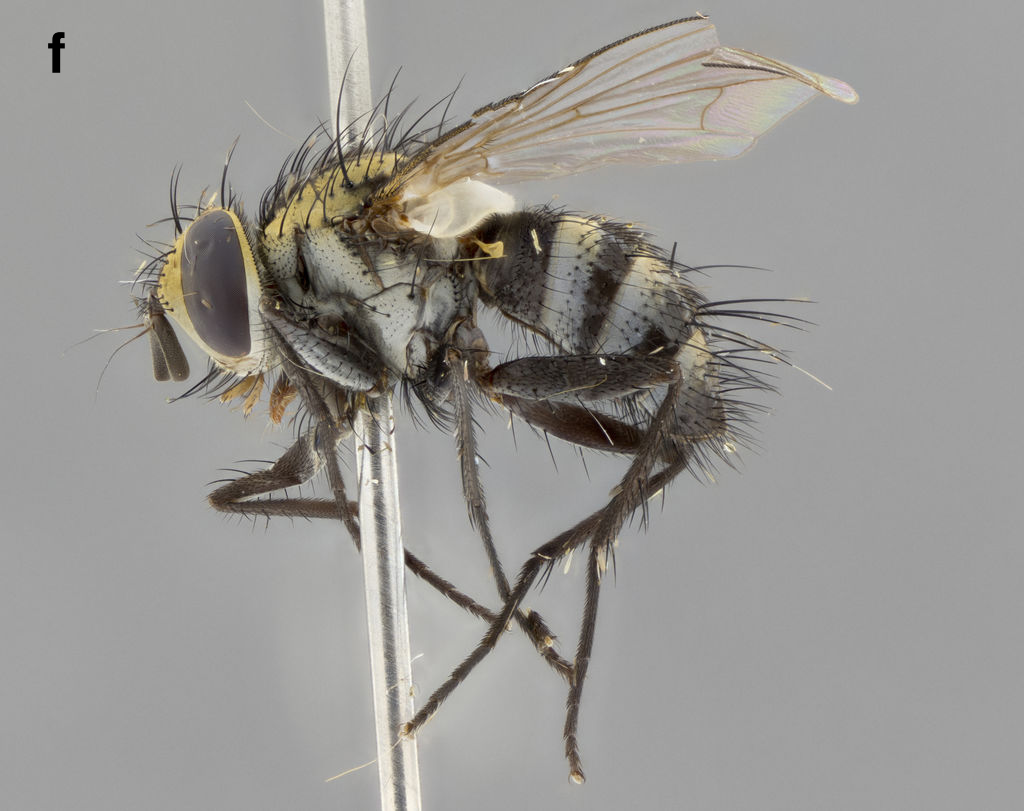
habitus in lateral view

**Figure 29a.**
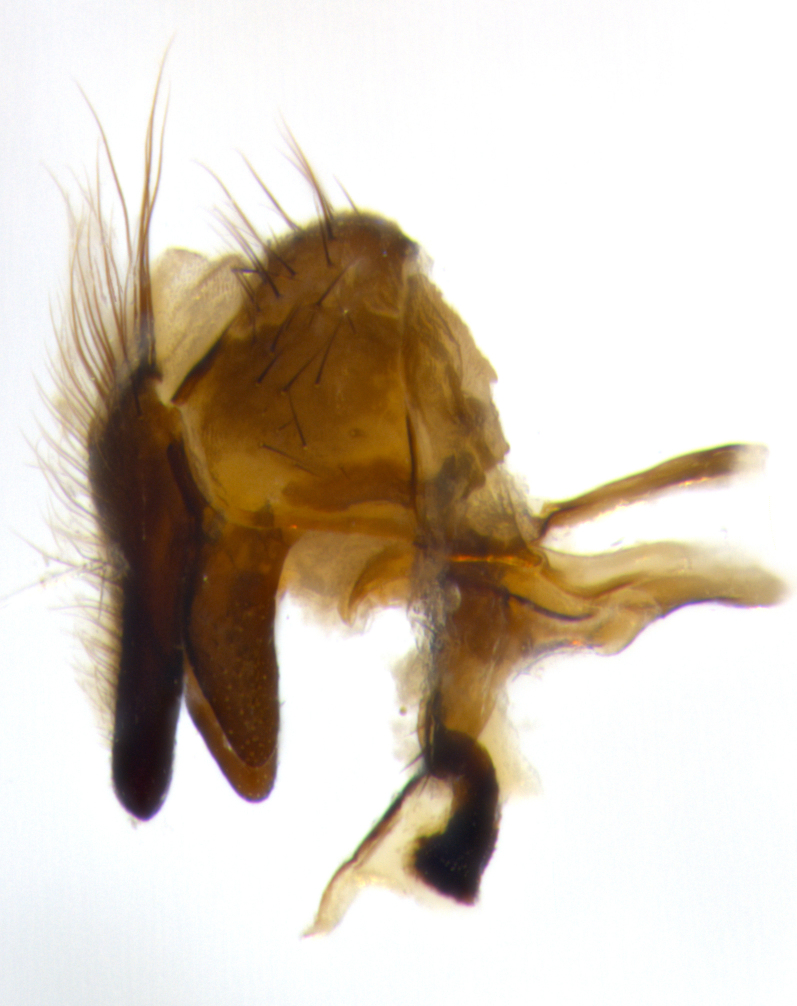
lateral view

**Figure 29b.**
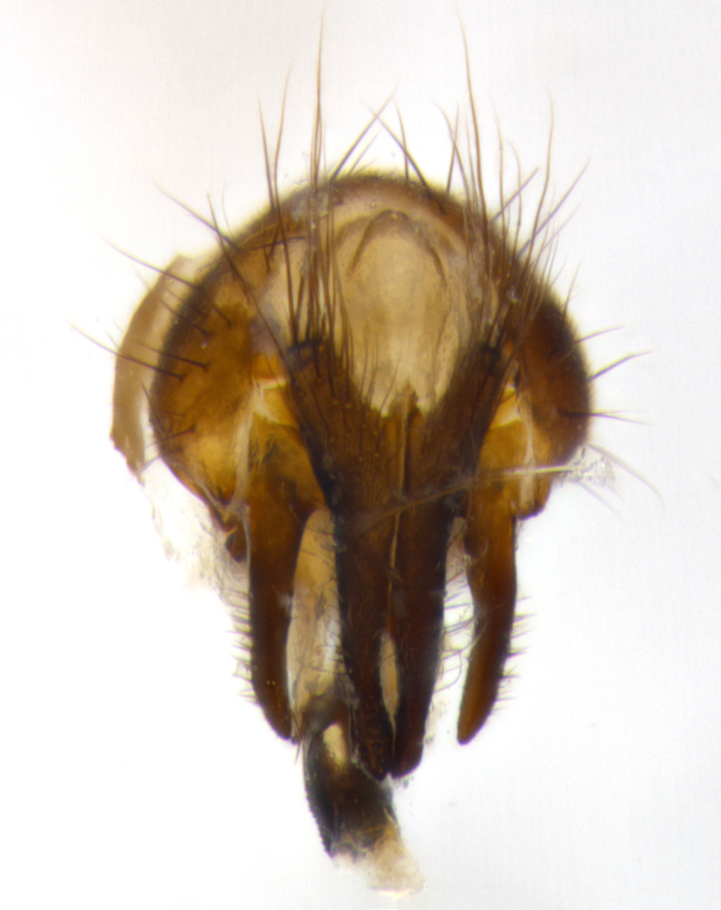
dorsal view

**Figure 29c.**
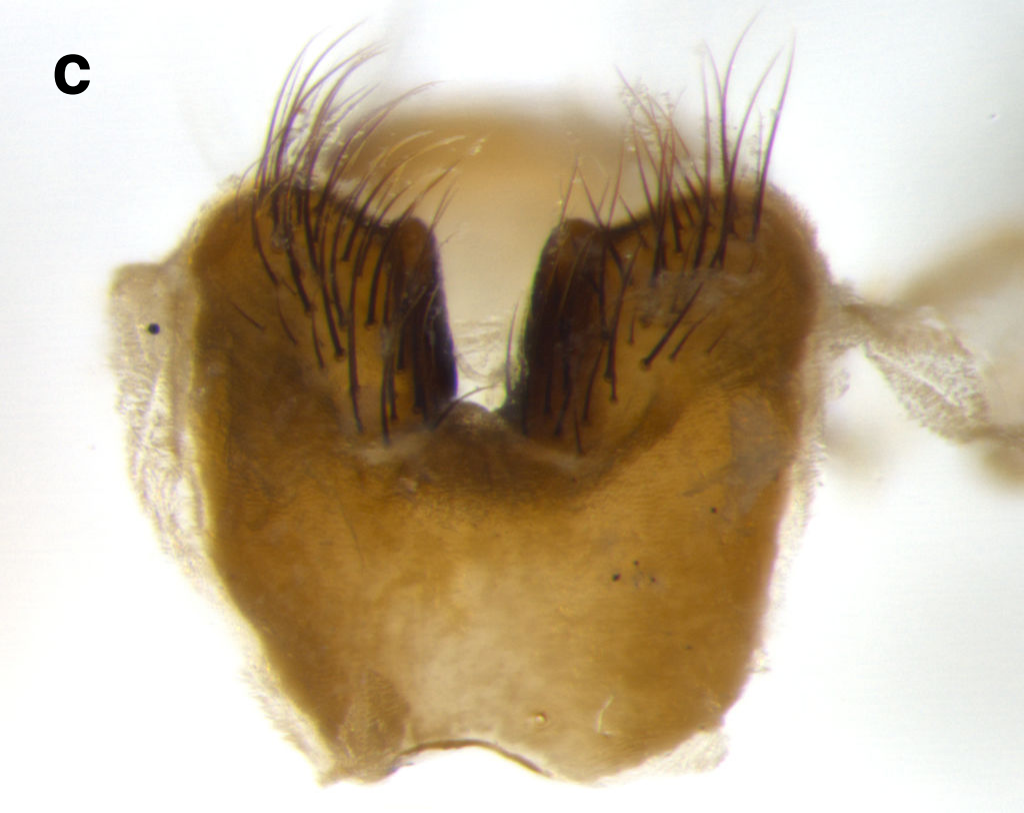
sternite 5 in ventral view

**Figure 30a.**
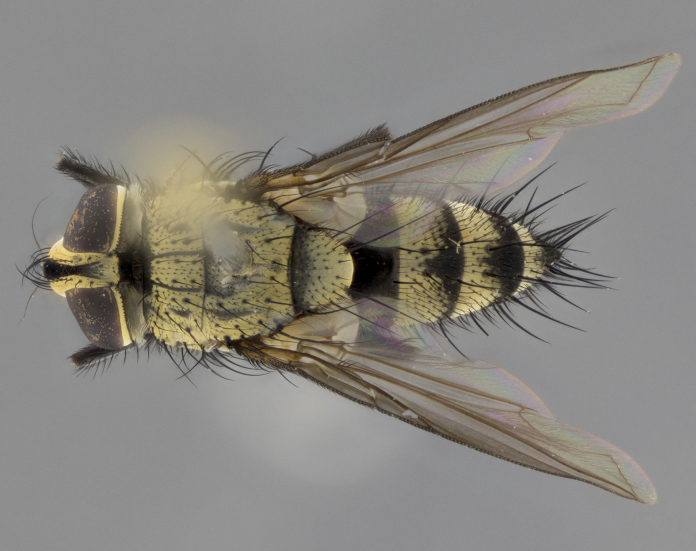
habitus in lateral view

**Figure 30b.**
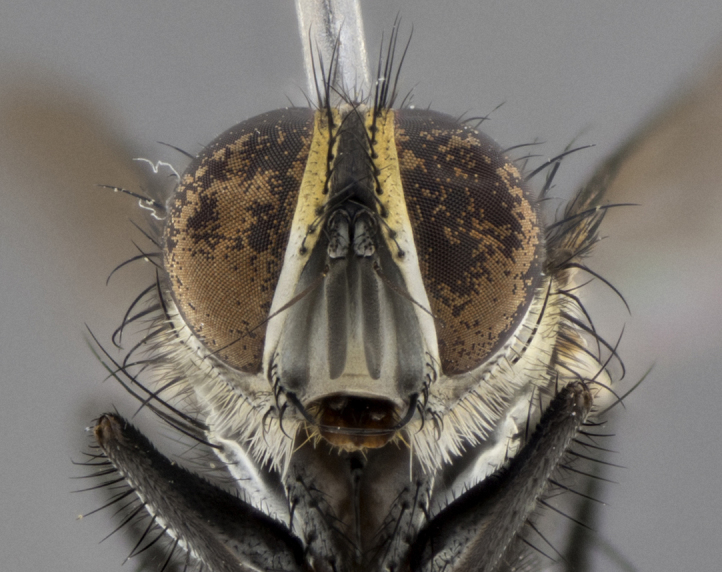
head in frontal view

**Figure 30c.**
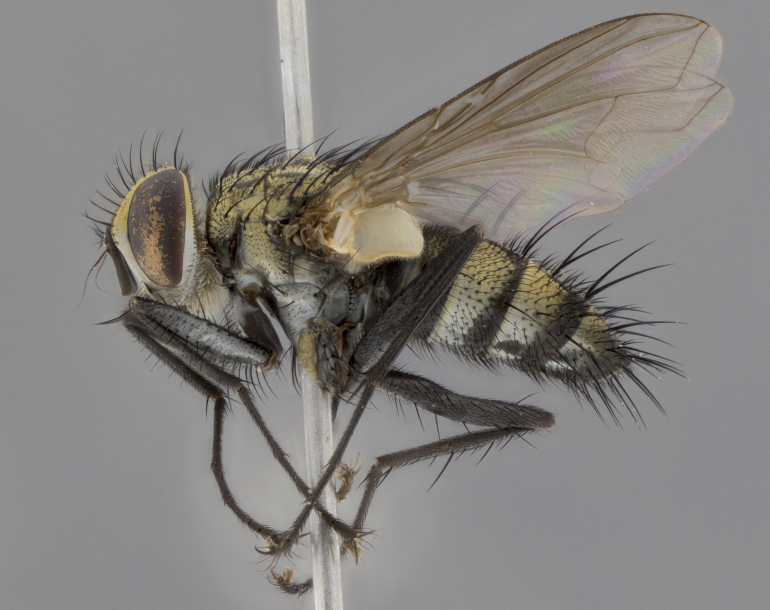
habitus in lateral view

**Figure 31a.**
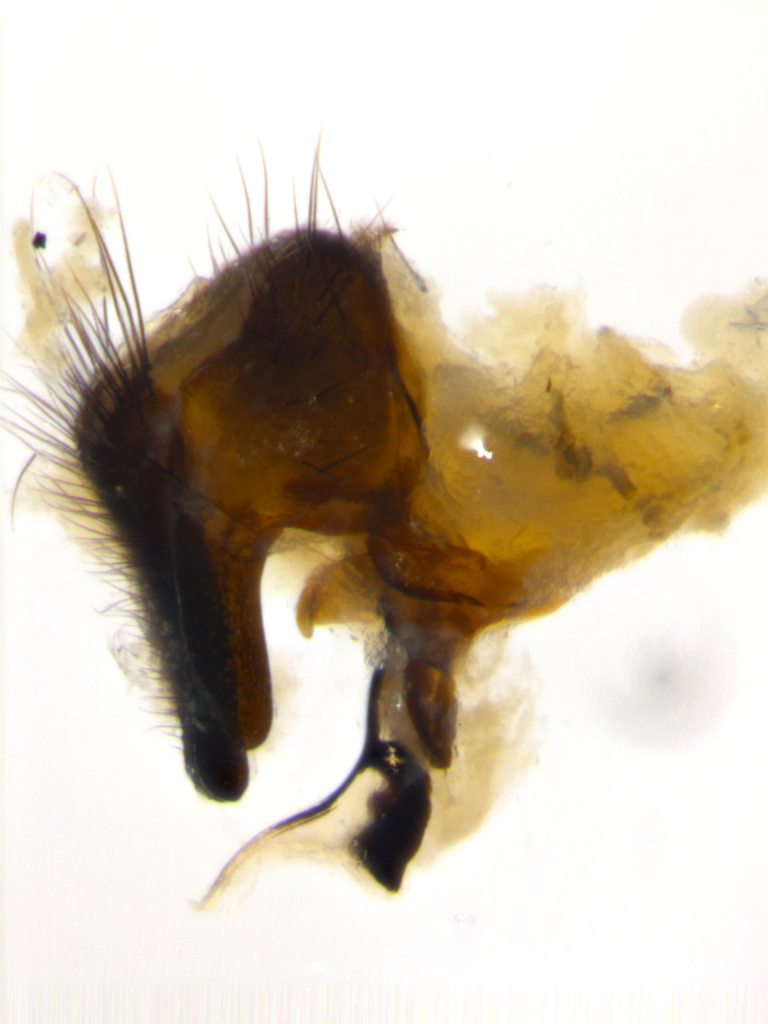
lateral view

**Figure 31b.**
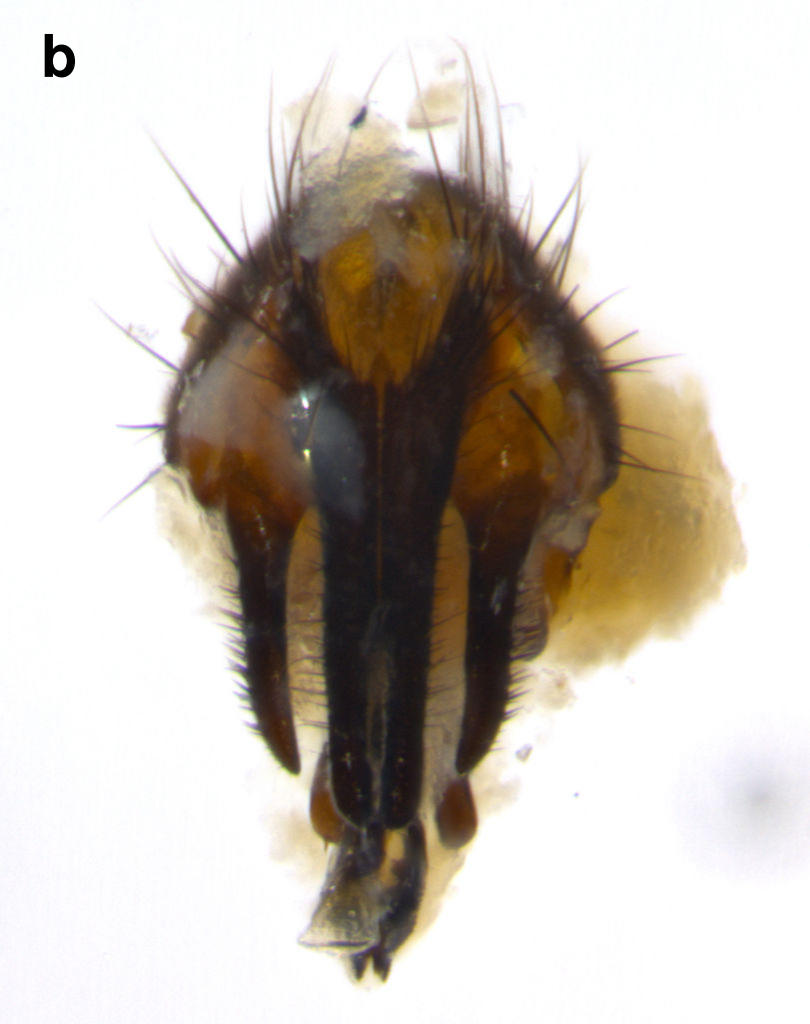
dorsal view

**Figure 31c.**
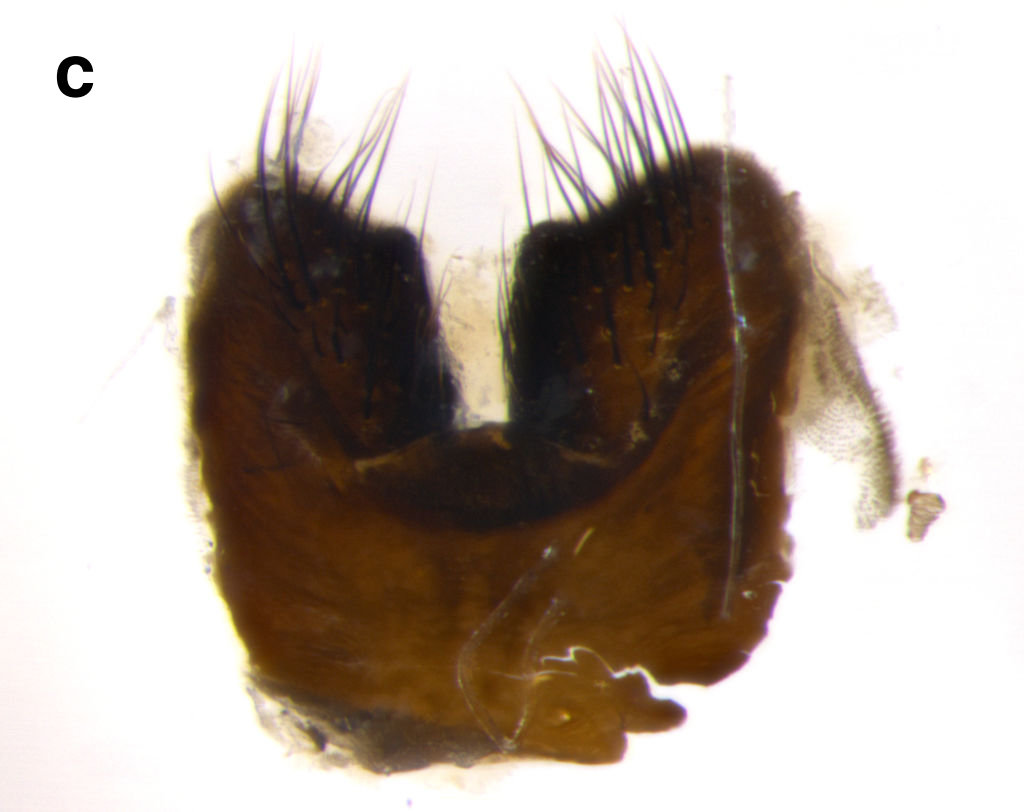
sternite 5 in ventral view

**Figure 32a.**
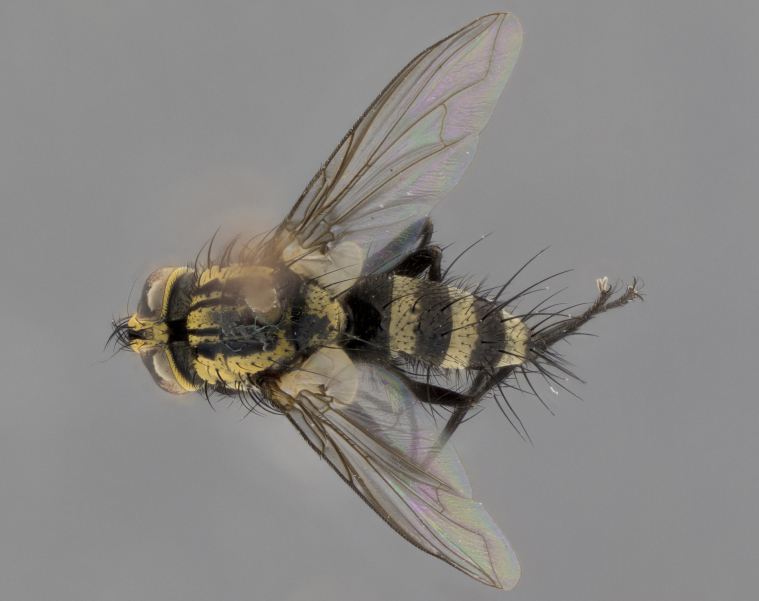
habitus in dorsal view

**Figure 32b.**
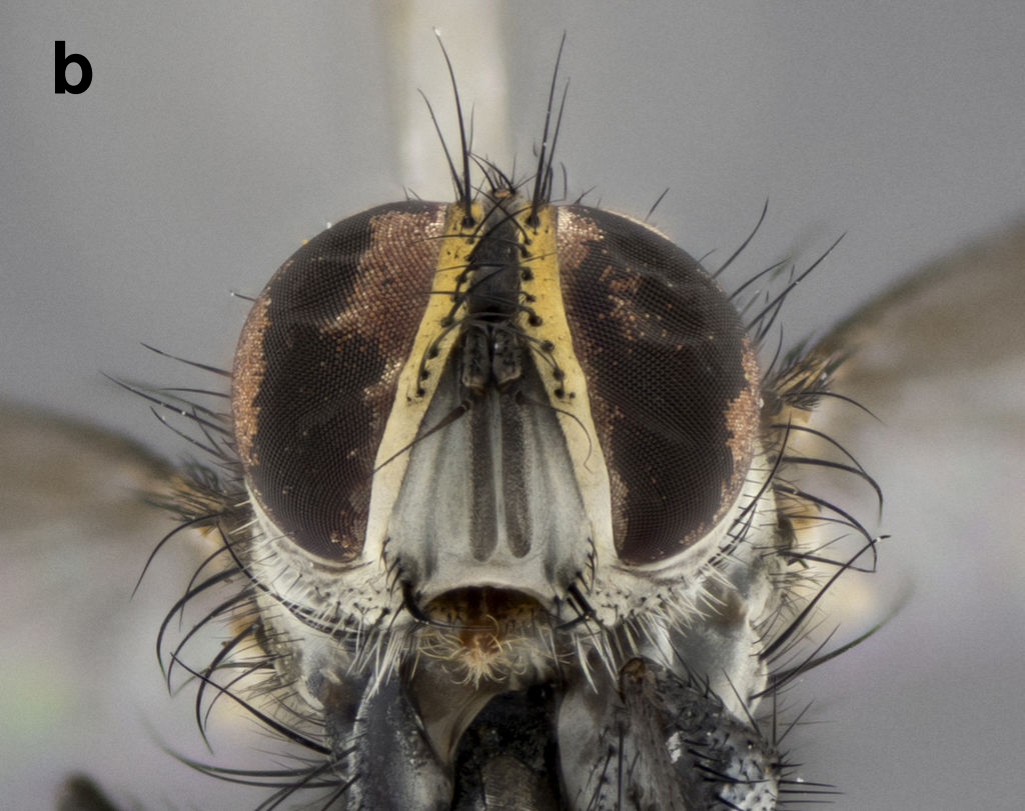
head in frontal view

**Figure 32c.**
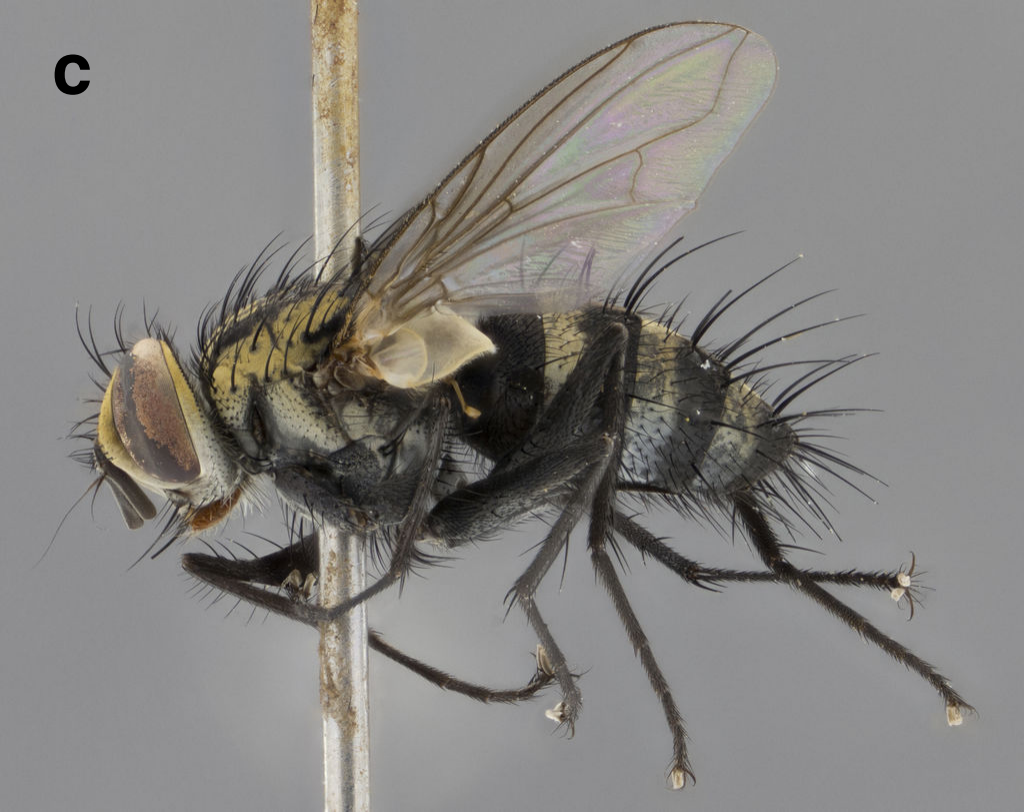
habitus in lateral view

**Figure 32d.**
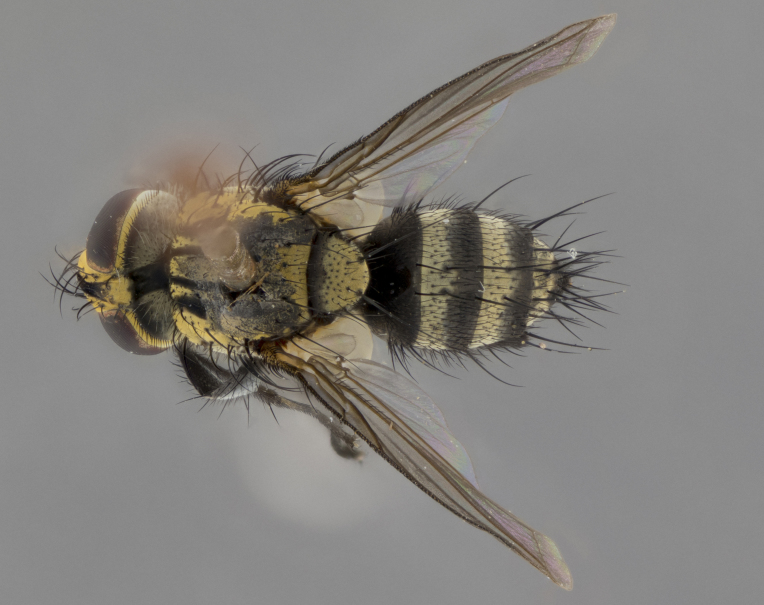
habitus in dorsal view

**Figure 32e.**
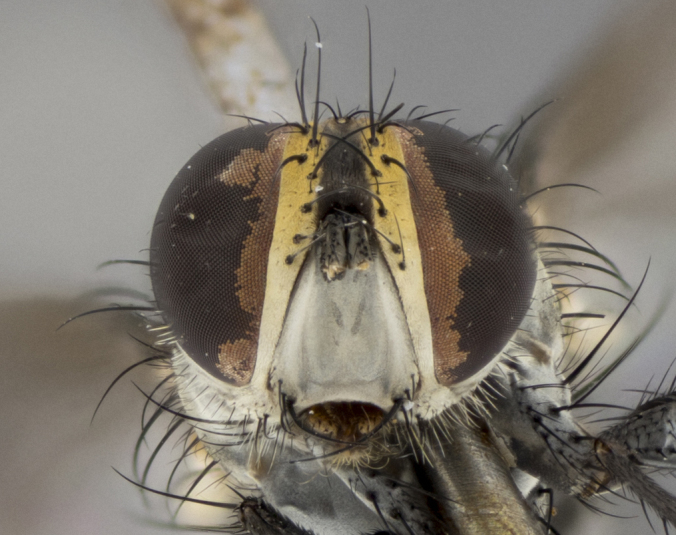
head in frontal view

**Figure 32f.**
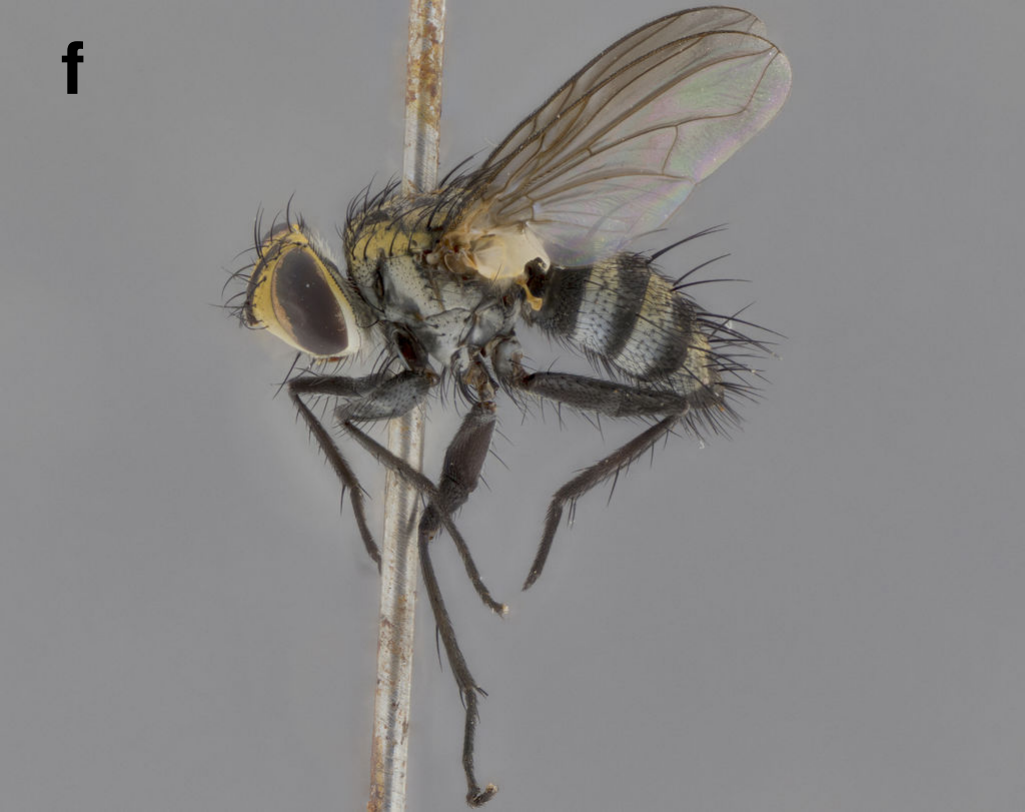
habitus in lateral view

**Figure 33a.**
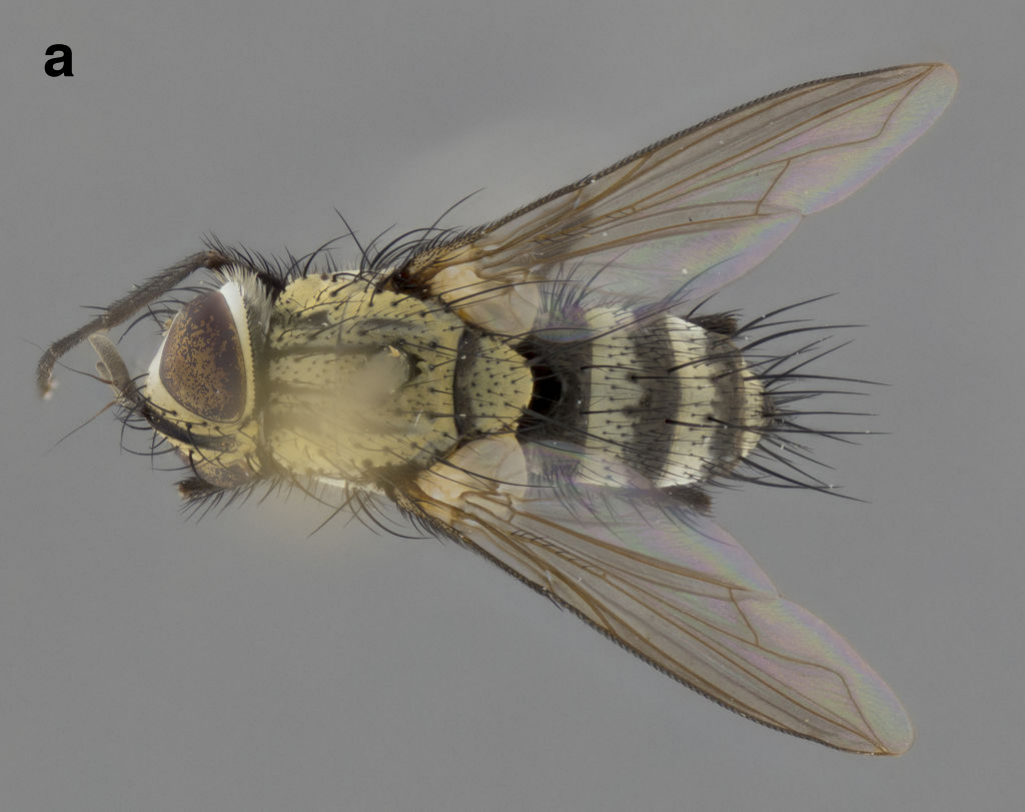
habitus in lateral view

**Figure 33b.**
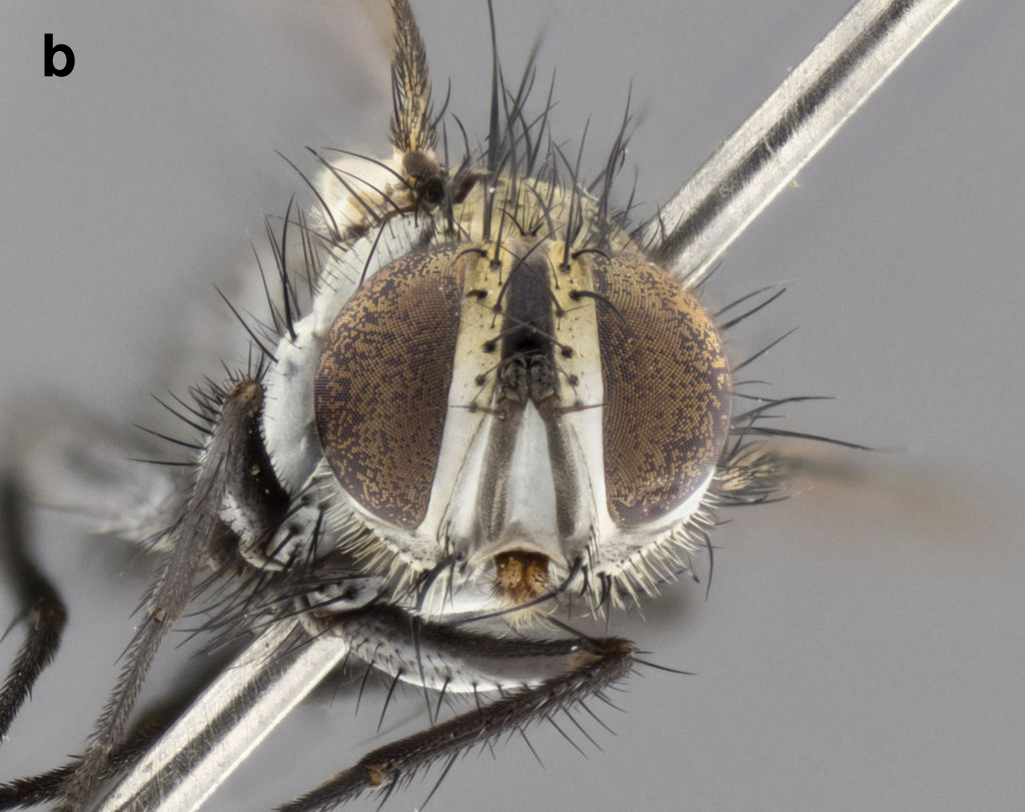
head in frontal view

**Figure 33c.**
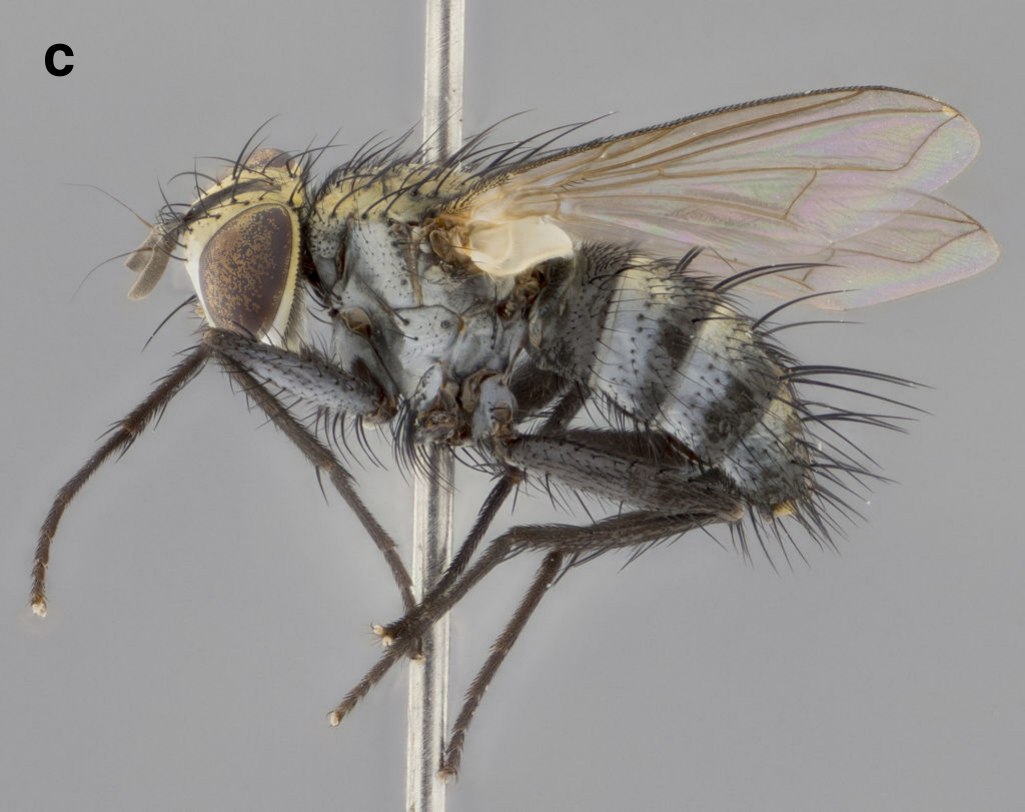
habitus in lateral view

**Figure 34a.**
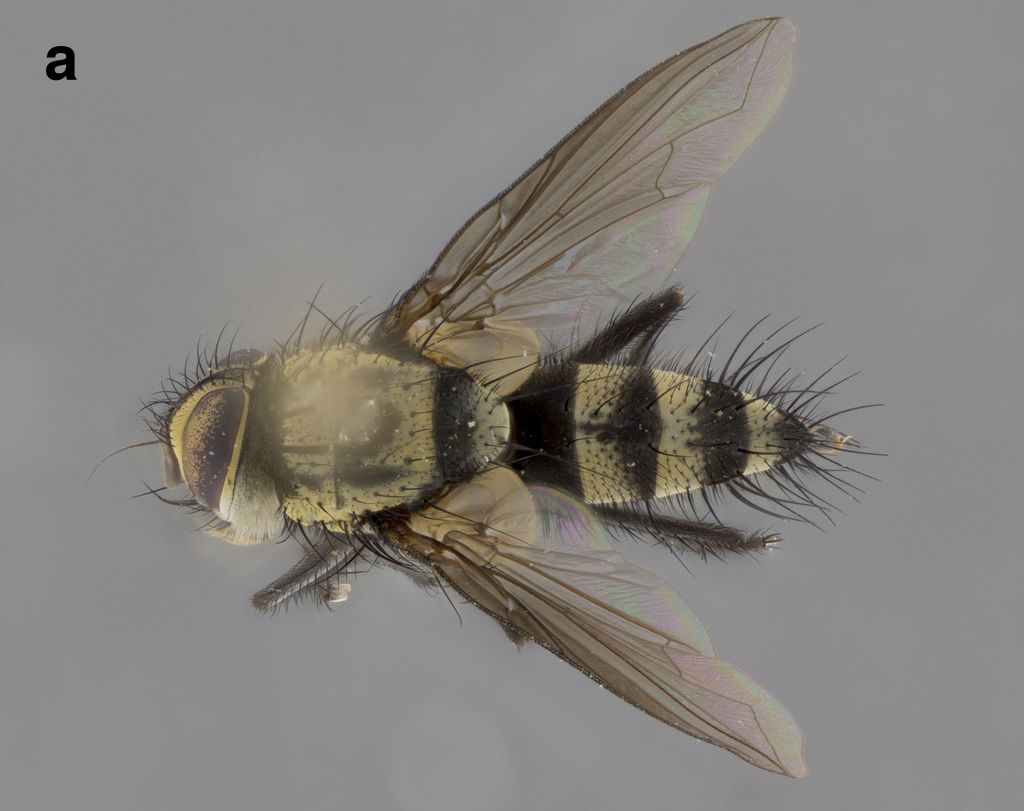
habitus in dorsal view

**Figure 34b.**
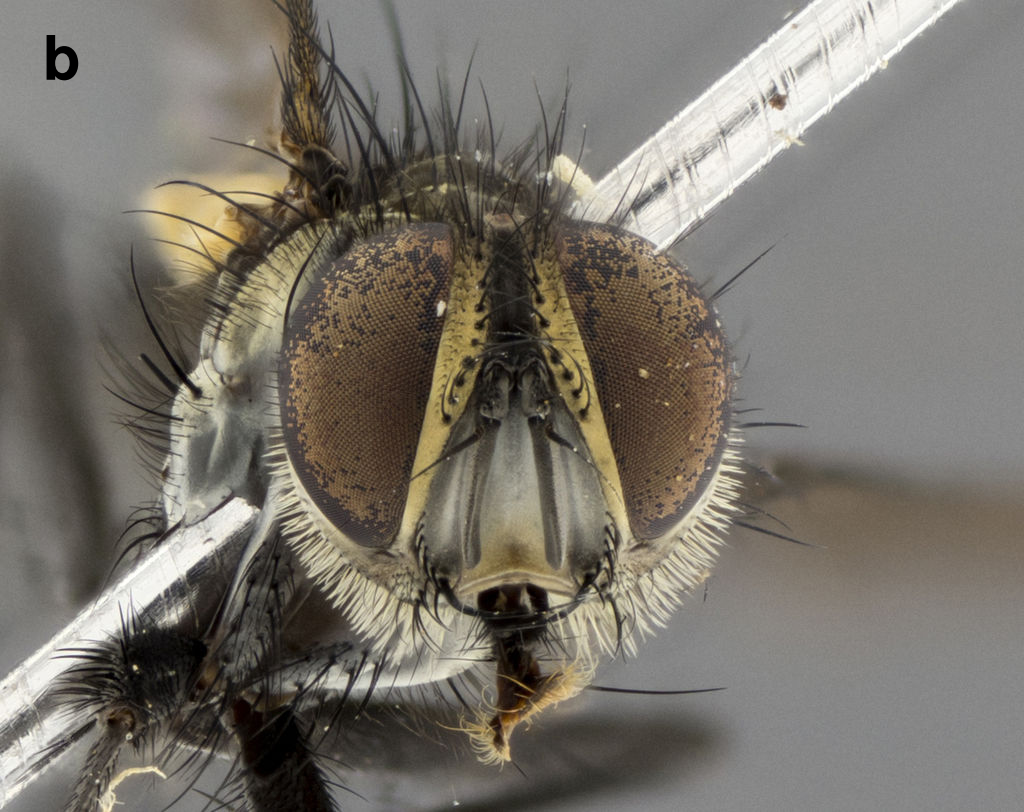
head in frontal view

**Figure 34c.**
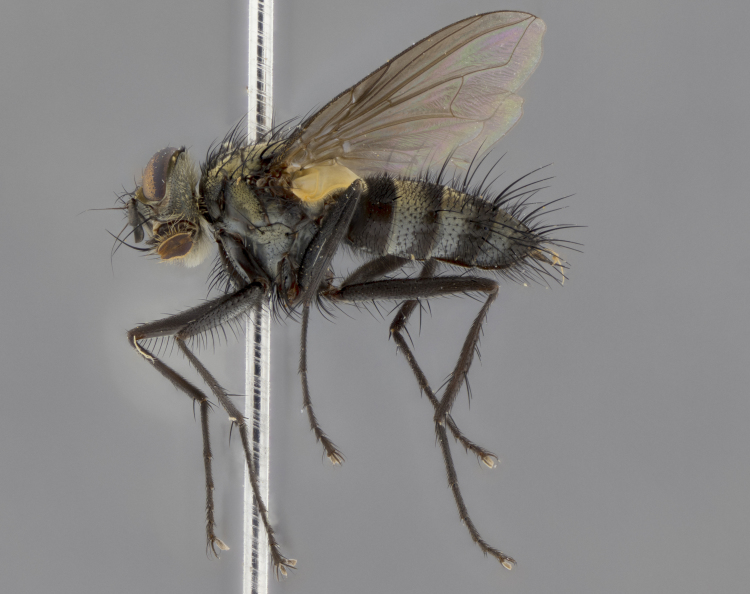
habitus in lateral view

**Figure 34d.**
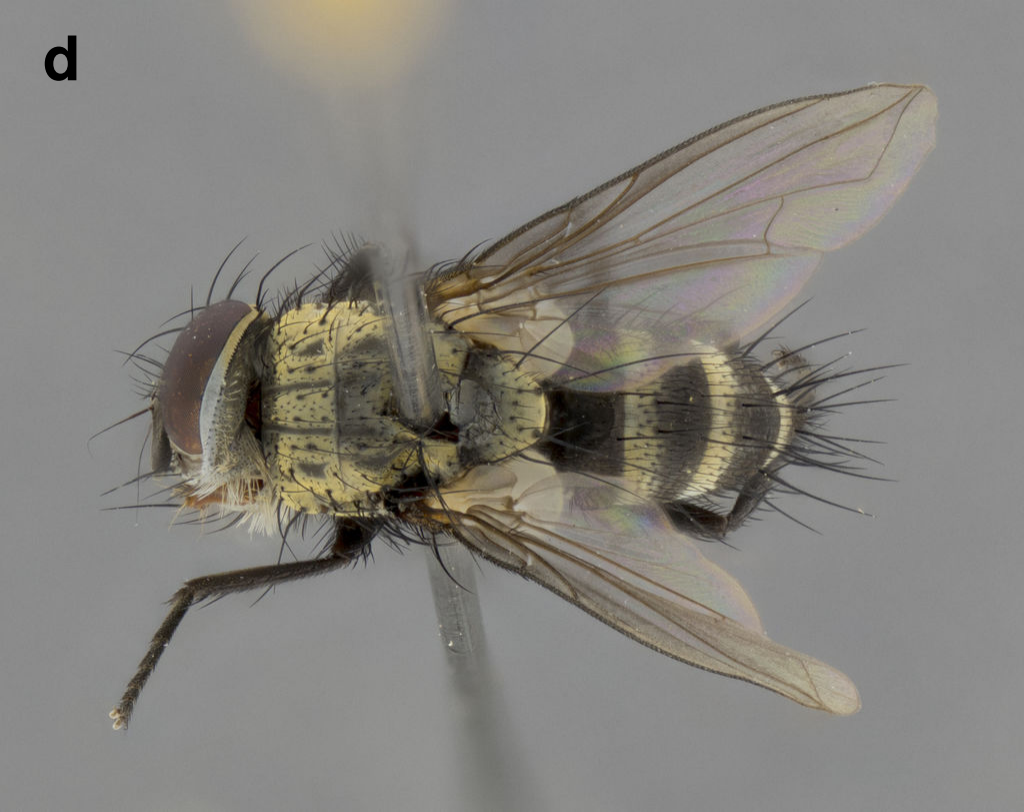
habitus in dorsal view

**Figure 34e.**
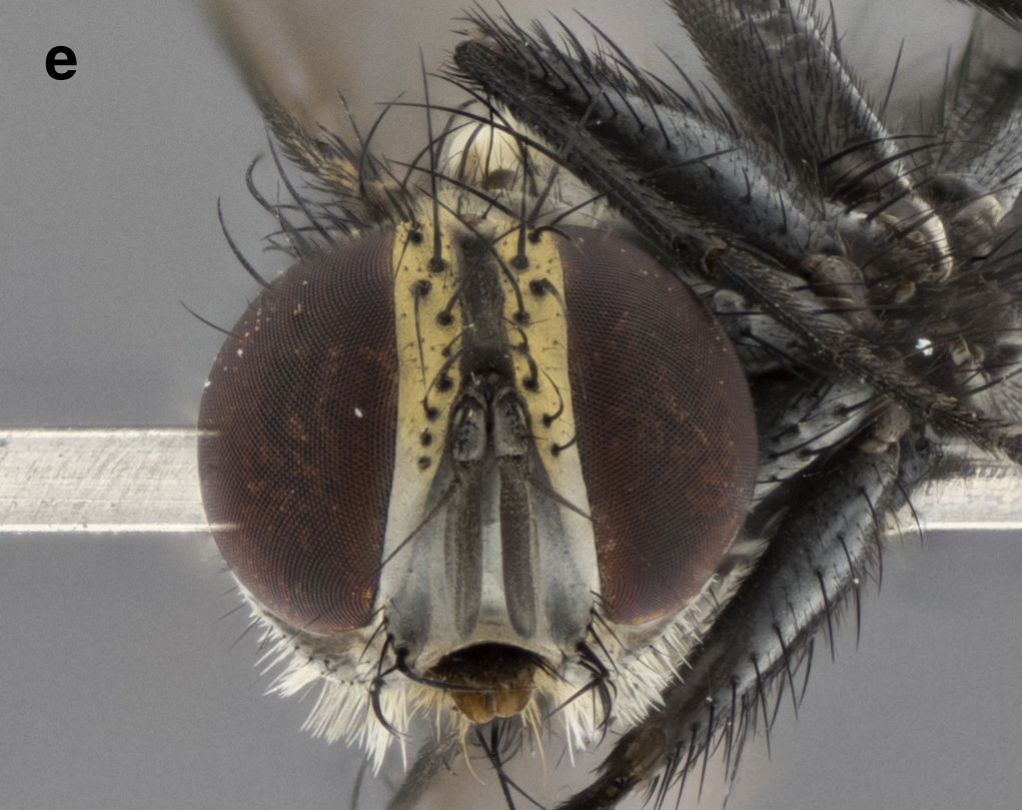
head in frontal view

**Figure 34f.**
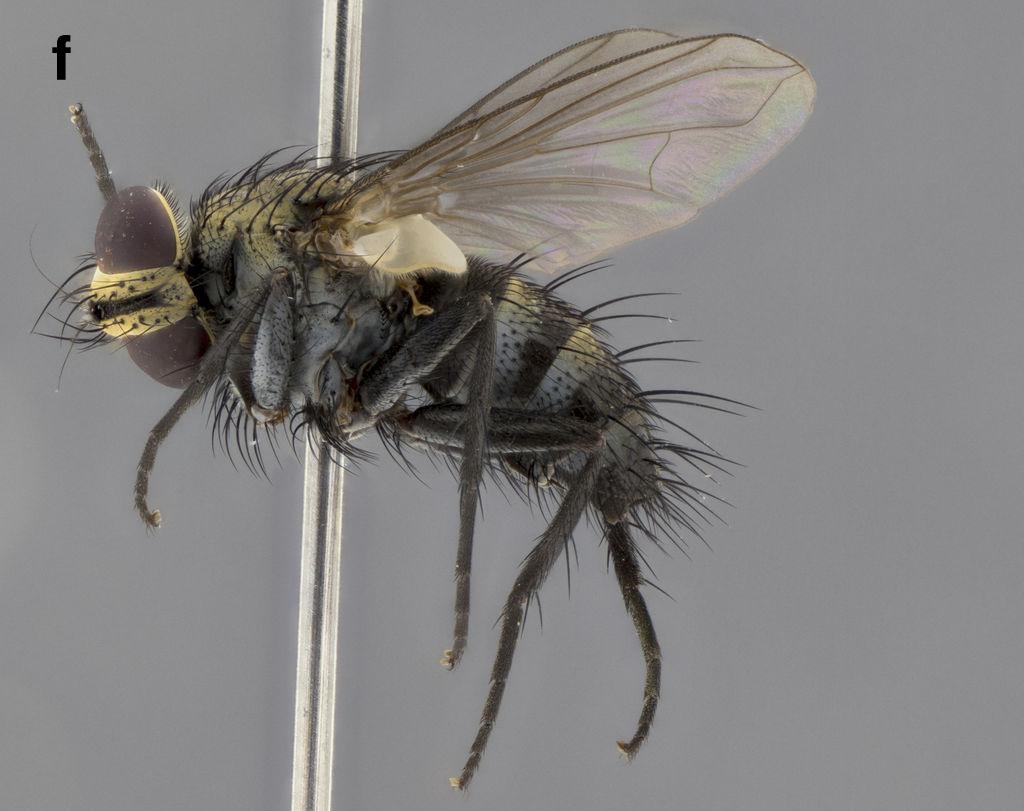
habitus in lateral view

**Figure 35a.**
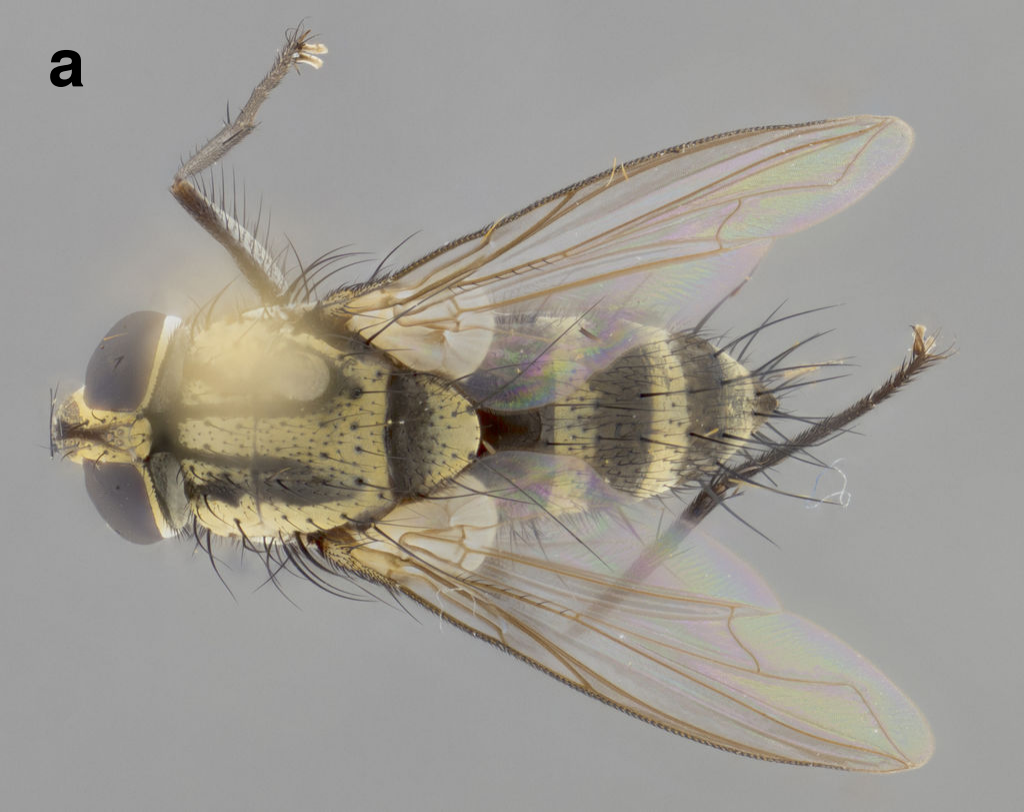
habitus in dorsal view

**Figure 35b.**
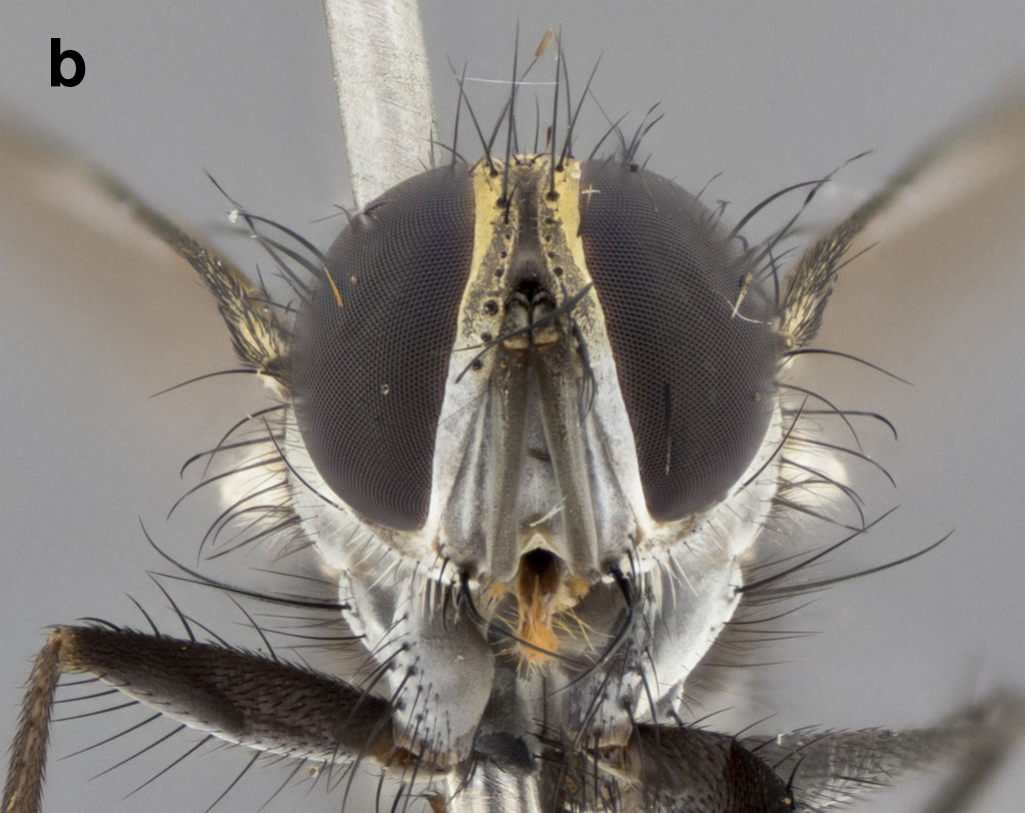
head in frontal view

**Figure 35c.**
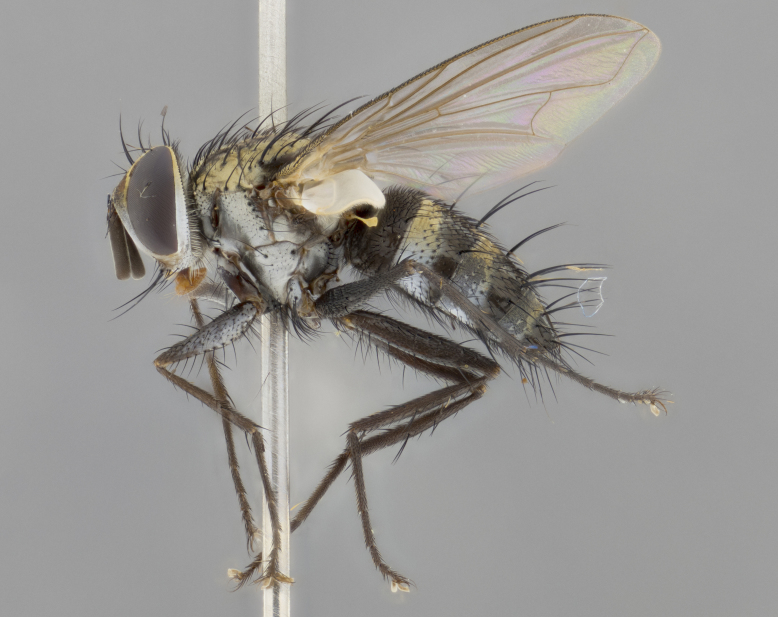
habitus in lateral view

**Figure 35d.**
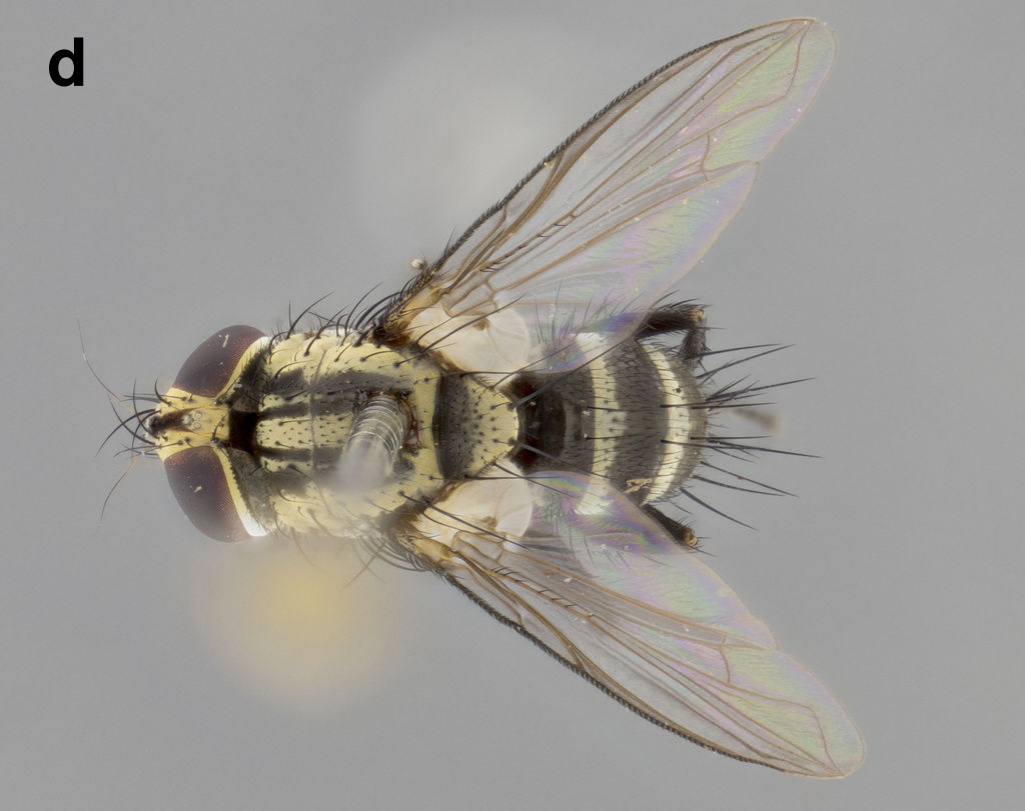
habitus in dorsal view

**Figure 35e.**
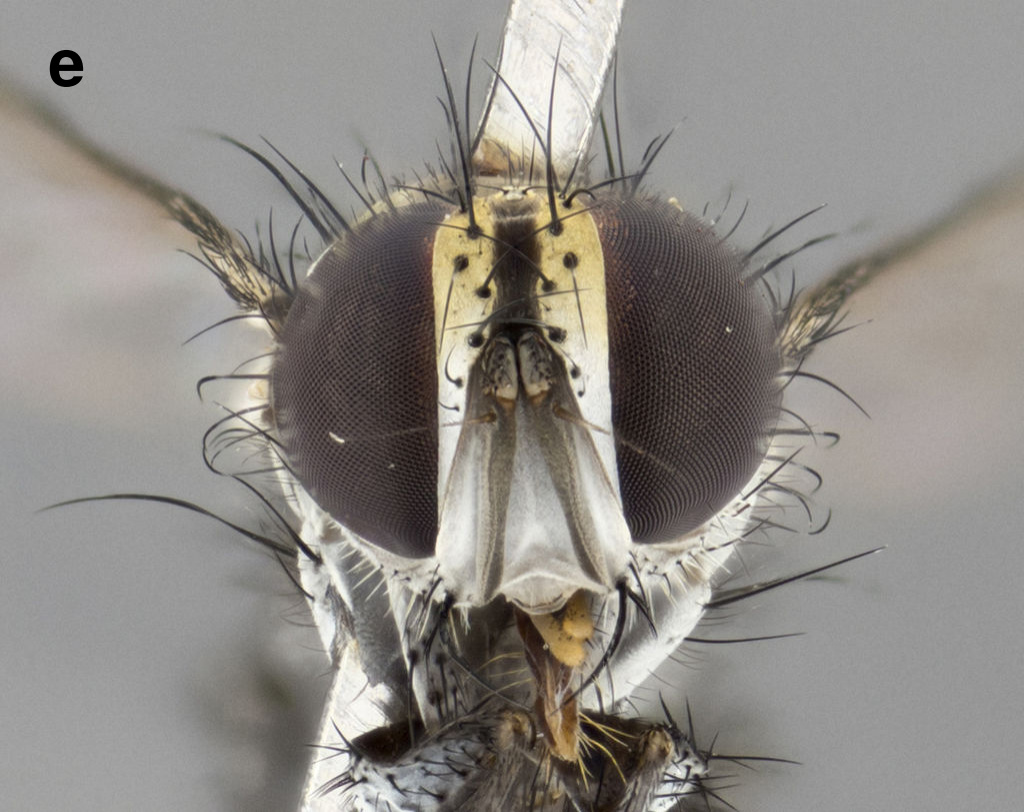
head in frontal view

**Figure 35f.**
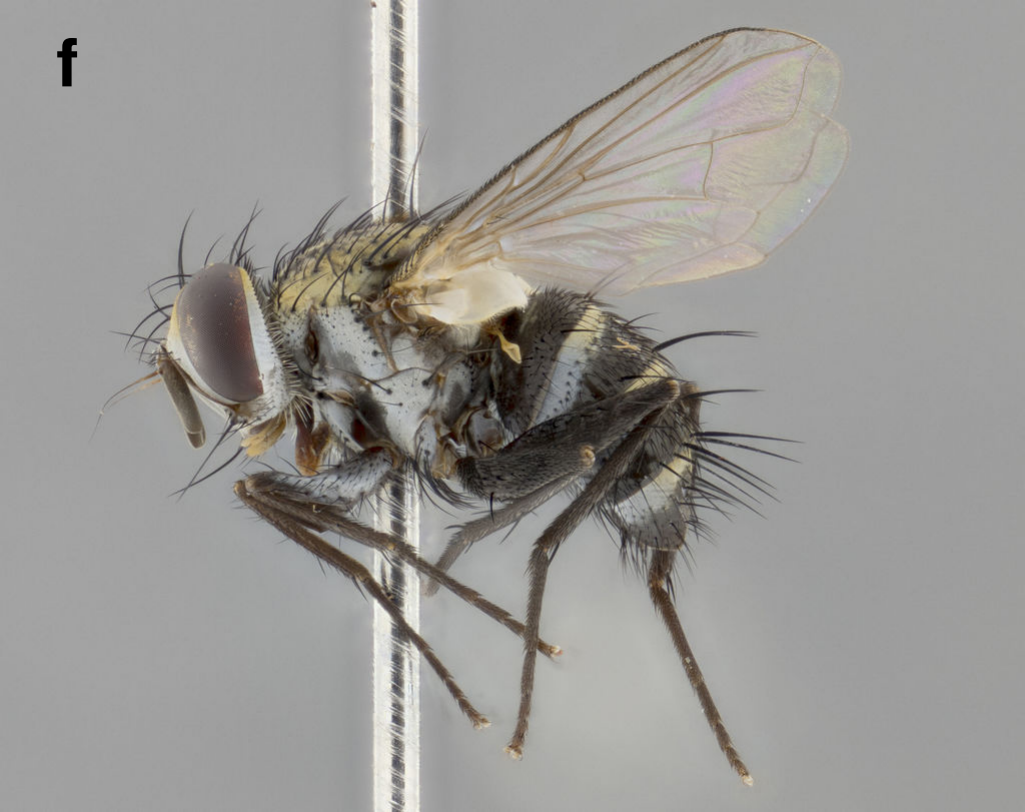
habitus in lateral

**Figure 36a.**
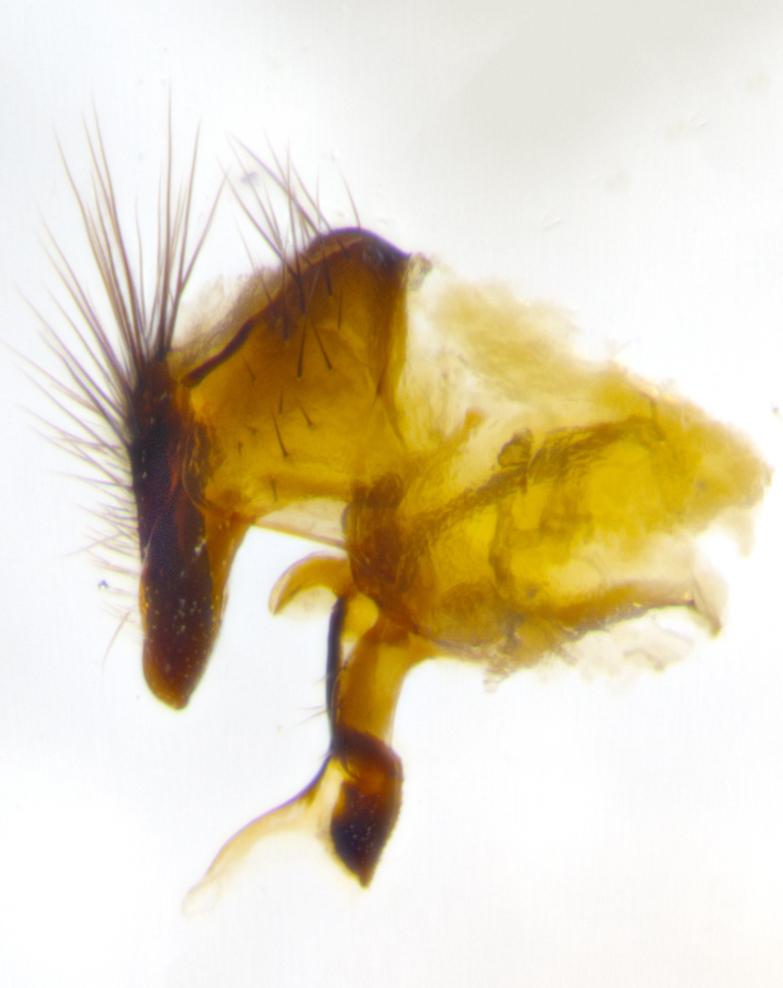
lateral view

**Figure 36b.**
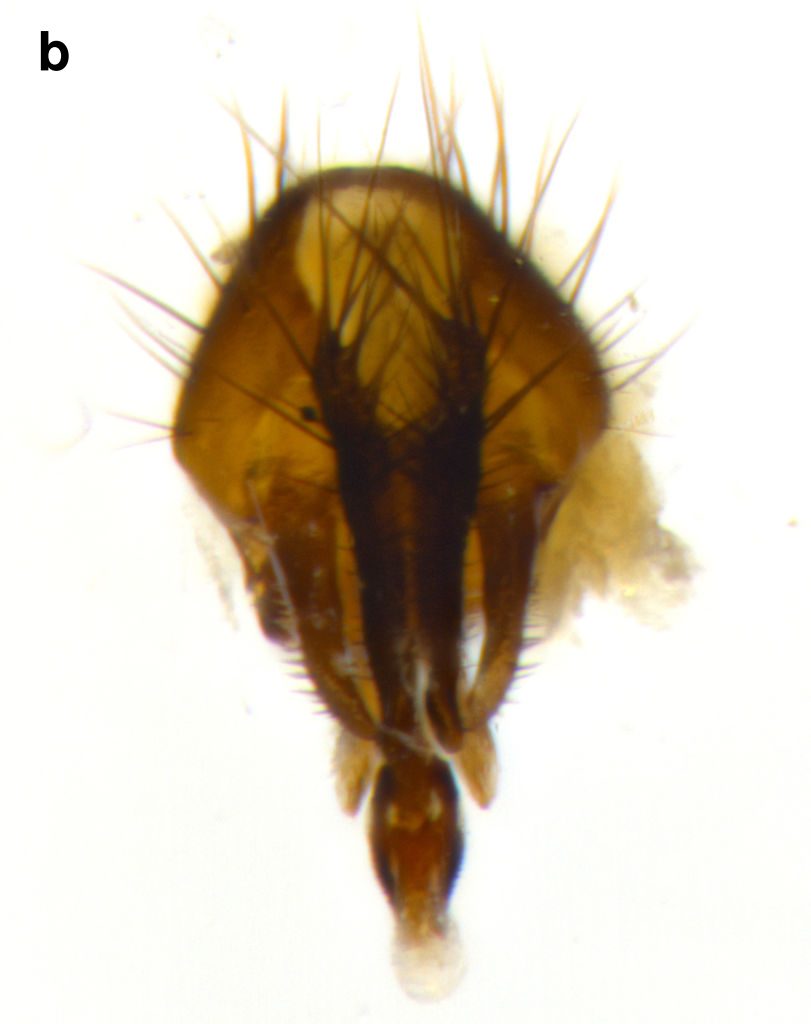
dorsal view

**Figure 36c.**
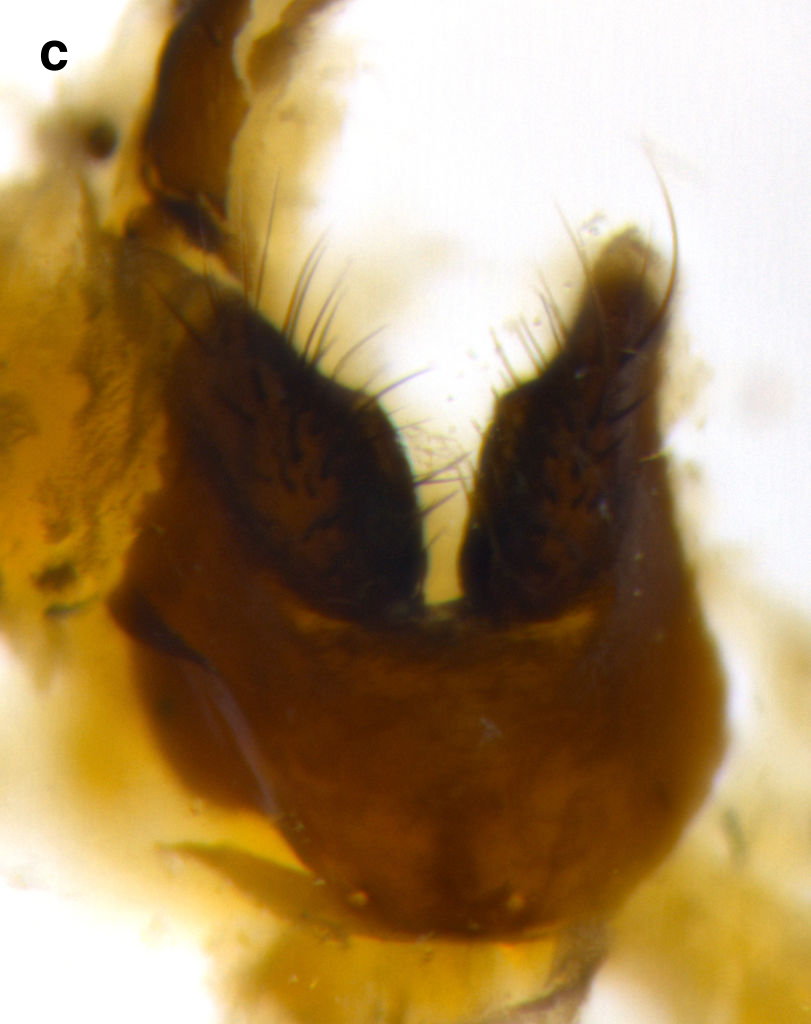
sternite 5 in ventral view

**Figure 37a.**
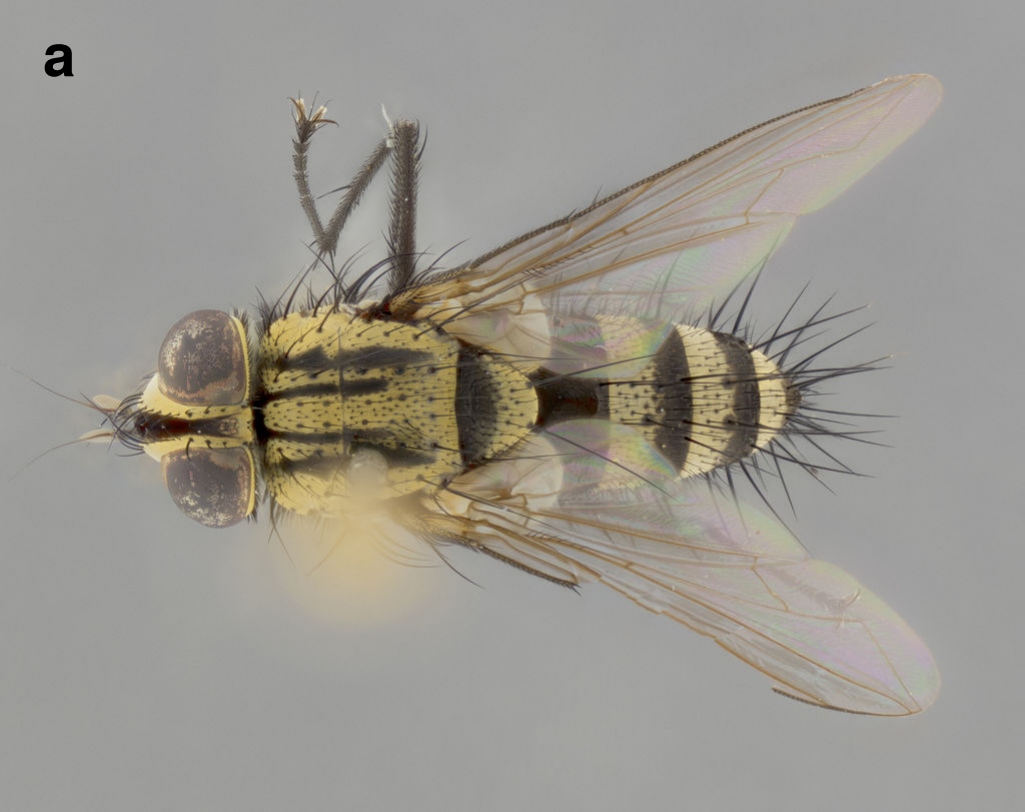
habitus in dorsal view

**Figure 37b.**
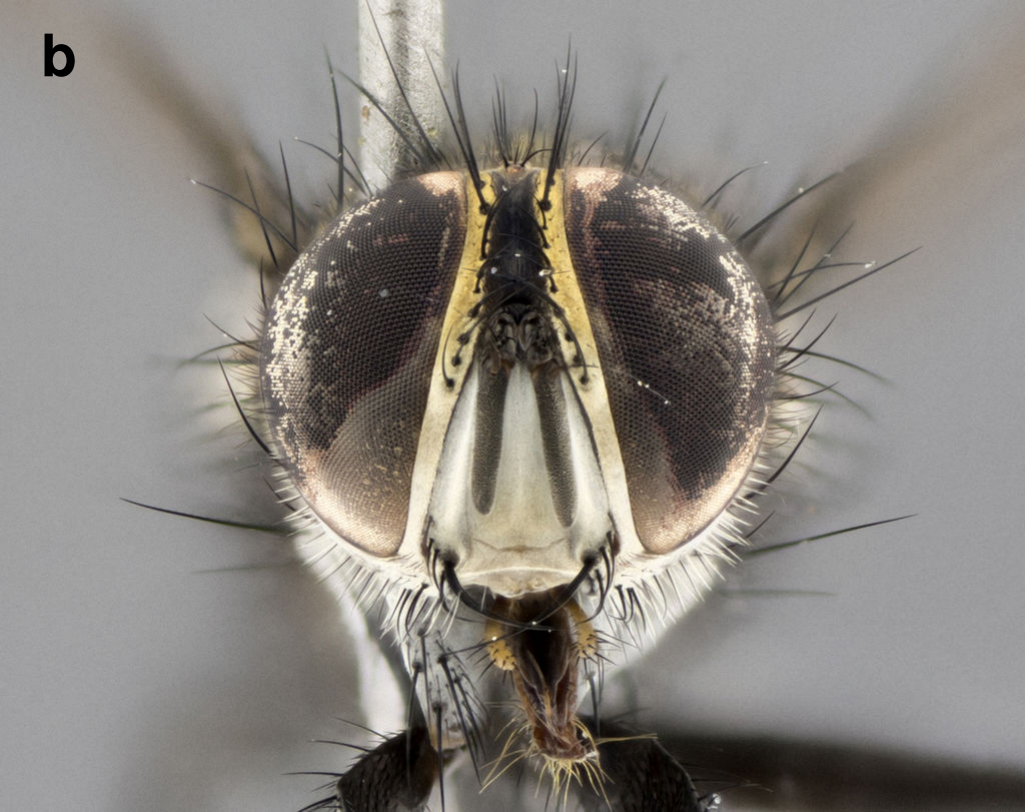
head in frotnal view

**Figure 37c.**
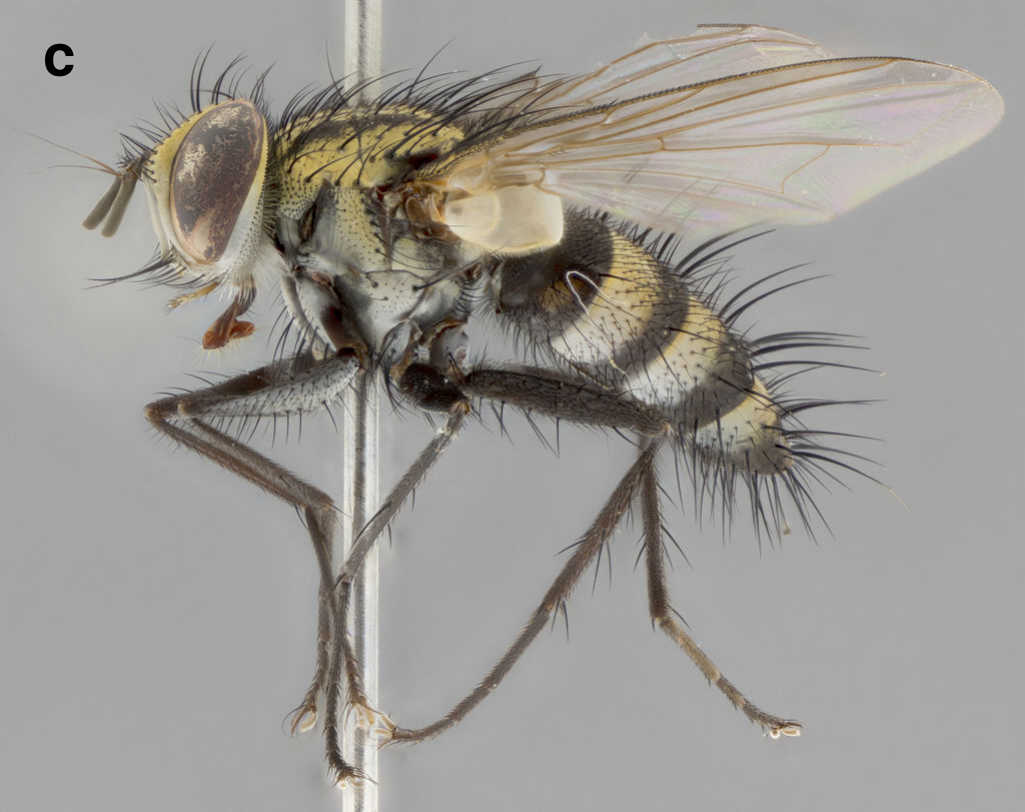
habitus in lateral view

**Figure 37d.**
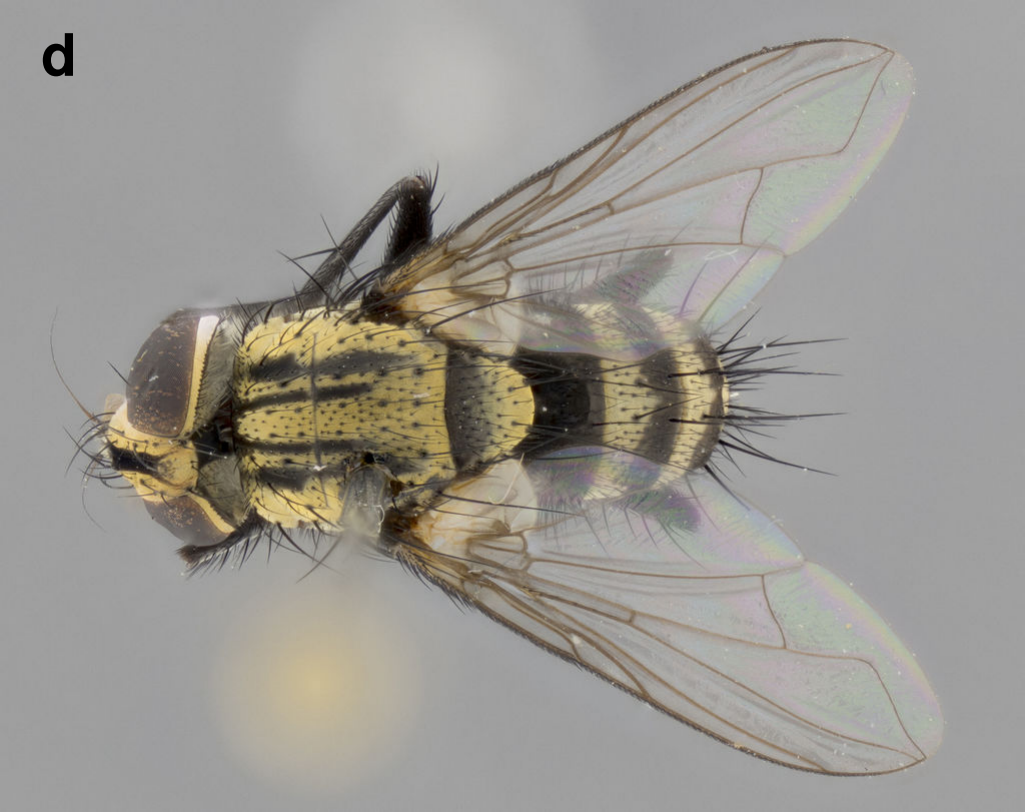
habitus in dorsal view

**Figure 37e.**
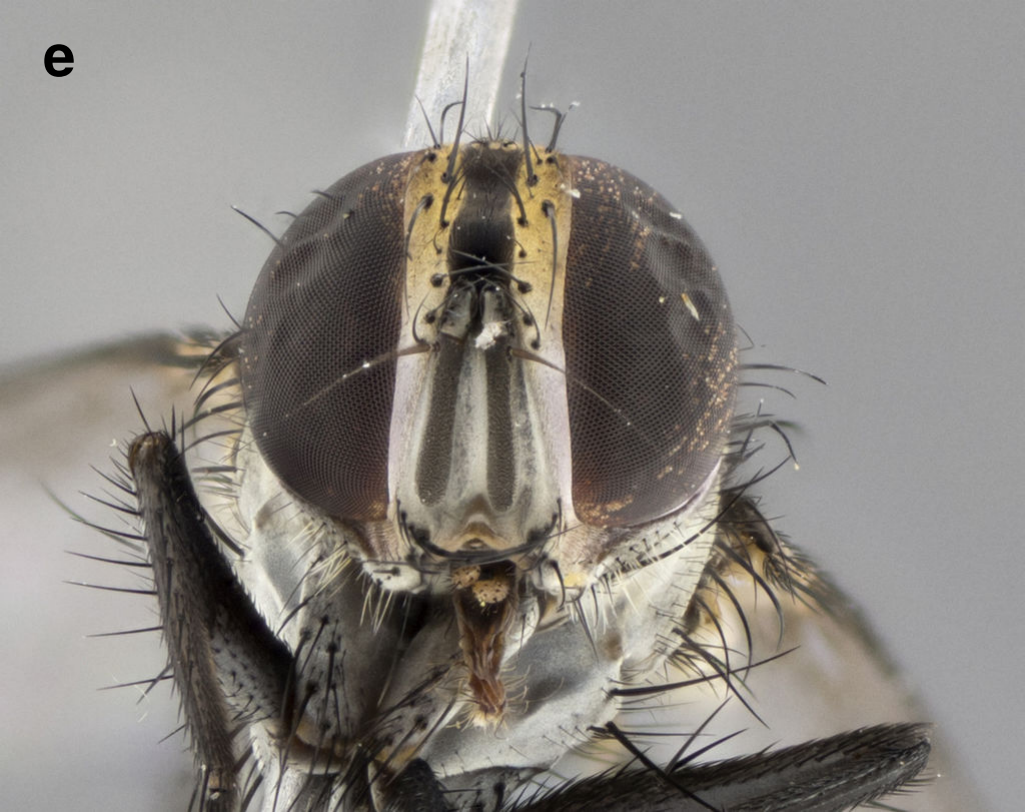
head in frontal view

**Figure 37f.**
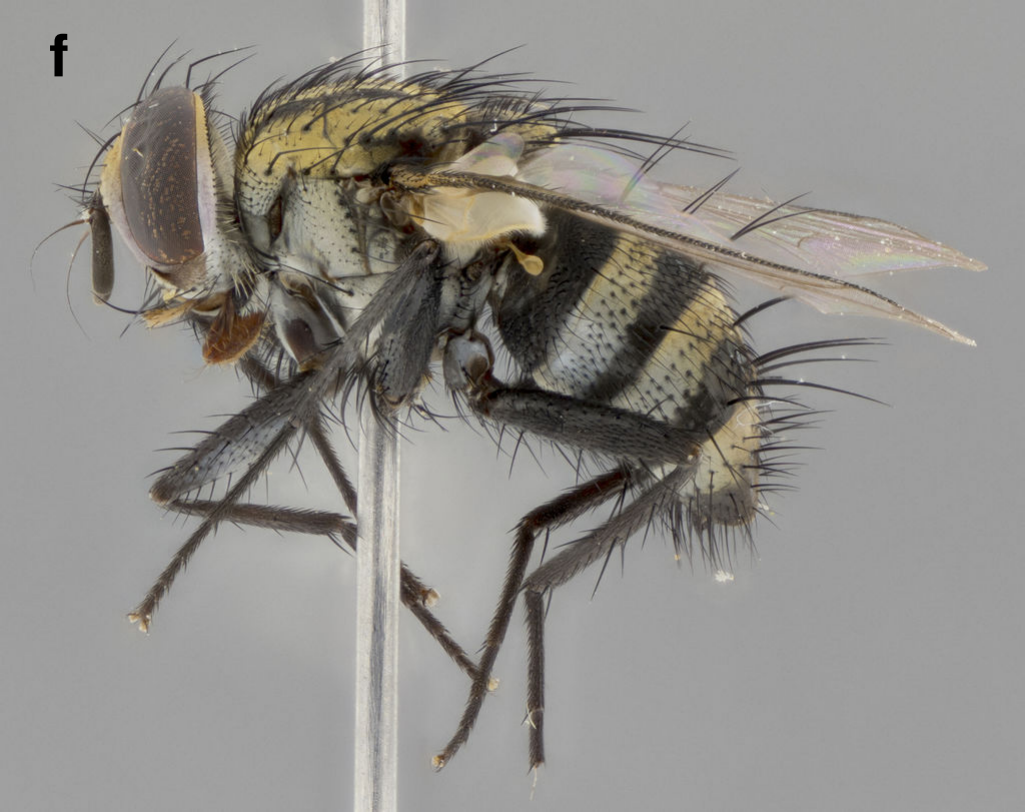
habitus in lateral view

**Figure 38a.**
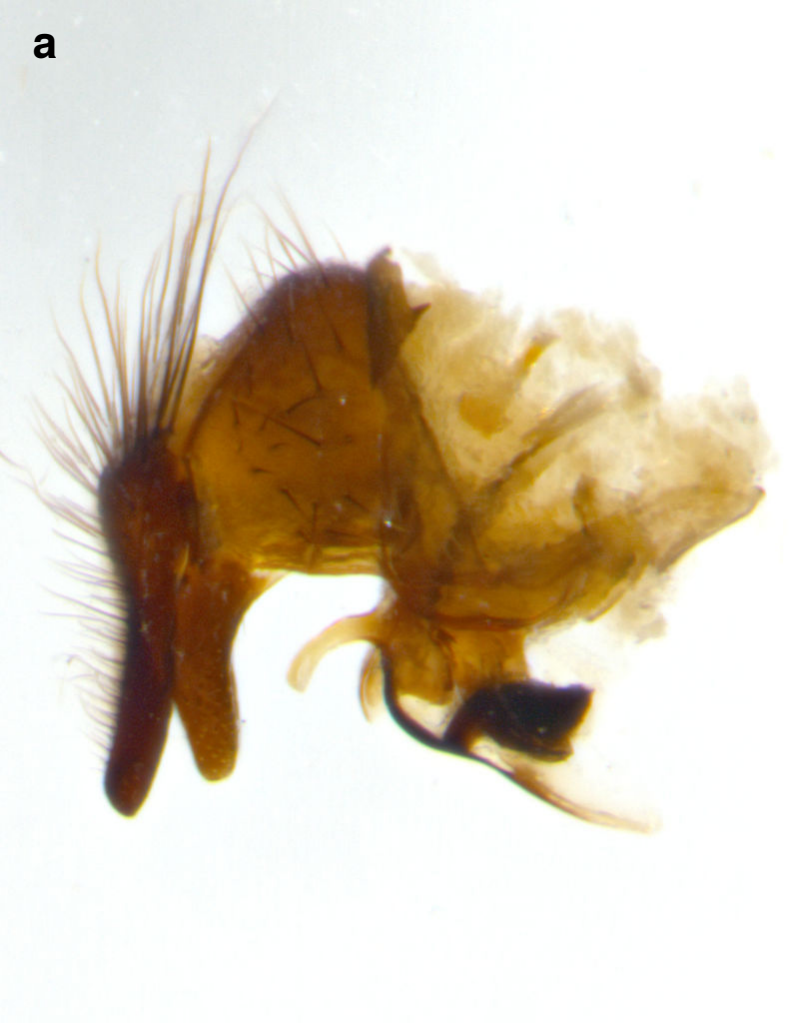
lateral view

**Figure 38b.**
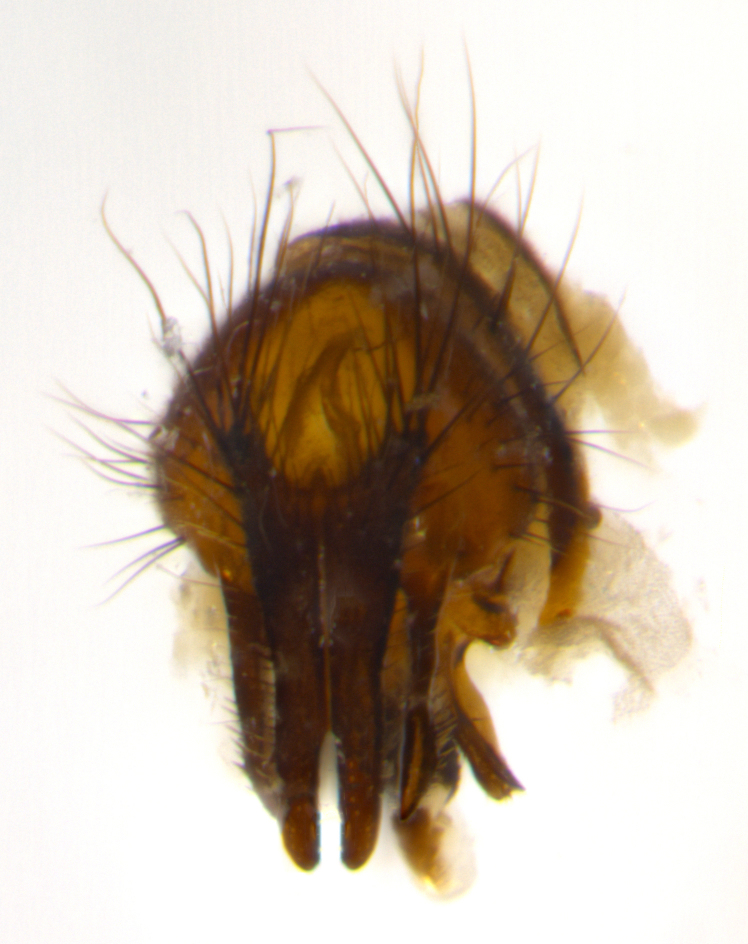
dorsal view

**Figure 38c.**
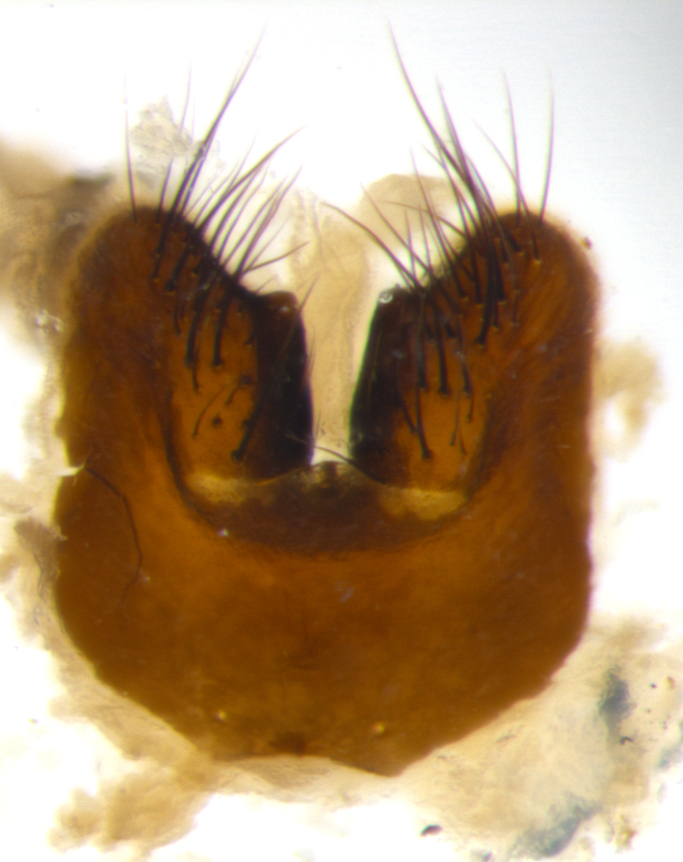
sternite 5 in ventral view
